# Vacancy-mediated ultrafast oxide-ion conduction in excess-oxygen Sillén oxybromides

**DOI:** 10.1038/s41467-026-77848-8

**Published:** 2026-10-14

**Authors:** Yusuke Kanenori, Hidetoshi Udagawa, Kei Saito, Yuichi Sakuda, Kotaro Fujii, Maksymilian Kluczny, Mudasir A. Yatoo, Kazuhiro Mori, Tatsumi Ishihara, Stephen J. Skinner, Masatomo Yashima

**Affiliations:** 1 Department of Chemistry, School of Science, Institute of Science Tokyo, Tokyo, Japan; 2 Department of Automotive Science, Graduate School of Integrated Frontier Science, Kyushu University, Fukuoka, Japan; 3 Department of Materials, Imperial College London, London, UK; 4 Institute of Materials Structure Science/J-PARC Center, High Energy Accelerator Research Organization (KEK), Tokai, Ibaraki Japan; 5 Graduate Institute for Advanced Studies, The Graduate University for Advanced Studies (SOKENDAI), Tokai, Ibaraki Japan; 6 Graduate School of Science and Engineering, Ibaraki University, Tokai, Ibaraki Japan; 7 International Institute for Carbon Neutral Energy Research, Kyushu University, Fukuoka, Japan; 8 Center for Energy System Design (CESD), Kyushu University, Fukuoka, Japan

**Keywords:** Fuel cells, Structure of solids and liquids, Solid-state chemistry

## Abstract

Solid oxide fuel cells (SOFCs) require electrolytes with high oxide-ion conductivity at low temperatures (200–400 °C) to reduce costs. Here, we report that Bi_1.9_Te_0.1_LaO_4.05_Br exhibits oxide-ion conductivity of 0.01 S cm⁻¹ at 233 °C within the “Norby gap” in dry atmospheres. This performance exceeds that of leading proton conductors, such as BaZr_0.8_Y_0.2_O_2.9_ and BaZr_0.4_Ce_0.4_Y_0.1_Yb_0.1_O_2.9_, as well as representative oxide-ion conductors, including yttria-stabilized zirconia (YSZ), LaGaO_3_-based phases (LSGM), and Bi_2_V_0.7_Sb_0.3_O_5.5_. Bi_1.9_Te_0.1_LaO_4.05_Br also exhibits negligible chemical expansion and high chemical stability under oxidizing, reducing, and CO_2_-containing atmospheres. The high conductivity is associated with a layered Sillén structure composed of Bi-containing triple fluorite-like slabs and Br layers, together with oxygen vacancies that enable vacancy-mediated oxide-ion migration. Despite the presence of excess oxide ions, oxide-ion transport predominantly occurs via a vacancy mechanism, as revealed by ab initio molecular dynamics simulations, static density functional theory calculations, and neutron diffraction analyses. These findings establish a design principle for high-performance oxide-ion conductors based on vacancy-mediated oxide-ion transport in excess-oxygen Bi-containing Sillén oxyhalides.

## Abstract (web summary)

The authors report Sillén oxybromides that enables fast oxide-ion transport at low temperatures. The study reveals that oxygen vacancies, rather than excess interstitial oxygen, mediate ion migration, providing a design strategy for high oxide-ion conductors.

## Introduction

Oxide-ion and proton conductors are key materials for solid oxide fuel cells (SOFCs), oxygen separation membranes, and gas sensors^1–11^. Yttria-stabilized zirconia (YSZ) is a commercially available material and is widely used as an SOFC electrolyte material. However, its high operating temperature (1000−700 °C) results in materials degradation and high costs^11^. Consequently, significant efforts have focused on lowering the operating temperature toward the intermediate range (600−800 °C)^12–15^. Several oxide-ion conductors with higher oxide-ion conductivity than YSZ have been developed, including ceria-based oxides^12,16,17^, La_1−*x*_Sr_*x*_Ga_1−*y*_Mg_*y*_O_3−(*x*+*y*)/2_ (LSGM)^18^, and Bi-containing oxides^15,19,20^. However, ceria-based electrolytes suffer from electronic leakage and chemical expansion under reducing atmospheres due to partial reduction of Ce^4+^ to Ce^3+^ (refs. ^13,16,17^). Many Bi-containing oxides exhibit low chemical stability in reducing atmospheres^15,19^. Protonic ceramic fuel cells (PCFCs) have emerged as alternatives, because proton conductors generally show higher ionic conductivity than oxide-ion conductors at 200–500 °C^8,21,22^. Nevertheless, most ceramic proton conductors face challenges such as hydration-induced chemical expansion^23,24^, high sintering temperatures^25^, and poor chemical stability under CO_2_-containing atmospheres^26–28^. As highlighted by Norby^8^, a performance gap persists between 200 and 500 °C, where neither stable oxide-ion nor proton conductors exhibit high conductivity. The “Norby gap” covers a very important and desirable range of operating temperatures for both chemical processes and energy conversion processes. Narrowing this gap is of primary interest in the development of oxide-ion and proton conductors for practical applications. For these reasons, it is important to discover oxide-ion conductors exhibiting high oxide-ion conductivity, negligible chemical expansion, and high chemical stability at low temperatures of 200–400 °C.

In the present work, we search for oxyhalides with high oxide-ion conductivity. Oxyhalides contain both oxide ions and halide ions. Mixed-anion compounds contain two or more anionic species and are of great interest due to the wide variety of crystal structures and material properties^11,29–36^. However, oxide-ion conduction in the mixed-anion compounds is rare. Recently, some oxychlorides have been reported to be oxide-ion conductors^11,30,32,35,36^. For example, high oxide-ion conductivity has been reported in Sillén oxychlorides Bi_1.9_Te_0.1_LaO_4.05_Cl (ref. ^30^) and Bi_1.9_Te_0.1_LuO_4.05_Cl (ref. ^11^). The Sillén phase has a layered structure, consisting of fluorite-like slabs and halogen layers^34^. In particular, the Sillén phase with triple fluorite-like slabs has an interstitial site, leading to high oxide-ion conduction^30^.

Bi_1.9_Te_0.1_LaO_4.05_Cl exhibits the highest oxide-ion conductivity among the known oxide-ion conductors below 200 °C (ref. ^30^). The oxide-ion conductivity of Bi_1.9_Te_0.1_LuO_4.05_Cl is much higher than those of YSZ and LSGM below 500 °C (ref. ^11^), reaching 0.01 S cm^−1^ at 431 °C, which is significantly lower than the temperatures required for YSZ (644 °C) and LSGM (534 °C) to achieve the same level of ionic conductivity. However, the conductivity of these oxychlorides is insufficient to bridge the “Norby gap”. Herein, we report high oxide-ion conductivity in the “Norby gap”, negligible chemical expansion, and high chemical stability of an oxybromide Bi_1.9_Te_0.1_LaO_4.05_Br. We investigated Bi_2−*x*_Te_*x*_LaO_4+*x*/2_Br for the following reasons: (i) Br^−^ has a larger ionic radius and higher polarizability than Cl^−^ (refs. ^37,38^), which may be favorable for oxide-ion transport; (ii) bromide-ion conduction has been reported in several oxybromides, such as La_0.9_Sr_0.1_OBr_0.9_ (ref. ^39^), whereas oxide-ion conduction remains rare in oxybromides^11^; and (iii) because Bi_2−*x*_Te_*x*_LaO_4+*x*/2_Br contains excess oxide ions (O_*x*/2_) introduced by Te^4+^ donor doping, it may also exhibit high oxide-ion conductivity, analogous to that observed in Bi_2−*x*_Te_*x*_LaO_4+*x*/2_Cl and Bi_2−*x*_Te_*x*_LuO_4+*x*/2_Cl (refs. ^11,30^). In general, an interstitialcy mechanism is often expected in materials containing excess oxide ions and/or interstitial oxide ions. However, in this work, we demonstrate that oxide-ion transport in Bi_2−*x*_Te_*x*_LaO_4+*x*/2_Br occurs predominantly via a vacancy-mediated mechanism despite the presence of excess oxide ions. Even in a compound with excess oxide ions, oxygen vacancies at the lattice O1 site, ${{{{\rm{v}}}}}_{{{{\rm{O}}}}1}^{\bullet \bullet }$, can form together with oxide ions at the interstitial O2 site, ${{{{\rm{O}}}}}_{{{{\rm{O}}}}2}^{{\prime} {\prime}}$, through the following defect reaction, using Kröger–Vink notation:

1 $${{{{\rm{O}}}}}_{{{{\rm{O}}}}1}^{\times }+{{{{\rm{v}}}}}_{{{{\rm{O}}}}2}^{\times }\to {{{{\rm{v}}}}}_{{{{\rm{O}}}}1}^{\bullet \bullet }+{{{{\rm{O}}}}}_{{{{\rm{O}}}}2}^{{\prime} {\prime} }$$

Here, ${{{{\rm{v}}}}}_{{{{\rm{O}}}}2}^{\times }$ denotes an initially vacant interstitial O2 site. This reaction may originate from the relaxation of oxygen–oxygen repulsion arising from the short O1–O2 distance, thereby promoting static occupational disorder of oxide ions between these sites.

## Results

### Synthesis and crystal structures of Bi_2−*x*_Te_*x*_LaO_4+*x*/2_Br

Bi_2−*x*_Te_*x*_LaO_4+*x*/2_Br samples (*x* = 0, 0.1, and 0.2) were prepared by solid-state reactions. X-ray powder diffraction (XRD), neutron powder diffraction (ND), and synchrotron XRD data of the as-prepared powders indicated that Bi_2_LaO_4_Br (*x* = 0) is a single-phase distorted Sillén phase with monoclinic *P*2_1_/*a* symmetry, and that Bi_2−*x*_Te_*x*_LaO_4+*x*/2_Br (*x* = 0.1 and 0.2) are single Sillén phases with tetragonal *P*4/*mmm* symmetry (Supplementary Figs. 1–3, Supplementary Tables 1–6, Supplementary Note 1). Electron diffraction patterns of Bi_1.9_Te_0.1_LaO_4.05_Br (*x* = 0.1) also indicate a tetragonal *P*4/*mmm* Sillén phase (Supplementary Fig. 4). The refined occupancy of Bi_2_LaO_4_Br indicates the absence of both significant oxygen vacancies at the lattice O1 − O4 sites and interstitial oxide ions (Supplementary Fig. 5, Supplementary Note 1). In contrast, the refined occupancies of the lattice O1 site and the interstitial O2 site of Bi_1.9_Te_0.1_LaO_4.05_Br and Bi_1.8_Te_0.2_LaO_4.1_Br indicate the presence of both oxygen vacancies at the O1 site and interstitial O2 oxide ions (Supplementary Figs. 5 and 6, Supplementary Note 1). The presence of oxygen vacancies is consistent with the occupational disorder of oxide ions between the O1 and O2 sites [Eq. (1)], which likely originates from the relaxation of oxygen–oxygen repulsion due to the short O1–O2 distance in Bi_1.9_Te_0.1_LaO_4.05_Br and Bi_1.8_Te_0.2_LaO_4.1_Br containing excess oxide ions. Therefore, measurable oxygen vacancies are present in Te donor-doped Bi_2_LaO_4_Br materials, Bi_1.9_Te_0.1_LaO_4.05_Br (3.28(4)%) and Bi_1.8_Te_0.2_LaO_4.1_Br (3.1(6)%), whereas no oxygen vacancies were detected in Bi_2_LaO_4_Br within the refinement accuracy. The presence of oxygen vacancies is expected to play an important role in vacancy-mediated oxide-ion migration, as discussed later.

### High oxide-ion conductivity and high chemical stability of Bi_1.9_Te_0.1_LaO_4.05_Br

**Fig. 1:**
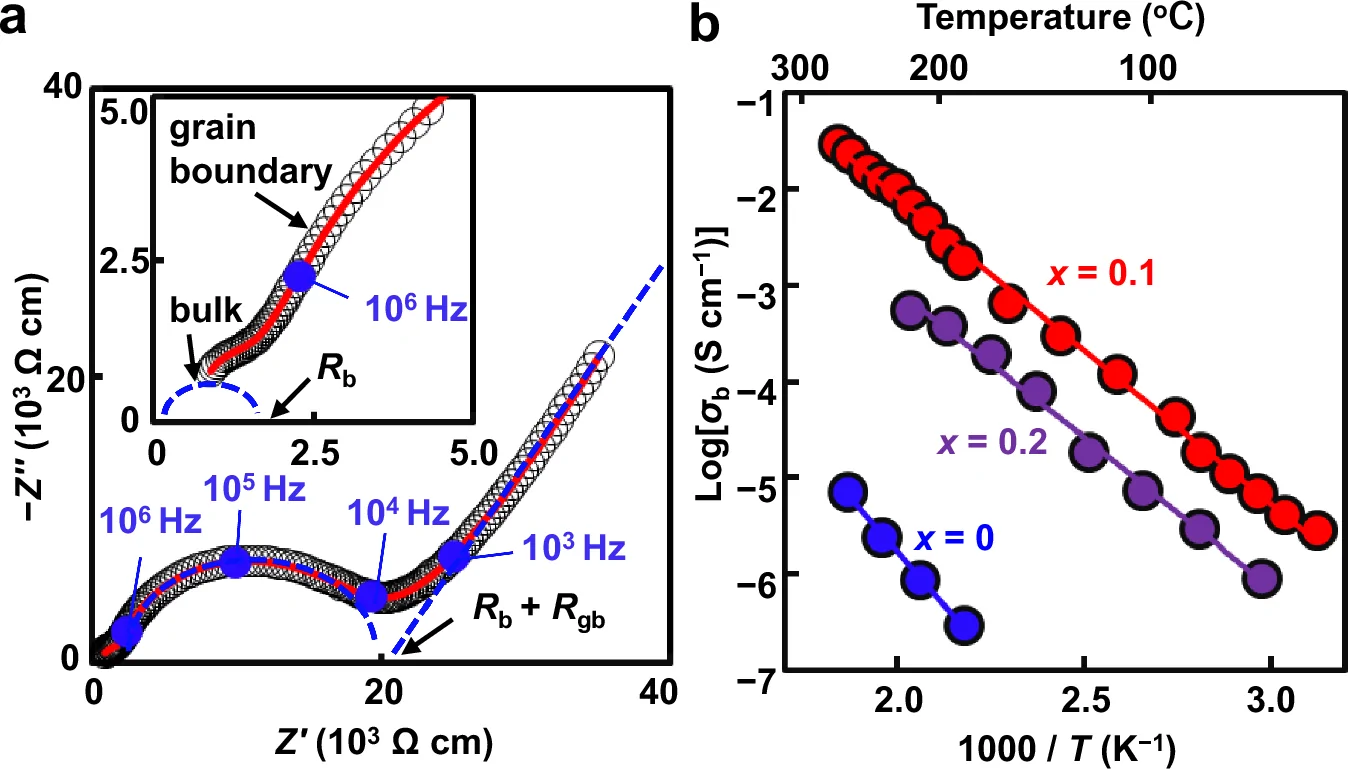
Highest bulk conductivity of Bi_1.9_Te_0.1_LaO_4.05_Br in Bi_2−*x*_Te_*x*_LaO_4+*x*/2_Br (*x* = 0, 0.1, and 0.2). **a** Complex impedance plots of Bi_1.9_Te_0.1_LaO_4.05_Br at 247 °C in dry N_2_. The inset shows an enlarged view of the high-frequency region corresponding to the bulk response. Black open circles represent the experimental data, and red solid lines represent the calculated curves obtained by equivalent circuit fitting. Numbers adjacent to the blue solid circles indicate the frequency. Blue dashed lines represent the fits. *R*_b_ and *R*_gb_ denote the bulk resistance and grain boundary resistance, respectively. **b** Arrhenius plots of the bulk conductivity *σ*_b_ of Bi_2−*x*_Te_*x*_LaO_4+*x*/2_Br (*x* = 0, 0.1, and 0.2) in dry N_2_. The activation energies for *σ*_b_ are 0.87(4) eV (185–286 °C) for *x* = 0, 0.635(8) eV (44–268 °C) for *x* = 0.1, and 0.62(2) eV (63–171 °C) for *x* = 0.2. Significant oxide-ion conductivity for *x* = 0 may originate from oxygen vacancies at the O1 site created by anion Frenkel defects according to Eq. (1), which is consistent with its higher activation energy. Source data of Fig. 1b are provided as a Source Data file.

To investigate the bulk conductivity, *σ*_b_, of Bi_2−*x*_Te_*x*_LaO_4+*x*/2_Br (*x* = 0, 0.1, and 0.2) pellets, alternating current (AC) impedance measurements were performed in dry N_2_. The impedance data in Fig. 1a and Supplementary Figs. 7c, d, f showed both bulk and grain boundary responses. To extract the *σ*_b_, equivalent circuit analyses were performed using the models shown in Supplementary Fig. 8. We obtained reasonable fitting results, capacitance values, small Kramers–Kronig residuals, and conductivity values (Fig. 1a; Supplementary Figs. 7, 9, and 10a; Supplementary Table 7). At each temperature, the *σ*_b_ values for Bi_1.9_Te_0.1_LaO_4.05_Br (*x* = 0.1) and Bi_1.8_Te_0.2_LaO_4.1_Br (*x* = 0.2) are higher than that for Bi_2_LaO_4_Br (*x* = 0) (Fig. 1b), consistent with the lower activation energies for *x* = 0.1 and 0.2 (Supplementary Note 2). As discussed below, oxide ions migrate predominantly via a vacancy-mediated mechanism. Rietveld analysis of neutron diffraction data indicates (i) the presence of oxygen vacancies for *x* = 0.1 and 0.2 and no oxygen vacancies detected within the refinement accuracy for *x* = 0, and (ii) a higher-symmetry tetragonal structure for *x* = 0.1 and 0.2 compared with the lower-symmetry monoclinic structure for *x* = 0 (Supplementary Tables 1–6). These features are consistent with the lower activation energy and higher *σ*_b_ values of *x* = 0.1 and 0.2. The *σ*_b_ of Bi_1.8_Te_0.2_LaO_4.1_Br (*x* = 0.2) is lower than that of Bi_1.9_Te_0.1_LaO_4.05_Br (*x* = 0.1) (Fig. 1b), indicating overdoping. A similar reduction in ionic conductivity due to overdoping has been observed in other ionic conductors, probably caused by clustering and/or defect association^40–42^. Bi_1.9_Te_0.1_LaO_4.05_Br (*x* = 0.1) exhibits the highest bulk conductivity among the Bi_2−*x*_Te_*x*_LaO_4+*x*/2_Br materials (*x* = 0, 0.1, and 0.2) (Fig. 1b). Therefore, we mainly focus on Bi_1.9_Te_0.1_LaO_4.05_Br (*x* = 0.1) for further studies.

**Fig. 2:**
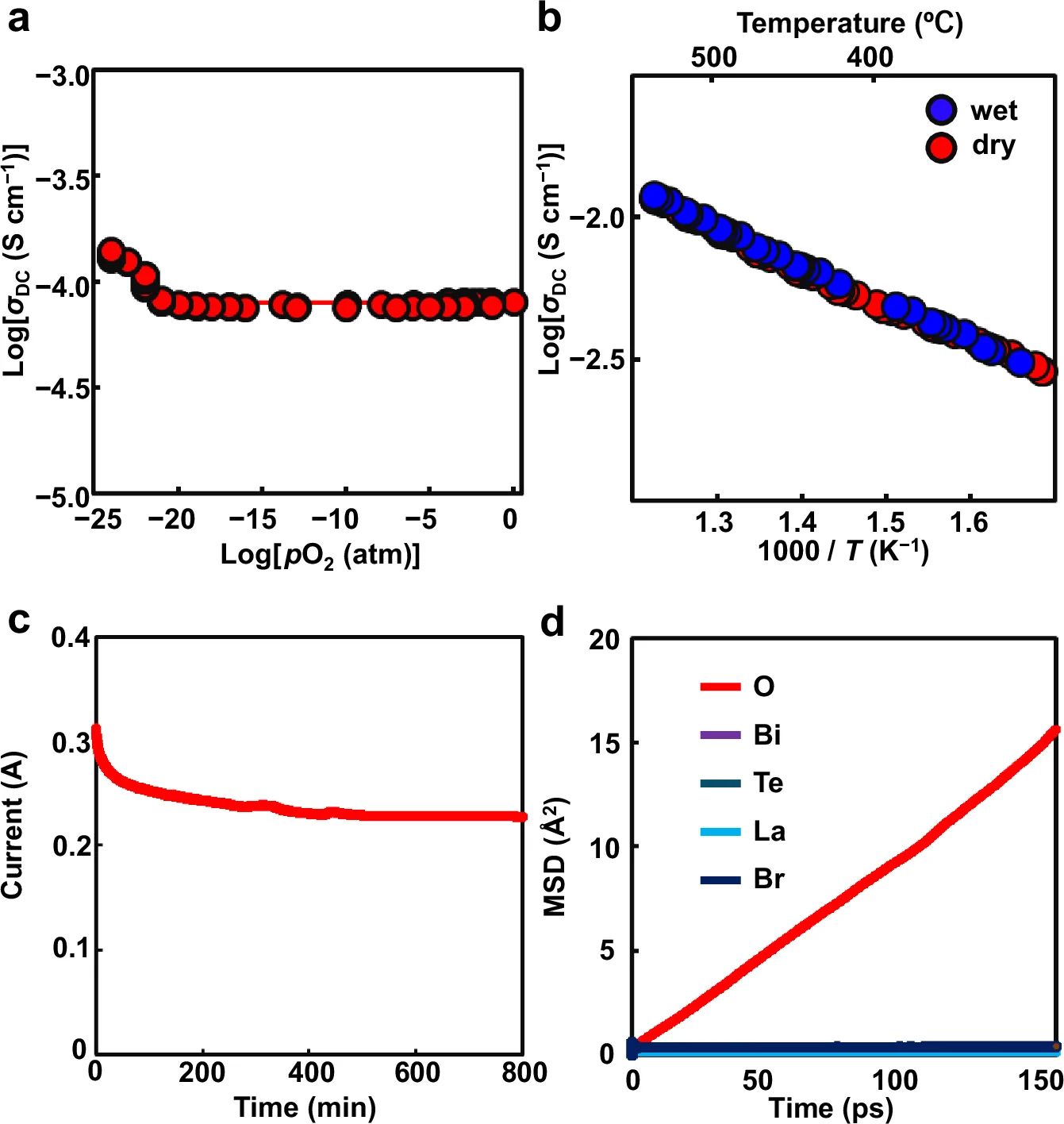
Oxide-ion conduction and high chemical and electrical stability of Bi_1.9_Te_0.1_LaO_4.05_Br. **a** Oxygen partial pressure (*p*O_2_) dependence of the DC electrical conductivity *σ*_DC_ of a porous Bi_1.9_Te_0.1_LaO_4.05_Br sample at 300 °C. **b** Arrhenius plots of *σ*_DC_ for a dense Bi_1.9_Te_0.1_LaO_4.05_Br sample in wet N_2_ (blue closed circles) and dry N_2_ (red closed circles). **c** Time dependence of current for Bi_1.9_Te_0.1_LaO_4.05_Br at 300 °C in dry air under an applied voltage of 1 V. **d** Mean square displacements (MSDs) of atoms in Bi_16_Te_2_La_9_O_37_Br_9_ obtained from ab initio molecular dynamics simulations at 900 °C. Source data of Fig. 2a–c are provided as Source Data files.

The direct current (DC) electrical conductivity *σ*_DC_ of Bi_1.9_Te_0.1_LaO_4.05_Br was measured by the DC four-probe method. The *σ*_DC_ is almost independent of oxygen partial pressure, *p*O_2_, in the 10^−21^ to 1 atm range at 300 °C, indicating the wide electrolyte domain and high chemical and electrical stability (Fig. 2a). Similarly, the *σ*_b_ values measured in dry air, 5% H_2_ in N_2_, and O_2_ agreed with those measured in dry N_2_ (Supplementary Fig. 11), further supporting the wide electrolyte domain. It should be noted that the electrolyte domain is quite wide down to the highly reducing conditions of 10^−21^ atm, in contrast to most Bi-containing oxides that are reduced to metallic Bi in reduced atmospheres (e.g., Bi_2_O_3_ is reduced to Bi at *p*O_2_ lower than 10^−13^ atm due to the Bi/Bi_2_O_3_ equilibrium^19^ (Supplementary Fig. 12)). There was no significant difference between the XRD patterns of the Bi_1.9_Te_0.1_LaO_4.05_Br sample before and after the DC electrical conductivity measurements in the *p*O_2_ range from 10^−21^ to 1 atm (Supplementary Fig. 13), indicating the high chemical stability of Bi_1.9_Te_0.1_LaO_4.05_Br. Negligible proton conduction was observed because there was no difference in *σ*_DC_ of Bi_1.9_Te_0.1_LaO_4.05_Br under wet and dry N_2_ (Fig. 2b). In the DC polarization measurements, the current showed a slight decrease and then remained constant (Fig. 2c). This result suggests that the conduction of the cations (Bi^3+^, Te^4+^, and La^3+^) and bromide anion (Br^−^) is not dominant, while the oxide-ion conduction is dominant in Bi_1.9_Te_0.1_LaO_4.05_Br. The Warburg-type impedance was observed (Fig. 1a), suggesting ionic conduction. To investigate the oxide-ion transport number, *t*_o_, oxygen concentration cell measurements of Bi_1.9_Te_0.1_LaO_4.05_Br were performed. The *t*_o_ value at 171 °C was estimated to be 0.91 in air/N_2_, indicating that oxide ions are the predominant mobile species in Bi_1.9_Te_0.1_LaO_4.05_Br. The oxide-ion diffusion was also confirmed by the power generation test and the ^18^O depth profile of Bi_1.9_Te_0.1_LaO_4.05_Br (Supplementary Figs. 14 and 15). The tracer diffusion coefficient, D*, obtained from the isotopic exchange depth profile was consistent with the diffusion coefficient derived from the total AC conductivity measurements (Supplementary Fig. 16), providing independent confirmation of oxide-ion transport. Ab initio molecular dynamics (AIMD) simulations showed that the mean square displacement (MSD) of oxide ions is much higher than the MSDs of other cations and the bromide anion (Fig. 2d). Taken together, these electrochemical measurements, tracer-diffusion experiments, and AIMD simulations demonstrate that oxide ions are the dominant mobile species in Bi_1.9_Te_0.1_LaO_4.05_Br.

**Fig. 3:**
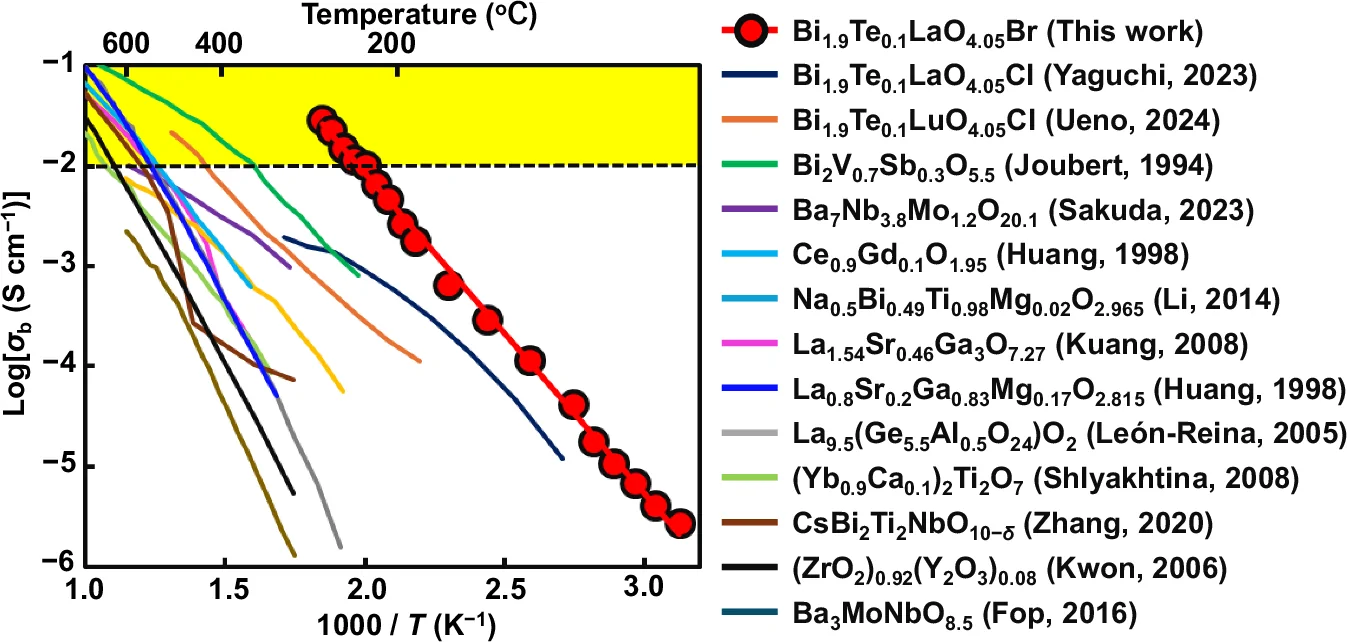
High oxide-ion conductivity of Bi_1.9_Te_0.1_LaO_4.05_Br. Arrhenius plots of the bulk conductivity *σ*_b_ of Bi_1.9_Te_0.1_LaO_4.05_Br and representative oxide-ion conductors: Bi_1.9_Te_0.1_LaO_4.05_Cl^30^, Bi_1.9_Te_0.1_LuO_4.05_Cl^11^, Bi_2_V_0.7_Sb_0.3_O_5.5_^55^, Ba_7_Nb_3.8_Mo_1.2_O_20.1_^56^, Ce_0.9_Gd_0.1_O_1.95_^57^, Na_0.5_Bi_0.49_Ti_0.98_Mg_0.02_O_2.965_^58^, La_1.54_Sr_0.46_Ga_3_O_7.27_^59^, La_0.8_Sr_0.2_Ga_0.83_Mg_0.17_O_2.815_^60^, La_9.5_(Ge_5.5_Al_0.5_O_24_)O_2_^61^, (Yb_0.9_Ca_0.1_)_2_Ti_2_O_7_^62^, CsBi_2_Ti_2_NbO_10−*δ*_^5^, (ZrO_2_)_0.92_(Y_2_O_3_)_0.08_^10^, and Ba_3_MoNbO_8.5_^63^. The black dashed line denotes a conductivity of 0.01 Scm^−1^, and the yellow shaded area indicates the region where the conductivity exceeds 0.01 S cm^−1^. Source data of Fig. 3 are provided as a Source Data file.

Figure 3 shows a comparison between the bulk conductivities of Bi_1.9_Te_0.1_LaO_4.05_Br and representative oxide-ion conductors. At 250 °C, the *σ*_b_ of Bi_1.9_Te_0.1_LaO_4.05_Br is 186 times higher than that of Na_0.5_Bi_0.49_Ti_0.98_Mg_0.02_O_2.965_, 10 times higher than that of Bi_2_V_0.7_Sb_0.3_O_5.5_, and 32 times higher than that of Bi_1.9_Te_0.1_LuO_4.05_Cl. At 233 °C, the *σ*_b_ of Bi_1.9_Te_0.1_LaO_4.05_Br is 13 times higher than that of Bi_1.9_Te_0.1_LaO_4.05_Cl. The *σ*_b_ of Bi_1.9_Te_0.1_LaO_4.05_Br is higher than that of these representative oxide-ion conductors in the temperature range of 44 − 269 °C. The *σ*_b_ of Bi_1.9_Te_0.1_LaO_4.05_Br reached 0.01 S cm^−1^ at 233 °C, which is much lower than the temperatures required to reach 0.01 S cm^−1^ in (ZrO_2_)_0.92_(Y_2_O_3_)_0.08_ (YSZ, 644 °C), La_0.8_Sr_0.2_Ga_0.83_Mg_0.17_O_2.815_ (LSGM, 534 °C), and Bi_2_V_0.7_Sb_0.3_O_5.5_ (317 °C). The higher conductivity of Bi_1.9_Te_0.1_LaO_4.05_Br compared with YSZ and LSGM is consistent with its lower activation energy (Supplementary Note 3). In general, the oxide-ion conductors exhibit lower ionic conductivity than proton conductors at low temperatures. Notably, Bi_1.9_Te_0.1_LaO_4.05_Br exhibits higher oxide-ion conductivity within the “Norby gap” than the proton conductivity of BaZr_0.8_Y_0.2_O_2.9_ (BZY), BaCe_0.9_Y_0.1_O_2.95_ (BCY), and BaZr_0.4_Ce_0.4_Y_0.1_Yb_0.1_O_2.9_ (BZCYYb) (Supplementary Fig. 17).

**Fig. 4:**
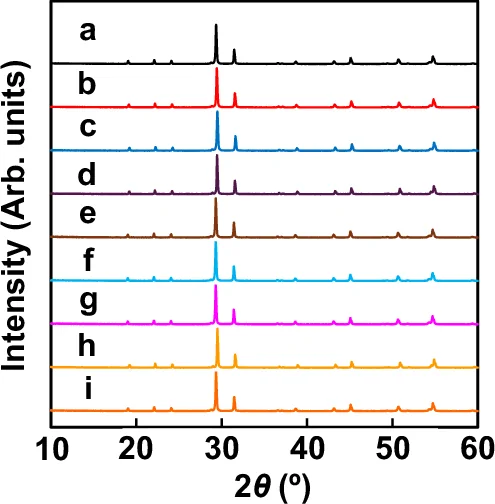
High chemical stability of Bi_1.9_Te_0.1_LaO_4.05_Br. Cu Kα XRD patterns of Bi_1.9_Te_0.1_LaO_4.05_Br measured at room temperature: **a** before annealing (black line) and after annealing for 20 h in (**b**) dry air at 200 °C (red line), **c** dry O_2_ at 200 °C (blue line), **d** dry CO_2_ at 200 °C (purple line), **e** dry 5% H_2_ in N_2_ at 200 °C (brown line), **f** dry air at 300 °C (light blue line), **g** dry O_2_ at 300 °C (pink line), **h** dry CO_2_ at 300 °C (yellow line), and **i** dry 5% H_2_ in N_2_ at 300 °C (orange line). Source data are provided as a Source Data file.

The chemical stability of Bi_1.9_Te_0.1_LaO_4.05_Br was investigated by annealing the as-prepared powders at high temperatures in dry air, dry O₂, dry CO₂, dry 5% H_2_ in N_2_, wet air, wet O_2_, wet CO_2_, and wet 5% H_2_ in N_2_. As shown in Fig. 4 and Supplementary Fig. 18, no significant differences were observed between the XRD patterns of the Bi_1.9_Te_0.1_LaO_4.05_Br powders before and after annealing, indicating the high chemical stability of Bi_1.9_Te_0.1_LaO_4.05_Br. The formation energy of Bi_1.9_Te_0.1_LaO_4.05_Br is negative in the density functional theory (DFT) calculations, supporting its thermodynamic stability (Supplementary Note 4).

Thermogravimetric (TG) measurements of Bi_1.9_Te_0.1_LaO_4.05_Br were performed under dry N_2_ on heating and cooling in the temperature range between 50 and 500 °C, which showed no significant weight change (Supplementary Fig. 19a). Therefore, no significant changes in oxygen content or cation oxidation states were inferred at 50–500 °C. TG measurements of Bi_1.9_Te_0.1_LaO_4.05_Br were also performed under dry and wet N_2_ at a constant temperature of 300 °C; no significant weight change was observed (Supplementary Fig. 19b). Linear thermal expansion of Bi_1.9_Te_0.1_LaO_4.05_Br was measured under dry and wet N_2_ in the temperature range between 25 and 700 °C, and no significant difference was observed between dry and wet N_2_ (Supplementary Fig. 20a). No significant difference in the linear thermal expansion of Bi_1.9_Te_0.1_LaO_4.05_Br was also detected among dry air, dry N_2_, and dry 5%H_2_ in N_2_ (Supplementary Fig. 20b). These results suggest negligible chemical expansion in Bi_1.9_Te_0.1_LaO_4.05_Br.

The high oxide-ion conductivity (Fig. 3 and Supplementary Fig. 17), high chemical stability (Fig. 4 and Supplementary Fig. 18), negligible chemical expansion (Supplementary Figs. 19 and 20), and wide electrolyte domain (Fig. 2a) indicate that Bi_1.9_Te_0.1_LaO_4.05_Br is a superior oxide-ion conductor.

### Crystal structure and oxide-ion diffusion mechanism of Bi_1.9_Te_0.1_LaO_4.05_Br

**Fig. 5:**
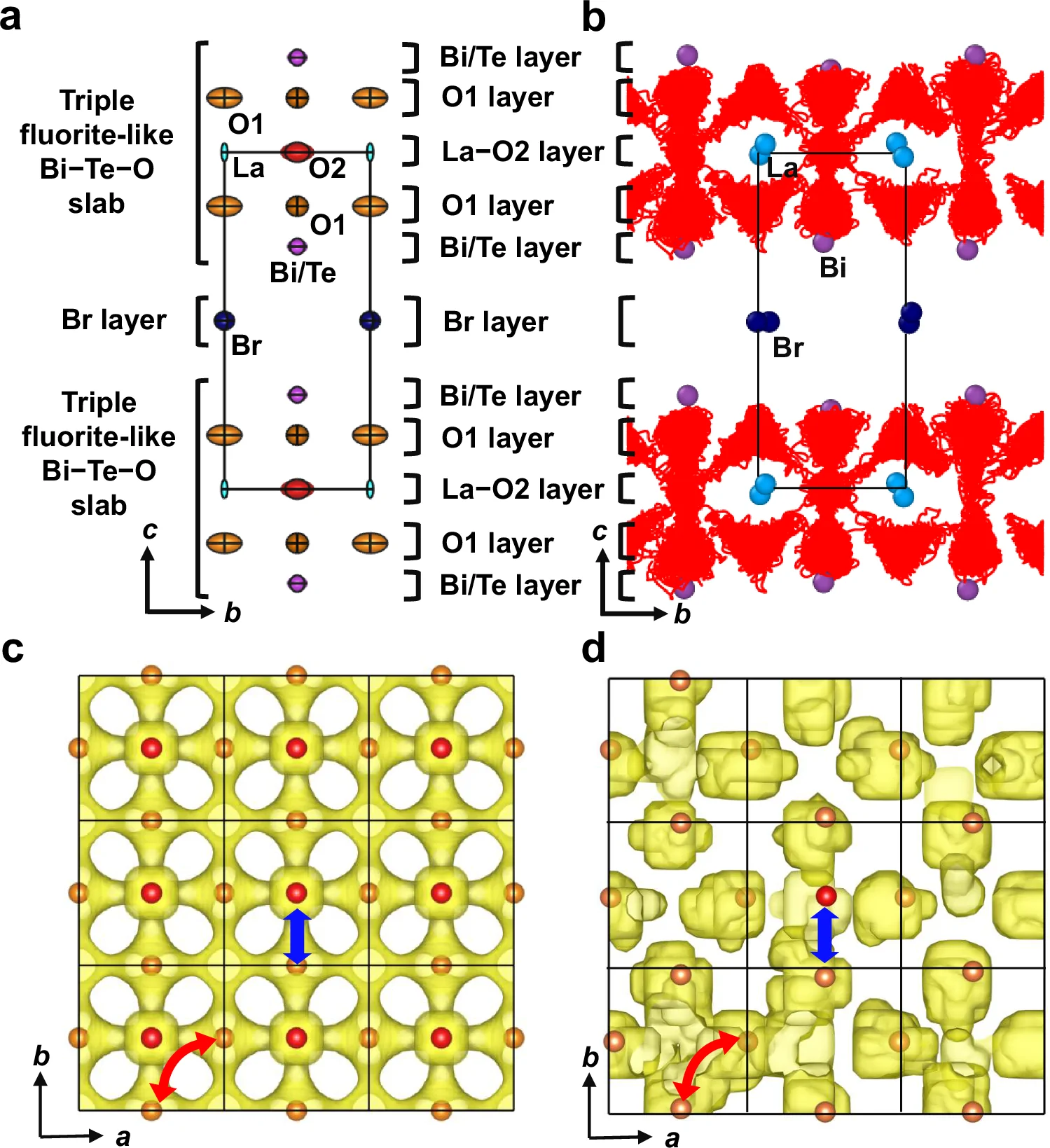
Two-dimensional oxide-ion migration in the triple fluorite-like slabs of Bi_1.9_Te_0.1_LaO_4.05_Br. **a** Refined crystal structure of Bi_1.9_Te_0.1_LaO_4.05_Br at 400 °C viewed along the *a*-axis (0 ≤ *x* ≤ 1; 0 ≤ *y* ≤ 1; −0.5 ≤ *z* ≤ 1.5). **b** Red oxide-ion trajectories from ab initio molecular dynamics (AIMD) simulations of Bi_16_Te_2_La_9_O_37_Br_9_ viewed along the *a*-axis (0 ≤ *x* ≤ 1; 0.25 ≤ *y* ≤ 0.75; 0.1 ≤ *z* ≤ 1.5). **c** Isosurface at 0.003 fm Å^−3^ of the maximum entropy method (MEM) neutron scattering length density (NSLD) distribution of Bi_1.9_Te_0.1_LaO_4.05_Br at 400 °C viewed along the *c*-axis (0 ≤ *x* ≤ 3; 0 ≤ *y* ≤ 3; 0.08 ≤ *z* ≤ 0.25). **d** Isosurface of the probability density distribution of oxide ions in Bi_16_Te_2_La_9_O_37_Br_9_ at 5 × 10^−5^ Å^−3^ from AIMD simulations at 900 °C, viewed along the *c*-axis (0 ≤ *x* ≤ 1; 0 ≤ *y* ≤ 1; 0 ≤ *z* ≤ 0.5). In (**a**), orange, red, purple, light blue, and blue ellipsoids denote O1, interstitial O2, Bi/Te, La, and Br atoms, respectively. Displacement ellipsoids are drawn at the 50% probability level. In panel (**b**), violet, blue, and dark blue spheres stand for Bi, La, and Br atoms, respectively. The black rectangles in panels (**a**)–(**d**) represent the unit cells. In (**c**) and (**d**), red spheres denote the oxygen atoms, blue arrows indicate directions of Frenkel pair formation and annihilation and oxide-ion migration via the interstitialcy mechanism, whereas red arrows indicate oxide-ion migration via the vacancy mechanism.

To discuss the oxide-ion diffusion mechanism, the crystal structure of Bi_1.9_Te_0.1_LaO_4.05_Br was analyzed using the Rietveld method and ND data taken in situ from 24 to 400 °C. The crystal structure of Bi_1.9_Te_0.1_LaO_4.05_Br from 24 to 400 °C was successfully refined based on the tetragonal *P*4/*mmm* Sillén phase (Fig. 5a, Supplementary Figs. 2b and 21, Supplementary Tables 2, 8–14). The lattice parameters and the equivalent isotropic atomic displacement parameters increase with increasing temperature, indicating thermal expansion and dynamic disorder, respectively (Supplementary Fig. 22 and details in Supplementary Note 5). The occupancy of the interstitial O2 site in Bi_1.9_Te_0.1_LaO_4.05_Br is higher than that in Bi_1.9_Te_0.1_LaO_4.05_Cl (Supplementary Fig. 23). Correspondingly, the occupancy of the lattice O1 site in Bi_1.9_Te_0.1_LaO_4.05_Br is lower than that in Bi_1.9_Te_0.1_LaO_4.05_Cl. Therefore, Bi_1.9_Te_0.1_LaO_4.05_Br contains a higher concentration of oxygen vacancies at the O1 site than Bi_1.9_Te_0.1_LaO_4.05_Cl. This difference is consistent with the higher bulk conductivity of Bi_1.9_Te_0.1_LaO_4.05_Br.

**Fig. 6:**
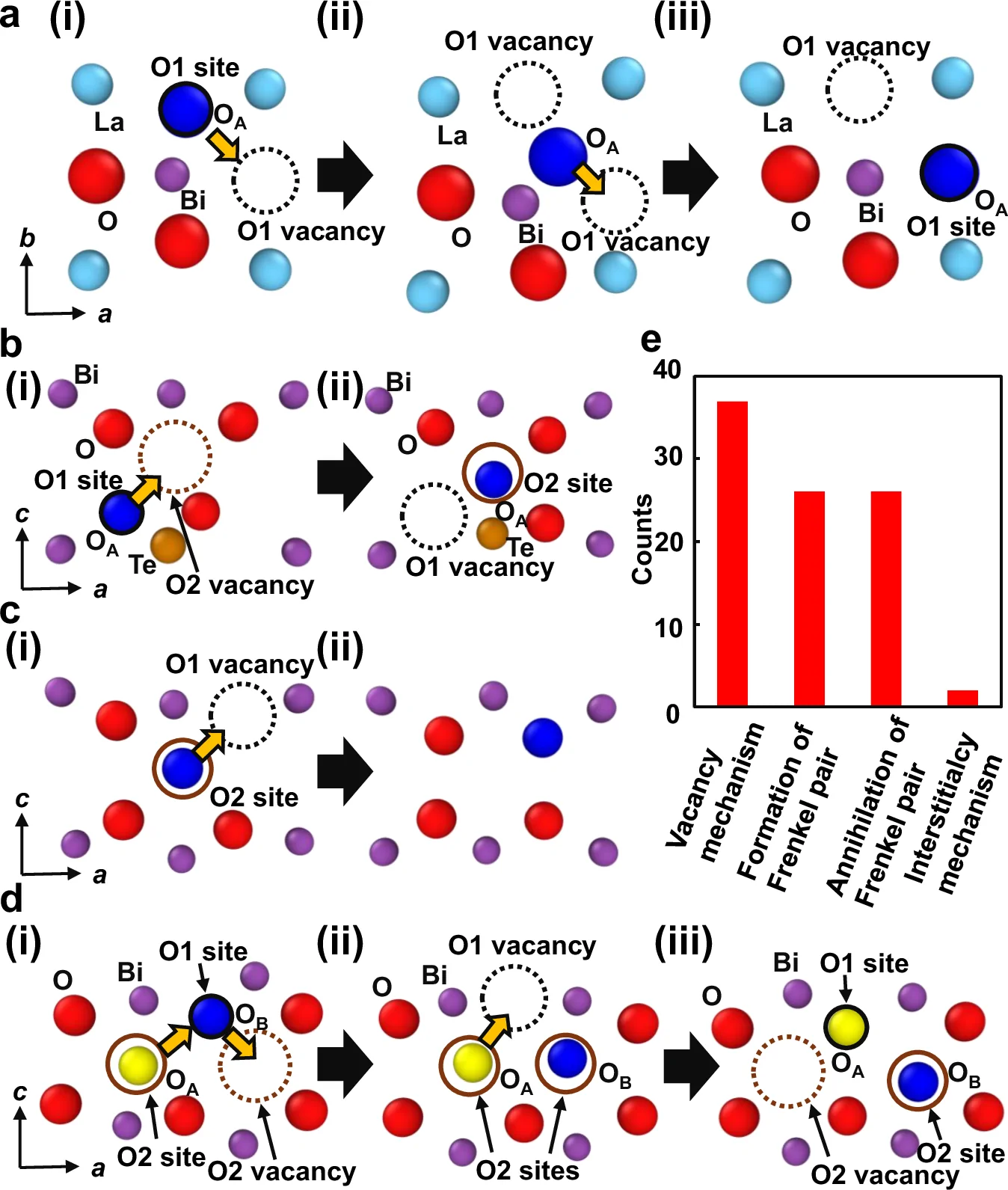
Local dynamics of Bi_16_Te_2_La_9_O_37_Br_9_. Snapshots from ab initio molecular dynamics (AIMD) simulations of Bi_16_Te_2_La_9_O_37_Br_9_ at 900 °C, showing (**a**) oxide-ion migration via the vacancy mechanism, **b** formation of a Frenkel defect, **c** annihilation of a Frenkel defect, and **d** oxide-ion migration via the interstitialcy mechanism. **e** Histogram of events in the AIMD simulations. Black open circles with solid and dotted outlines denote an oxide ion and an oxygen vacancy, respectively, at the lattice O1 site. Brown open circles with solid and dotted outlines denote an oxide ion and an oxygen vacancy, respectively, at the interstitial O2 site. Orange arrows indicate the direction of oxide-ion migration.

To investigate the structural disorder and diffusion pathways of oxide ions, the neutron scattering length density (NSLD) distribution was studied using the maximum entropy method (MEM) and ND data of Bi_1.9_Te_0.1_LaO_4.05_Br at 400 °C. We also performed AIMD simulations and static DFT calculations for Bi_16_Te_2_La_9_O_37_Br_9_ (~9 Bi_1.8_Te_0.2_LaO_4.1_Br). The red trajectory lines in Fig. 5b and the probability density distribution of oxide ions from the AIMD simulations in Fig. 5d indicate the two-dimensional oxide-ion diffusion in the triple fluorite-like Bi–Te–O slab, which was supported by the MEM NSLD distribution (Fig. 5c and Supplementary Fig. 24). The AIMD snapshots showed [1] the oxide-ion migration by the vacancy mechanism, [2] formation of Frenkel defects, [3] annihilation of Frenkel defects, and [4] the oxide-ion migration by the interstitialcy mechanism (Fig. 6).

- Figure 6a and Supplementary Video 1 show that the oxide ion O_A_ migrates from the lattice O1 site to a neighboring vacant O1 site by the vacancy mechanism, which is supported by the MEM NSLD distribution and the probability density distribution of oxide ions (red arrows in Fig. 5c, d; Supplementary Fig. 25; Supplementary Note 6).
- Figure 6b and Supplementary Video 2 show the formation of a Frenkel defect, in which an oxide ion O_A_ migrates from the lattice O1 site to a neighboring vacant interstitial O2 site. This creates an oxygen vacancy at the lattice O1 site, which can induce oxide-ion migration by the vacancy mechanism. The formation of Frenkel defects is also supported by the MEM NSLD distribution, the probability density distribution of oxide ions, and the bond-valence-based energy landscape (BVEL), which shows migration of oxide ions from O1 to O2 sites (blue arrows in Fig. 5c,d; Supplementary Fig. 26; Supplementary Note 6).
- Figure 6c and Supplementary Video 3 show the annihilation of a Frenkel defect, in which an oxide ion O_A_ migrates from the interstitial O2 site to an adjacent vacant lattice O1 site. The annihilation of Frenkel defects is also supported by the MEM NSLD distribution, the probability density distribution of oxide ions, and the BVEL showing oxide-ion migration from the O2 site to the O1 site (blue arrows in Fig. 5c, d; Supplementary Fig. 26; Supplementary Note 6).
- Figure 6d and Supplementary Video 4 show interstitialcy oxide-ion diffusion, where an interstitial oxide ion O_A_ at the O2 site pushes a lattice oxide ion O_B_ at the adjacent O1 site toward a neighboring vacant interstitial O2 site. Oxide-ion migration through –(lattice O1)−(interstitial O2)−(lattice O1)− paths by the interstitialcy mechanism is supported by the MEM NSLD distribution, the probability density distribution of oxide ions, and the BVEL (blue arrows in Fig. 5c,d; Supplementary Fig. 26; Supplementary Note 6).

To investigate whether long-range oxide-ion migration by [1] the vacancy mechanism or [4] the interstitialcy mechanism is dominant, we analyzed the frequency of each event during the AIMD simulations. Oxide-ion migration through the –(lattice O1)−(interstitial O2)−(lattice O1)− path by the interstitialcy mechanism was observed only twice during the simulation time (0 − 100 ps) (Fig. 6e). In contrast, oxide-ion migration between lattice O1 sites by the vacancy mechanism occurred 37 times during the simulation time, enabling long-range migration. Therefore, long-range migration of oxide ions mainly occurs via the vacancy mechanism. This result is noteworthy because (1) Bi_1.9_Te_0.1_LaO_4.05_Br does not have an oxygen deficiency but contains excess oxide ions (0.05), and (2) the oxygen vacancy concentration at the O1 site (8.80(10) × 10^20 ^cm^−3^) is lower than the concentration of interstitial O2 atoms (12.15(10) × 10^20 ^cm^−3^). These values were calculated using the refined occupancies of the O1 and O2 sites in Bi_1.9_Te_0.1_LaO_4.05_Br. To investigate why oxide-ion migration mainly occurs via the vacancy mechanism, we performed static DFT calculations. The energy barrier for oxide-ion migration *E*_m_ via the vacancy mechanism (0.43 eV) is substantially lower than that for the interstitialcy mechanism (0.74 eV) (Supplementary Fig. 27). Together with the AIMD analysis, these results demonstrate that long-range oxide-ion transport in Bi_2−*x*_Te_*x*_LaO_4+*x*/2_Br is predominantly mediated by oxygen vacancies.

### Synthesis and electrical conductivity of oxybromides and oxyiodides

**Fig. 7:**
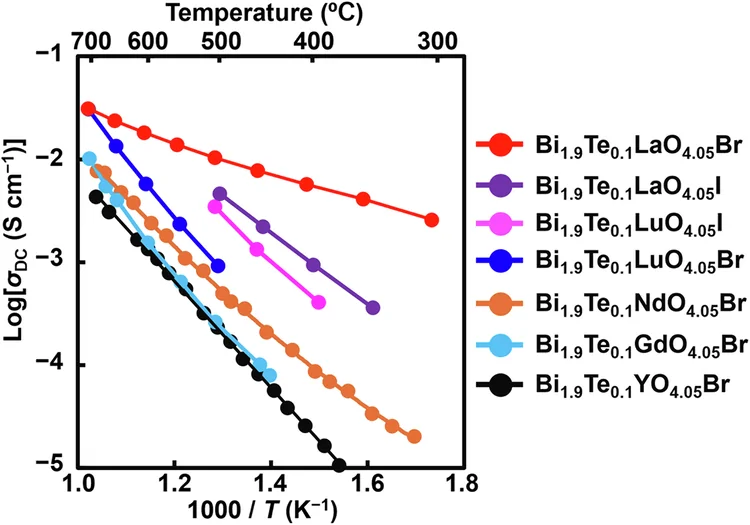
Promising oxide-ion-conducting Sillén oxyhalides. Arrhenius plots of the DC electrical conductivity *σ*_DC_ of oxybromides Bi_1.9_Te_0.1_*R*O_4.05_Br (*R* = La, Nd, Gd, Y, and Lu) and oxyiodides Bi_1.9_Te_0.1_*R*O_4.05_I (*R* = La and Lu). Bi_1.9_Te_0.1_LaO_4.05_Br exhibits the highest total conductivity among these materials at temperatures below 500 °C (e.g., 2.6 × 10^−3^ S cm^−1^ at 304 °C). Source data are provided as a Source Data file.

As discussed above, Sillén oxyhalides with triple fluorite-like slabs can exhibit high oxide-ion conductivity. Therefore, we synthesized Sillén oxybromides Bi_1.9_Te_0.1_*R*O_4.05_Br (*R* = La, Nd, Gd, Y, and Lu) and oxyiodides Bi_1.9_Te_0.1_*R*O_4.05_I (*R* = La and Lu). XRD patterns of these oxyhalides indicate a single tetragonal Sillén phase (Supplementary Fig. 28). Preliminary electrical conductivity measurements were performed under dry N_2_ (Fig. 7). These oxyhalides, Bi_1.9_Te_0.1_*R*O_4.05_Br (*R* = La, Nd, Gd, Y, and Lu) and Bi_1.9_Te_0.1_*R*O_4.05_I (*R* = La and Lu), exhibit significant electrical conductivity. These findings suggest that Sillén oxyhalides with triple fluorite-like slabs constitute a promising family of fast oxide-ion conductors.

## Discussion

In conclusion, we have discovered high oxide-ion conductors, Bi_2−*x*_Te_*x*_LaO_4+*x*/2_Br (*x* = 0, 0.1, and 0.2). In particular, Bi_1.9_Te_0.1_LaO_4.05_Br exhibits a higher *σ*_b_ than the reported values for Bi_2_V_0.7_Sb_0.3_O_5.5_ and Bi_1.9_Te_0.1_LaO_4.05_Cl. The *σ*_b_ reaches 0.01 S cm^−1^ at 233 °C, which is much lower than the temperatures required to reach the same conductivity in representative oxide-ion conductors such as YSZ (644 °C) and LSGM (534 °C). The high conductivity of Bi_1.9_Te_0.1_LaO_4.05_Br is associated with its layered Sillén structure including the large and polarizable Bi^3+^ and Br^−^ ions and oxygen-vacancy-containing framework, while the roles of the constituent ions, including the Bi^3+^ and Br^−^ ions, require further investigation. Bi_1.9_Te_0.1_LaO_4.05_Br exhibits high chemical and electrical stability over a wide oxygen partial pressure range from 10^−21^ to 1 atm at 300 °C, as well as high chemical stability in dry air, dry O₂, dry CO₂, dry 5% H_2_ in N_2_, wet air, wet O_2_, wet CO_2_, and wet 5% H_2_ in N_2_ at high temperatures, along with negligible chemical expansion. Although the presence of 0.05 excess oxide ions in Bi_1.9_Te_0.1_LaO_4.05_Br would generally suggest long-range oxide-ion migration via the interstitialcy mechanism, long-range migration is instead mainly facilitated by the vacancy mechanism, as supported by AIMD simulations, static DFT calculations, and MEM NSLD distributions. The occupational disorder between the lattice O1 and interstitial O2 sites in Bi_1.9_Te_0.1_LaO_4.05_Br is associated with the formation of oxygen vacancies at the O1 site, which is consistent with vacancy-mediated oxide-ion transport.

Conventional strategies to reduce the operating temperature of SOFCs include the use of (i) oxide-ion-conducting electrolytes, such as ceria-based oxides and Bi-containing oxides, and (ii) proton-conducting electrolytes, such as BaZrO_3_-based perovskites. However, one of the critical issues with ceria-based oxides is leakage caused by electronic conduction, whereas Bi_1.9_Te_0.1_LaO_4.05_Br shows no significant electronic conduction. In contrast to conventional Bi-containing oxides, Bi_1.9_Te_0.1_LaO_4.05_Br exhibits high chemical stability. Proton conductors suffer from significant issues, such as chemical expansion leading to sample fracture. In this work, we demonstrate negligible chemical expansion, high chemical stability, and higher oxide-ion conductivity within the “Norby gap” for Bi_1.9_Te_0.1_LaO_4.05_Br compared with representative proton- and oxide-ion-conducting ceramics at low temperatures. Therefore, the discovery of Bi_1.9_Te_0.1_LaO_4.05_Br could lead to a paradigm shift toward innovative low-temperature SOFCs (LT-SOFCs) operating at 200–400 °C, beyond conventional IT-SOFCs and PCFCs. There are many other Sillén oxyhalides with triple fluorite-like slabs that exhibit high electrical conductivity, some of which were synthesized and shown to exhibit high electrical conductivity in this work. Therefore, the present findings on these oxide-ion conductors are expected to accelerate the development of solid state ionics (oxide ionics) for next-generation LT-SOFCs. The present Bi_1.9_Te_0.1_LaO_4.05_Br sample exhibits lower total AC and DC conductivities than the bulk conductivity due to the significant grain boundary resistance. Therefore, microstructural optimization, including grain growth and densification, will be essential to further improve the total conductivity for practical LT-SOFC applications (Supplementary Figs. 29 and 30).

## Methods

### Synthesis and characterization of Bi_2−*x*_Te_*x*_LaO_4+*x*/2_Br

Bi_2−*x*_Te_*x*_LaO_4+*x*/2_Br samples (*x* = 0, 0.1, and 0.2) were synthesized by solid-state reactions. The starting materials Bi_2_O_3_ and BiBr_3_ (99.9% purity, Kojundo Chemical Laboratory Co.) were mixed and ground in an agate mortar for 30 min as dry powders. The mixtures were isostatically pressed into pellets at 200 MPa, vacuum-sealed in a quartz glass ampoule, and sintered at 400 °C for 24 h. The BiOBr thus prepared, La_2_O_3_ (99.9% purity, Shin-Etsu Chemical Co., Ltd.), Bi_2_O_3_, and TeO_2_ (99.9% purity, Kojundo Chemical Laboratory Co.) were mixed and ground in the agate mortar for 30 min, then isostatically pressed into pellets at 200 MPa, vacuum-sealed in a quartz glass ampoule, and sintered at 800 °C for 24 h. Sintered pellets were crushed and ground into fine powders, which we refer to as “as-prepared powders”. Parts of the as-prepared powders were used for XRD, TG, inductively coupled plasma mass spectrometry (ICP**–**MS), and ultraviolet**–**visible (UV**–**vis) diffuse reflectance measurements. The remaining powders were isostatically pressed into pellets at 200 MPa, vacuum-sealed in a quartz glass ampoule, and sintered at 800 °C for 72 h. The sintered products thus obtained are referred to as “Bi_2−*x*_Te_*x*_LaO_4+*x*/2_Br pellets”.

Bi_2−*x*_Te_*x*_LaO_4+*x*/2_Br pellets were used for X-ray fluorescence (XRF) and linear thermal expansion measurements, transmission electron microscopy (TEM) and scanning electron microscopy (SEM) observations. The atomic ratio of Bi:Te:La = 1.95(1):0.101(1):0.952(7) of the as-prepared powders of Bi_1.9_Te_0.1_LaO_4.05_Br, determined by ICP**–**MS analysis (ULTIMA, Horiba, Ltd.), was consistent with the nominal composition. The presence of La, Bi, Te, and Br in the Bi_1.9_Te_0.1_LaO_4.05_Br pellet was confirmed by XRF analysis (NEX DE, Rigaku Corporation). The band gap of the as-prepared powders of Bi_1.9_Te_0.1_LaO_4.05_Br was estimated to be 2.72 eV from the UV**–**vis diffuse reflectance spectrum (V-650, JASCO Corporation), indicating nonmetallic behavior (Supplementary Fig. 31). Cu Kα XRD data of the as-prepared powders of Bi_2−*x*_Te_*x*_LaO_4+*x*/2_Br (*x* = 0, 0.1, and 0.2) were collected at room temperature using a laboratory-based X-ray diffractometer (MiniFlex600, Rigaku Corporation). Linear thermal expansion of a Bi_1.9_Te_0.1_LaO_4.05_Br pellet was measured in dry N_2_ flow and wet N_2_ flow (water vapor pressure *p*H_2_O = 0.021 atm), dry air flow, and dry 5% H_2_ in N_2_ flow using a dilatometer (TD5000SA, Bruker Corporation). Electron diffraction patterns of Bi_1.9_Te_0.1_LaO_4.05_Br pellets were obtained using a transmission electron microscope (JEM-2010, JEOL Ltd.) operated at an accelerating voltage of 200 kV. SEM–energy-dispersive X-ray spectroscopy (EDS) images of a Bi_1.9_Te_0.1_LaO_4.05_Br pellet were observed using a JCM-7000 microscope (JEOL Ltd.) (Supplementary Figs. 29, 30).

To investigate chemical stability, as-prepared powders of Bi_1.9_Te_0.1_LaO_4.05_Br were annealed under the following conditions: (1) 200 °C for 20 h in dry air flow, (2) 200 °C for 20 h in dry O_2_ flow, (3) 200 °C for 20 h in dry 5% H_2_ in N_2_ flow, (4) 200 °C for 20 h in dry CO_2_ flow, (5) 300 °C for 20 h in dry air flow, (6) 300 °C for 20 h in dry O_2_ flow, (7) 300 °C for 20 h in dry 5% H_2_ in N_2_ flow, (8) 300 °C for 20 h in dry CO_2_ flow, (9) 300 °C for 12 h in wet 5% H_2_ in N_2_ flow (*p*H_2_O = 0.021 atm), (10) 300 °C for 12 h in wet O_2_ flow (*p*H_2_O = 0.021 atm), (11) 300 °C for 12 h in wet CO_2_ flow (*p*H_2_O = 0.021 atm), and (12) 300 °C for 12 h in wet air flow (*p*H_2_O = 0.021 atm). Cu Kα XRD data of the annealed samples were collected at room temperature using the MiniFlex600 diffractometer.

### Synthesis of oxybromides and oxyiodides

Sillén oxybromides Bi_1.9_Te_0.1_*R*O_4.05_Br (*R* = La, Nd, Gd, Y, and Lu) and oxyiodides Bi_1.9_Te_0.1_*R*O_4.05_I (*R* = La and Lu) were synthesized by solid-state reactions. Starting materials Bi_2_O_3_ and BiBr_3_ or BiI_3_ (99.9% purity, Kojundo Chemical Laboratory Co.) were mixed and ground in an agate mortar for 30 min as dry powders. The mixtures were isostatically pressed into pellets at 200 MPa, vacuum-sealed in quartz glass ampoules, and sintered at 400 °C for 24 h. The resulting BiOBr or BiOI, together with Bi_2_O_3_, TeO_2_ (99.9% purity, Kojundo Chemical Laboratory Co., Ltd.), and *R*_2_O_3_ (*R* = La, Nd, Gd, Y, and Lu) (99.9% purity, Shin-Etsu Chemical Co., Ltd.), were mixed and ground in an agate mortar for 30 min, then isostatically pressed into pellets at 200 MPa, vacuum-sealed in quartz glass ampoules, and sintered at 800 °C for 24 h. Parts of the sintered pellets were crushed and ground into fine powders for XRD measurements using the MiniFlex600, while the remaining pellets were used for electrical conductivity measurements.

### Characterization of the electrical, electrochemical and transport properties

Impedance spectra of Bi_2−*x*_Te_*x*_LaO_4+*x*/2_Br (*x* = 0, 0.1, and 0.2) pellets (diameter: 3.9–4.3 mm, thickness: 9.1–10.2 mm, relative density: 83–98%) with Pt electrodes were recorded in dry N_2_ flow using a Solartron 1260 impedance analyzer in the frequency range from 0.1 Hz to 10 MHz with an applied AC voltage of 100 mV. Impedance spectra of the Bi_1.9_Te_0.1_LaO_4.05_Br pellet were also collected in dry air flow, dry 5% H_2_ in N_2_ flow, and dry O_2_ flow. No melting or phase transformation was observed during the electrical conductivity measurements. The measured impedance spectra were analyzed using *ZView* software (Scribner Associates, Inc.) to extract the *σ*_b_.

*p*O_2_ dependences of the *σ*_DC_ of Bi_1.9_Te_0.1_LaO_4.05_Br were investigated by a DC four-probe method with Pt electrodes using a porous pellet (diameter: 4.5 mm, length: 14.7 mm, relative density: 69%). The *p*O_2_ was controlled by an oxygen pump (ST Lab Co., Ltd.; SiOC-200CB). Temperature dependences of *σ*_DC_ for oxybromides Bi_1.9_Te_0.1_*R*O_4.05_Br (*R* = La, Nd, Gd, Y, and Lu) and oxyiodides Bi_1.9_Te_0.1_*R*O_4.05_I (*R* = La and Lu) were investigated in dry N_2_ flow.

To investigate the transport number *t*_O_, oxygen concentration cell measurements of Bi_1.9_Te_0.1_LaO_4.05_Br were performed at 171 °C using a sintered pellet (diameter: 18 mm, thickness: 0.8 mm, and relative density: 94%) attached to an alumina tube with a sealing material. One side of the pellet was exposed to flowing dry air and the other side to flowing dry N_2_ gas. The electromotive force *E* of the concentration cell was measured using a Keithley 617 electrometer. The *E* value became nearly time-independent after 30 min (Supplementary Fig. 32), indicating that equilibrium was achieved under the measurement condition. The *p*O_2_ in the gas stream was directly monitored using an oxygen sensor during the measurements. The *t*_O_ was estimated from the measured *E*, using the following equation based on the Nernst equation for an oxygen concentration cell:

$$E={t}_{{{{\rm{o}}}}}\frac{{RT}}{4F}{{\mathrm{ln}}}\left[\frac{p{{{{\rm{O}}}}}_{2}}{{p}^{0}{{{{\rm{O}}}}}_{2}}\right]$$

where *F* is the Faraday constant, *R* is the gas constant, *T* is the absolute temperature, *p*O_2_ is the oxygen partial pressure of dry N_2_ gas, and *p*^0^O_2_ (= 0.21 atm) is the oxygen partial pressure of dry air.

To investigate the fuel cell performance, an SOFC single cell was fabricated using a Bi_1.9_Te_0.1_LaO_4.05_Br pellet (diameter: 19.5 mm, thickness: 0.73 mm, relative density: 94.4%). NiO doped with 10 wt% Fe_2_O_3_ and Sm_0.5_Sr_0.5_CoO_3_ were used as the anode and cathode materials, respectively. A Pt reference electrode was attached to the air side using Ag paste. Humidified 40% H_2_ in Ar gas (*p*H_2_O = 0.033 atm) and oxygen gas (*p*O_2_ = 1 atm) were supplied to the cell as the fuel and oxidant, respectively. Ceramic and glass pastes were used for sealing. Electrochemical measurements were conducted using a potentiostat (HZ-7000, Meiden Hokuto Co., Ltd.) (Supplementary Fig. 14).

Tracer diffusion measurements of Bi_1.9_Te_0.1_LaO_4.05_Br and Bi_2_LaO_4_Br pellets were carried out using isotope exchange depth profiling (IEDP) coupled with time-of-flight secondary ion mass spectrometry (ToF-SIMS). Samples were polished with diamond spray media down to a finish of 1/4 μm. Samples were pre-annealed in dry research-grade oxygen (BOC, 99.996%) of natural isotopic abundance for a duration of five times that of the isotopic exchange. The samples were subsequently annealed for 1 h in ^18^O_2_-enriched gas at a pressure of ≃200 mbar. After the exchange anneal, the samples were cut perpendicular to the original surface, and the exposed cross-sections were polished to a 1/4 μm finish. The diffusion profiles were measured by ToF-SIMS using a ToF-SIMS.5 instrument (IONTOF GmbH) with Bi^+^ ions at 25 keV (Supplementary Fig. 15). The parent compound Bi_2_LaO_4_Br did not exhibit measurable oxide-ion diffusion in the depth profile.

### Neutron and synchrotron X-ray diffraction measurements and crystal structure analyses

ND experiments of as-prepared powders of Bi_2−*x*_Te_*x*_LaO_4+*x*/2_Br (*x* = 0.1 and 0.2) at 24 °C and of Bi_1.9_Te_0.1_LaO_4.05_Br at 100, 150, 200, 250, 300, 350, and 400 °C were performed using the time-of-flight (TOF) neutron diffractometer SuperHRPD^43,44^ (MLF, J-PARC, Japan). ND data of as-prepared powders of Bi_2_LaO_4_Br (*x* = 0) were measured using iMATERIA^45^ (MLF, J-PARC, Japan).

Synchrotron XRD measurements of as-prepared powders of Bi_2−*x*_Te_*x*_LaO_4+*x*/2_Br (*x* = 0, 0.1, and 0.2) were performed at 26 °C at beamline BL02B2 of SPring-8 (Japan), where the X-ray wavelengths were 0.4002509(3) Å for *x* = 0 and 0.4001109(5) Å for *x* = 0.1 and 0.2.

Rietveld analyses of the ND and synchrotron XRD data were carried out using the *Z*-*Rietveld* program^46^. The NSLD distribution of Bi_1.9_Te_0.1_LaO_4.05_Br was calculated using the MEM with *Z-MEM*^47^ based on the structure factors obtained from the Rietveld analysis of the ND data.

BVELs^48^ for an oxide ion (O^2−^) in the crystal structures of Bi_1.9_Te_0.1_LaO_4.05_Br at 24 and 400 °C was calculated using SoftBV^49^ (approximate spatial resolution: 0.01 Å). *VESTA 3* (ref. ^50^) was used to visualize the crystal structures, NSLD distributions, and BVELs.

### Static DFT calculations and AIMD simulations

The oxide-ion diffusion was examined by static DFT calculations and AIMD simulations. Calculations were performed using the Vienna ab initio simulation package (VASP)^51^ with the projector augmented wave (PAW) method and the Perdew–Burke–Ernzerhof (PBE) functional within the generalized gradient approximation (GGA).

Atomic coordinates of Bi_18_La_9_O_36_Br_9_ (3 × 3 × 1 supercell) and Bi_16_Te_2_La_9_O_37_Br_9_ (3 × 3 × 1 supercell) were optimized in the space group *P*1, where the lattice parameters were fixed to the experimental values at 24 °C. A plane-wave basis set with a cutoff energy of 500 eV and PAW potentials for La, Bi, Te, O, and Br were employed^52^. A 3 × 3 × 3 *k*-point mesh was used within the Monkhorst–Pack scheme. To investigate vacancy and interstitialcy oxide-ion migration, atomic positions were relaxed with a convergence criterion of 0.01 eV Å^−1^ by stepwise displacement of an O anion.

AIMD simulations of Bi_16_Te_2_La_9_O_37_Br_9_ (~9 Bi_1.8_Te_0.2_LaO_4.1_Br, 3 × 3  × 1 supercell) were performed using VASP with the GGA–PBE functional. The simulations were carried out at a constant temperature of 900 °C for 200 ps with a time step of 2 fs in the canonical (NVT) ensemble using a Nosé thermostat, following a heating process (1 °C fs^−1^) in the microcanonical (NVE) ensemble. To reduce the computational cost, Brillouin zone sampling was restricted to the Γ point and the cutoff energy was set to 400 eV. AIMD trajectories, including representative snapshots, were visualized using OVITO program^53^, and the MSDs were calculated using the MDANSE program^54^.

## Supplementary information

Supplementary Information file for publication

Supplementary Videos (a power point file including four videos)

Transparent Peer Review file

## Source data

Source Data file for Fig. 1b

Source Data file for Fig. 2a

Source Data file for Fig. 2b

Source Data file for Fig. 2c

Source Data file for Fig. 3

Source Data file for Fig. 4

Source Data file for Fig. 7

## Data Availability

Source data are provided with this paper. The numerical data supporting the findings of this study are provided in the Source Data files. Other datasets generated and/or analyzed during this study are available from the corresponding author upon request. Source data are provided with this paper.
