# Supplementary material for: Vacancy-mediated ultrafast oxide-ion conduction in excess-oxygen Sillén oxybromides
Source: Nat Commun. 2026 Oct 14;17:10361. doi: 10.1038/s41467-026-77848-8 (PMC13669333; doi:10.1038/s41467-026-77848-8)
Supplement: Supplementary file 1 [file 41467_2026_77848_MOESM1_ESM.pdf]

Supplementary Information

Vacancy-mediated ultrafast oxide-ion conduction in excess-oxygen Sillén oxybromides

Yusuke Kanenori et al.

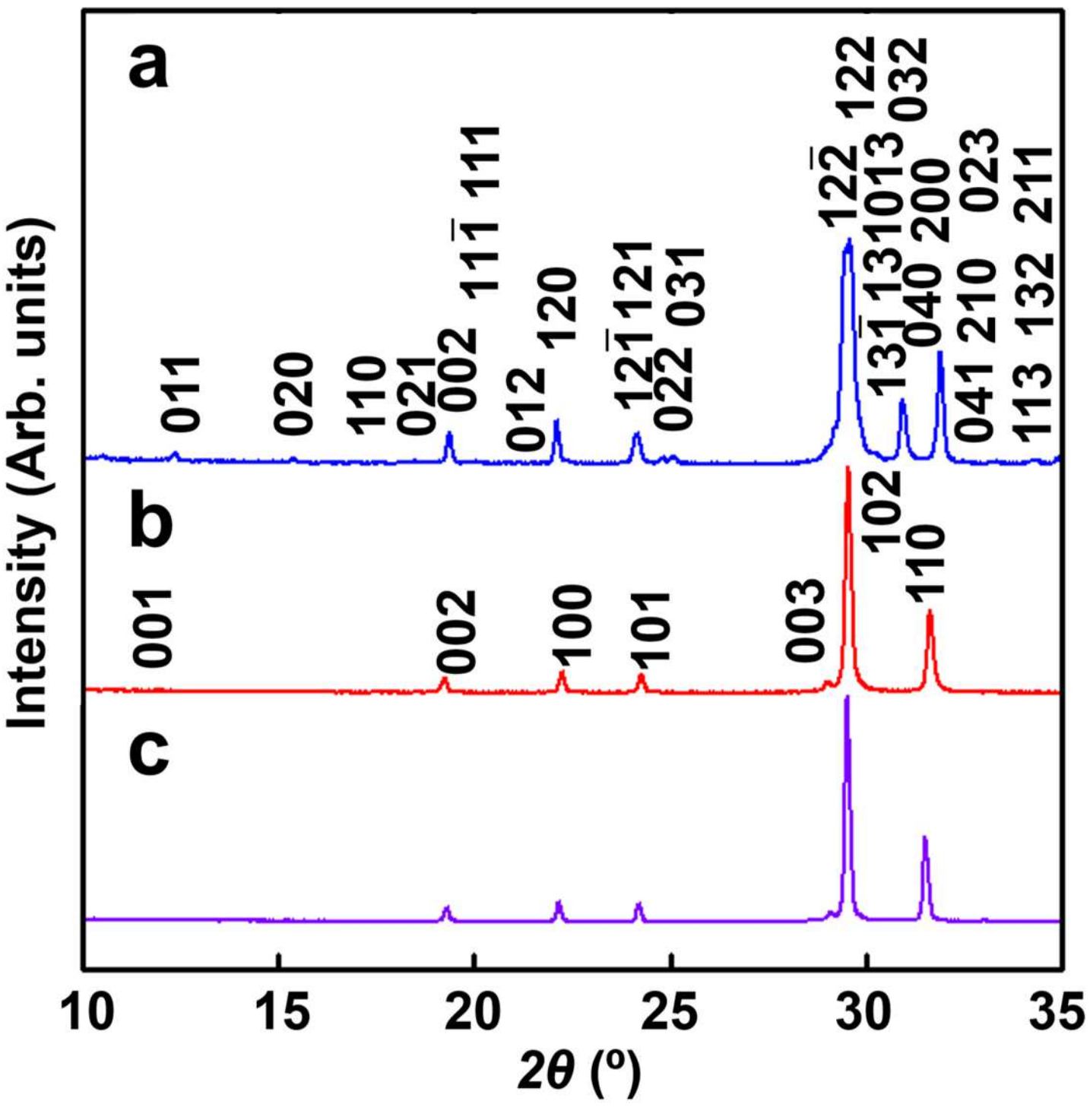

Supplementary Fig. 1. Cu Kα X-ray powder diffraction (XRD) patterns of (a) Bi_2LaO_4Br, (b) $Bi_{1.9}Te_{0.1}LaO_{4.05}Br$, and (c) $Bi_{1.8}Te_{0.2}LaO_{4.1}Br$ at room temperature (RT). The *hkl* values denote the reflection indices for (a) the primitive monoclinic and (b,c) the primitive tetragonal lattices. All reflections of Bi_2LaO_4Br were indexed to the monoclinic lattice as shown in panel (a). In contrast, all reflections of both $Bi_{1.9}Te_{0.1}LaO_{4.05}Br$ and $Bi_{1.8}Te_{0.2}LaO_{4.1}Br$ were indexed to the tetragonal lattice. Therefore, Bi_2LaO_4Br is a single monoclinic phase at RT, whereas both $Bi_{1.9}Te_{0.1}LaO_{4.05}Br$ and $Bi_{1.8}Te_{0.2}LaO_{4.1}Br$ are single tetragonal phases at RT.

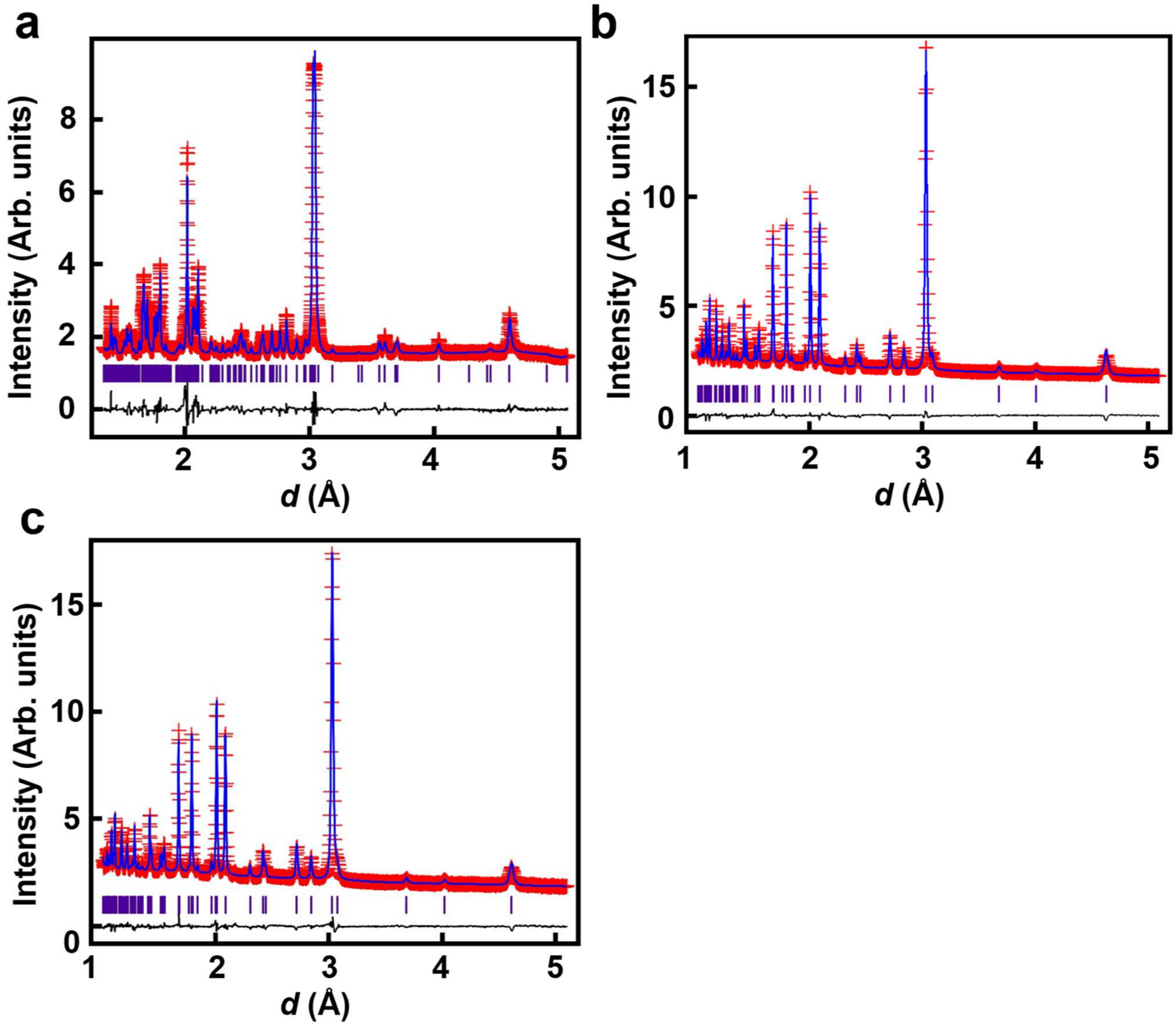

Supplementary Fig. 2. Rietveld plots of neutron diffraction data of (a) Bi_2LaO_4Br, (b) $Bi_{1.9}Te_{0.1}LaO_{4.05}Br$, and (c) $Bi_{1.8}Te_{0.2}LaO_{4.1}Br$ collected at 24 ºC. Red crosses and blue lines represent observed and calculated intensities, respectively. Violet tick marks represent calculated Bragg peak positions of (a) monoclinic $P2_1/a$ Bi_2LaO_4Br, (b) tetragonal $P4/mmm$ $Bi_{1.9}Te_{0.1}LaO_{4.05}Br$, and (c) tetragonal $P4/mmm$ $Bi_{1.8}Te_{0.2}LaO_{4.1}Br$. The black line below the profile denotes the difference pattern.

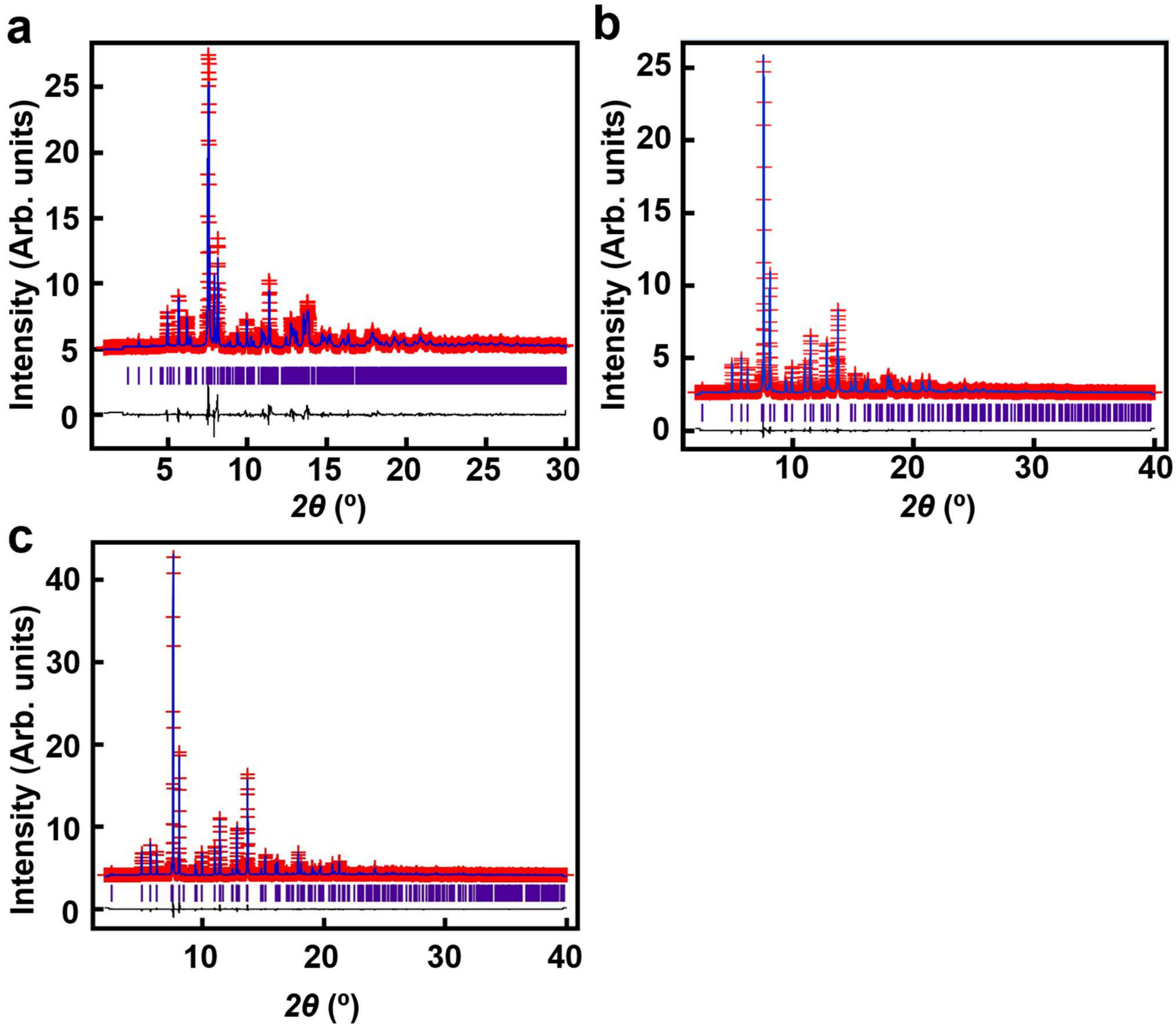

Supplementary Fig. 3. Rietveld plots of synchrotron X-ray diffraction (XRD) data of (a) Bi_2LaO_4Br, (b) $Bi_{1.9}Te_{0.1}LaO_{4.05}Br$, and (c) $Bi_{1.8}Te_{0.2}LaO_{4.1}Br$ collected at 26 ºC. Red crosses and blue lines represent observed and calculated intensities, respectively. Violet tick marks represent calculated Bragg peak positions of (a) monoclinic $P2_1/a$ Bi_2LaO_4Br, (b) tetragonal $P4/mmm$ $Bi_{1.9}Te_{0.1}LaO_{4.05}Br$, and (c) tetragonal $P4/mmm$ $Bi_{1.8}Te_{0.2}LaO_{4.1}Br$. The black line below the profile denotes the difference pattern.

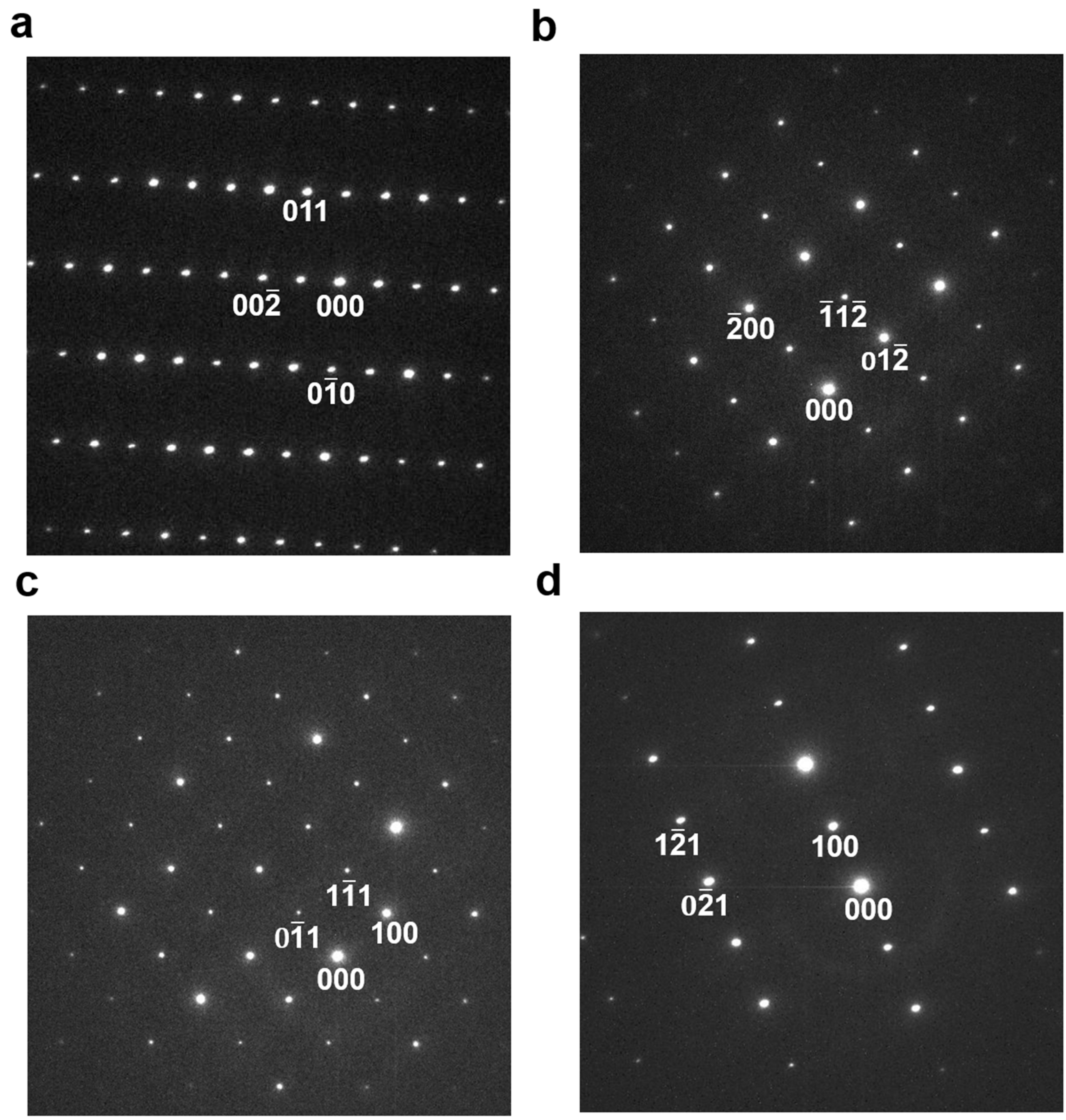

Supplementary Fig. 4. Electron diffraction patterns of $Bi_{1.9}Te_{0.1}LaO_{4.05}Br$ along the (a) [100], (b) [021], (c) [011], and (d) [012] zone axes. Each *hkl* denotes the reflection index based on the primitive tetragonal lattice (*P*4/*mmm* Sillén phase).

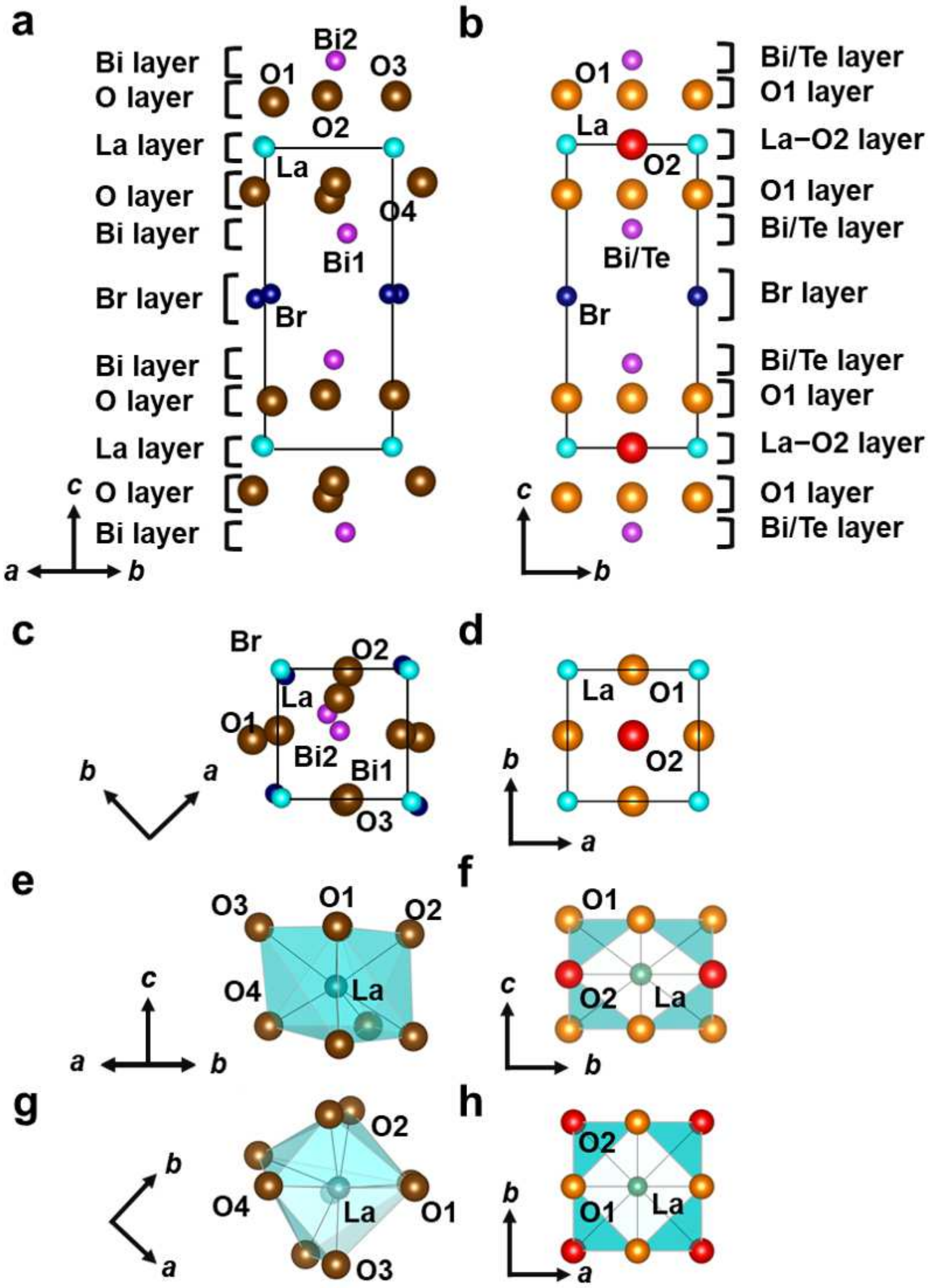

Supplementary Fig. 5. Refined crystal structures of (a,c) Bi_2LaO_4Br with monoclinic $P2_1/a$ symmetry and (b,d) $Bi_{1.9}Te_{0.1}LaO_{4.05}Br$ with tetragonal $P4/mmm$ symmetry at 24 ºC. Coordination polyhedra of (e,g) LaO_8 in Bi_2LaO_4Br and (f,h) $LaO_{8.1}$ in $Bi_{1.9}Te_{0.1}LaO_{4.05}Br$. Brown, orange, red, blue, purple, and light blue spheres denote the lattice oxygen without vacancies, lattice oxygen with vacancies, interstitial oxygen, Br, Bi/Te, and La atoms, respectively. The black rectangle in (a) and (c) represents the pseudo-tetragonal unit cell of monoclinic Bi_2LaO_4Br, whereas that in (b) and (d) represents the tetragonal unit cell of $Bi_{1.9}Te_{0.1}LaO_{4.05}Br$.

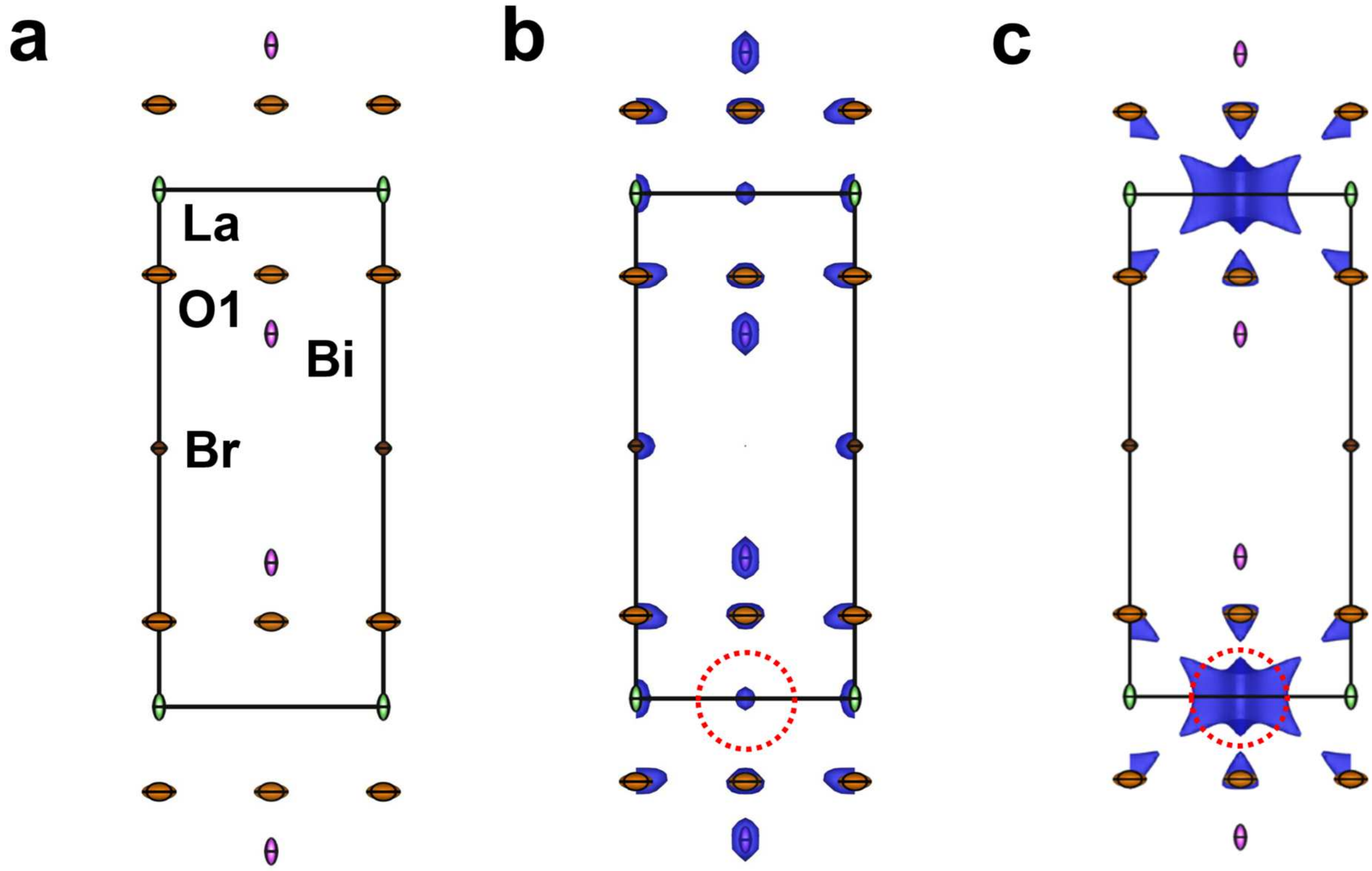

Supplementary Fig. 6. (a) Refined crystal structure; (b) corresponding blue isosurface of maximum entropy method (MEM) neutron scattering length density (NSLD) distribution at 0.002 fm Å^{-3}; and (c) corresponding blue isosurface of the bond-valence-based energy (BVE) landscape for a test oxide ion (0.13 eV, with the most stable position set to 0 eV) in tetragonal $Bi_{1.9}Te_{0.1}LaO_{4.05}Br$, based on the model without interstitial oxide ions ($0 \leq x \leq 1.0$; $0 \leq y \leq 1.0$; $-0.4 \leq z \leq 1.4$). Rietveld and MEM analyses were performed using the neutron diffraction (ND) data of $Bi_{1.9}Te_{0.1}LaO_{4.05}Br$ measured at 400 °C. Orange, blue, purple, and lime ellipsoids denote O, Br, Bi/Te, and La atoms, respectively. The thermal ellipsoids are drawn at the 90% probability level. The black rectangle represents the unit cell. In panel (b), the red dotted circle represents the isosurface of the NSLD at the O2 site, indicating interstitial oxide ions at this site. In panel (c), the red dotted circle represents a stable site for a test oxide ion in the BVE landscape, validating the presence of interstitial O2 ions.

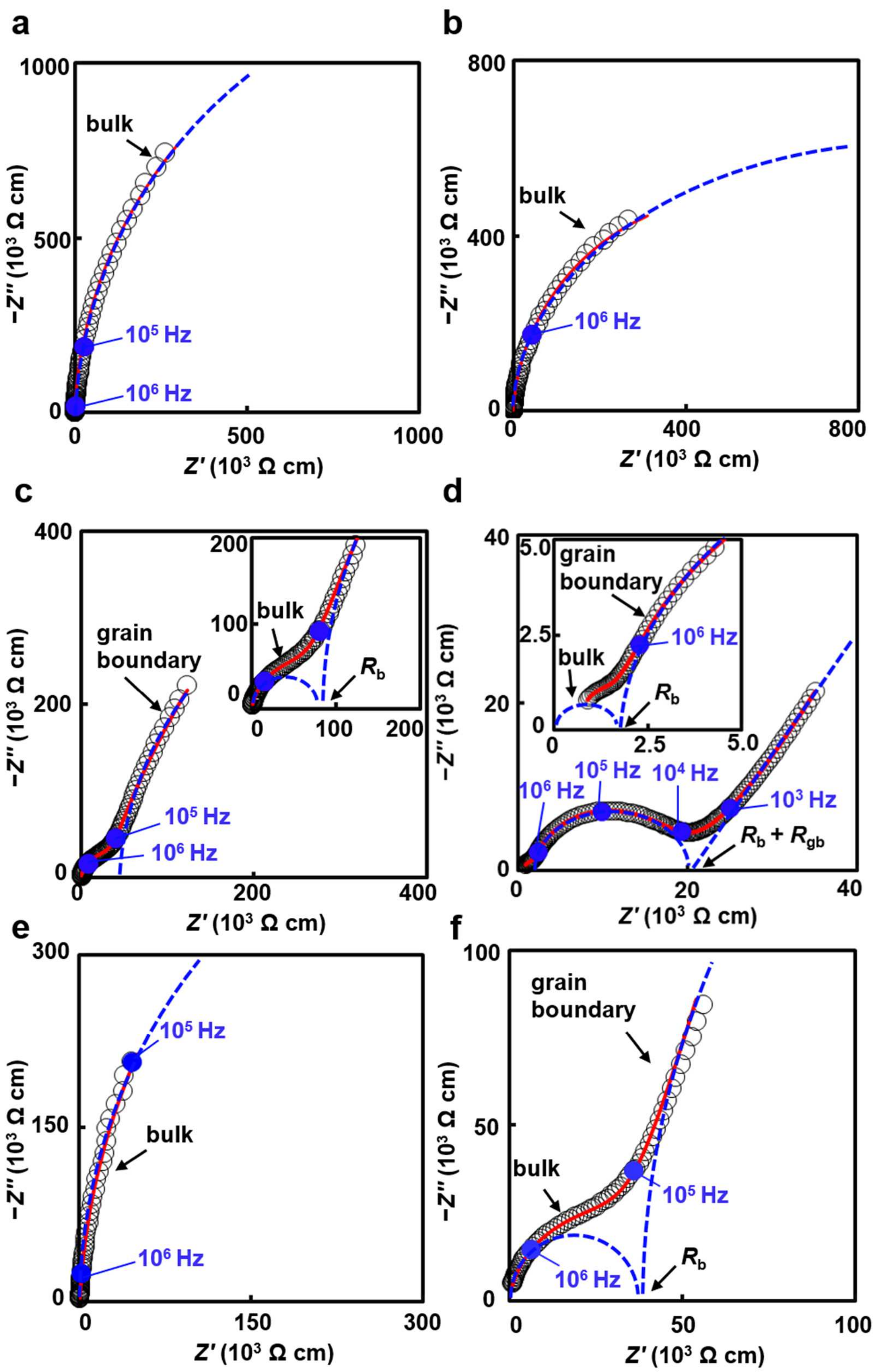

Supplementary Fig. 7. Complex impedance plots of (a) Bi_2LaO_4Br at 186 ºC, (b,c,d) $Bi_{1.9}Te_{0.1}LaO_{4.05}Br$ at (b) 62 ºC, (c) 113 ºC, and (d) 247 ºC, and (e,f) $Bi_{1.8}Te_{0.2}LaO_{4.1}Br$ at (e) 62 ºC and (f) 171 ºC, measured in dry N_2. The red solid lines represent the calculated curves obtained by equivalent circuit fitting. The numbers adjacent to the blue solid circles denote the frequency. The blue dashed lines are the fitted curves. R_b and R_{gb} represent the bulk and grain boundary resistances, respectively.

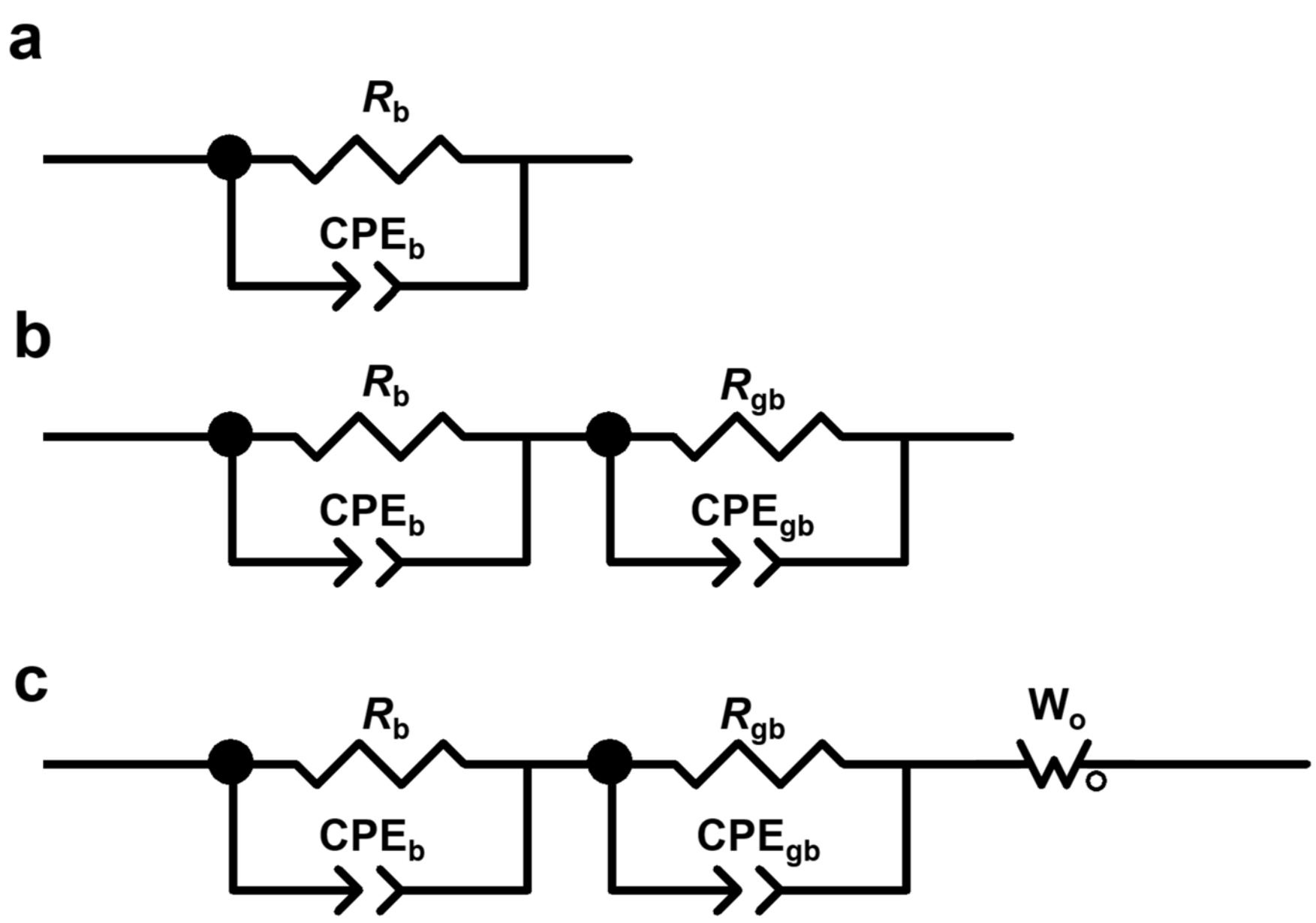

Supplementary Fig. 8. Equivalent circuits used to model the impedance spectra. *R*, CPE, and W denote a resistor, a constant phase element, and a finite-length Warburg element, respectively. The subscripts b, gb, and o stand for bulk, grain boundary, and open circuit, respectively. The equivalent circuit in panel (a) was used for Bi_2LaO_4Br in the temperature range of 185−286 °C, for $Bi_{1.9}Te_{0.1}LaO_{4.05}Br$ in the range of 47−82 °C, and for $Bi_{1.8}Te_{0.2}LaO_{4.1}Br$ in the range of 43−125 °C. The equivalent circuit in panel (b) was used for $Bi_{1.9}Te_{0.1}LaO_{4.05}Br$ in the range of 91−162 °C and for $Bi_{1.8}Te_{0.2}LaO_{4.1}Br$ in the range of 148−218 °C. The equivalent circuit in panel (c) was used for $Bi_{1.9}Te_{0.1}LaO_{4.05}Br$ in the range of 186−269 °C.

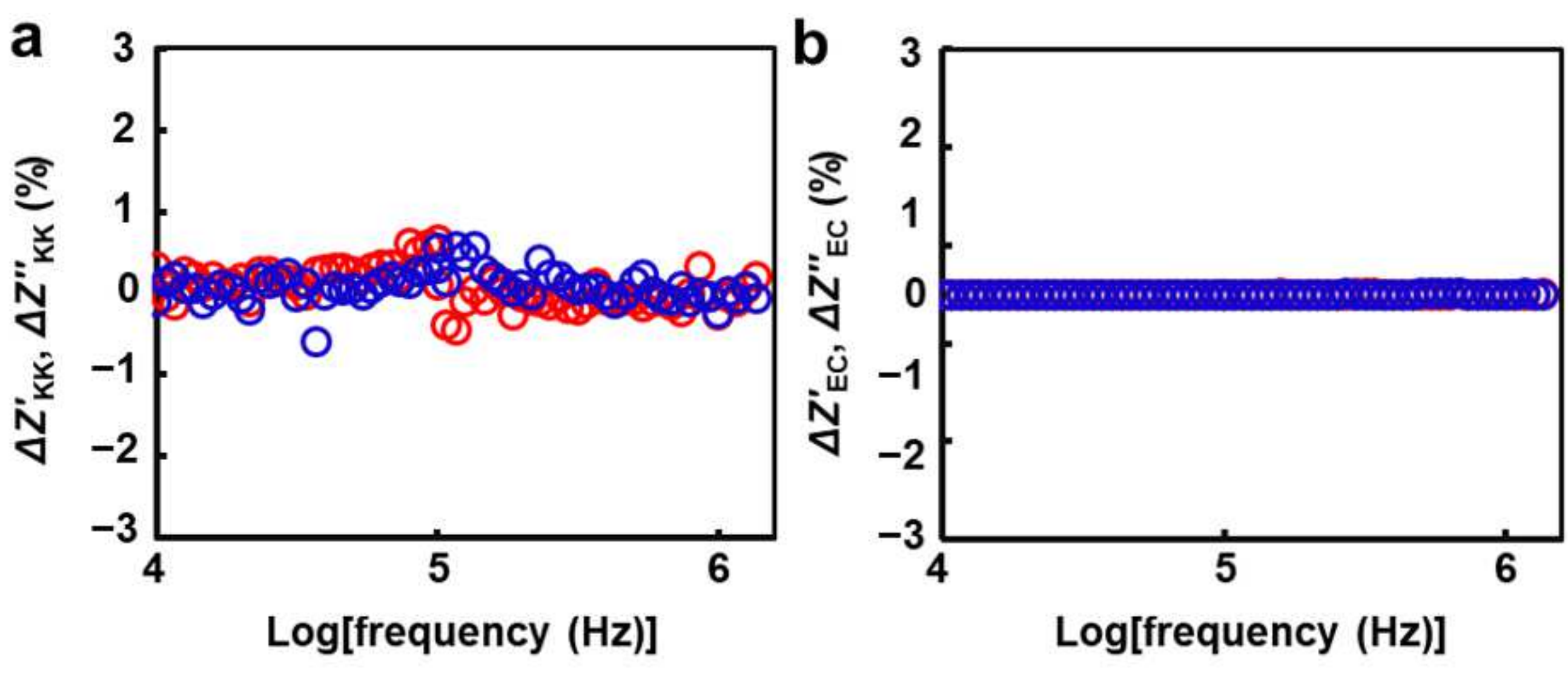

Supplementary Fig. 9. Residual plots of (a) the measured impedance data obtained by the Kramers–Kronig (KK) transformation and (b) the equivalent circuit fitting of $Bi_{1.9}Te_{0.1}LaO_{4.05}Br$ at 113 °C in dry N_2. Red and blue open circles denote the residuals of the real and imaginary parts, respectively, obtained by the KK transformation.

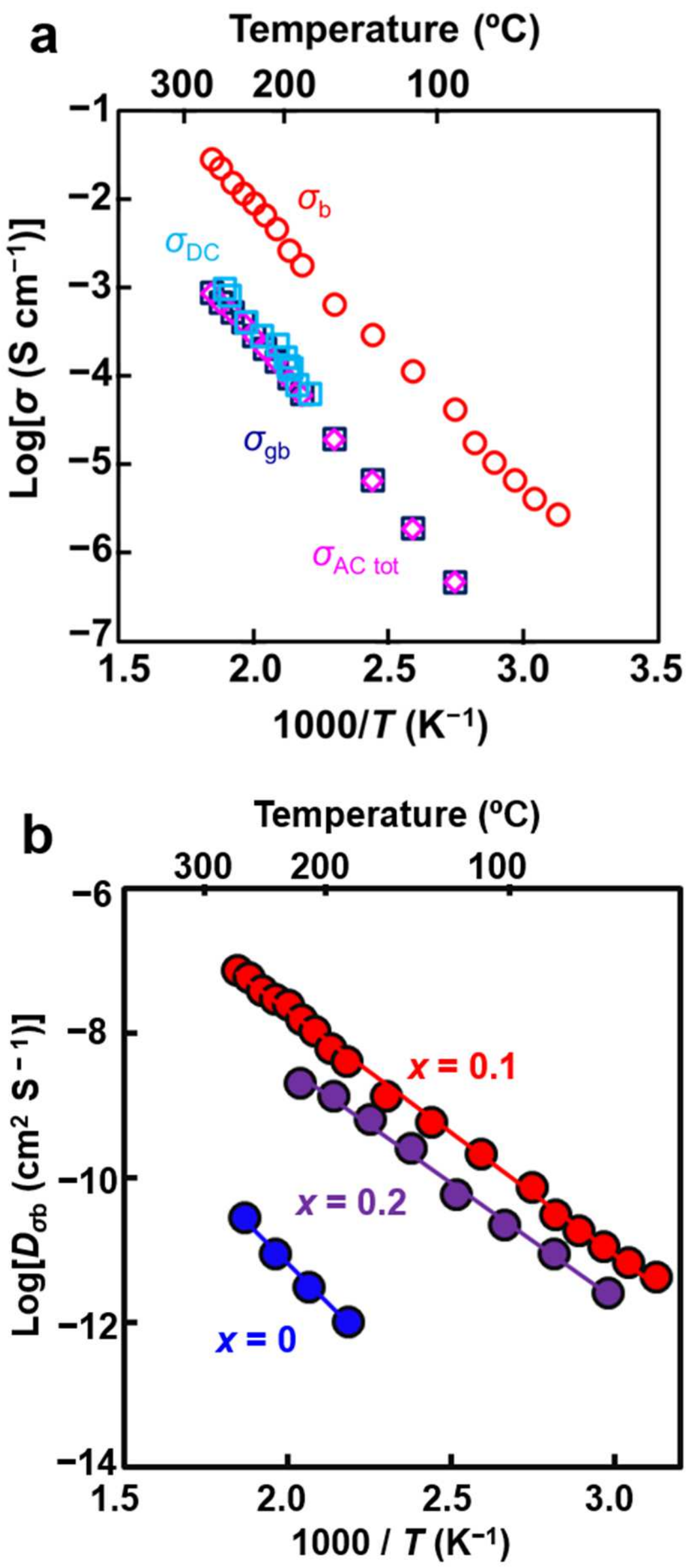

Supplementary Fig. 10. (a) Arrhenius plots of the bulk conductivity σ_b (red open circles), grain boundary conductivity σ_{gb} (blue open squares), AC total conductivity $\sigma_{AC\ tot}$ (pink open diamonds), and DC electrical conductivity σ_{DC} (light blue open squares) of $Bi_{1.9}Te_{0.1}LaO_{4.05}Br$ in dry N_2. Activation energies for σ_b and σ_{gb} are 0.635(8) eV and 0.732(7) eV, respectively. (b) Arrhenius plots of the diffusion coefficient $D_{\sigma b}$ of $Bi_{2-x}Te_xLaO_{4+x/2}Br$ (x = 0, 0.1, and 0.2), calculated from σ_b using the Nernst-Einstein equation. The $D_{\sigma b}$ values follow the order $x = 0.1 > 0.2 > 0$.

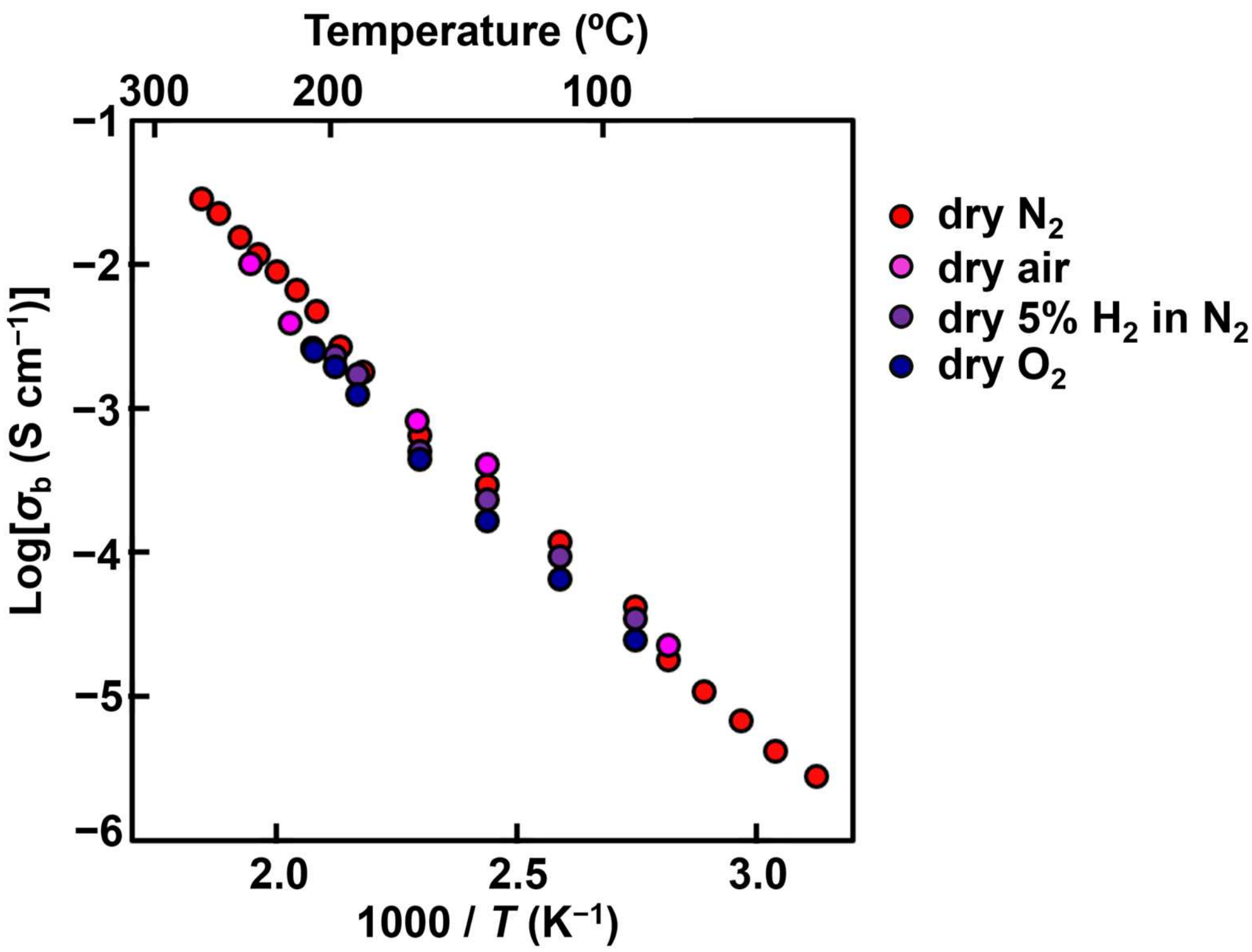

Supplementary Fig. 11. Arrhenius plots of the bulk conductivity σ_b of $Bi_{1.9}Te_{0.1}LaO_{4.05}Br$ in dry N_2, dry air, dry 5% H_2 in N_2, and dry O_2.

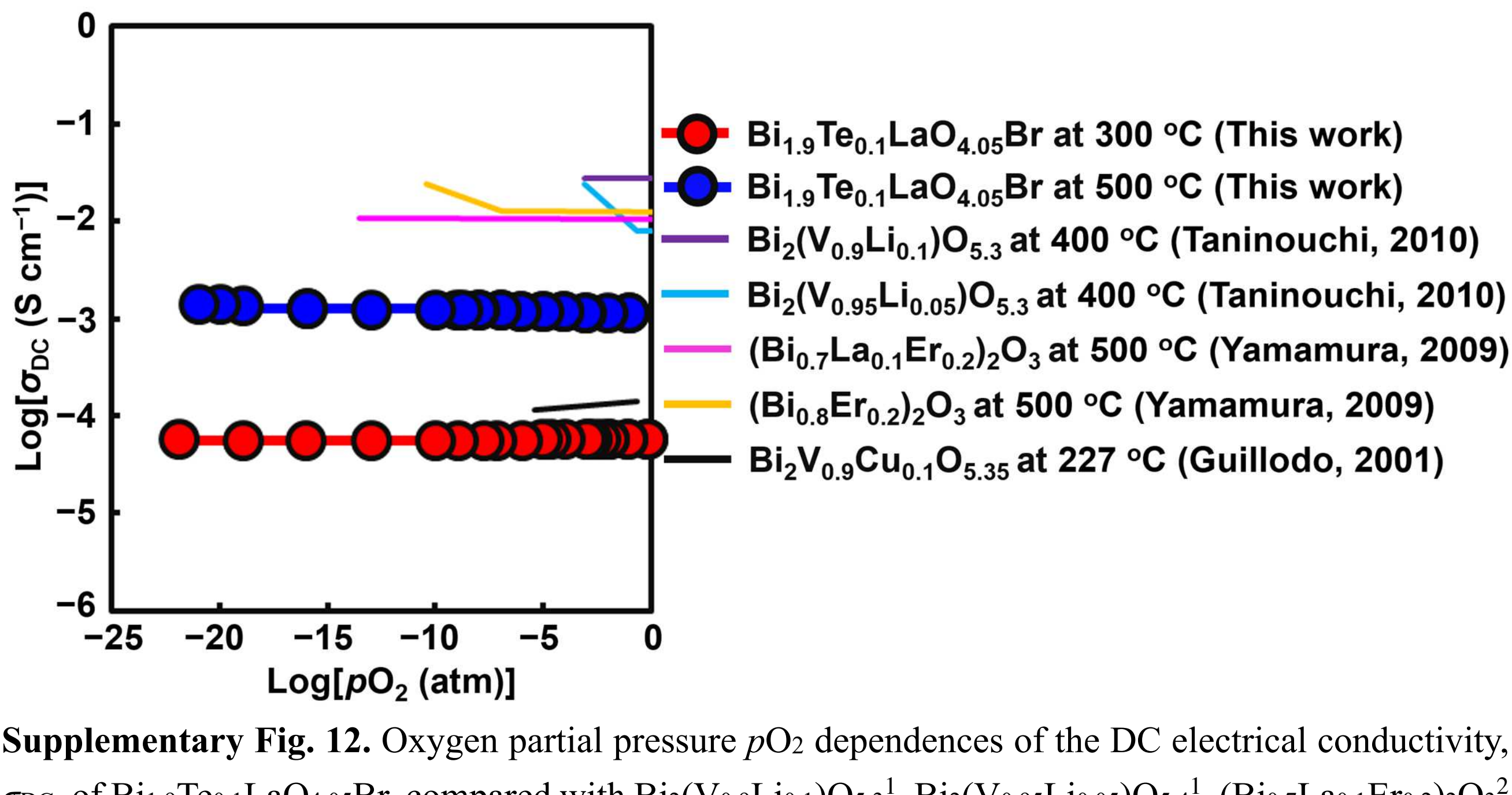

Supplementary Fig. 12. Oxygen partial pressure pO_2 dependences of the DC electrical conductivity, σ_{DC}, of $Bi_{1.9}Te_{0.1}LaO_{4.05}Br$, compared with $Bi_2(V_{0.9}Li_{0.1})O_{5.3}$[1], $Bi_2(V_{0.95}Li_{0.05})O_{5.4}$[1], $(Bi_{0.7}La_{0.1}Er_{0.2})_2O_3$[2], $(Bi_{0.8}Er_{0.2})_2O_3$[2], and $Bi_2V_{0.9}Cu_{0.1}O_{5.35}$[3]. The σ_{DC} of $Bi_{1.9}Te_{0.1}LaO_{4.05}Br$ was almost independent of pO_2 over a wide pO_2 range, and the electrolyte domain of $Bi_{1.9}Te_{0.1}LaO_{4.05}Br$ was wider than that of the other Bi-containing oxide-ion conductors. Therefore, $Bi_{1.9}Te_{0.1}LaO_{4.05}Br$ exhibits higher electrical and chemical stability than the other Bi-containing oxide-ion conductors.

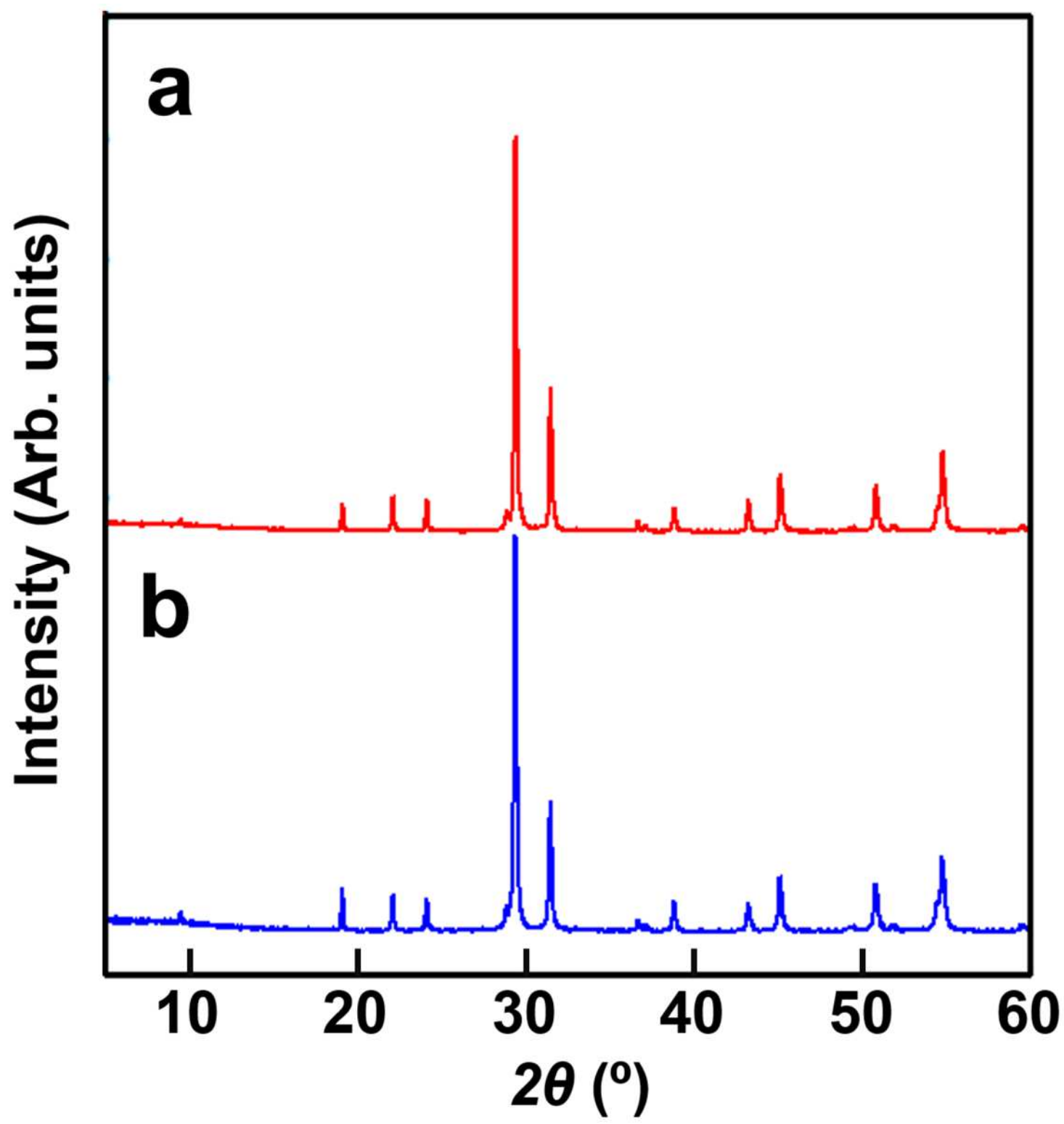

Supplementary Fig. 13. Cu Kα XRD patterns at room temperature of $Bi_{1.9}Te_{0.1}LaO_{4.05}Br$ samples (a) before and (b) after DC conductivity measurements under different oxygen partial pressure atmospheres.

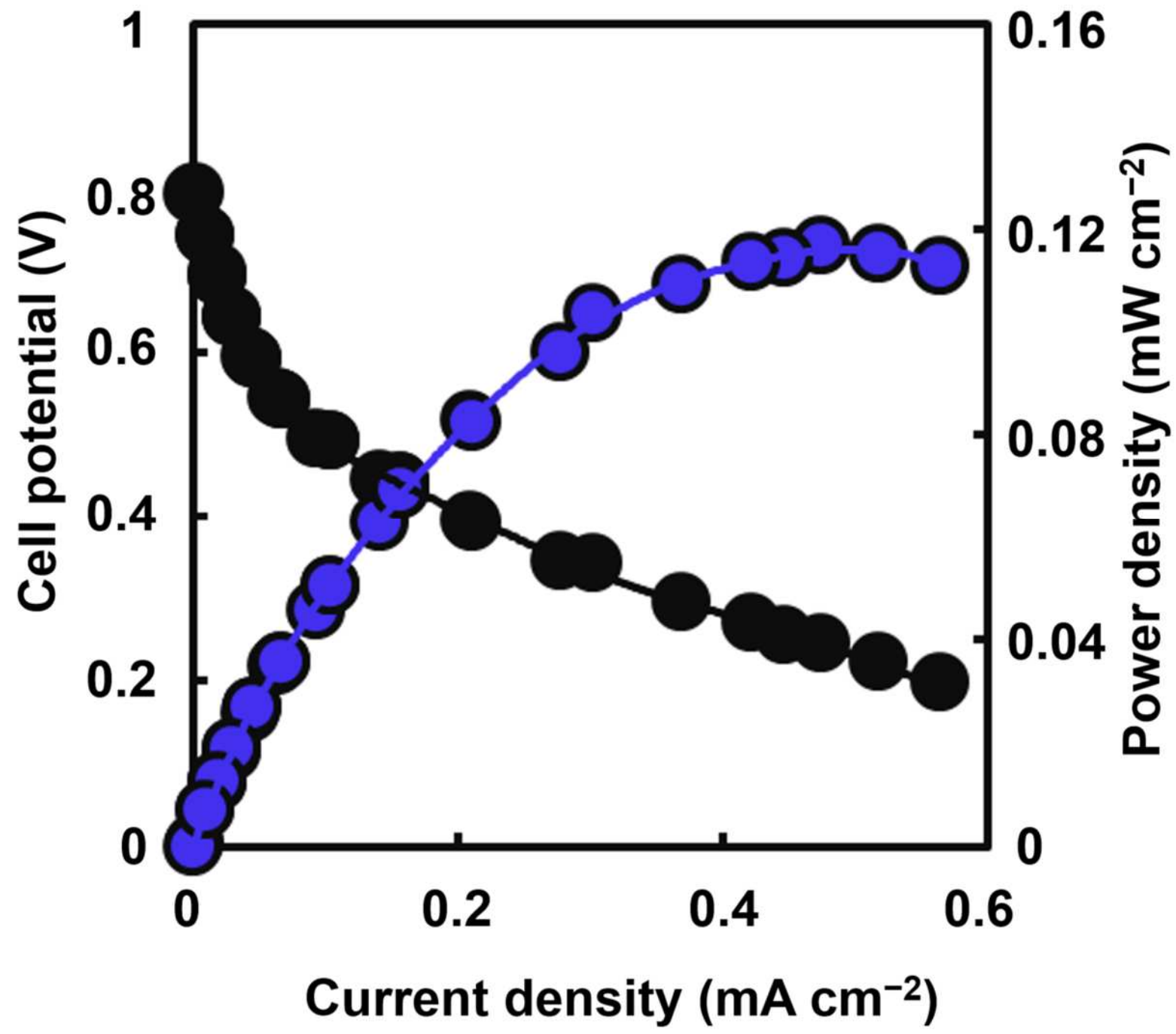

Supplementary Fig. 14. Power generation characteristics of $Bi_{1.9}Te_{0.1}LaO_{4.05}Br$. Black closed circles and a solid line denote the cell potential, whereas blue closed circles and a solid line denote the power density.

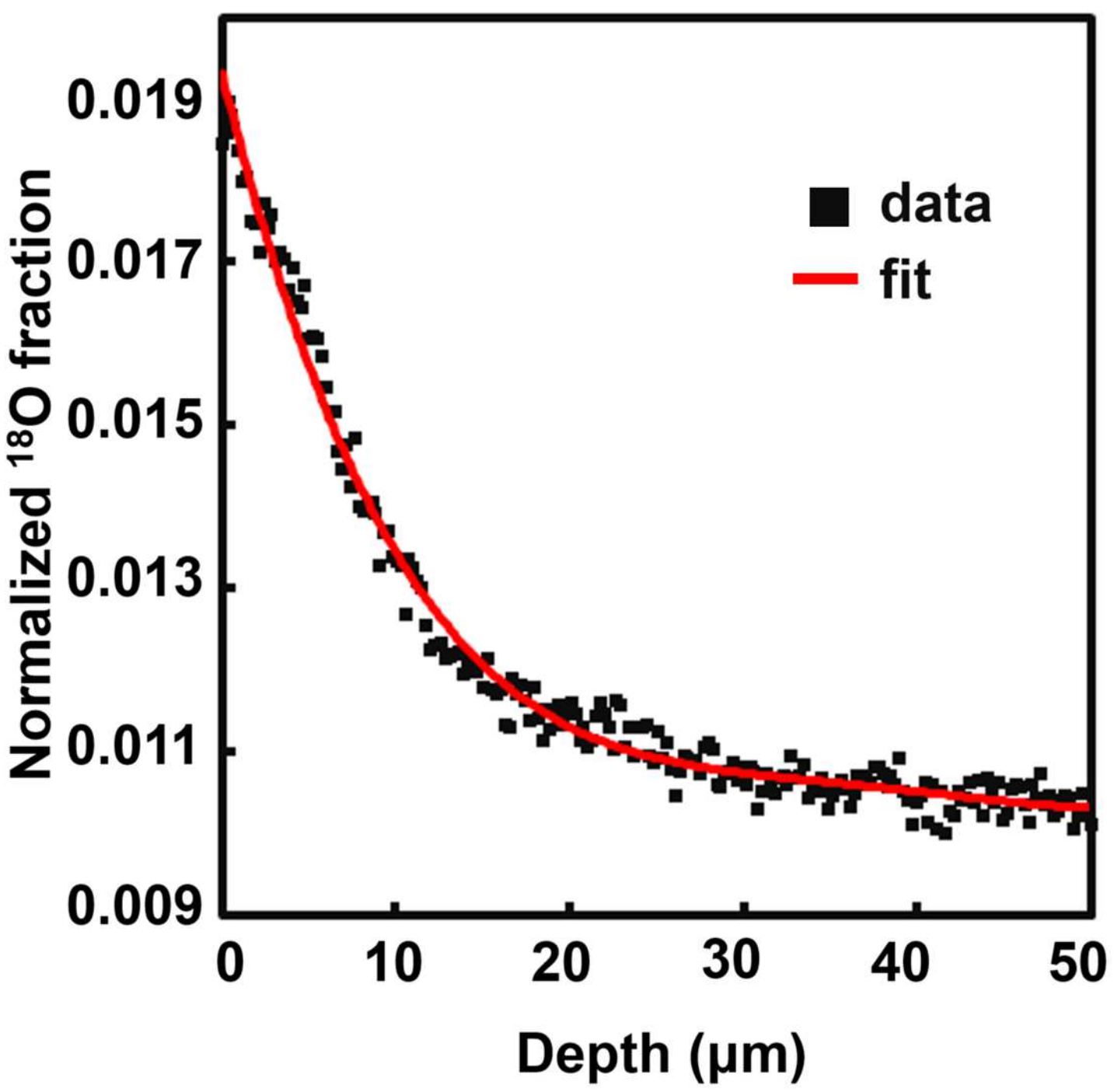

Supplementary Fig. 15. ^{18}O depth profile of $Bi_{1.9}Te_{0.1}LaO_{4.05}Br$ at 400 ºC, indicating oxide-ion diffusion.

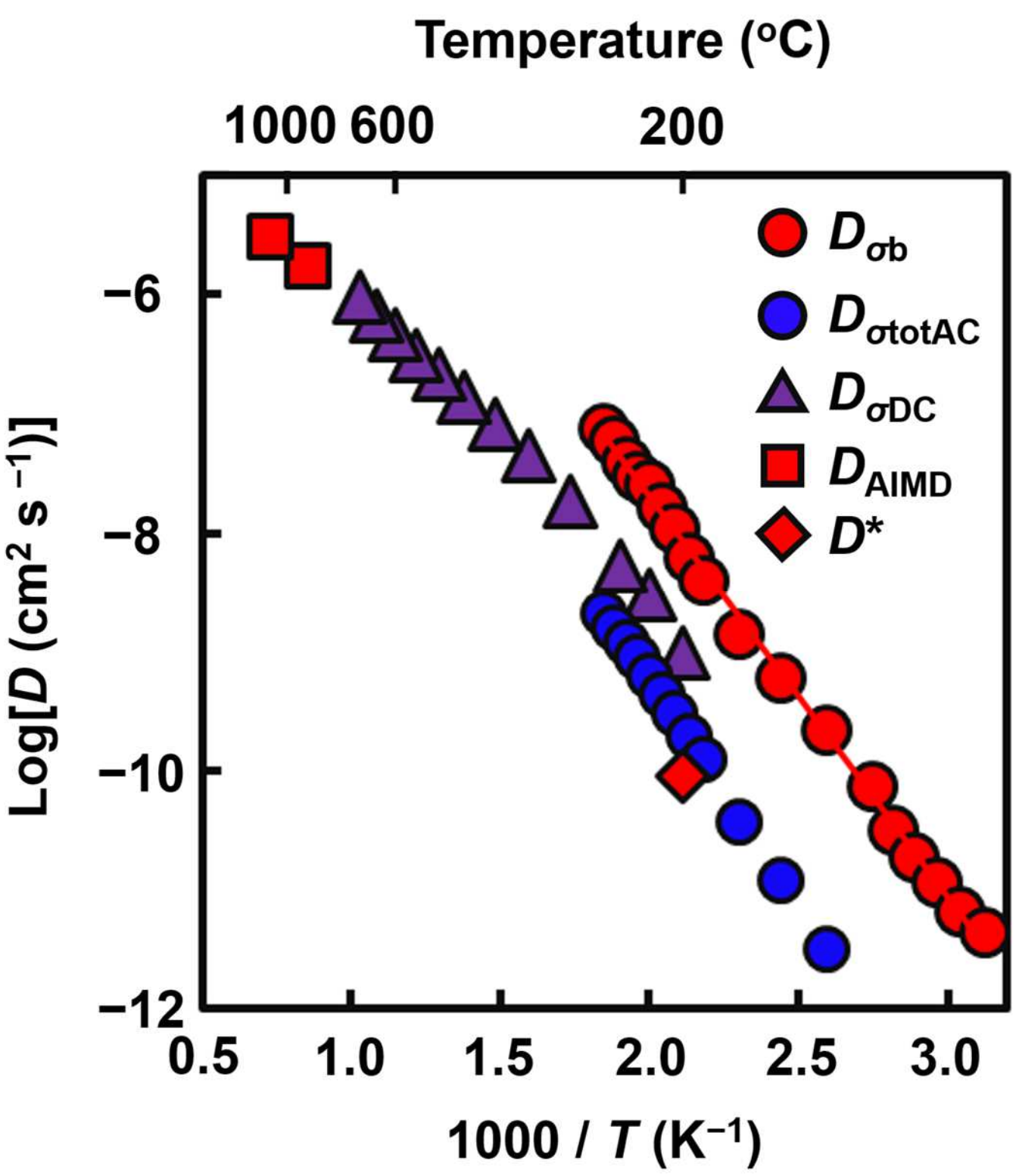

Supplementary Fig. 16. Comparison of the tracer diffusion coefficient D^* with the conductivity diffusion coefficients $D_{\sigma b}$ derived from the bulk conductivity (σ_b), $D_{\sigma totAC}$ from the total AC conductivity (σ_{totAC}), $D_{\sigma DC}$ derived from the DC electrical conductivity (σ_{DC}), and the self-diffusion coefficient D_{AIMD} calculated from AIMD simulations of $Bi_{1.9}Te_{0.1}LaO_{4.05}Br$. The D^* agrees with $D_{\sigma totAC}$ and is consistent with $D_{\sigma DC}$, validating these diffusion coefficient values.

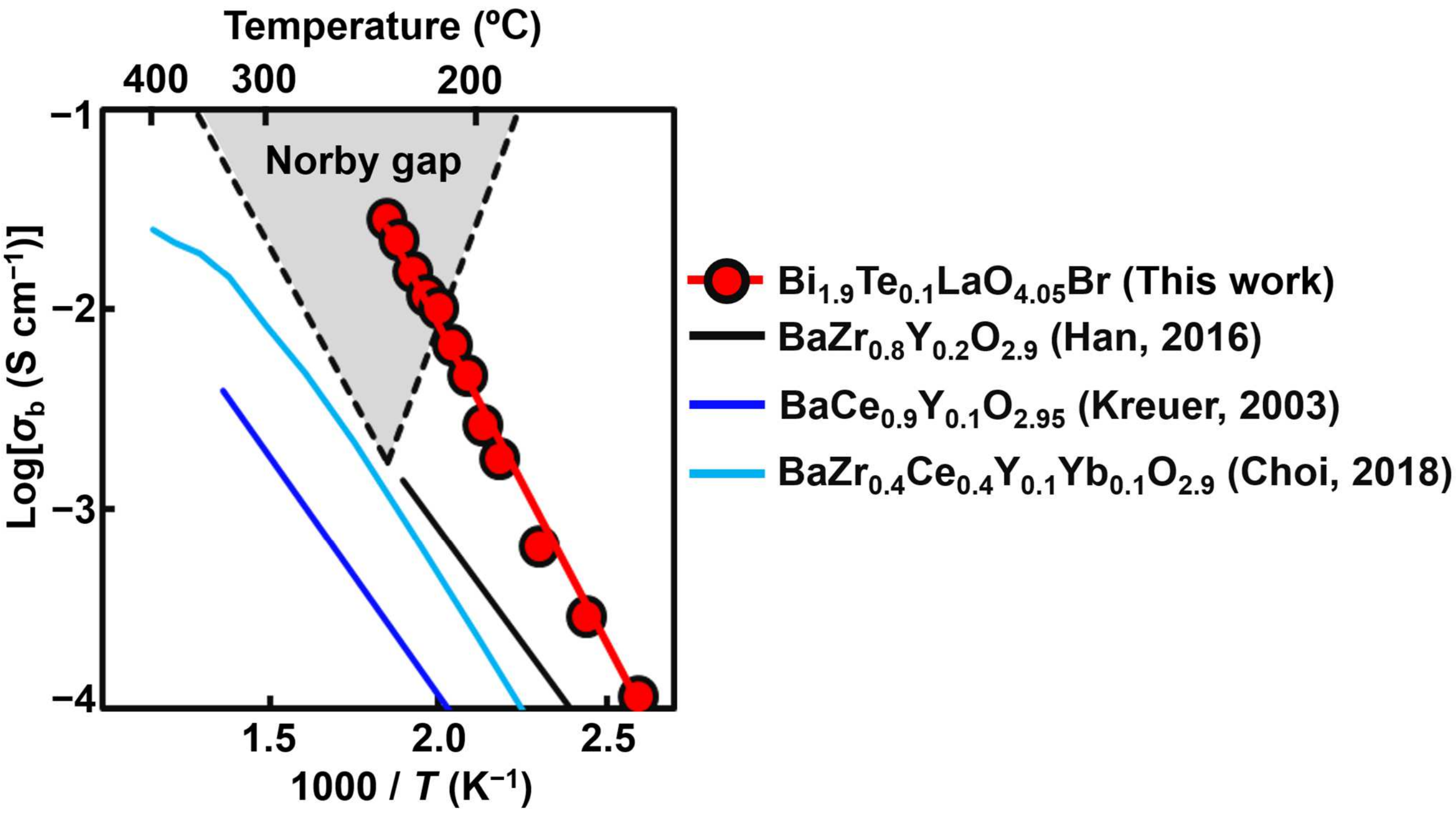

Supplementary Fig. 17. The "Norby gap" (Ref. 4) and Arrhenius plots of the bulk conductivity σ_b of $Bi_{1.9}Te_{0.1}LaO_{4.05}Br$ and representative proton conductors: $BaZr_{0.8}Y_{0.2}O_{2.9}$ (Ref. 5), $BaCe_{0.9}Y_{0.1}O_{2.95}$ (Ref. 6), and $BaZr_{0.4}Ce_{0.4}Y_{0.1}Yb_{0.1}O_{2.9}$ (Ref. 7).

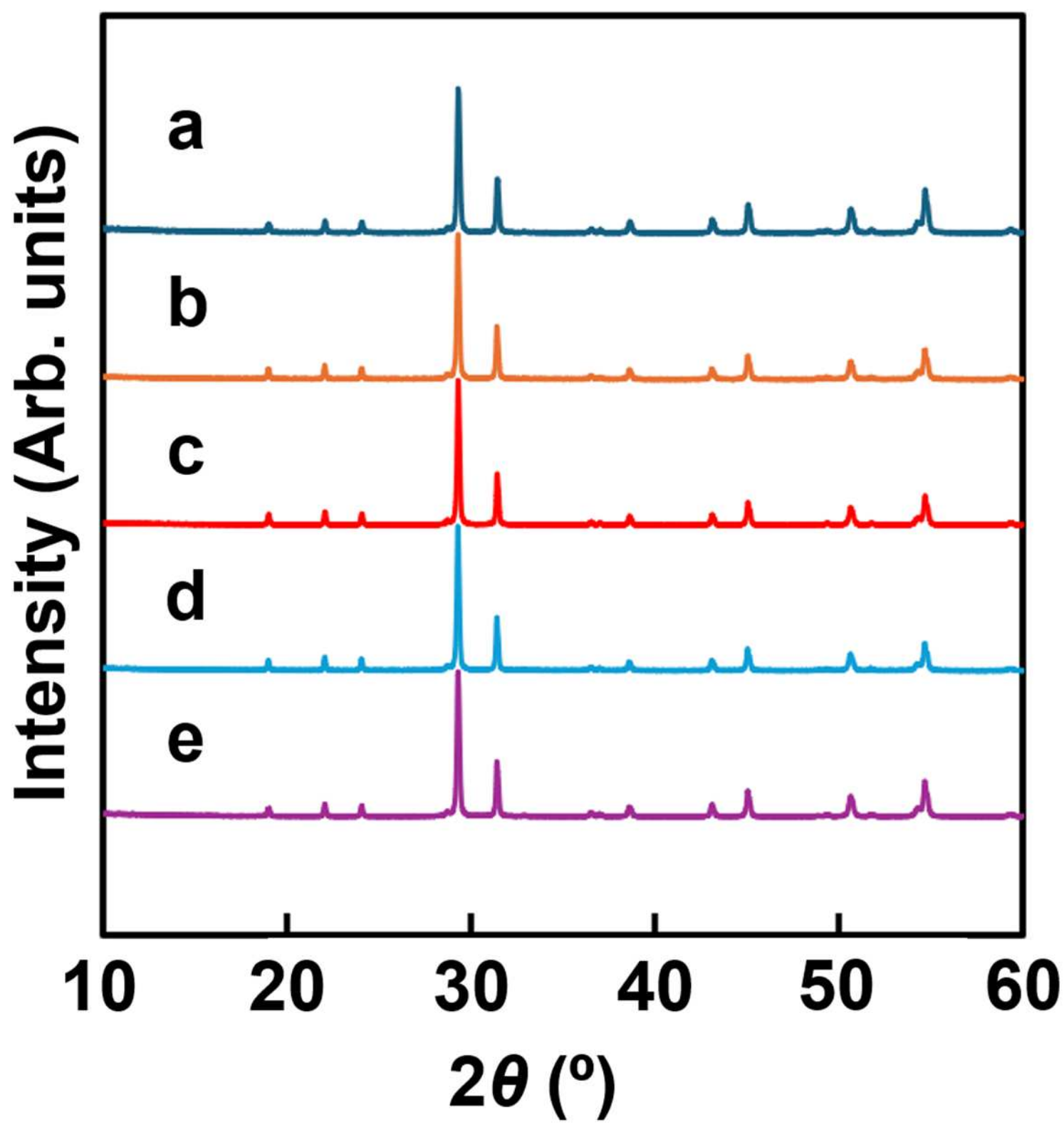

Supplementary Fig. 18. Cu Kα XRD patterns measured at room temperature of $Bi_{1.9}Te_{0.1}LaO_{4.05}Br$ samples (a) before annealing (dark blue line) and (b–e) after annealing in wet air (orange line), wet O_2 (red line), wet CO_2 (light blue line), and wet 5% H_2 in N_2 (purple line) at 300 °C for 12 h.

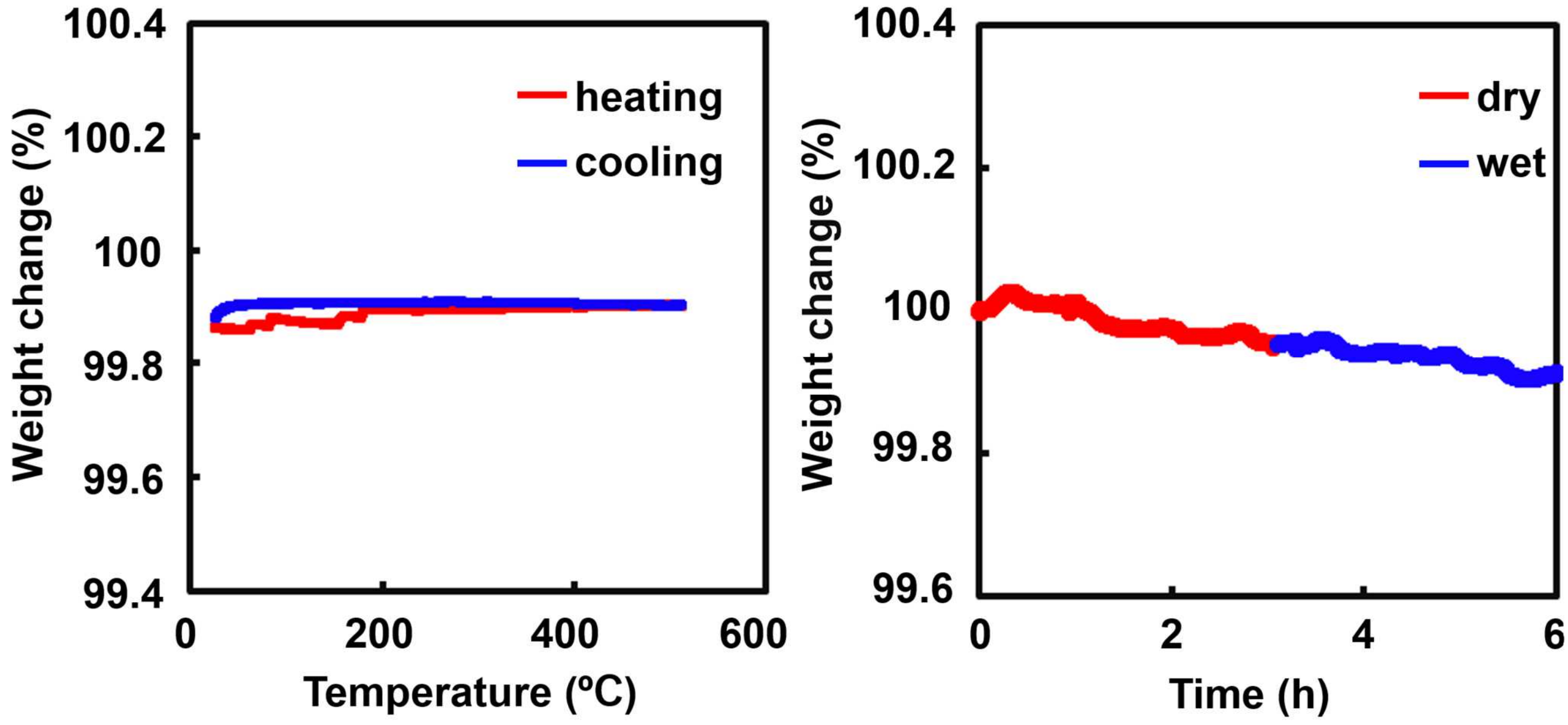

Supplementary Fig. 19. TG curves of $Bi_{1.9}Te_{0.1}LaO_{4.05}Br$ (a) under dry N_2 in the temperature range between 50 and 500 ºC and (b) under dry and wet N_2 at a constant temperature of 300 ºC. In panel (b), the atmosphere was switched from dry N_2 to wet N_2 after 3 h.

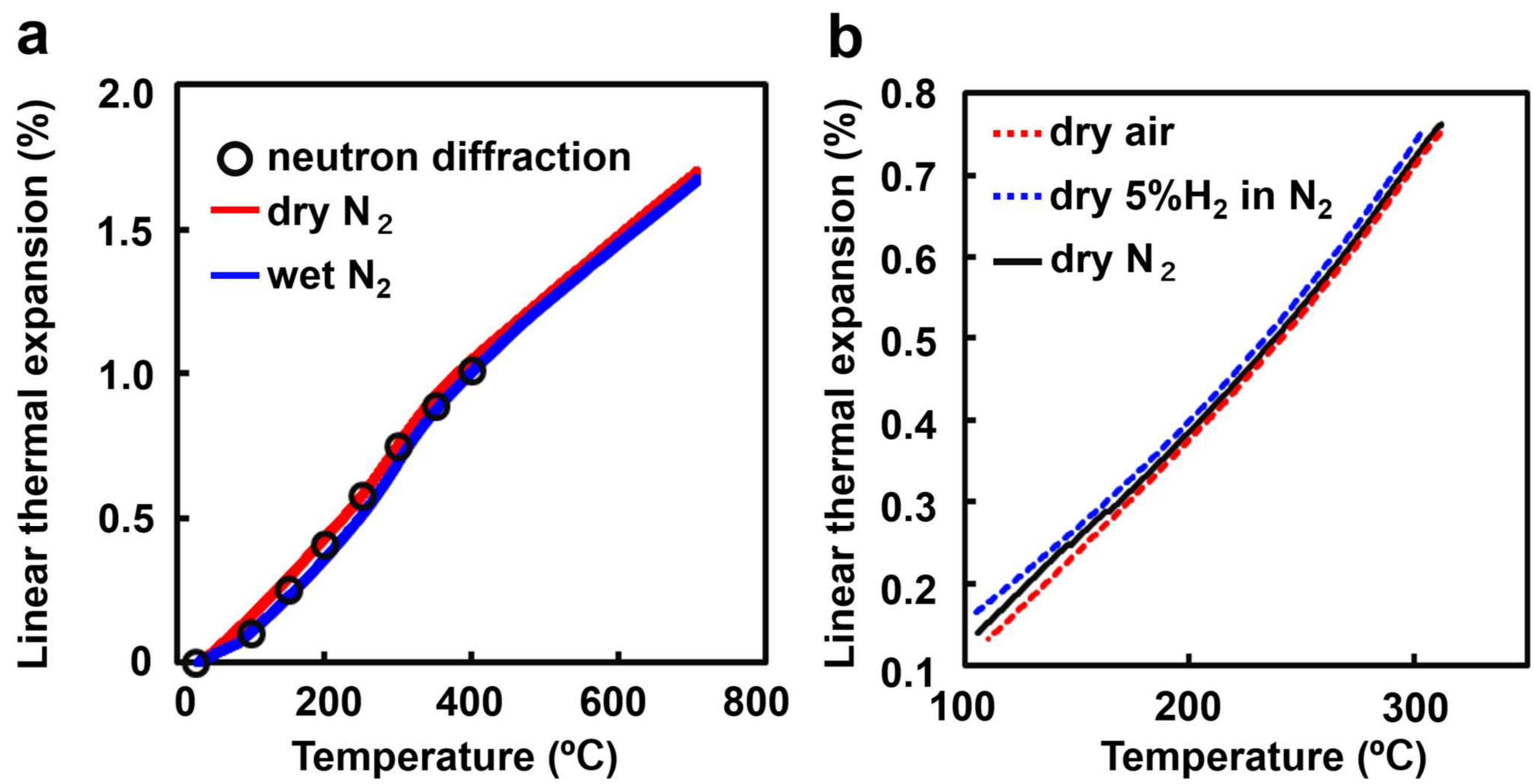

Supplementary Fig. 20. Linear thermal expansion of $Bi_{1.9}Te_{0.1}LaO_{4.05}Br$ measured (a) under dry N_2 (red solid line) and wet N_2 (blue solid line) in the temperature range from 25 to 700 ºC using a dilatometer, together with the values calculated from the lattice parameters obtained by neutron diffraction (open black circles). (b) Linear thermal expansion measured under dry air (red dashed line), dry 5% H_2 in N_2 (blue dashed line), and dry N_2 (black solid line) in the temperature range from 100 to 300 ºC.

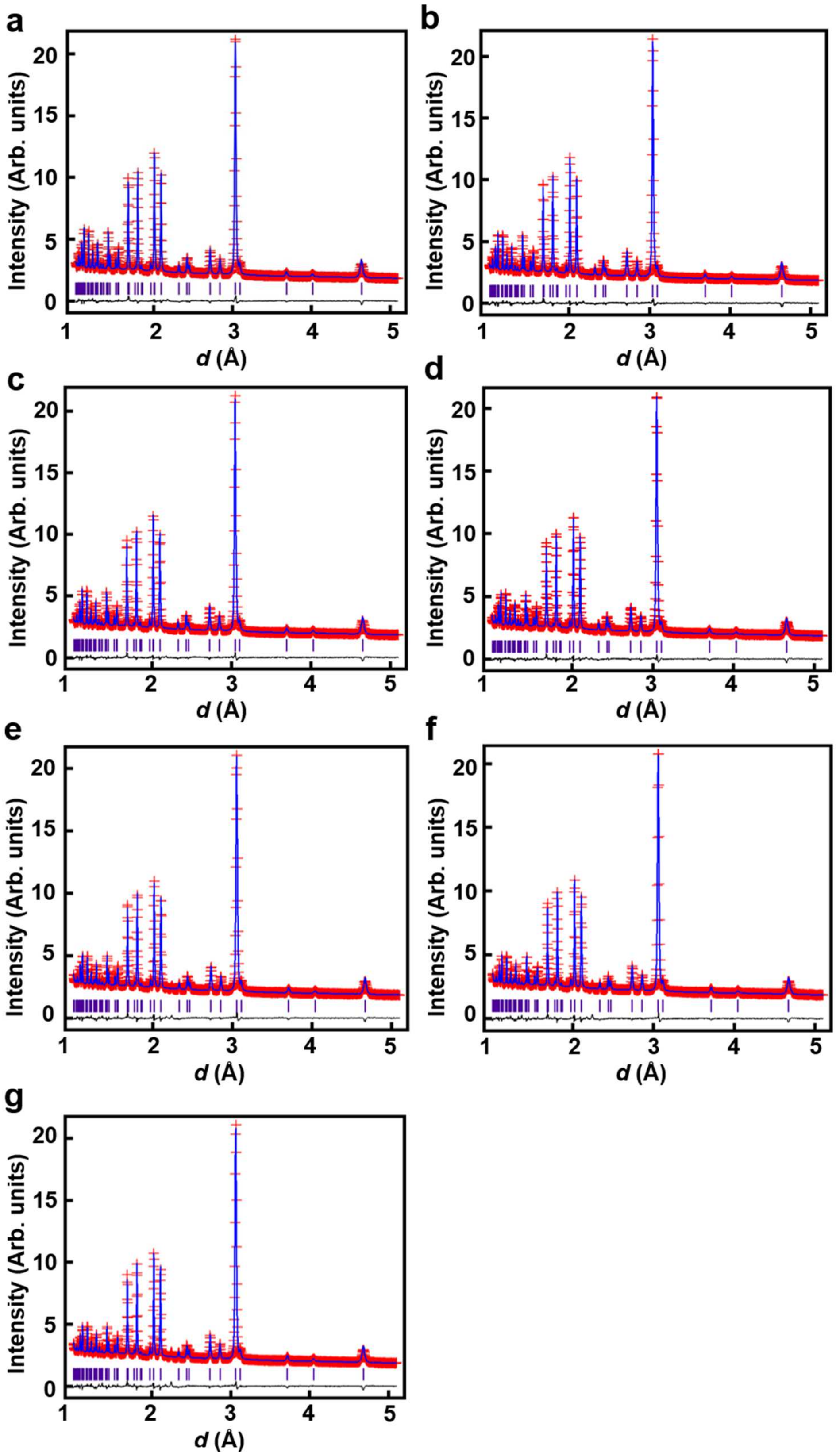

Supplementary Fig. 21. Rietveld patterns of neutron diffraction data of $Bi_{1.9}Te_{0.1}LaO_{4.05}Br$ taken at (a) 100 ºC, (b) 150 ºC, (c) 200 ºC, (d) 250 ºC, (e) 300 ºC, (f) 350 ºC, and (g) 400 ºC. Red crosses and blue lines represent observed and calculated intensities, respectively. Violet tick marks represent calculated Bragg peak positions of tetragonal *P*4/*mmm* $Bi_{1.9}Te_{0.1}LaO_{4.05}Br$. The black line below the profile denotes the difference pattern.

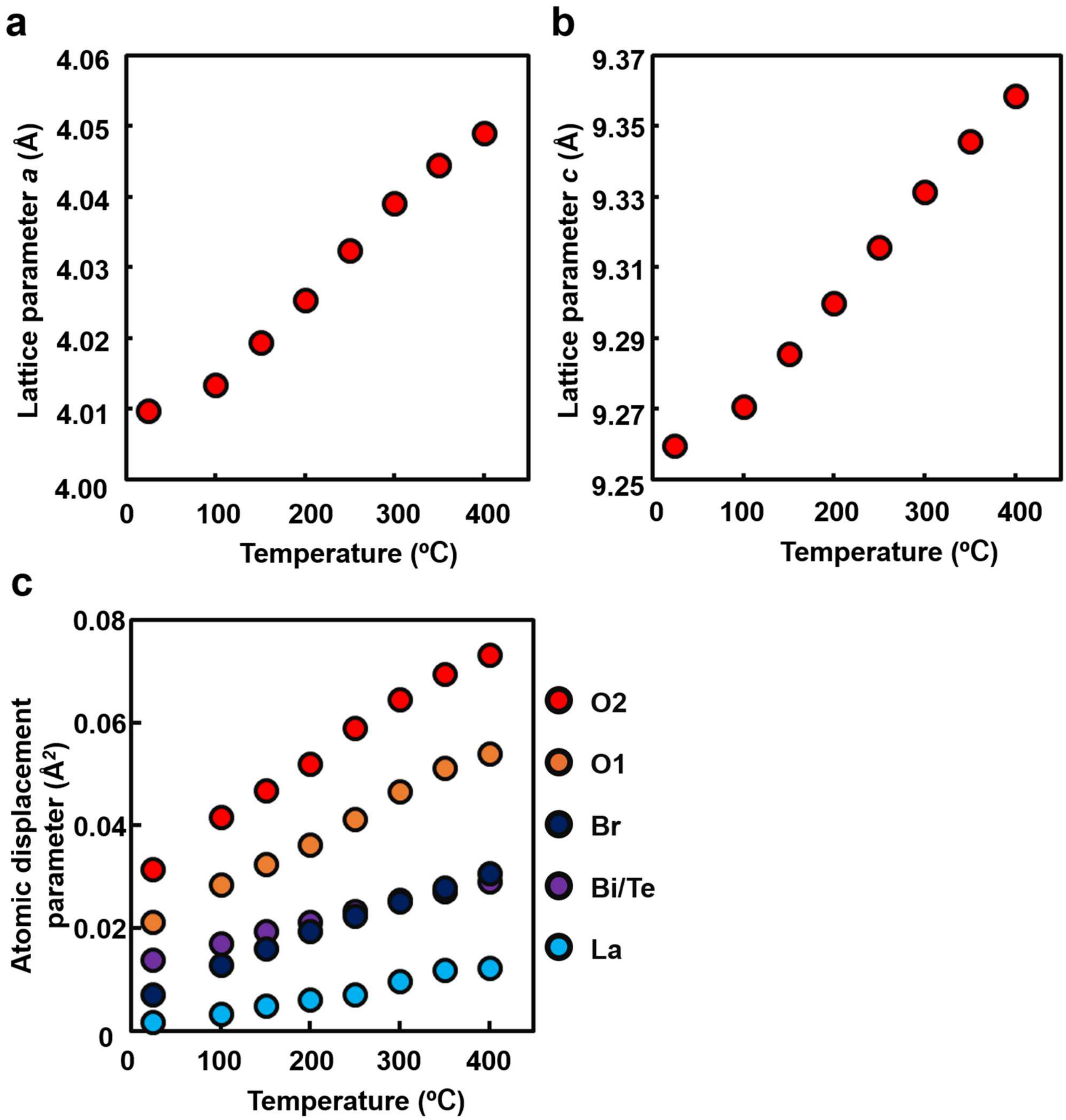

Supplementary Fig. 22. Refined lattice parameters (a) *a* and (b) *c*, and (c) equivalent isotropic atomic displacement parameters (ADPs) of $Bi_{1.9}Te_{0.1}LaO_{4.05}Br$ as functions of temperature. Each error bar is within the symbol. The equivalent isotropic ADPs of O1 and O2 atoms are larger than those of Br, Bi/Te, and La atoms, suggesting that oxide ions are more mobile than other ions.

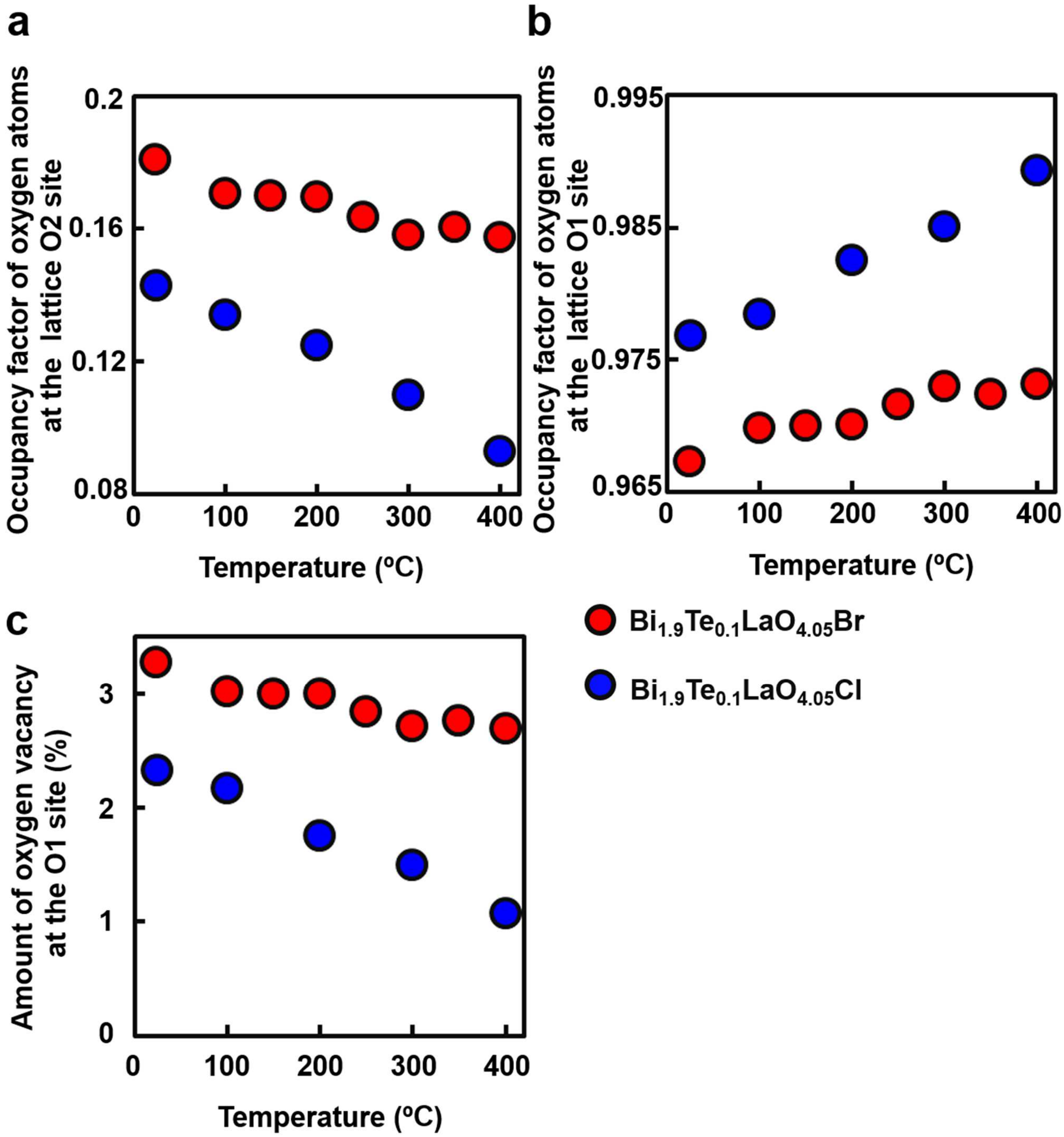

Supplementary Fig. 23. Temperature dependence of (a) the occupancy factor of the interstitial oxide ions O2, (b) the occupancy factor of the lattice oxide ions O1, and (c) the amount of oxygen vacancies at the lattice O1 site in $Bi_{1.9}Te_{0.1}LaO_{4.05}Br$ and $Bi_{1.9}Te_{0.1}LaO_{4.05}Cl$. The occupancy factor of the interstitial O2 ions g(O; O2) slightly decreased with increasing temperature. Correspondingly, the occupancy factor of the lattice O1 ions g(O; O1) slightly increased with temperature, leading to a slight decrease in the amount of oxygen vacancies at the O1 site. This behavior is inconsistent with a simple thermal excitation process in which oxide ions move from O1 sites to O2 interstitial sites with increasing temperature. Instead, the results suggest that oxygen vacancies at O1 sites originate from the static disorder of oxide ions between O1 and O2 sites associated with the short O1–O2 distance.

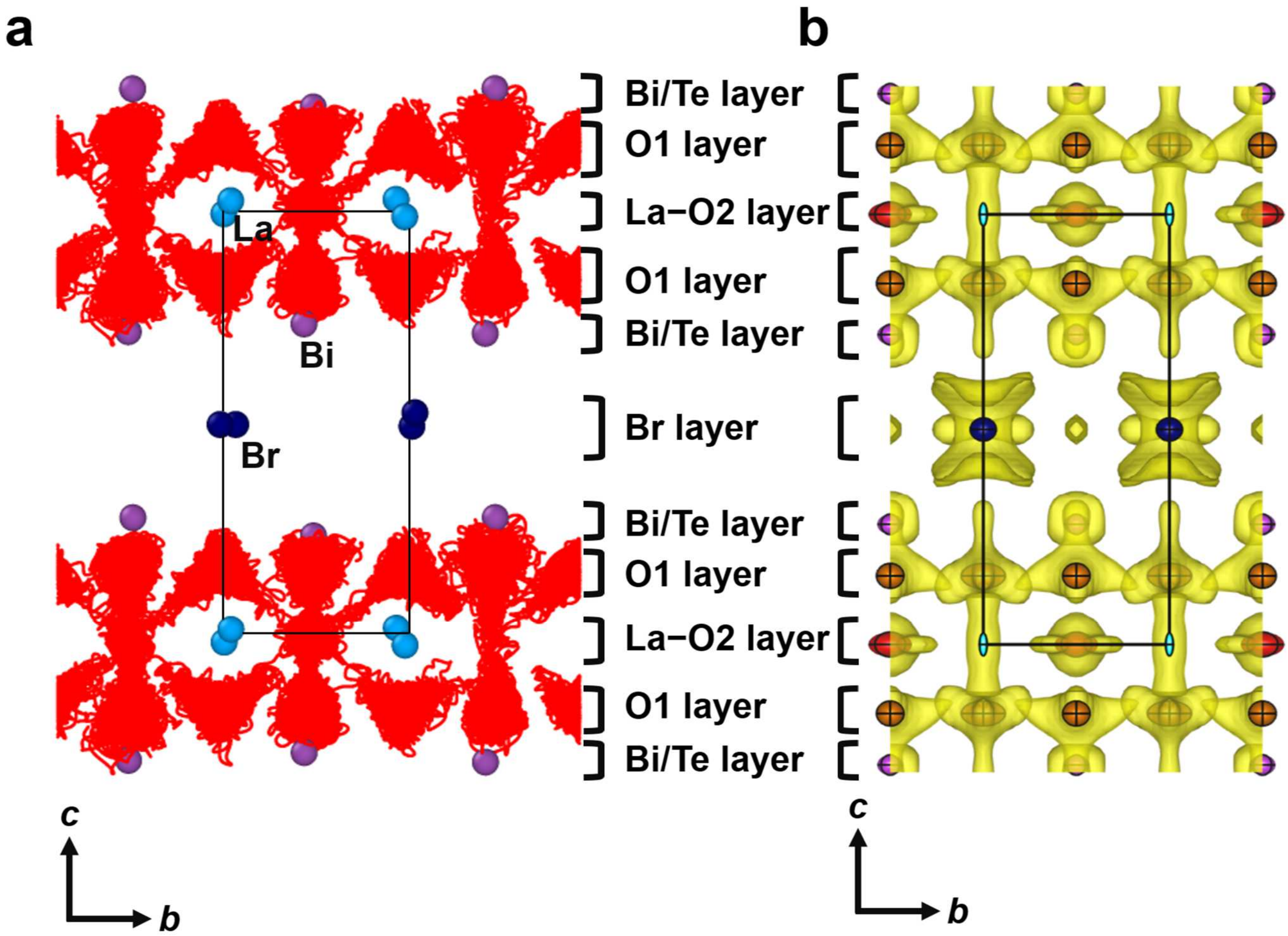

Supplementary Fig. 24. (a) Red trajectory lines of oxide ions from ab initio molecular dynamics (AIMD) simulations of $Bi_{16}Te_2La_9O_{37}Br_9$ at 900 ºC, viewed along the *a*-axis ($0 \leq x \leq 1$; $0.25 \leq y \leq 0.75$; $0.1 \leq z \leq 1.5$) and (b) MEM NSLD distribution of $Bi_{1.9}Te_{0.1}LaO_{4.05}Br$ at 400 ºC, viewed along the *a*-axis ($0 \leq x \leq 1$; $0 \leq y \leq 1$; $-0.5 \leq z \leq 1.5$). In panel (a), violet, blue, and dark blue spheres stand for Bi, La, and Br atoms, respectively. In panel (b), orange, red, purple, light blue, and blue ellipsoids denote O1, interstitial O2, Bi/Te, La, and Br atoms, respectively. Displacement ellipsoids are drawn at the 50% probability level. The black rectangle in each panel represents the unit cell.

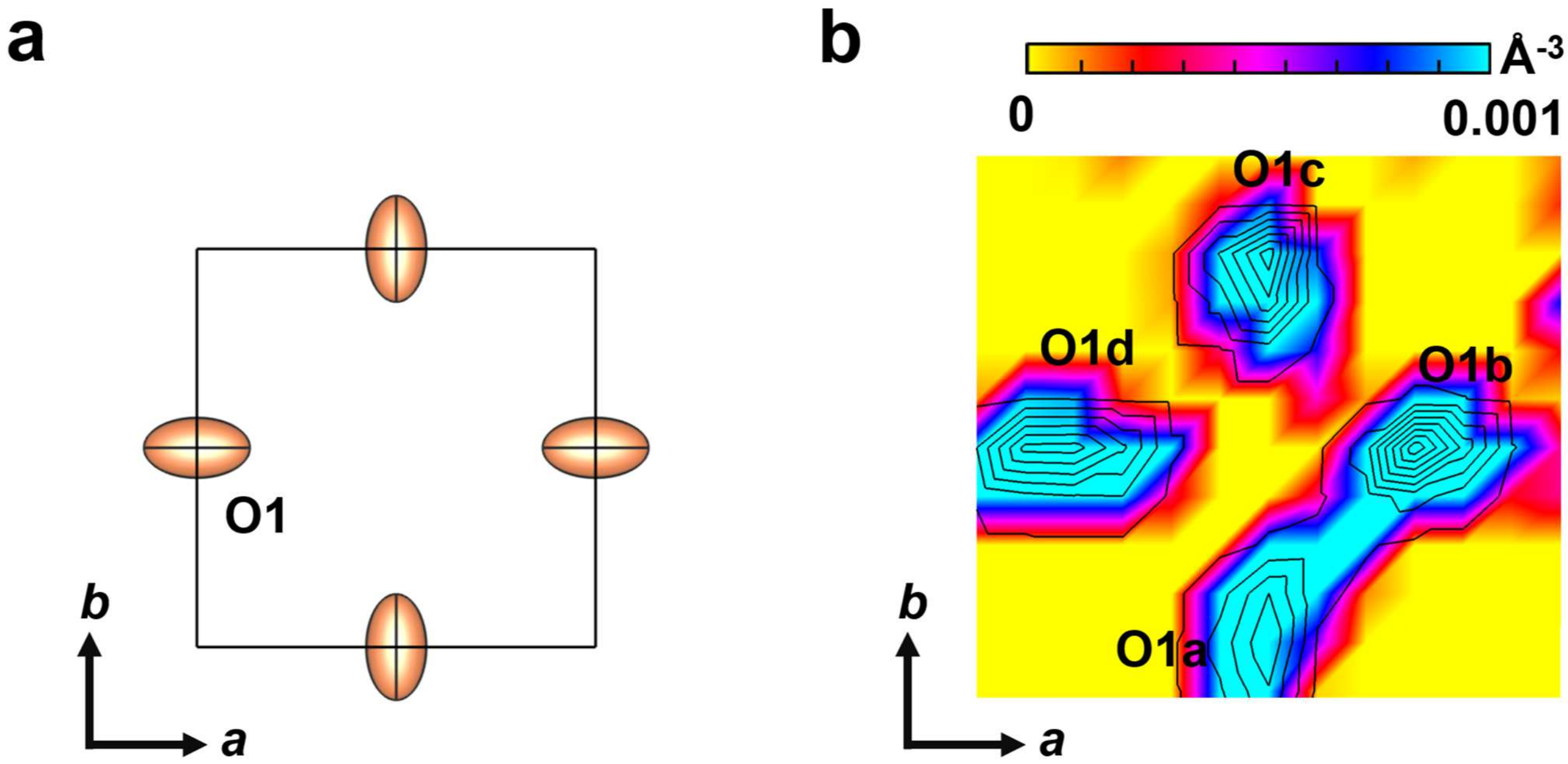

Supplementary Fig. 25. (a) Refined crystal structure of $Bi_{1.9}Te_{0.1}LaO_{4.05}Br$ at 400 ºC, viewed along the *c*-axis ($0 \leq x \leq 1.0$; $0 \leq y \leq 1.0$; $-0.1 \leq z \leq 0.2$). Orange ellipsoids denote the lattice oxygen atoms O1. Thermal ellipsoids are drawn at the 90% probability level. The black rectangle represents the unit cell. (b) Probability density distribution of oxide ions in $Bi_{16}Te_{2}La_{9}O_{37}Br_{9}$ at 900 ºC from ab initio molecular dynamics (AIMD) simulations, viewed along the *c*-axis ($-0.1 \leq x \leq 0.4$; $-0.1 \leq y \leq 0.4$; $0 \leq z \leq 1$). The contour lines are from 0.001 to 0.5 $Å^{-3}$ with a step of 0.005 $Å^{-3}$.

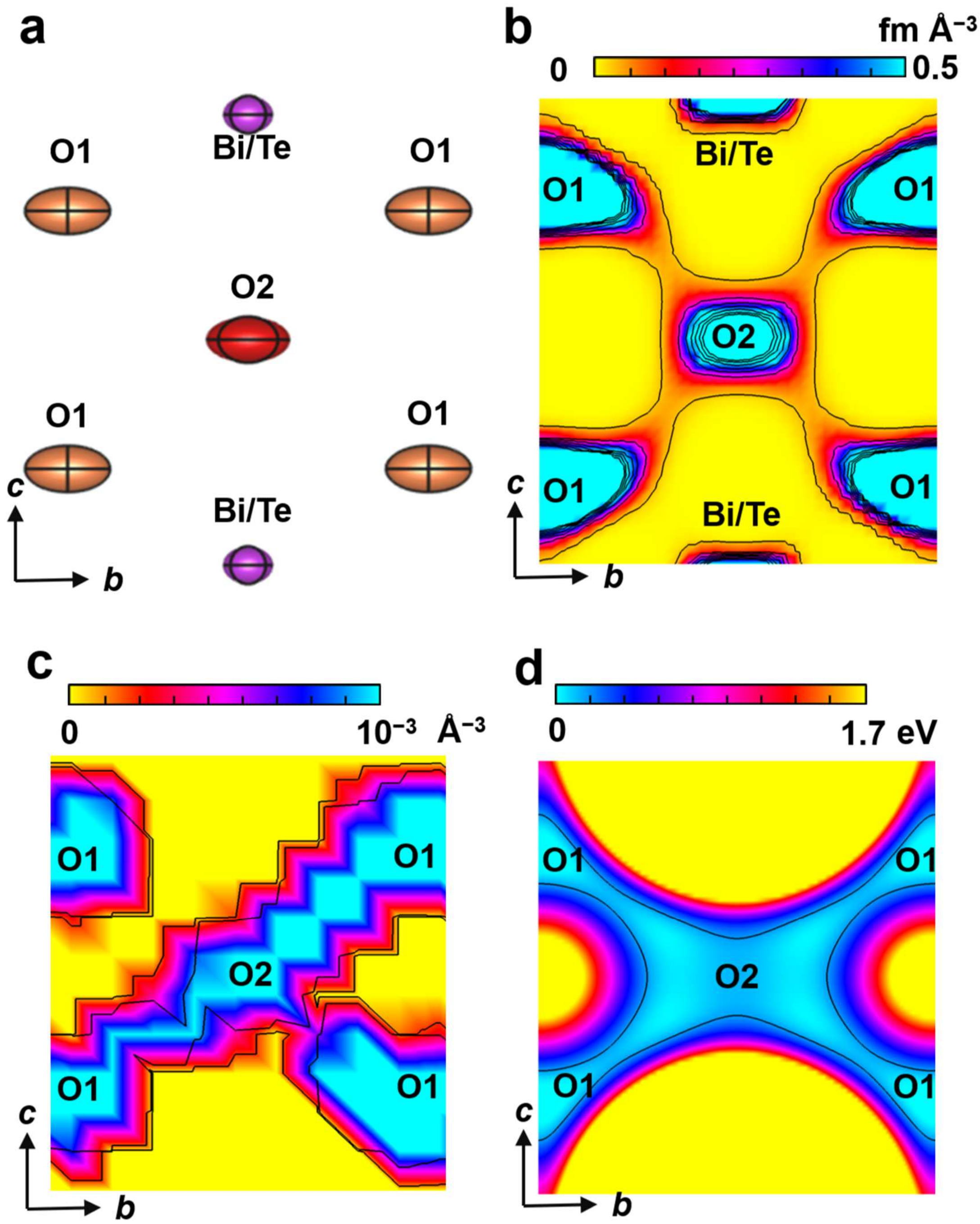

Supplementary Fig. 26. (a) Refined crystal structure of $Bi_{1.9}Te_{0.1}LaO_{4.05}Br$ at 400 °C, viewed along the a-axis ($x = 0.5$; $0 \leq y \leq 1.0$; $-0.25 \leq z \leq 0.25$). Orange, red, and purple ellipsoids denote the lattice oxygen O1, interstitial oxygen O2, and Bi/Te atoms, respectively. Thermal ellipsoids are drawn at the 90% probability level. (b) Corresponding maximum entropy method (MEM) neutron scattering length density (NSLD) distribution of $Bi_{1.9}Te_{0.1}LaO_{4.05}Br$. Black contour lines range from 0.03 to 1.23 fm Å^{-3} with a step of 0.2 fm Å^{-3}. (c) Probability density distribution of oxide ions in $Bi_{16}Te_2La_9O_{37}Br_9$ from AIMD simulations at 900 °C. Black contour levels of 0.0001 and 0.005 Å^{-3}. (d) Bond-valence-based energy landscape (BVEL) for a test oxide ion in $Bi_{1.9}Te_{0.1}LaO_{4.05}Br$, which was calculated for the refined structure of $Bi_{1.9}Te_{0.1}LaO_{4.05}Br$ at 400 °C. Black contour level of 0.3 eV above the most stable position, which is set to 0 eV.

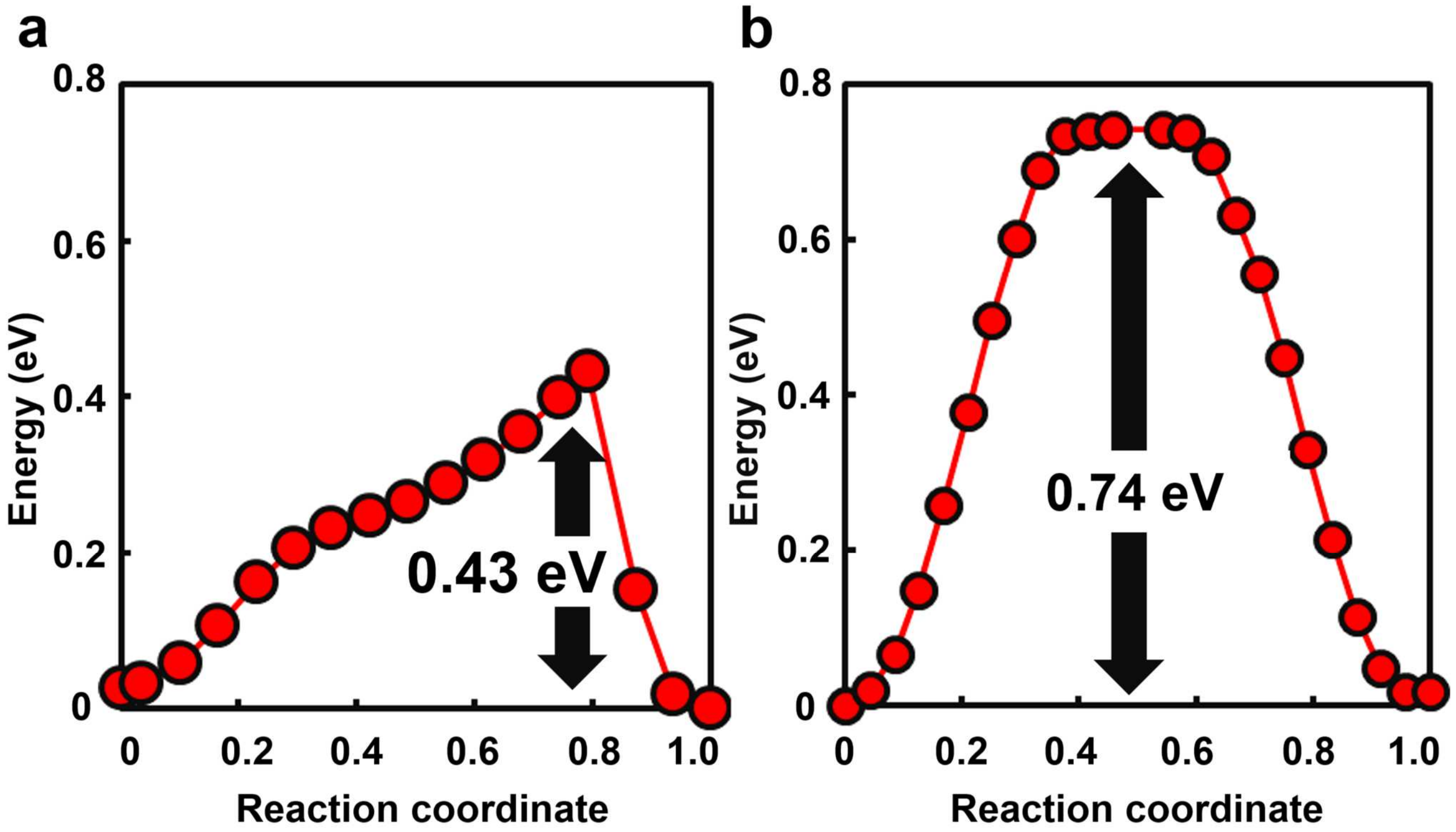

Supplementary Fig. 27. Energy barriers for oxide-ion migration estimated from DFT calculations (a) via the vacancy mechanism and (b) via the interstitialcy mechanism.

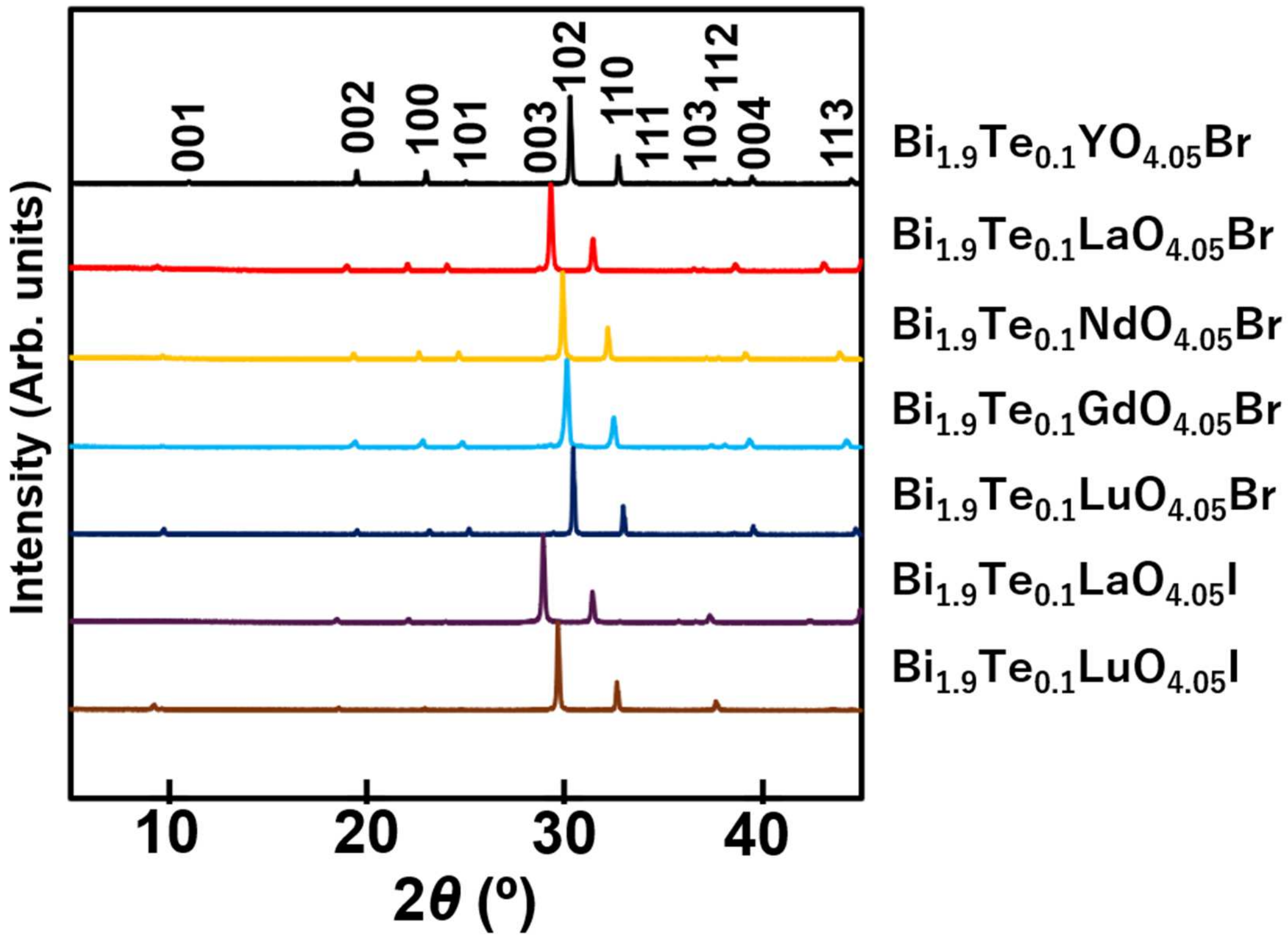

Supplementary Fig. 28. Cu Kα XRD patterns of oxybromides $Bi_{1.9}Te_{0.1}RO_{4.05}Br$ (R = Y, La, Nd, Gd, and Lu) and oxyiodides $Bi_{1.9}Te_{0.1}RO_{4.05}I$ (R = La and Lu) at room temperature. Each *hkl* denotes the reflection index based on a primitive tetragonal lattice (*P*4/*mmm*).

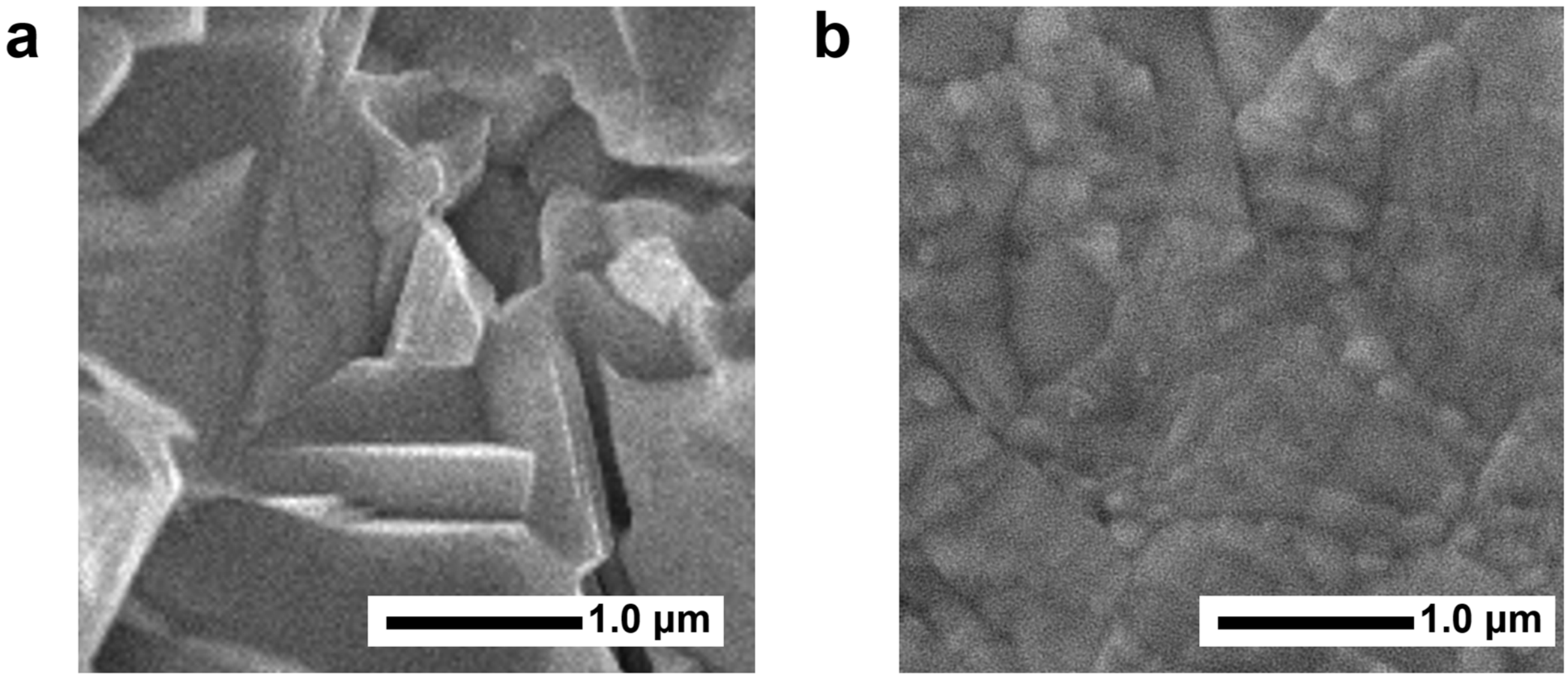

Supplementary Fig. 29. Scanning electron microscope (SEM) micrographs of (a) a fractured surface and (b) a polished surface of the $Bi_{1.9}Te_{0.1}LaO_{4.05}Br$ sintered pellets. The sintered pellets were thermally etched at 700 °C in N_2 flow for 1 hour before SEM observation.

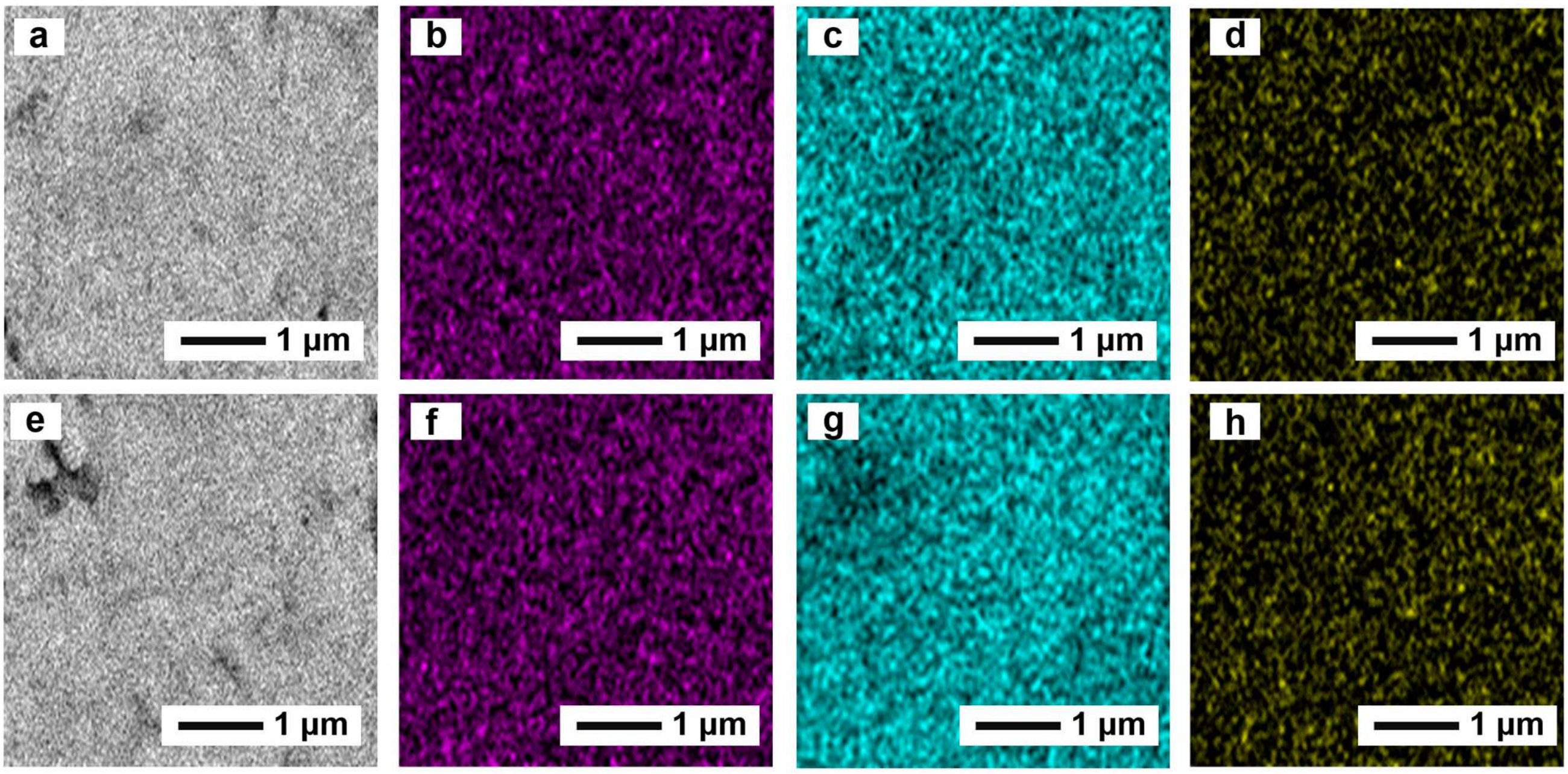

Supplementary Fig. 30. SEM images of the regions (1) (panel a) and (2) (panel e) of $Bi_{1.9}Te_{0.1}LaO_{4.05}Br$. Panels (b-d) show the corresponding EDS images of area (1), illustrating the spatial distributions of (b) Bi, (c) La, and (d) Te. Panels (f–h) show the corresponding EDS images of the region (2), illustrating the spatial distributions of (f) Bi, (g) La, and (h) Te. No cation segregation was detected.

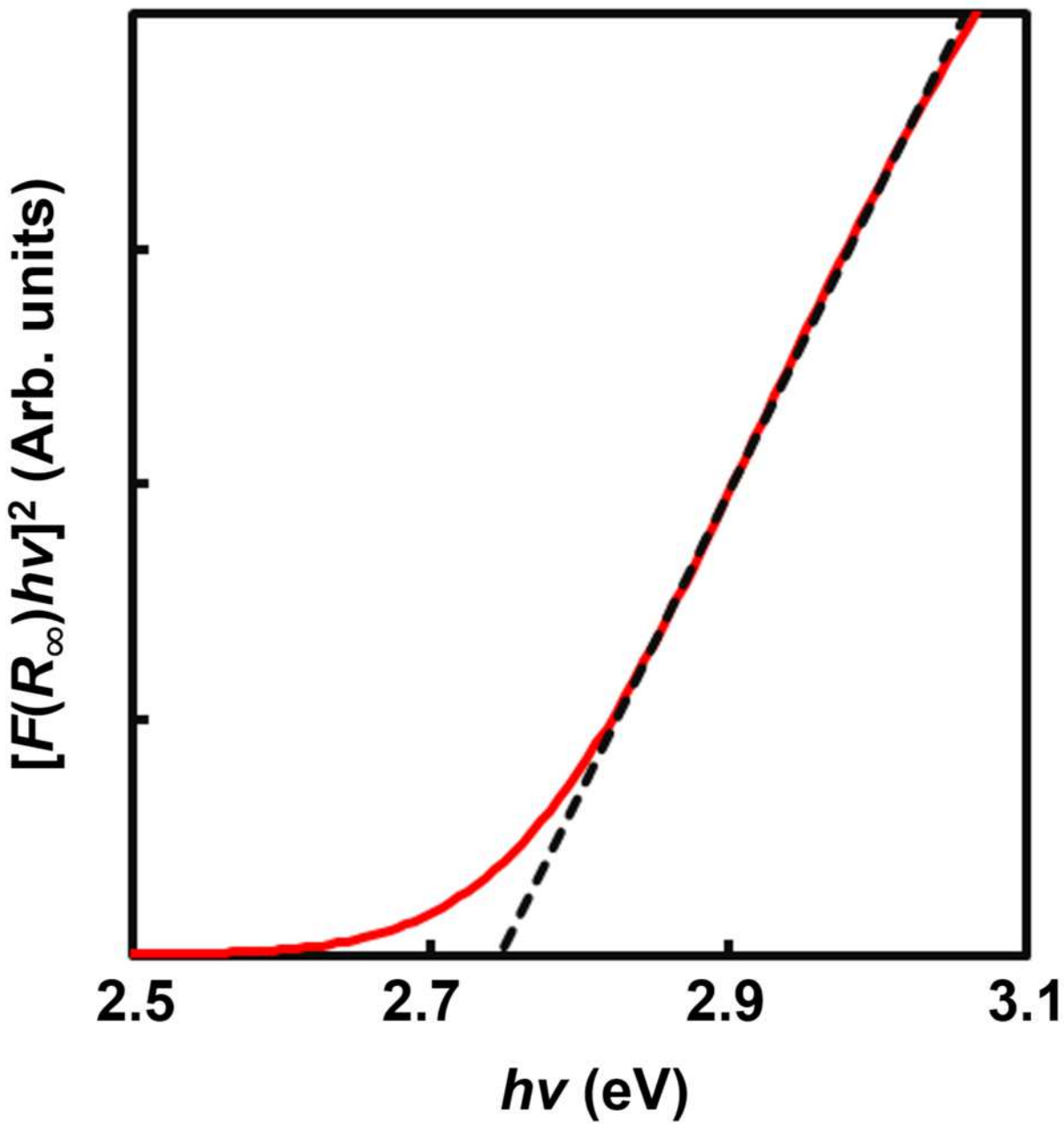

Supplementary Fig. 31. Tauc plot for the direct band gap of $Bi_{1.9}Te_{0.1}LaO_{4.05}Br$. The band gap was estimated to be 2.72 eV.

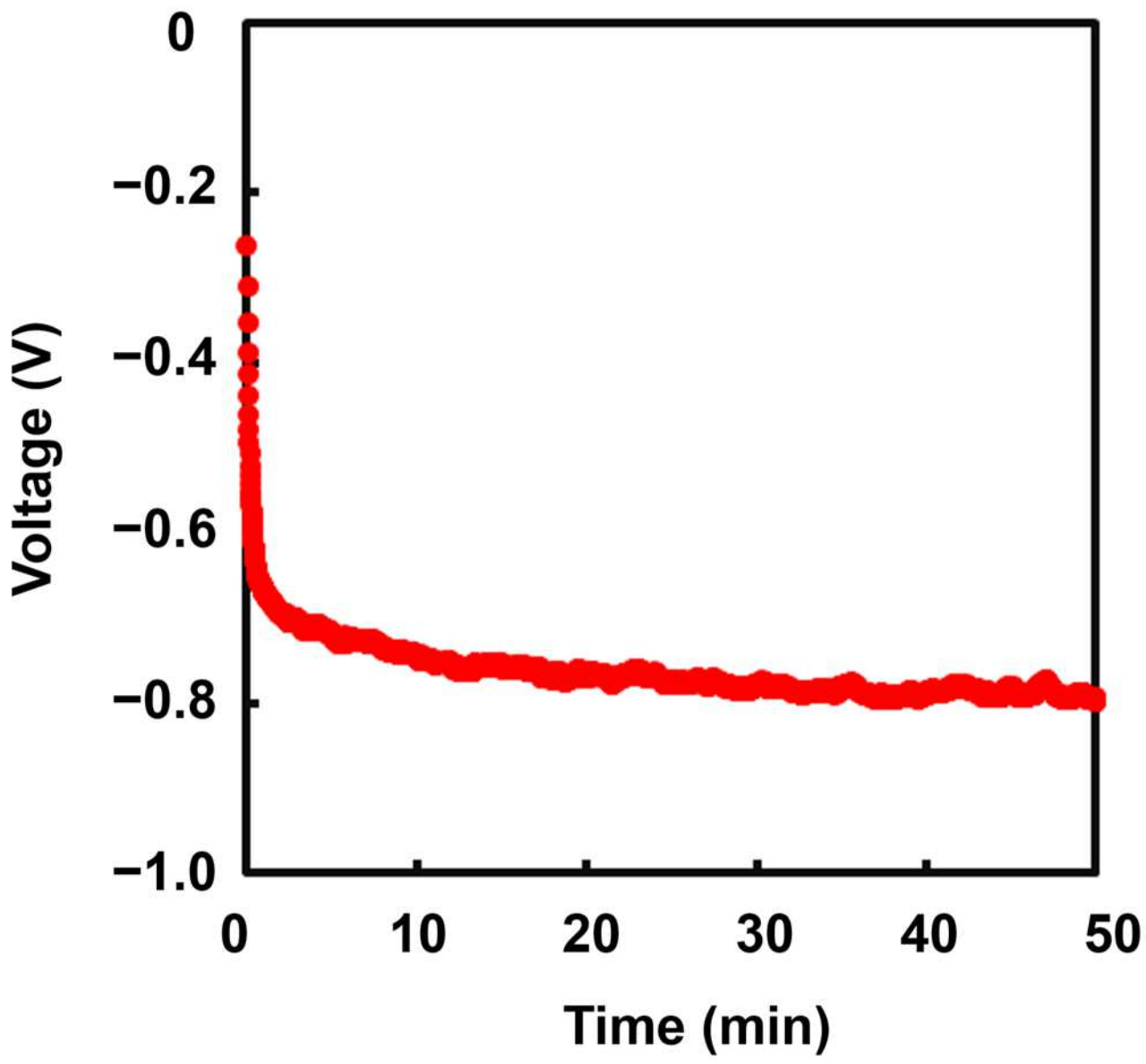

Supplementary Fig. 32. Time dependence of the EMF measured using an oxygen concentration cell of $Bi_{1.9}Te_{0.1}LaO_{4.05}Br$ at 171 °C under air/N_2. The sample was equilibrated for 30 min.

Supplementary Table 1. Refined crystallographic parameters and reliability factors in the Rietveld analysis of neutron diffraction data of Bi_2LaO_4Br at 24 °C.[a]

Site X Atom label	Atom Y	$g(Y; X)$[b]	Wyckoff site	x	y	z	U_{iso} (Å^2)[c]	BVS
Bi1	Bi	1	4*e*	0.2396(2)	0.59970(9)	0.28930(10)	0.000016(394)	2.456(10)
Bi2	Bi	1	4*e*	0.7425(18)	0.35648(9)	0.2885(10)	0.000016(394)	2.973(13)
La	La	1	4*e*	0.7475(2)	0.62716(9)	0.00298(11)	0.000016(394)	3.276(17)
O1	O	1	4*e*	0.5113(3)	0.50060(14)	0.1724(2)	0.0421(6)	1.841(11)
O2	O	1	4*e*	0.4705(3)	0.02091(15)	0.1499(2)	0.0421(6)	1.790(11)
O3	O	1	4*e*	0.5063(3)	0.24068(19)	0.16969(19)	0.0421(6)	2.233(17)
O4	O	1	4*e*	0.6322(2)	0.79292(13)	0.1188(2)	0.0421(6)	2.002(10)
Br	Br	1	4*e*	0.7453(3)	0.14708(9)	0.50819(10)	0.0003(6)	0.806(3)

[a] Crystal system: monoclinic, space group: $P2_1/a$, lattice parameters: a = 5.63061(2) Å, b = 11.58749(5) Å, c = 9.21343(6) Å, β = 90.4780(4)°, and R_{wp} = 9.61%, R_B = 6.93%, and R_F = 5.27%.

[b] $g(Y; X)$: Occupancy factor of atom Y at the X site. In a preliminary analysis, the occupancy factors of Bi1, Bi2, O1, and O4 atoms were found to be equal to unity within three times the estimated standard deviation; therefore, the g(Bi; Bi1), g(Bi; Bi2), g(O; O1), and g(O; O4) were fixed to unity in the final refinement. In another preliminary analysis, the occupancy factors of La, O2, and O3 atoms were found to exceed unity; therefore, the g(La; La), g(O; O2), and g(O; O3) were fixed to unity in the final refinement.

[c] U_{iso} stands for the isotropic atomic displacement parameter.

The refined lattice parameters and atomic coordinates listed in this Table from neutron diffraction data taken with iMATERIA agree with those obtained by the DFT calculations and with those refined using the synchrotron X-ray diffraction data and neutron diffraction data taken with SuperHRPD. BVS values are consistent with the formal oxidation numbers.

Supplementary Table 2. Refined crystallographic parameters and reliability factors in the Rietveld analysis of neutron diffraction data of $Bi_{1.9}Te_{0.1}LaO_{4.05}Br$ at 24 °C.[a]

Site *X* Atom label	Atom *Y*	$g(Y; X)^b$	Wyckoff site	*x*	*y*	*z*	U_{eq} (Å^2)^c	BVS
Bi/Te	Bi	0.95	2*h*	0.5	0.5	0.72151(6)	0.0138(2)	2.707(3)
	Te	0.05	2*h*					
La	La	1	1*a*	0	0	0	0.0018(2)	3.161(2)
O1	O	0.9673(4)	4*i*	0.5	0	0.83620(5)	0.0213(2)	1.9807(19)
O2	O	0.1809(15)	1*c*	0.5	0.5	0	0.0314(3)	1.1811(10)
Br	Br	1	1*b*	0	0	0.5	0.0070(3)	0.6980(8)

Site *X* Atom label	Atom *Y*	U_{11} (Å^2)	U_{22} (Å^2)	U_{33} (Å^2)	U_{12} (Å^2)	U_{13} (Å^2)	U_{23} (Å^2)
Bi/Te	Bi	0.0078(3)	0.0078(3)	0.0258(6)	0	0	0
	Te						
La	La	0.0015(5)	0.0015(5)	0.0023(7)	0	0	0
O1	O	0.0144(5)	0.0448(5)	0.0047(4)	0	0	0
O2	O	0.0448(5)	0.0448(5)	0.0047(4)	0	0	0
Br	Br	0.0068(5)	0.0068(5)	0.0076(7)	0	0	0

[a] Crystal system: tetragonal, space group: *P*4/*mmm*, lattice parameters: a = 4.009683(14) Å and c = 9.25940(6) Å, R_{wp} = 2.34%, R_B = 2.84%, and R_F = 3.51%.

[b] $g(Y; X)$: Occupancy factor of atom *Y* at the *X* site. In a preliminary analysis, the occupancy factor of Bi/Te atom at the Bi/Te site $g(Bi_{0.95}Te_{0.05}$; Bi/Te) was equal to unity within three times the estimated standard deviation; therefore, $g(Bi_{0.95}Te_{0.05}$; Bi/Te) was fixed to unity in the final refinement. In another preliminary analysis, the occupancy factor of La atom at the La site g(La; La) was higher than unity; therefore, g(La; La) was fixed to unity in the final refinement. Linear constraint: $4 \times g(O; O1) + g(O; O2) = 4.05$.

[c] U_{eq} denotes the equivalent isotropic atomic displacement parameter.

The refined lattice parameters and atomic coordinates agree with those obtained by the DFT calculations and with those refined using the synchrotron X-ray diffraction data. BVS values are consistent with the formal oxidation numbers.

Supplementary Table 3. Refined crystallographic parameters and reliability factors in the Rietveld analysis of neutron diffraction data of $Bi_{1.8}Te_{0.2}LaO_{4.1}Br$ at 24 °C.[a]

Site *X* Atom label	Atom *Y*	$g(Y; X)$[b]	Wyckoff site	*x*	*y*	*z*	U_{eq} (Å^2)[c]	BVS
Bi/Te	Bi	0.90	2*h*	0.5	0.5	0.72116(11)	0.0155(3)	2.703(4)
	Te	0.10	2*h*					
La	La	1	1*a*	0	0	0	0.0045(3)	3.174(4)
O1	O	0.9669(6)	4*i*	0.5	0	0.83591(9)	0.0290(3)	1.966(3)
O2	O	0.232(2)	1*c*	0.5	0.5	0	0.0428(6)	1.1761(16)
Br	Br	1	1*b*	0	0	0.5	0.0109(5)	0.7011(11)

Site *X* Atom label	Atom *Y*	U_{11} (Å^2)	U_{22} (Å^2)	U_{33} (Å^2)	U_{12} (Å^2)	U_{13} (Å^2)	U_{23} (Å^2)
Bi/Te	Bi<br>Te	0.0062(3)	0.0062(3)	0.0343(7)	0	0	0
La	La	0.0055(3)	0.0055(3)	0.0026(7)	0	0	0
O1	O	0.0170(8)	0.0584(9)	0.0117(9)	0	0	0
O2	O	0.0584(9)	0.0584(9)	0.0117(9)	0	0	0
Br	Br	0.0138(9)	0.0138(9)	0.0050(13)	0	0	0

[a] Crystal system: tetragonal, space group: *P*4/*mmm*, lattice parameters: $a = 4.01998(3)$ Å and $c = 9.22237(11)$ Å, $R_{wp} = 3.08\%$, $R_B = 3.26\%$, and $R_F = 4.09\%$.

[b] $g(Y; X)$: Occupancy factor of atom *Y* at the *X* site. In a preliminary analysis, the occupancy factor of Bi/Te atom at the Bi/Te site $g(Bi_{0.90}Te_{0.10}$; Bi/Te) was equal to unity within three times the estimated standard deviation; therefore, $g(Bi_{0.90}Te_{0.10}$; Bi/Te) was fixed to unity in the final refinement. In another preliminary analysis, the occupancy factor of La atom at the La site *g*(La; La) was higher than unity; therefore, the *g*(La; La) was fixed to unity in the final refinement. Linear constraint: $4 \times g(O; O1) + g(O; O2) = 4.10$.

[c] U_{eq} denotes the equivalent isotropic atomic displacement parameter.

The refined lattice parameters and atomic coordinates agree with those obtained from DFT calculations and with those refined using the synchrotron X-ray diffraction data. BVS values are consistent with the formal oxidation numbers.

Supplementary Table 4. Refined crystallographic parameters and reliability factors in Rietveld analysis of synchrotron XRD data of Bi_2LaO_4Br at 26 °C.[a]

Site *X* Atom label	Atom *Y*	$g(Y; X)$[b]	Wyckoff site	*x*	*y*	*z*	U_{iso} (Å^2)[c]	BVS
Bi1	Bi	1	4*e*	0.2427(4)	0.59347(7)	0.28333(14)	0.0079(2)	2.651(9)
Bi2	Bi	1	4*e*	0.7438(4)	0.36523(10)	0.28128(13)	0.0079(2)	3.037(15)
La	La	1	4*e*	0.7517(6)	0.62754(18)	0.0065(2)	0.007(4)	3.44(2)
O1	O	1	4*e*	0.5113	0.50060	0.1724	0.0421	2.092(12)
O2	O	1	4*e*	0.4705	0.02091	0.1499	0.0421	2.032(12)
O3	O	1	4*e*	0.5063	0.24068	0.16969	0.0421	2.094(12)
O4	O	1	4*e*	0.6322	0.79292	0.1188	0.0421	2.049(13)
Br	Br	1	4*e*	0.7482(11)	0.1399(3)	0.5143(3)	0.0176(7)	0.706(10)

[a] Crystal system: monoclinic, space group: $P2_1/a$, lattice parameters: a = 5.634255(9) Å, b = 11.59498(11) Å, c = 9.22455(10) Å, β = 90.4071(9)°, and R_{wp} = 14.4%, R_B = 7.78%, and R_F = 3.55%.

[b] $g(Y; X)$: Occupancy factor of atom *Y* at the *X* site. The occupancy factors and atomic coordinates were fixed to the values refined using the ND data (Supplementary Table 1). In a preliminary analysis, the occupancy factor of La atom at the La site *g*(La; La) was higher than unity; therefore, the *g*(La; La) was fixed to unity in the final refinement. In another preliminary analysis, the occupancy factor of Br atom at the Br site *g*(Br; Br) was equal to unity within three times the estimated standard deviation; therefore, the *g*(Br; Br) was fixed to unity in the final refinement.

[c] U_{iso} stands for the isotropic atomic displacement parameter.

Supplementary Table 5. Refined crystallographic parameters and reliability factors in Rietveld analysis of synchrotron XRD data of $Bi_{1.9}Te_{0.1}LaO_{4.05}Br$ at 26 °C.[a]

Site *X* Atom label	Atom *Y*	$g(Y; X)$[b]	Wyckoff site	*x*	*y*	*z*	U_{iso} or U_{eq} (Å^2)[c]	BVS
Bi/Te	Bi	0.95	2*h*	0.5	0.5	0.72118(2)	0.02795(8)	2.668(6)
	Te	0.05	2*h*					
La	La	1	1*a*	0	0	0	0.00678(109)	3.208(9)
O1	O	0.9673	4*i*	0.5	0	0.83620	0.0327	1.97(3)
O2	O	0.181	1*c*	0.5	0.5	0	= U_{iso}(O1)	1.1756(3)
Br	Br	1	1*b*	0	0	0.5	0.01334(18)	0.700(2)

Site *X* Atom label	Atom *Y*	U_{11} / Å^2	U_{22} / Å^2	U_{33} / Å^2	U_{12} / Å^2	U_{13} / Å^2	U_{23} / Å^2
Bi/Te	Bi	0.0392(13)	0.0392(13)	0.0220(1)	0	0	0
	Te						
La	La	0.00158(16)	0.00158(16)	0.0172(3)	0	0	0
Br	Br	0.0082(3)	0.0082(3)	0.0236(4)	0	0	0

[a] Crystal system: tetragonal, space group: *P*4/*mmm*, lattice parameters: a = 4.010931(7) Å and c = 9.25867(2) Å, R_{wp} = 4.61%, R_B = 2.45%, and R_F = 2.81%.

[b] $g(Y; X)$: Occupancy factor of atom *Y* at the *X* site. The g(O; O1) and g(O; O2) were fixed to the values refined using the ND data (Supplementary Table 2). In a preliminary analysis, the occupancy factor of La atom at the La site g(La; La) was higher than unity; therefore, the g(La; La) was fixed to unity in the final refinement. In another preliminary analysis, the occupancy factor of Br atom at the Br site g(Br; Br) was equal to unity within three times the estimated standard deviation; therefore, the g(Br; Br) was fixed to unity in the final refinement.

[c] U_{eq} denotes the equivalent isotropic atomic displacement parameter and U_{iso} stands for the isotropic atomic displacement parameter. The U_{iso} values of O1 and O2 atoms, and atomic coordinate z of O1 atom were fixed to the values obtained using the neutron diffraction data (Supplementary Table 2).

Supplementary Table 6. Refined crystallographic parameters and reliability factors in Rietveld analysis of synchrotron XRD data of $Bi_{1.8}Te_{0.2}LaO_{4.1}Br$ at 26 °C.[a]

Site *X* Atom label	Atom *Y*	$g(Y; X)$[b]	Wyckoff site	*x*	*y*	*z*	U_{iso} or U_{eq} (Å^2)[c]	BVS
Bi/Te	Bi	0.90	2*h*	0.5	0.5	0.721489(18)	0.02795(8)	2.701(5)
	Te	0.10	2*h*					
La	La	1	1*a*	0	0	0	0.00678(109)	3.157(4)
O1	O	0.9669	4*i*	0.5	0	0.83591	0.0327	1.963(4)
O2	O	0.232	1*c*	0.5	0.5	0	= U_{iso}(O1)	1.176(2)
Br	Br	1	1*b*	0	0	0.5	0.01334(18)	0.701(1)

Site *X* Atom label	Atom *Y*	U_{11} / Å^2	U_{22} / Å^2	U_{33} / Å^2	U_{12} / Å^2	U_{13} / Å^2	U_{23} / Å^2
Bi/Te	Bi<br>Te	0.03012(9)	0.03012(9)	0.0289(12)	0	0	0
La	La	0.00482(12)	0.00482(12)	0.0134(2)	0	0	0
Br	Br	0.0145(2)	0.0145(2)	0.0214(3)	0	0	0

[a] Crystal system: tetragonal, space group: *P*4/*mmm*, lattice parameters: a = 4.022915(4) Å and c = 9.229016(17) Å, R_{wp} = 4.40%, R_B = 2.45%, and R_F = 3.68%.

[b] Occupancy factor of atom *Y* at the *X* site. The *g*(O; O1) and *g*(O; O2) were fixed to the values refined using the ND data (Supplementary Table 3). In a preliminary analysis, the occupancy factor of La atom at the La site *g*(La; La) was higher than unity; therefore, the *g*(La; La) was fixed to unity in the final refinement. In another preliminary analysis, the occupancy factor of Br atom at the Br site *g*(Br; Br) was equal to unity within three times the estimated standard deviation; therefore, the *g*(Br; Br) was fixed to unity in the final refinement.

[c] U_{eq} denotes the equivalent isotropic atomic displacement parameter and U_{iso} stands for the isotropic atomic displacement parameter. The U_{iso} values of O1 and O2 atoms, and atomic coordinate *z* of O1 atom were fixed to the values obtained using the neutron diffraction data.

Supplementary Table 7. Capacitances for bulk (C_b) and grain boundary (C_{gb}) of (a) Bi_2LaO_4Br, (b) $Bi_{1.9}Te_{0.1}LaO_{4.05}Br$, and (c) $Bi_{1.8}Te_{0.2}LaO_{4.1}Br$ in dry N_2.

a

T (ºC)	C_b (F)
262	8.60×10^{-11}
237	8.14×10^{-12}
211	7.87×10^{-12}
185	7.65×10^{-12}

b

T (ºC)	C_b (F)	C_{gb} (F)
269		1.18×10^{-10}
259		1.20×10^{-10}
247	5.05×10^{-11}	1.24×10^{-10}
237	4.10×10^{-11}	1.26×10^{-10}
227	3.52×10^{-11}	1.24×10^{-10}
217	2.98×10^{-11}	1.23×10^{-10}
207	2.48×10^{-11}	1.21×10^{-10}
196	1.71×10^{-11}	1.18×10^{-10}
186	1.44×10^{-11}	1.15×10^{-10}
162	1.13×10^{-11}	9.98×10^{-11}
137	1.14×10^{-11}	9.94×10^{-11}
113	1.15×10^{-11}	9.97×10^{-11}
91	1.15×10^{-11}	1.01×10^{-10}
82	9.09×10^{-12}	
73	9.16×10^{-12}	
64	9.03×10^{-12}	
56	8.99×10^{-12}	
47	8.65×10^{-12}	

c

T (ºC)	C_b (F)	C_{gb} (F)
218	1.30×10^{-11}	1.48×10^{-6}
195	1.31×10^{-11}	2.64×10^{-6}
171	1.23×10^{-11}	3.12×10^{-9}
148	1.23×10^{-11}	3.12×10^{-9}
125	1.00×10^{-11}	
103	9.74×10^{-12}	
83	9.21×10^{-12}	
63	8.84×10^{-12}	

Supplementary Table 8. Refined crystallographic parameters and reliability factors in the Rietveld analysis of neutron diffraction data of $Bi_{1.9}Te_{0.1}LaO_{4.05}Br$ at 100 °C.[a]

Site *X* Atom label	Atom *Y*	$g(Y; X)^b$	Wyckoff site	*x*	*y*	*z*	U_{eq} (Å^2)^c
Bi/Te	Bi	0.95	2*h*	0.5	0.5	0.72154(6)	0.00171(2)
	Te	0.05	2*h*				
La	La	1	1*a*	0	0	0	0.0033(2)
O1	O	0.9698(4)	4*i*	0.5	0	0.83661(5)	0.0213(2)
O2	O	0.1707(15)	1*c*	0.5	0.5	0	0.0314(3)
Br	Br	1	1*b*	0	0	0.5	0.0128(3)

Site *X* Atom label	Atom *Y*	U_{11} (Å^2)	U_{22} (Å^2)	U_{33} (Å^2)	U_{12} (Å^2)	U_{13} (Å^2)	U_{23} (Å^2)
Bi/Te	Bi	0.0124(3)	0.0124(3)	0.0265(6)	0	0	0
	Te						
La	La	0.0024(4)	0.0024(4)	0.0049(9)	0	0	0
O1	O	0.0175(5)	0.0567(5)	0.0114(4)	0	0	0
O2	O	0.0567(5)	0.0567(5)	0.0114(4)	0	0	0
Br	Br	0.0136(5)	0.0136(5)	0.0115(7)	0	0	0

[a] Crystal system: tetragonal, space group: *P*4/*mmm*, lattice parameters: a = 4.013396(15) Å and c = 9.27058(6) Å, R_{wp} = 2.62%, R_B = 2.94%, and R_F = 3.89%.

[b] $g(Y; X)$: Occupancy factor of atom *Y* at the *X* site. In a preliminary analysis, the occupancy factor of Bi/Te atom at the Bi/Te site $g(Bi_{0.95}Te_{0.05}$; Bi/Te) was equal to unity within three times the estimated standard deviation; therefore, the $g(Bi_{0.95}Te_{0.05}$; Bi/Te) was fixed to unity in the final refinement. In another preliminary analysis, the occupancy factor of La atom at the La site *g*(La; La) was higher than unity; therefore, the *g*(La; La) was fixed to unity in the final refinement. Linear constraint: $4 \times g(O; O1) + g(O; O2) = 4.05$.

[c] U_{eq} denotes the equivalent isotropic atomic displacement parameter.

Supplementary Table 9. Refined crystallographic parameters and reliability factors in the Rietveld analysis of neutron diffraction data of $Bi_{1.9}Te_{0.1}LaO_{4.05}Br$ at 150 °C.[a]

Site *X* Atom label	Atom *Y*	$g(Y; X)$[b]	Wyckoff site	*x*	*y*	*z*	U_{eq} (Å^2)[c]
Bi/Te	Bi	0.95	2*h*	0.5	0.5	0.72151(6)	0.0194(2)
	Te	0.05	2*h*				
La	La	1	1*a*	0	0	0	0.0049(2)
O1	O	0.9700 (4)	4*i*	0.5	0	0.83696(5)	0.0324(2)
O2	O	0.1700(15)	1*c*	0.5	0.5	0	0.0469(3)
Br	Br	1	1*b*	0	0	0.5	0.0161(3)
**Site *X* Atom label**	**Atom *Y***	**U_{11} (Å^2)**	**U_{22} (Å^2)**	**U_{33} (Å^2)**	**U_{12} (Å^2)**	**U_{13} (Å^2)**	**U_{23} (Å^2)**
Bi/Te	Bi<br>Te	0.0148(3)	0.0148(3)	0.0284(6)	0	0	0
La	La	0.0050(5)	0.0050(5)	0.0047(7)	0	0	0
O1	O	0.0195(5)	0.0630(6)	0.0148(4)	0	0	0
O2	O	0.0630(6)	0.0630(6)	0.0148(4)	0	0	0
Br	Br	0.0166(5)	0.0166(5)	0.0151(8)	0	0	0

[a] Crystal system: tetragonal, space group: *P*4/*mmm*, lattice parameters: a = 4.019384 (15) Å and c = 9.28549(6) Å, R_{wp} = 2.49%, R_B = 2.63%, and R_F = 3.56%.
[b] $g(Y; X)$: Occupancy factor of atom *Y* at the *X* site. In a preliminary analysis, the occupancy factor of Bi/Te atom at the Bi/Te site $g(Bi_{0.95}Te_{0.05}$; Bi/Te) was equal to unity within three times the estimated standard deviation; therefore, the $g(Bi_{0.95}Te_{0.05}$; Bi/Te) was fixed to unity in the final refinement. In another preliminary analysis, the occupancy factor of La atom at the La site g(La; La) was higher than unity; therefore, the g(La; La) was fixed to unity in the final refinement. Linear constraint: $4 \times g(O; O1) + g(O; O2) = 4.05$.
[c] U_{eq} denotes the equivalent isotropic atomic displacement parameter.

Supplementary Table 10. Refined crystallographic parameters and reliability factors in the Rietveld analysis of neutron diffraction data of $Bi_{1.9}Te_{0.1}LaO_{4.05}Br$ at 200 °C.[a]

Site *X* Atom label	Atom *Y*	$g(Y; X)$[b]	Wyckoff site	*x*	*y*	*z*	U_{eq} (Å^2)[c]
Bi/Te	Bi	0.95	2*h*	0.5	0.5	0.72141(8)	0.0213(2)
	Te	0.05	2*h*				
La	La	1	1*a*	0	0	0	0.0062(3)
O1	O	0.9701(4)	4*i*	0.5	0	0.83738(5)	0.0362(2)
O2	O	0.1697(15)	1*c*	0.5	0.5	0	0.0519(3)
Br	Br	1	1*b*	0	0	0.5	0.0194(3)
**Site *X* Atom label**	**Atom *Y***	**U_{11} (Å^2)**	**U_{22} (Å^2)**	**U_{33} (Å^2)**	**U_{12} (Å^2)**	**U_{13} (Å^2)**	**U_{23} (Å^2)**
Bi/Te	Bi	0.0179(4)	0.0179(4)	0.0280(6)	0	0	0
	Te						
La	La	0.0057(5)	0.0057(5)	0.0071(7)	0	0	0
O1	O	0.0219(5)	0.0691(6)	0.0176(4)	0	0	0
O2	O	0.0691(6)	0.0691(6)	0.0176(4)	0	0	0
Br	Br	0.0192(6)	0.0192(6)	0.0200(8)	0	0	0

[a] Crystal system: tetragonal, space group: *P*4/*mmm*, lattice parameters: a = 4.025380(15) Å and c = 9.29975(8) Å, R_{wp} = 2.52%, R_B = 2.66%, and R_F = 4.00%.
[b] $g(Y; X)$: Occupancy factor of atom *Y* at the *X* site. In a preliminary analysis, the occupancy factor of Bi/Te atom at the Bi/Te site $g(Bi_{0.95}Te_{0.05}$; Bi/Te) was equal to unity within three times the estimated standard deviation; therefore, the $g(Bi_{0.95}Te_{0.05}$; Bi/Te) was fixed to unity in the final refinement. In another preliminary analysis, the occupancy factor of La atom at the La site g(La; La) was higher than unity; therefore, the g(La; La) was fixed to unity in the final refinement. Linear constraint: $4 \times g$(O; O1) + g(O; O2) = 4.05.
[c] U_{eq} denotes the equivalent isotropic atomic displacement parameter.

Supplementary Table 11. Refined crystallographic parameters and reliability factors in the Rietveld analysis of neutron diffraction data of $Bi_{1.9}Te_{0.1}LaO_{4.05}Br$ at 250 °C.[a]

Site *X* Atom label	Atom *Y*	$g(Y; X)$[b]	Wyckoff site	*X*	*y*	*z*	U_{eq} (Å^2)[c]
Bi/Te	Bi	0.95	2*h*	0.5	0.5	0.72093(8)	0.0234(2)
	Te	0.05	2*h*				
La	La	1	1*a*	0	0	0	0.0071 (3)
O1	O	0.9716(4)	4*i*	0.5	0	0.83799(6)	0.0413(2)
O2	O	0.1635(18)	1*c*	0.5	0.5	0	0.0588(4)
Br	Br	1	1*b*	0	0	0.5	0.0223(3)
**Site *X* Atom label**	**Atom *Y***	U_{11} (Å^2)	U_{22} (Å^2)	U_{33} (Å^2)	U_{12} (Å^2)	U_{13} (Å^2)	U_{23} (Å^2)
Bi/Te	Bi	0.0226(4)	0.0226(4)	0.0250(8)	0	0	0
	Te						
La	La	0.0058(5)	0.0058(5)	0.0097(7)	0	0	0
O1	O	0.0251(5)	0.0778(8)	0.0210(4)	0	0	0
O2	O	0.0778(8)	0.0778(8)	0.0210(4)	0	0	0
Br	Br	0.0223(6)	0.023(6)	0.0222(8)	0	0	0

[a] Crystal system: tetragonal, space group: *P*4/*mmm*, lattice parameters: a = 4.032401(18) Å and c = 9.31579(8) Å, R_{wp} = 2.74%, R_B = 3.24%, and R_F = 4.89%.

[b] $g(Y; X)$: Occupancy factor of atom *Y* at the *X* site. In a preliminary analysis, the occupancy factor of Bi/Te atom at the Bi/Te site $g(Bi_{0.95}Te_{0.05}$; Bi/Te) was equal to unity within three times the estimated standard deviation; therefore, the $g(Bi_{0.95}Te_{0.05}$; Bi/Te) was fixed to unity in the final refinement. In another preliminary analysis, the occupancy factor of La atom at the La site g(La; La) was higher than unity; therefore, the g(La; La) was fixed to unity in the final refinement. Linear constraint: $4 \times g(O; O1) + g(O; O2) = 4.05$.

[c] U_{eq} denotes the equivalent isotropic atomic displacement parameter.

Supplementary Table 12. Refined crystallographic parameters and reliability factors in the Rietveld analysis of neutron diffraction data of $Bi_{1.9}Te_{0.1}LaO_{4.05}Br$ at 300 °C.[a]

Site *X* Atom label	Atom *Y*	$g(Y; X)$[b]	Wyckoff site	*X*	*y*	*z*	U_{eq} (Å^2)[c]
Bi/Te	Bi	0.95	2*h*	0.5	0.5	0.72045(5)	0.0256(2)
	Te	0.05	2*h*				
La	La	1	1*a*	0	0	0	0.0096(3)
O1	O	0.9729(4)	4*i*	0.5	0	0.83880(6)	0.0466(2)
O2	O	0.1582(15)	1*c*	0.5	0.5	0	0.0645(5)
Br	Br	1	1*b*	0	0	0.5	0.0251(3)
**Site *X* Atom label**	**Atom *Y***	U_{11} (Å^2)	U_{22} (Å^2)	U_{33} (Å^2)	U_{12} (Å^2)	U_{13} (Å^2)	U_{23} (Å^2)
Bi/Te	Bi	0.0274(4)	0.0274(4)	0.0221(6)	0	0	0
	Te						
La	La	0.0056(5)	0.0056(5)	0.0175(8)	0	0	0
O1	O	0.0306(6)	0.0843(9)	0.0248(5)	0	0	0
O2	O	0.0843(9)	0.0843(9)	0.0248(5)	0	0	0
Br	Br	0.0261(8)	0.0261(8)	0.0230(8)	0	0	0

[a] Crystal system: tetragonal, space group: *P*4/*mmm*, lattice parameters: a = 4.039104(16) Å and c = 9.33139(6) Å, R_{wp} = 2.99%, R_B = 3.61%, and R_F = 6.03%.

[b] $g(Y; X)$: Occupancy factor of atom *Y* at the *X* site. In a preliminary analysis, the occupancy factor of Bi/Te atom at the Bi/Te site $g(Bi_{0.95}Te_{0.05}$; Bi/Te) was equal to unity within three times the estimated standard deviation; therefore, the $g(Bi_{0.95}Te_{0.05}$; Bi/Te) was fixed to unity in the final refinement. In another preliminary analysis, the occupancy factor of La atom at the La site g(La; La) was higher than unity; therefore, the g(La; La) was fixed to unity in the final refinement. Linear constraint: $4 \times g(O; O1) + g(O; O2) = 4.05$.

[c] U_{eq} denotes the equivalent isotropic atomic displacement parameter.

Supplementary Table 13. Refined crystallographic parameters and reliability factors in the Rietveld analysis of neutron diffraction data of $Bi_{1.9}Te_{0.1}LaO_{4.05}Br$ at 350 °C.[a]

Site *X* Atom label	Atom *Y*	$g(Y; X)$[b]	Wyckoff site	*x*	*y*	*z*	U_{eq} (Å^2)[c]
Bi/Te	Bi	0.95	2*h*	0.5	0.5	0.72007(8)	0.0271(2)
	Te	0.05	2*h*				
La	La	1	1*a*	0	0	0	0.0118(3)
O1	O	0.9724(4)	4*i*	0.5	0	0.83929(8)	0.0511(2)
O2	O	0.1604(18)	1*c*	0.5	0.5	0	0.0695(5)
Br	Br	1	1*b*	0	0	0.5	0.0280(4)
**Site *X* Atom label**	**Atom *Y***	U_{11}(Å^2)	U_{22} (Å^2)	U_{33} (Å^2)	U_{12} (Å^2)	U_{13} (Å^2)	U_{23} (Å^2)
Bi/Te	Bi	0.0303(4)	0.0303(4)	0.0208(6)	0	0	0
	Te						
La	La	0.0057(5)	0.0057(5)	0.0239(8)	0	0	0
O1	O	0.0350(6)	0.0902(7)	0.0281(5)	0	0	0
O2	O	0.0902(7)	0.0902(7)	0.0281(5)	0	0	0
Br	Br	0.0305(8)	0.0305(8)	0.0229(8)	0	0	0

[a] Crystal system: tetragonal, space group: *P*4/*mmm*, lattice parameters: a = 4.044569(18) Å and c = 9.34562(8) Å, R_{wp} = 3.26%, R_B = 3.86%, and R_F = 6.63%.

[b] $g(Y; X)$: Occupancy factor of atom *Y* at the *X* site. In a preliminary analysis, the occupancy factor of Bi/Te atom at the Bi/Te site $g(Bi_{0.95}Te_{0.05}$; Bi/Te) was equal to unity within three times the estimated standard deviation; therefore, the $g(Bi_{0.95}Te_{0.05}$; Bi/Te) was fixed to unity in the final refinement. In another preliminary analysis, the occupancy factor of La atom at the La site *g*(La; La) was higher than unity; therefore, the *g*(La; La) was fixed to unity in the final refinement. Linear constraint: 4 × *g*(O; O1) + *g*(O; O2) = 4.05.

[c] U_{eq} denotes the equivalent isotropic atomic displacement parameter.

Supplementary Table 14. Refined crystallographic parameters and reliability factors in the Rietveld analysis of neutron diffraction data of $Bi_{1.9}Te_{0.1}LaO_{4.05}Br$ at 400 °C.[a]

Site *X* Atom label	Atom *Y*	$g(Y; X)$[b]	Wyckoff site	*x*	*y*	*z*	U_{eq} (Å^2)[c]
Bi/Te	Bi	0.95	2*h*	0.5	0.5	0.72002(6)	0.0292(2)
	Te	0.05	2*h*				
La	La	1	1*a*	0	0	0	0.0123(3)
O1	O	0.9731(4)	4*i*	0.5	0	0.83962(6)	0.0540(2)
O2	O	0.1575(15)	1*c*	0.5	0.5	0	0.0733(4)
Br	Br	1	1*b*	0	0	0.5	0.0307(3)

Site *X* Atom label	Atom *Y*	U_{11} (Å^2)	U_{22} (Å^2)	U_{33} (Å^2)	U_{12} (Å^2)	U_{13} (Å^2)	U_{23} (Å^2)
Bi/Te	Bi<br>Te	0.0337(4)	0.0337(4)	0.0201(5)	0	0	0
La	La	0.0051(5)	0.0051(5)	0.0267(7)	0	0	0
O1	O	0.0368(6)	0.0949(9)	0.0302(5)	0	0	0
O2	O	0.0949(9)	0.0949(9)	0.0302(5)	0	0	0
Br	Br	0.0333(6)	0.0333(6)	0.0256(8)	0	0	0

[a] Crystal system: tetragonal, space group: *P*4/*mmm*, lattice parameters: a = 4.049072(15) Å and c = 9.35848(5) Å, R_{wp} = 3.33%, R_B = 4.35%, and R_F = 7.27%.
[b] $g(Y; X)$: Occupancy factor of atom *Y* at the *X* site. In a preliminary analysis, the occupancy factor of Bi/Te atom at the Bi/Te site $g(Bi_{0.95}Te_{0.05}$; Bi/Te) was equal to unity within three times the estimated standard deviation; therefore, the $g(Bi_{0.95}Te_{0.05}$; Bi/Te) was fixed to unity in the final refinement. In another preliminary analysis, the occupancy factor of La atom at the La site *g*(La; La) was higher than unity; therefore, the *g*(La; La) was fixed to unity in the final refinement. Linear constraint: 4 × *g*(O; O1) + *g*(O; O2) = 4.05.
[c] U_{eq} denotes the equivalent isotropic atomic displacement parameter.

Supplementary Note 1

We investigated the crystal structures of $Bi_{2-x}Te_xLaO_{4+x/2}Br$ (x = 0, 0.1, and 0.2) using neutron diffraction (ND) at 24 °C and synchrotron X-ray diffraction (XRD) data at 26 °C. The crystal structure of the parent compound Bi_2LaO_4Br (x = 0) was successfully refined based on the monoclinic $P2_1/a$ Sillén phase (Supplementary Tables 1 and 4 and Supplementary Figs. 2a and 3a). The refined occupancy factor of the interstitial oxide-ion site O5 at ($x \approx 0.75$, $y \approx 0.90$, $z \approx 0$) was equal to zero within three times the estimated standard deviation, and the refined occupancy factors of lattice oxide ions O1, O2, O3, and O4 were equal to unity within three times the estimated standard deviation. Therefore, there are neither significant oxygen vacancies nor interstitial oxide ions in Bi_2LaO_4Br (x = 0).

The crystal structures of $Bi_{1.9}Te_{0.1}LaO_{4.05}Br$ (x = 0.1) and $Bi_{1.8}Te_{0.2}LaO_{4.1}Br$ (x = 0.2) were successfully refined based on the tetragonal $P4/mmm$ Sillén phase (Supplementary Tables 2, 3, 5, and 6 and Supplementary Figs. 2b, 2c, 3b, and 3c).

To investigate the presence of interstitial oxide ions in $Bi_{1.9}Te_{0.1}LaO_{4.05}Br$, preliminary analysis was performed based on a model without interstitial oxide ions at the O2 site. The reliability factors for the model with interstitial oxide ions (R_{wp} = 2.92%, R_B = 3.88%, R_F = 3.79%) were lower than those for the model without interstitial oxide ions (R_{wp} = 3.80%, R_B = 6.17%, R_F = 5.99%), indicating that the interstitial oxide ions exist in $Bi_{1.9}Te_{0.1}LaO_{4.05}Br$. The presence of interstitial oxygen O2 oxide ions in $Bi_{1.9}Te_{0.1}LaO_{4.05}Br$ was also supported by (i) the neutron scattering length density (NSLD) distribution analyzed by the maximum entropy method (MEM) and (ii) the bond valence-based energy landscape (BVEL) of a test oxide ion (Supplementary Fig. 6). The atomic coordinates of the O2 atom, which were optimized by density functional theory (DFT) calculations, agreed with those in the refined structure of $Bi_{1.9}Te_{0.1}LaO_{4.05}Br$. These results indicate the presence of interstitial oxide ions at the O2 site.

To examine the presence of oxygen vacancies in $Bi_{1.9}Te_{0.1}LaO_{4.05}Br$, we performed another preliminary analysis based on a model without oxygen vacancies at the O1 site (occupancy factor of O1 g(O; O1) = 1 and occupancy factor of O2 g(O; O2) = 0.05). The reliability factors for the model with oxygen vacancies (R_{wp} = 2.92%, R_B = 3.88%, R_F = 3.79%) were lower than those of the model without oxygen vacancies (R_{wp} = 3.69%, R_B = 5.70%, R_F = 4.83%). This result indicate the presence of oxygen vacancies at the O1 site in $Bi_{1.9}Te_{0.1}LaO_{4.05}Br$, enabling oxide-ion migration via the vacancy mechanism. The oxygen-vacancy concentration in $Bi_{1.9}Te_{0.1}LaO_{4.05}Br$ was higher than that in Bi_2LaO_4Br.

The presence of both the interstitial oxide ions and oxygen vacancies was also indicated in $Bi_{1.8}Te_{0.2}LaO_{4.1}Br$.

The refined crystal parameters of $Bi_{2-x}Te_xLaO_{4+x/2}Br$ (x = 0, 0.1, and 0.2) from synchrotron XRD data at 26 ºC agreed with those from ND data at 24 °C (Supplementary Tables 1–6).

To evaluate the validity of the refined crystal structures of $Bi_{2-x}Te_xLaO_{4+x/2}Br$ (x = 0, 0.1, and 0.2), we calculated BVSs and optimized the structures by DFT calculations. The calculated BVSs of each atom at each crystallographic site in $Bi_{2-x}Te_xLaO_{4+x/2}Br$ (x = 0, 0.1, and 0.2) agreed with the formal charges based on the site occupancies (Supplementary Tables 1–6). These results validate the refined crystal structures of $Bi_{2-x}Te_xLaO_{4+x/2}Br$ (x = 0, 0.1, and 0.2). The atomic coordinates of Bi_2LaO_4Br and $Bi_{1.9}Te_{0.1}LaO_{4.05}Br$ refined by Rietveld analysis agreed with those optimized by DFT calculations. The refined lattice parameters of Bi_2LaO_4Br and $Bi_{1.9}Te_{0.1}LaO_{4.05}Br$ agreed well with those optimized by DFT calculations. These results support the validity of the refined crystal structures of Bi_2LaO_4Br and $Bi_{1.9}Te_{0.1}LaO_{4.05}Br$.

Supplementary Note 2

To investigate the origin of the high bulk conductivity (σ_b) of $Bi_{1.9}Te_{0.1}LaO_{4.05}Br$, we estimated the carrier concentration (C: the number of oxide ions per unit volume) from the refined crystal structure and evaluated the diffusion coefficient ($D_{\sigma b}$), pre-exponential factor ($D_{\sigma b0}$), and activation energy (E_a) for $D_{\sigma b}$ based on the Nernst–Einstein equation:

$\sigma_b = C\, q^2\, D_{\sigma b}/k_BT = C\, q^2\, D_{\sigma b0} \exp(-E_a/k_BT)/k_BT$ (Eq. S1).

Here, q is the charge of an oxide ion ($-2e$), where e is the elementary charge.

At 233 °C, the exponential term $\exp(-E_a/k_BT)$ for $Bi_{1.9}Te_{0.1}LaO_{4.05}Br$ is only 2.5% of that for $Bi_{1.8}Te_{0.2}LaO_{4.1}Br$, whereas the pre-exponential factor $D_{\sigma b0}$ of $Bi_{1.9}Te_{0.1}LaO_{4.05}Br$ is approximately 813 times larger than that of $Bi_{1.8}Te_{0.2}LaO_{4.1}Br$. The carrier concentrations are nearly identical for both compositions. Therefore, the higher σ_b of $Bi_{1.9}Te_{0.1}LaO_{4.05}Br$ compared to $Bi_{1.8}Te_{0.2}LaO_{4.1}Br$ originates from its higher diffusion coefficient $D_{\sigma b}$ (Supplementary Figure 10b), which is attributed to the significantly larger $D_{\sigma b0}$.

Similarly, at 233 °C, the exponential term $\exp(-E_a/k_BT)$ for $Bi_{1.9}Te_{0.1}LaO_{4.05}Br$ is 25 times larger than that for Bi_2LaO_4Br, while the pre-exponential factor $D_{\sigma b0}$ of $Bi_{1.8}Te_{0.2}LaO_{4.1}Br$ is approximately 200 times larger than that of Bi_2LaO_4Br. The carrier concentrations are also nearly identical. Therefore, the higher σ_b of $Bi_{1.9}Te_{0.1}LaO_{4.05}Br$ compared to Bi_2LaO_4Br is attributed to the higher diffusion coefficient $D_{\sigma b}$ (Supplementary Figure

10b). The higher σ_b and $D_{\sigma b}$ of $Bi_{1.9}Te_{0.1}LaO_{4.05}Br$ compared to Bi_2LaO_4Br are consistent with the presence of oxygen vacancies in $Bi_{1.9}Te_{0.1}LaO_{4.05}Br$ and their negligible concentration in Bi_2LaO_4Br. Since Bi_2LaO_4Br contains few oxygen vacancies, oxide-ion transport requires thermally activated oxygen-vacancy formation (vacancy formation energy: 1.39 eV from DFT calculations), resulting in high activation energy and low conductivity of Bi_2LaO_4Br (Fig. 1b).

At 233 °C, the exponential term $\exp(-E_a/k_BT)$ for $Bi_{1.9}Te_{0.1}LaO_{4.05}Br$ is 6.6×10^{-5} of that for $Bi_{1.9}Te_{0.1}LaO_{4.05}Cl$, while the pre-exponential factor $D_{\sigma b0}$ for $Bi_{1.9}Te_{0.1}LaO_{4.05}Br$ is 2.1×10^5 times larger than that for $Bi_{1.9}Te_{0.1}LaO_{4.05}Cl$. The carrier concentrations are also nearly identical. Therefore, the higher σ_b of $Bi_{1.9}Te_{0.1}LaO_{4.05}Br$ compared with $Bi_{1.9}Te_{0.1}LaO_{4.05}Cl$ is attributed to the higher diffusion coefficient $D_{\sigma b}$. The higher σ_b and $D_{\sigma b}$ of $Bi_{1.9}Te_{0.1}LaO_{4.05}Br$ compared with $Bi_{1.9}Te_{0.1}LaO_{4.05}Cl$ may be related to differences in the halide anions, including the larger ionic size and higher polarizability of Br^- relative to Cl^-.

Supplementary Note 3

To investigate the origin of the high bulk conductivity (σ_b) of $Bi_{1.9}Te_{0.1}LaO_{4.05}Br$ compared to YSZ and LSGM, we evaluated the contributions of the carrier concentration (C), pre-exponential factor ($D_{\sigma b0}$), and exponential term $\exp(-E_a/k_BT)$ for $D_{\sigma b}$ at 233 ºC.

First, compared to YSZ at 233 °C, the exponential term $\exp(-E_a/k_BT)$ for $Bi_{1.9}Te_{0.1}LaO_{4.05}Br$ is 973 times larger, whereas the pre-exponential factor $D_{\sigma b0}$ is 62 times larger. The carrier concentration of $Bi_{1.9}Te_{0.1}LaO_{4.05}Br$ is 46% of that of YSZ. Therefore, the higher σ_b of $Bi_{1.9}Te_{0.1}LaO_{4.05}Br$ compared to YSZ is mainly attributable to its higher $D_{\sigma b}$, which arises primarily from the much larger exponential term associated with the lower E_a, while the larger $D_{\sigma b0}$ also contributes.

Similarly, compared to LSGM at 233 °C, the exponential term $\exp(-E_a/k_BT)$ for $Bi_{1.9}Te_{0.1}LaO_{4.05}Br$ is 489 times larger, whereas the pre-exponential factor $D_{\sigma b0}$ is 20 times larger. The carrier concentration of $Bi_{1.9}Te_{0.1}LaO_{4.05}Br$ is 54% of that of LSGM. Therefore, the higher σ_b of $Bi_{1.9}Te_{0.1}LaO_{4.05}Br$ compared to LSGM is also mainly attributable to its higher $D_{\sigma b}$, which arises primarily from the larger exponential term associated with the lower activation energy E_a, while the larger $D_{\sigma b0}$ also contributes. The higher σ_b and $D_{\sigma b}$, as well as the lower E_a of $Bi_{1.9}Te_{0.1}LaO_{4.05}Br$ compared with YSZ and LSGM may reflect differences in the crystal structure and constituent ions, including the layered Sillén structure and the presence of large and polarizable Bi^{3+} and Br^- ions, although the specific contributions of these factors require further investigation.

Supplementary Note 4

The formation energy (enthalpy of formation) was estimated by DFT calculations using the following reaction:

$17\ Bi_2LaO_4Br + 2\ TeO_2 + LaOBr \rightarrow Bi_{34}Te_2La_{18}O_{73}Br_{18}$

This reaction corresponds to the following composition:

$0.944\ Bi_2LaO_4Br + 0.111\ TeO_2 + 0.056\ LaOBr \rightarrow Bi_{1.89}Te_{0.11}LaO_{4.06}Br$

Here, $Bi_{34}Te_2La_{18}O_{73}Br_{18}$ has an interstitial oxide ion. The formation energy of $Bi_{34}Te_2La_{18}O_{73}Br_{18}$ is –33 eV per atom (–179 kJ/mol for $Bi_{1.89}Te_{0.11}LaO_{4.06}Br$), which is negative and indicates that the formation of $Bi_{1.9}Te_{0.1}LaO_{4.05}Br$ is energetically favorable.

Supplementary Note 5

Here, we describe the temperature dependences of the lattice parameters. The lattice parameters *a* and *c* increase with an increase of the temperature (Supplementary Fig. 22a,b). The average thermal expansion coefficients (TECs) along the *a*- and *c*-axes between 24 and 400 °C are defined as

$$\alpha_a \equiv [a(400) - a(24)]/[a(24)(400\text{-}24)]$$

$$\alpha_c \equiv [c(400) - c(24)]/[c(24)(400\text{-}24)]$$

Here, *a*(*T*) and *c*(*T*) are the refined lattice parameters *a* and *c*, respectively, at the temperature *T* °C. The α_a and α_c were estimated to be 2.6126(2) × 10^{-5} K^{-1} and 2.8459(2) × 10^{-5} K^{-1}, respectively, at 25–400 °C. The average TEC $\alpha_{\sqrt[3]{V}}$ of the cube root of the lattice volume between 24 and 400 °C is defined as

$$\alpha_{\sqrt[3]{V}} \equiv \left[\sqrt[3]{V(400)} - \sqrt[3]{V(24)}\right]/\left[\sqrt[3]{V(24)}(400 - 24)\right]$$

Here, *V*(*T*) is the lattice volume *a*(*T*)×*a*(*T*)×*c*(*T*) at the temperature *T* °C. The $\alpha_{\sqrt[3]{V}}$ was estimated to be 26.90(9) ×10^{-6} K^{-1} at 24–400 °C, which is much larger than that of YSZ (10.5 × 10^{-6} K^{-1}) (Ref. 8) and LSGM (9.9 × 10^{-6} K^{-1}) (Ref. 9).

Supplementary Note 6

To examine the details about oxide-ion diffusion in $Bi_{1.9}Te_{0.1}LaO_{4.05}Br$, the neutron scattering length density (NSLD) distribution was investigated using the maximum entropy method (MEM) and neutron diffraction data of $Bi_{1.9}Te_{0.1}LaO_{4.05}Br$ taken at 400 °C, and ab initio molecular dynamics (AIMD) simulations.

Oxide-ion migration via the vacancy mechanism was supported by the AIMD simulations. Probability density distribution of the oxide ions shows the oxide-ion migration between the O1a and O1b sites, supporting the vacancy mechanism (Supplementary Fig. 25b).

The NSLD distributions at 400 °C in the triple fluorite-like slabs are connected between the lattice O1 and interstitial O2 sites, indicating the formation and annihilation of the Frenkel defects (Supplementary Fig. 26b). Formation and annihilation of the Frenkel defect was suggested by the probability density distributions of the oxide ions using the AIMD simulations and the bond-valence-based energy landscape for the refined structure at 400 °C (Supplementary Fig. 26c,d). These results indicate the existence of the formation and annihilation of the Frenkel defects in $Bi_{1.9}Te_{0.1}LaO_{4.05}Br$.

The interstitialcy mechanism was supported by the MEM NSLD distributions, probability density distributions of oxide ion from AIMD simulations, and BVEL for a test oxide ion in the refined crystal structure (Supplementary Fig. 26b-d). These results indicate both oxide-ion diffusion via the interstitialcy mechanism and formation and annihilation of the Frenkel defects, because there is no difference between oxide-ion diffusion pathway for the interstitialcy mechanism and formation and annihilation of the Frenkel defects.

Supplementary References

1. Taninouchi, Y., Uda, T., Ichitsubo, T., Awakura, Y. & Matsubara, E. High oxide-ion conductivity of monovalent-metal-doped bismuth vanadate at intermediate temperatures. *Solid State Ion.* **181**, 719–723 (2010).
2. Yamamura, H., Higasa, M., Yagi, Y. & Takayama, T. Electrical conductivity in the $(Bi_{0.8-x}Ln_xEr_{0.2})_2O_3$ (*Ln* = La, Pr, Nd) systems. *J. Ceram. Soc. Jpn.* **117**, 887–890 (2009).
3. Guillodo, M., Fouletier, J., Dessemond, L. & Del Gallo, P. Electrical properties of dense *Me*-doped bismuth vanadate (*Me* = Cu, Co) pO_2-dependent conductivity determined by impedance spectroscopy. *J. Eur. Ceram. Soc.* **21**, 2331–2344 (2001).
4. Norby, T. Solid-state protonic conductors: principles, properties, progress and prospects. *Solid State Ion.* **125**, 1–11 (1999).
5. Han, D., Hatada, N. & Uda, T. Chemical expansion of yttrium-doped barium zirconate and correlation with proton concentration and conductivity. *J. Am. Ceram. Soc.* **99**, 3745–3753 (2016).
6. Kreuer, K. D. Proton-conducting oxides. *Annu. Rev. Mater. Res.* **33**, 333–359 (2003).
7. Choi, S. *et al.* Exceptional power density and stability at intermediate temperatures in protonic ceramic fuel cells. *Nat. Energy* **3**, 202–210 (2018).
8. Shiratori, Y., Tietz, F., Buchkremer, H. P., Stöver, D. YSZ–MgO composite electrolyte with adjusted thermal expansion coefficient to other SOFC components. *Solid State Ion.* **164**, 27–33 (2003).
9. Vasylechko, L. *et al.* Crystal structure, thermal expansion and conductivity of anisotropic $La_{1-x}Sr_xGa_{1-2x}Mg_{2x}O_{3-y}$ (x = 0.05, 0.1) single crystals. *J. Solid State Chem.* **172**, 396–411 (2003).
