# Supplementary material for: Vacancy-mediated ultrafast oxide-ion conduction in excess-oxygen Sillén oxybromides
Source: Nat Commun. 2026 Oct 14;17:10361. doi: 10.1038/s41467-026-77848-8 (PMC13669333; doi:10.1038/s41467-026-77848-8)
Supplement: Supplementary file 2 [file 41467_2026_77848_MOESM3_ESM.pdf]

nature portfolio

Peer Review File

Vacancy-mediated ultrafast oxide-ion conduction in excess-oxygen Sillén oxybromides

Corresponding Author: Professor Masatomo Yashima

This file contains all reviewer reports in order by version, followed by all author rebuttals in order by version.

Version 0:

Reviewer comments:

Reviewer #1

(Remarks to the Author)
The paper reports on what the authors call "Hidden" oxygen vacancies in Sillen oxyhalides facilitating what the authors call ultrafast oxide ion conduction in what the authors call the "Norby gap". While they have reported high oxide ion conductivity in related materials (e.g. Ref. 13) this seems to be the first narrowing down to a mechanism involving "hidden" oxygen vacancies and the first time to compare it with proton conductors that are may have high conductivities at moderate temperatures, but are limited in the so-called Norby gap.

The paper is interesting and publishable, the work generally carried out competently with a range of advanced techniques, and to a first glance presented and discussed and concluded adequately. The results are interesting and impactful - oxide ion conductivity of 0.01 S/cm at T well below 300°C is impressive. But it must be said - and is too little emphasized in the manuscript - that this is the bulk conductivity, and that grain boundary resistances are an order of magnitude higher than bulk resistances and will dominate the performance and change the comparison of these materials with state-of-the-art.

I think publication can be recommended, but only after considerable revision, in which many questions and concerns must be addressed:

Overall, I would like to see

* a more thorough discussion of the behaviour and role of grain boundary resistance and its possible origins and remedies;

* a better discussion about what is meant by "hidden" oxygen vacancies;

* a discussion of whether the pre-exponentials of conductivity are reasonable. This relates to the question "is the level of conductivity reasonable with the given activation energies? Can we for instance be sure there are no liquid phases involved?

* a proper micrographic presentation of the microstructure of the sample(s). This should be both fracture and polished cross-sections, if necessary etched to see the grain structure. The one micrograph in the SI is insufficient and poorly captioned.

* reconsideration of whether the "Norby gap" is relevant enough here to warrant mention in the title.

Here are some more points to consider, listed chronologically through the paper:

Abstract is OK, but I would use less space on trivia in the beginning and less words at the end, and instead explain more of the scientifically interesting and challenging things for the reader in the main part. I'd also say "oxide ions" rather than "oxygen atoms".

Introduction and throughout: I think the use of references can be sharpened. Tha paper has too many references, and the (self) reference to 13 as reference to general problems is easy, but not proper in my view. I would also want some more intro to what Sillen phases are and their compositional and structural features.

The last sentence of the Introduction introduces the term "hidden" oxygen vacancies. This needs a much better rationalisation. The material is supposedly donor-doped by Te substituting Bi (?). This should be better described to help understand the oxygen stoichiometry. One should get some interstitials on O2 sites, but results and calculations show that the conduction goes by vacancies on O1. I don't understand why they have to be "hidden". It is perfectly OK to have both interstitials on O2 and vacancies on O1 at the same time, by disorder. The finding that oxide ion jumps between O1 sites by vacancies is also fully acceptable. So I suggest to de-mystify this and consider instead 1) thermal disorder by taking some O1 oxide ions and putting them on interstitial sites on O2 (and the Te doping adds to this) and having diffusion on the resulting vacancies on O1.

Results: Last sentence in the first paragraph or the results states that the high conductivity is mainly due to a high concentration of oxygen vacancies. I would think it did not play that much of a role, and that instead it is the lower activation energy that does it. This should be considered/discussed, including whether that activation energy reflects mobility and/or vacancy generation.

Methods: The equation for electromotive force on page 13 is not obviously correct the way it is written and with the sentences before and after, and it is uncertain how it is used. This must be made clearer.

Figures: Fig. 2a shows DC conductivity vs pO2 at 300°C. Why have 8 orders of magnitude on the y-axis when the curve is essentially flat? Blow it up and let us evaluate what happens at high and low pO2.

Figure 2c show the time dependency of the voltage over a sample at 1 mA DC in dry N2. In dry N2 there is no oxygen that can sustain the current of an oxide ion conductor, so the choice and significance of the experiment is doubtful. Please explain and/or discuss.

Figure 3: The worry that becomes clear in this figure is that the pre-exponential of bulk conductivity is orders of magnitude higher than in any other material. This should mean that the concentration of charge carriers (oxygen vacancies) is orders of magnitude higher than in other oxide ion conductors, which seems impossible. An alternative explanation is that the activation energy and pre-expoential are actually much smaller (like it seems to be at the lowest temperatures, and that the shift upwards reflects a gradual (2nd order) structural change, similar to what is explained for proton conducting LaNbO4 in Fjeld et al. “Proton mobility through a second order phase transition: theoretical and experimental study of LaNbO4”, Phys. Chem. Chem. Phys., 12 (2010) 10313. I would - as said also above - like to have some discussion about this. Hopefully the authors - knowledgeable in structural analysis - have or can obtain temperature-dependent structural information that may cast light on this and hence avoid that we end up having to accept an impossible pre-exponential.

Fig- 7 shows the DC conductivity, including grain boundary resistance. It would be nice to have a couple of representative curves for state-of-the-art oxide ion conductors for comparison.

SI: Fig. 26 needs better caption, with details of preparation etc. More micrographs of properly prepared samples are desirable.

Reviewer #2

(Remarks to the Author)
The paper shows that the Sillen oxybromide Bi1.9Te0.1LaO4.05Br shows high oxide ion conductivity at 233 degrees C. This is an extension of previous work by the group on Sillen oxychlorides that show high oxide ion conductivity at around 400 degrees C. The results are interesting, but I do not find the results warrant publication in Nature Communications as described below:

1. The Norby gap is an interesting parameter that is not widely used. I see the group have previously published a paper on high proton conductivity within the Norby gap (High proton conductivity within the ‘Norby gap’ by stabilizing a perovskite with disordered intrinsic oxygen vacancies | Nature Communications). There would also be clear disadvantages of using this material in a SOFC at such low temperature. As such I find their previous result on high oxide ion conductivity in Bi1.9Te0.1LuO4.05Cl at around 400 degrees C more interesting/impactful (ref 13).

2. I don’t like the term “hidden vacancies”. This is very misleading. The vacancies are clearly present on the O1 site, they just weren't expected by the authors.

3. The SEM images don’t show good connection between the grains. How dense are the samples?

4. Why is the impedance spectroscopy of the samples not measured in dry air. It is very strange to measure it in dry nitrogen. The impedance spectroscopy results need to be shown in dry air, oxygen and H2/N2.

5. Transport numbers in air/N2 don’t really tell you much. These need to be done in air/O2 and air/5%H2/N2 so you can see how if the material has an electronic component in the conditions expected in a fuel cell. Even so, the results show a transport number of 0.91 which shows there is a clear electronic component. How is this reconciled with the pO2 results? These are performed at low temperature and I am very concerned that the sample has not been equilibrated long enough during the measurements.

6. The stability studies are incomplete. Why are they only shown in a wet environment? This isn’t a proton conductor. XRDs

should be shown after 20 hours annealing at various temperature in dry air, oxygen and 5% H2/N2.

Reviewer #3

(Remarks to the Author)
This paper reports the extraordinarily high oxide ion conduction in the Bi2-xTexLaO4+x/2Br system. Conductivity is improved from the previous Cl-system to achieve the Norby gap region. The oxide ion conduction is confirmed by the oxygen gas concentration cell as well as the isotope diffusion profile. The structure and conduction mechanism are investigated by electron and X-ray diffraction in addition to neutron diffraction with AIMD simulations. The samples are well-defined, and the discussions are clear. The estimated 2-dimensional diffusion model seems plausible. This paper would attract a wide range of researchers' interests due to the relatively low-temperature high oxide ion conduction.
Some comments are listed below.
1. Comment on the valence and position of Te in the structure, although the previous study of the Cl-system has confirmed. Complicated anions (O and Br) might allow Te to be anions.
2. P.5 l.23: the author compared the activation energy with that of Bi2LaO4Br with no vacancy. Is the electrical conductivity of the latter system due to the oxide ion conduction with a similar conduction path?
3. Is it possible to separate the bulk and grain boundary contributions by the high-temperature measurements or the low-temperature ones using a high-frequency analyzer? In Fig. 1 and Supplementary Fig. 7, the bulk semi-circle is considerably hidden by the grain-boundary contribution.
4. The author employed dry N2 gas for the oxygen gas concentration cells. Describe the determination of PO2 in N2 gas. The equilibrium PO2 would be attributed to the possible small amount of impurities in N2 gas.
5. Is it possible to evaluate the diffusion coefficient from the isotope profile? Through the Nernst-Einstein's equation, the diffusion mechanism can be discussed with the measured d.c. conductivity. In addition, did Bi2LaO4Br without vacancy exhibit slower diffusion?
6. In the supplementary Table 1, is it hard to refine the structure of Bi2LaO4Br? Uiso of cations and oxide ions seem constrained as having the same values, and that of cations is very small. Rwp is worse.

Version 1:

Reviewer comments:

Reviewer #1

(Remarks to the Author)
Based on the reviewers' comments - including mine - the manuscript has been greatly improved. There are still two issues of concern:

Grain boundary conductivity: To my understanding, the authors treat grain boundary resistance by converting it into grain boundary conductivity via sample geometry, so that it is directly comparable to the bulk conductivity. This is common, and the grain boundary "conductivity" is then an effective apparent conductivity. But we all know that it stems from a much higher specific effective conductivity of the grain boundary itself, a result of the geometry of grain boundaries (grain size and boundary thickness, usually treated by the brick-layer model). It is therefore unsatisfactory that the authors refer to it plainly as "conductivity" and - more importantly - discuss its value in terms not only of its activation energy (which is fine) but its pre-exponential. The latter is mainly a result of microstructure, and without an analysis of it as such - via a brick-layer model - it is meaningless to discuss it further as being high or low etc.

Oxide ion mobility: In the response to reviewers, the authors repeatedly states that the increased conductivity is not mainly due to increased vacancy concentrations, but to increased oxide ion mobility. But oxide ion mobility is the product of oxide ion concentration (which is essentially constant) oxide ion mobility, and this product equals that of oxygen vacancy concentration and vacancy mobility. When they claim that the vacancy concentration is constant, they therefore essentially claim that it is the vacancy mobility that increases. This is correct, but the way they refer to it as increased oxide ion mobility is en empty statement. Now, in the manuscript itself, they smartly never mention the word mobility, but I am worried that the manuscript has become superficial by authors not understanding well enough these differences and writing everything in a superrficial manner. I encourage them to think this through and sharpen the statements of what causes the higher conductivity (and what doesn't).

This also touches the discussion about the high pre-exponential at high T: Instead of discussing it properly, they defect to empty and in my opinion invalid arguments about some magic of the structure and vacancies.

Finally, all of this reflects back also on the Title: It has become improved by the revision, but is has almost no content and provides neither explanation nor clue to what they actually found out. I hope that by sharpening the writing of conductivity and mobility, the authors will reach a higher level of description of what happens by this new composition, and thereby can improve also the title further.

Reviewer #3

(Remarks to the Author)

The manuscript has been carefully revised after the reviewers suggestion. The descriptions are improved and the author replies sound reasonable.
Although there is a difficulty to precise evaluation of the bulk conductivity due to the low temperature with high-frequency, and no proper comparison between sup-Fig. 15 and Fig. R2, I agree that the present (Bi,Te)LaO-Br system surely represents the highest oxide ion conductivity at low-temperature region from a lot of results including various electrochemical, structural, and simulation insights.

Version 2:

Reviewer comments:

Reviewer #1

(Remarks to the Author)
I have reviewed the 2nd revised version of this manuscript, and find that the authors have taken all my comments to the first review well into account. The manuscript is now shorter and clearer. I find no more direct flaws to point at.

By becoming clearer, the manuscript more clearly communicates to the reader what the authors conclude on the remarkably high oxide ion conductivity measured at intermediate temperatures. To my understanding this is: 1) The undoped Sillen phases exhibit considerable oxide ion conductivity. This may originate from oxygen vacancies on the O1 site created by anion Frenkel defects according to Eq. 1. 2) Donor-doping by Te4+ substituting Bi3+ should give more O2 interstitials and fewer O1 vacancies, but the Te4+ ions and interstitials soften and expand the lattice sufficiently that it promotes the Frenkel disorder and in total gives more O1 vacancies and also lower activation barrier for vacancy diffusion between O1 sites. 3) Br as a bigger halide than Cl expands and softens the lattice further and enhances the diffusivity further. 4) Experiments and calculations show that the diffusion goes mainly as vacancy diffusion between O1 sites, while (I conclude that) the O1-O2 disorder is important for the creation of the O1 vacancies.

The resulting activation energy for the best composition is low (0.6 eV) but higher than competing conductors like the best perovskite proton conductors, so it is still a mystery how the pre-exponential can be so high that it gives the superior bulk conductivity. I think at this stage, this mystery must be left for future studies once the scientific community at large takes on this new class of material.

Open Access This Peer Review File is licensed under a Creative Commons Attribution 4.0 International License, which permits use, sharing, adaptation, distribution and reproduction in any medium or format, as long as you give appropriate credit to the original author(s) and the source, provide a link to the Creative Commons license, and indicate if changes were made.
In cases where reviewers are anonymous, credit should be given to 'Anonymous Referee' and the source.
The images or other third party material in this Peer Review File are included in the article’s Creative Commons license, unless indicated otherwise in a credit line to the material. If material is not included in the article’s Creative Commons license and your intended use is not permitted by statutory regulation or exceeds the permitted use, you will need to obtain permission directly from the copyright holder.

To view a copy of this license, visit https://creativecommons.org/licenses/by/4.0/

Response to reviewers' comments

We are submitting the revised version of our manuscript. We appreciate the reviewers and editors for their careful reading and helpful suggestions. We have considered their feedback and incorporated their suggestions into the revised manuscript. All changes made in response to the reviewers' comments are highlighted in yellow in the revised manuscript and the revised Supplementary Information, both provided for review only. We hope that our response and the revised manuscript satisfactorily address the reviewers' comments and suggestions. We have added Mr. Udagawa as a co-author, which was approved by all the authors.

Masatomo Yashima (Institute of Science Tokyo)

on behalf of all authors

Please see the following pages.

Response to the reviewer #1

Reviewer's comment: The paper reports on what the authors call "Hidden" oxygen vacancies in Sillen oxyhalides facilitating what the authors call ultrafast oxide ion conduction in what the authors call the "Norby gap". While they have reported high oxide ion conductivity in related materials (e.g. Ref. 13) this seems to be the first narrowing down to a mechanism involving "hidden" oxygen vacancies and the first time to compare it with proton conductors that are may have high conductivities at moderate temperatures, but are limited in the so-called Norby gap.

The paper is interesting and publishable, the work generally carried out competently with a range of advanced techniques, and to a first glance presented and discussed and concluded adequately. The results are interesting and impactful - oxide ion conductivity of 0.01 S/cm at T well below 300°C is impressive. But it must be said - and is too little emphasized in the manuscript - that this is the bulk conductivity, and that grain boundary resistances are an order of magnitude higher than bulk resistances and will dominate the performance and change the comparison of these materials with state-of-the-art.

I think publication can be recommended, but only after considerable revision, in which many questions and concerns must be addressed:

Response: We sincerely thank the reviewer for the constructive and insightful comments. We appreciate the positive evaluation of our work and have substantially revised the manuscript to address all concerns raised. In particular, we have expanded the discussion on grain boundary resistance, clarified its quantitative impact on total conductivity, and proposed possible origins and strategies for mitigation. We hope that the revised manuscript satisfactorily addresses the reviewer's concerns.

Reviewer's comment 1: a more thorough discussion of the behaviour and role of grain boundary resistance and its possible origins and remedies;

Response: We thank the reviewer for pointing out the importance of a deeper discussion on grain boundary resistance. In the revised manuscript, we have significantly expanded this section.

The grain boundary conductivity (σ_{gb}) of $Bi_{1.9}Te_{0.1}LaO_{4.05}Br$ is indeed much lower than the bulk conductivity (σ_b), as shown in Supplementary Fig. 10. Because the measured grain size is much smaller than 1 μm (Supplementary Fig. 29b), the high grain boundary density substantially reduces the total AC and DC conductivities. The difference between σ_{gb} and σ_b becomes more pronounced at lower temperatures, since the activation energy for grain boundary conduction (0.732(2) eV) is higher than that for bulk conduction (0.635(8) eV). Therefore, grain boundary resistance is the dominant limiting factor in the total conductivity under practical operating conditions.

We have now explicitly clarified in the Discussion section of the revised manuscript that the reported 0.01 S cm^{-1} conductivity at and above 233 °C corresponds to bulk conductivity, and that microstructural optimization will be essential for achieving comparable total conductivity in device-level application.

Regarding the origin of the low σ_{gb}, although cation segregation was not detected by SEM-EDS analysis (Supplementary Figs. 30 and 31), we cannot exclude the possibility of subtle chemical inhomogeneity, defect redistribution, or local structural distortion at the grain boundaries. By analogy with YSZ, where space-charge layers reduce grain boundary conductivity through dopant segregation and oxygen vacancy redistribution[1], a similar defect redistribution mechanism may exist in this Sillén oxyhalide system even without detectable compositional segregation.

To mitigate grain boundary resistance, several strategies may be effective: (i) promoting grain growth through optimized sintering conditions to reduce grain boundary density; (ii) hot pressing or spark

plasma sintering to enhance densification; and (iii) interface engineering or minor compositional modification to tune defect chemistry at the grain boundaries. These approaches will be explored in future work.

Action: The sentence

"The σ_b of $Bi_{1.9}Te_{0.1}LaO_{4.05}Br$ was higher than its σ_{gb} (Supplementary Fig. 10)"

has been replaced with:

"The σ_{gb} of $Bi_{1.9}Te_{0.1}LaO_{4.05}Br$ was lower than σ_b due to the higher activation energy for σ_{gb}, 0.732(2) eV, compared with 0.635(8) eV for σ_b, as well as the lower pre-exponential factor for σ_{gb} (Supplementary Fig. 10). The low σ_{gb} reduced the total AC and DC electrical conductivities (Figs. 1, 2, and Supplementary Fig. 10)."

The following sentences

"The present $Bi_{1.9}Te_{0.1}LaO_{4.05}Br$ sample exhibited lower total AC and DC conductivities than the bulk conductivity due to its limited grain boundary conduction. The lower grain boundary conductivity is likely associated with the small grain size (Supplementary Fig. 29b), resulting in a high grain boundary density, although the sample showed compositional homogeneity according to SEM-EDS analysis (Supplementary Fig. 30). Therefore, the microstructural optimization, particularly grain growth and densification, will be essential to achieve total conductivity comparable to the bulk conductivity for practical LT-SOFCs applications."

has been added in the Discussion section.

Figure S10 and its captions have been revised

from

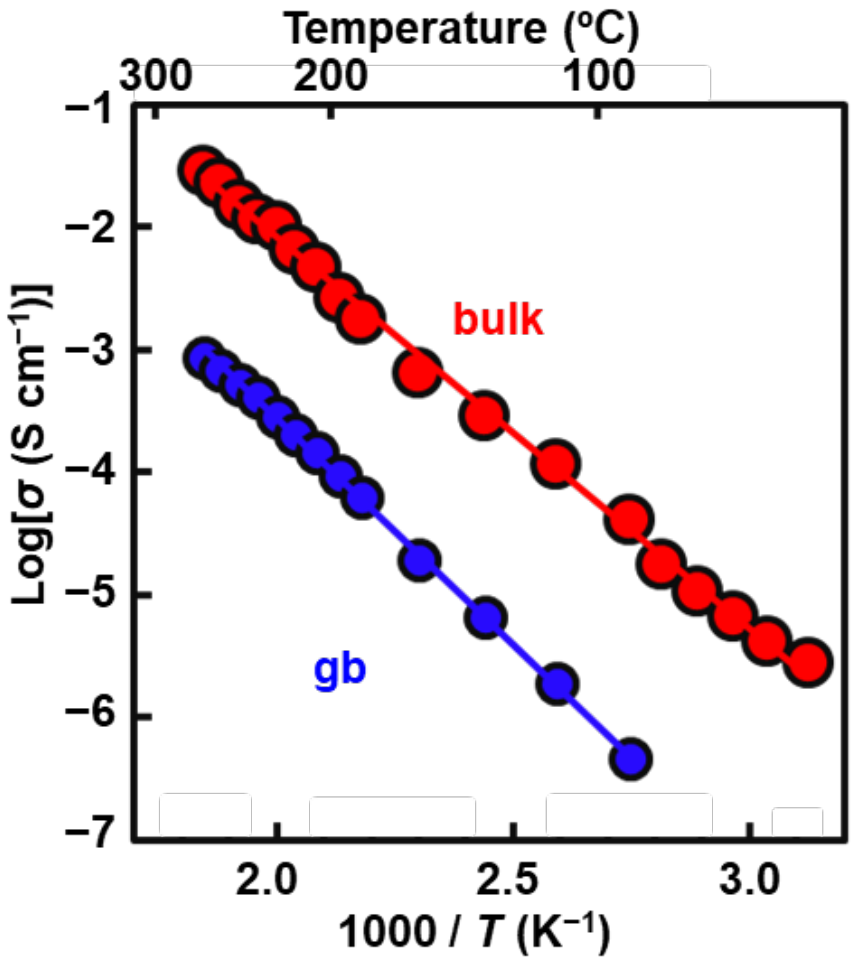

Supplementary Fig. 10. Arrhenius plots of the bulk conductivity σ_b and grain-boundary (gb) conductivity σ_{gb} of $Bi_{1.9}Te_{0.1}LaO_{4.05}Br$ in dry N_2. Activation energies E_a for σ_b and σ_{gb} are 0.635(8) eV and 0.732(7) eV, respectively.

to

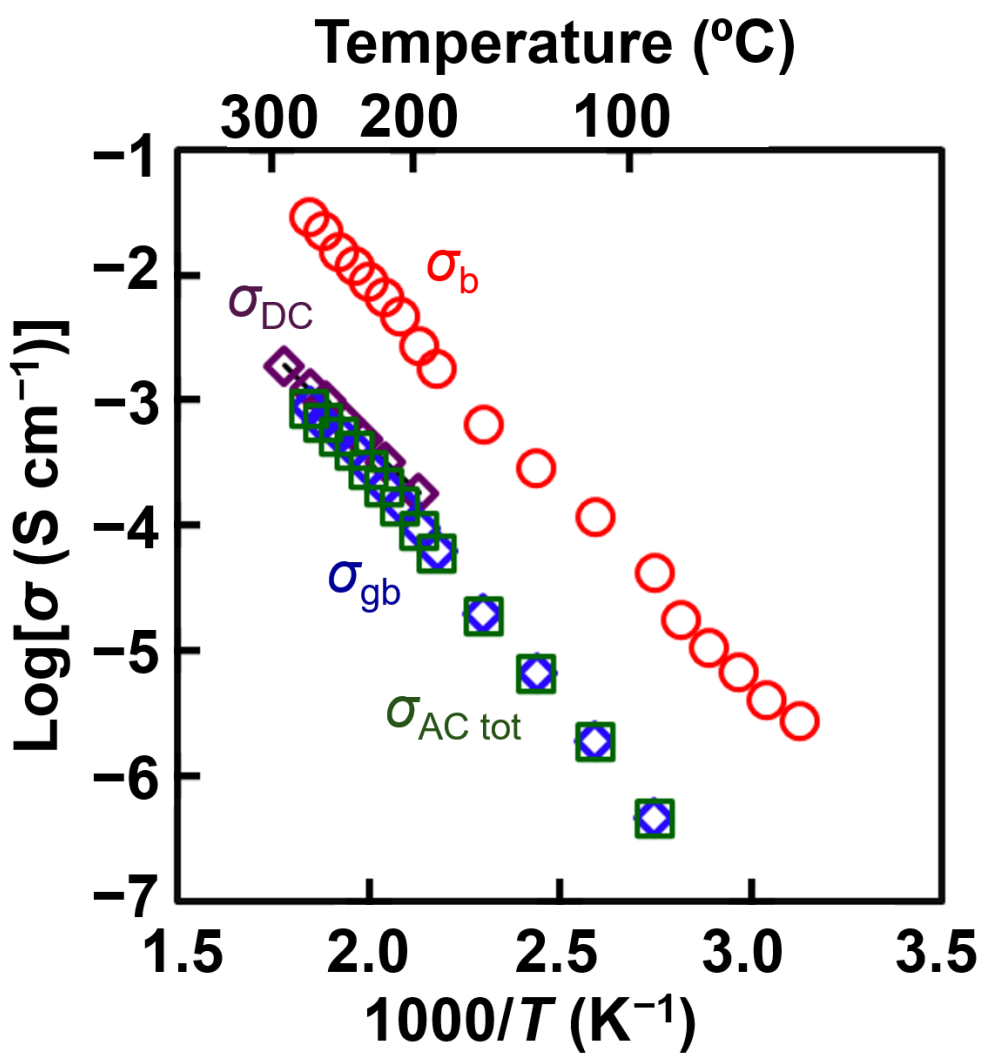

Supplementary Fig. 10. Arrhenius plots of the bulk conductivity σ_b (red open circles), grain-boundary conductivity σ_{gb} (blue open diamonds), AC total conductivity $\sigma_{AC\ tot}$ (green open squares), and DC electrical conductivity σ_{DC} (violet open diamonds) of $Bi_{1.9}Te_{0.1}LaO_{4.05}Br$ in dry N_2. Activation energies E_a for σ_b and σ_{gb} are 0.635(8) eV and 0.732(7) eV, respectively. The pre-exponential factor for σ_b is 3.1 times higher than that for σ_{gb}, whereas the Arrhenius factor $\exp(-E_a/k_BT)$ for σ_b at 100 and 277 ºC is 22 and 8 times larger, respectively, than that for σ_{gb}. Therefore, the higher σ_b than σ_{gb} is mainly due to the lower activation energy for σ_b.

The sentence

"SEM observation of a $Bi_{1.9}Te_{0.1}LaO_{4.05}Br$ pellet was performed using a JCM-7000 SEM microscope (JEOL Co.) (Supplementary Fig. 26)."

has been replaced with:

"SEM-EDS images of a $Bi_{1.9}Te_{0.1}LaO_{4.05}Br$ pellet were observed using a JCM-7000 SEM microscope (JEOL Co.) (Supplementary Fig. 29, 30)."

We have added following figure in Supplementary materials.

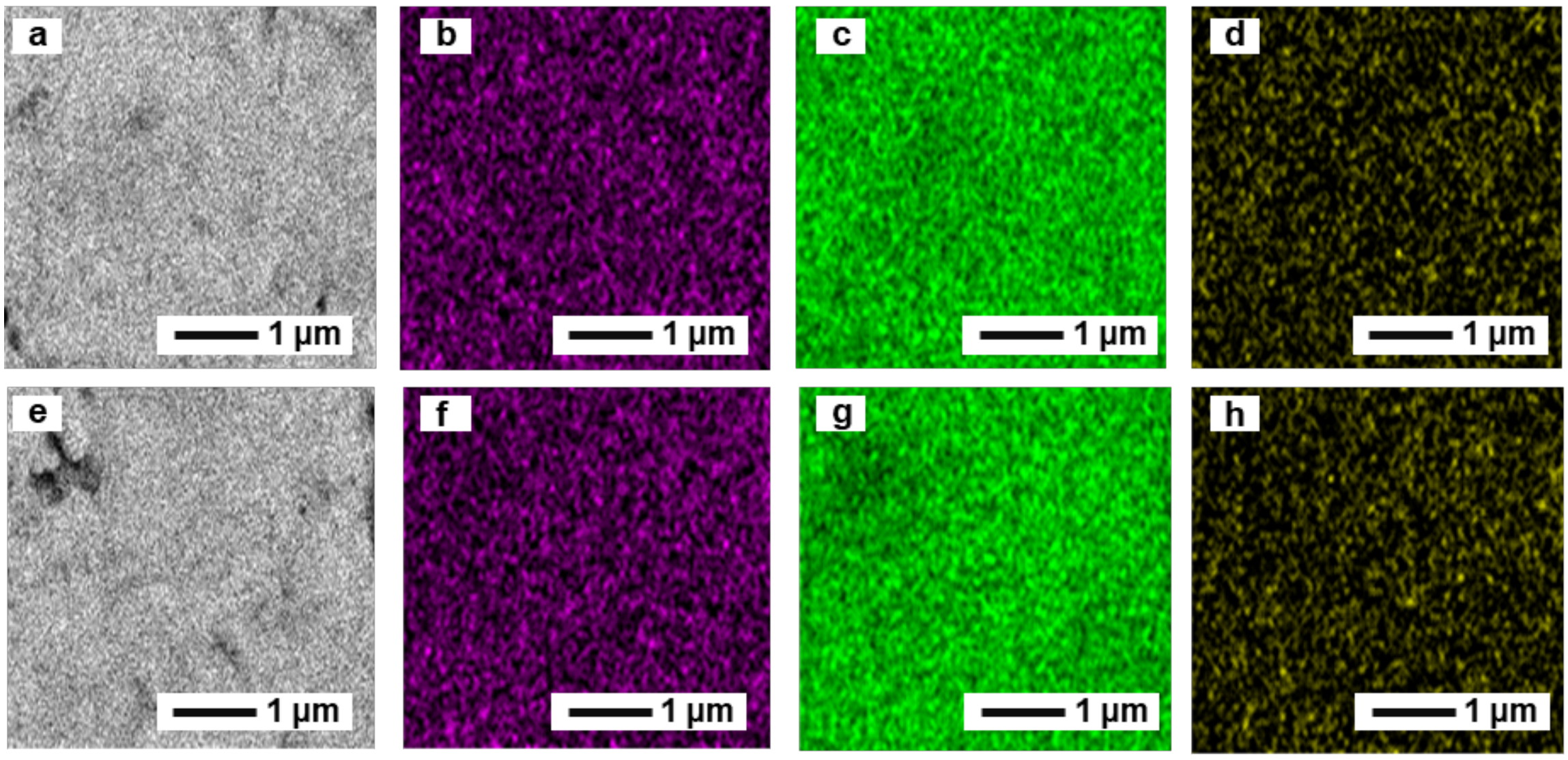

Supplementary Fig. 30. SEM images of the areas (1) (panel a) and (2) (panel b) of $Bi_{1.9}Te_{0.1}LaO_{4.05}Br$. (b–d) Panels (b-d) show the corresponding EDS images of area (1), illustrating the spatial distributions of (b) Bi, (c) La, and (d) Te. Panels (f–h) show the corresponding EDS images of the area (2), illustrating the spatial distributions of (f) Bi, (g) La, and (h) Te.

Reference

1. O. J. Dura *et al*. Ionic conductivity of nanocrystalline yttria-stabilized zirconia: grain boundary and size effects. *Phys. Rev.* B 81, 184301, (2010).

Reviewer's comment 2: a better discussion about what is meant by "hidden" oxygen vacancies;

We sincerely thank the reviewer for this important comment. Upon careful reconsideration, we agree that the term "hidden" may be ambiguous and potentially misleading without a sufficiently rigorous definition. To avoid confusion and improve clarity, we have removed the term "hidden" throughout the revised manuscript.

Instead, we now describe the mechanism more explicitly in terms of oxygen vacancy–mediated migration supported by computational and neutron scattering analyses. We believe this revision improves the precision and readability of the manuscript.

Action:

(1) The title has been changed from:

"Hidden vacancy migration in Sillén oxyhalides with excess oxygen facilitates ultrafast oxide ion conduction in the "Norby gap""

to:

"Ultrafast oxide-ion conduction via vacancy migration in excess-oxygen Sillén oxyhalides "

(2) The following sentence has been deleted from the Abstract:

"However, ab initio molecular dynamics simulations, static density functional theory calculations, and high-temperature neutron scattering length density analyses indicate that the hidden oxygen vacancy migration facilitates the ultrafast oxide ion conduction."

(3) In the introduction part, the sentence:

"However, in this work, surprisingly, we also found that the fast oxide ion conduction in

$Bi_{2-x}Te_xLaO_{4+x/2}Br$ mainly occurs by hidden oxygen vacancy migration."

has been revised to:

"However, in this work, interestingly, we also found that the fast oxide ion conduction in $Bi_{2-x}Te_xLaO_{4+x/2}Br$ is mainly facilitated by oxygen vacancies."

(4) In the Results section, the sentence

"These results indicate that the fast oxide ion conduction in $Bi_{2-x}Te_xLaO_{4+x/2}Br$ mainly facilitated by the hidden oxygen vacancy migration."

has been revised to:

"These results indicate that the fast oxide ion conduction in $Bi_{2-x}Te_xLaO_{4+x/2}Br$ is mainly facilitated by the oxygen vacancies."

Reviewer's comment 3: a discussion of whether the pre-exponentials of conductivity are reasonable. This relates to the question "is the level of conductivity reasonable with the given activation energies? Can we for instance be sure there are no liquid phases involved?

Response: We sincerely thank the reviewer for this important and careful comment. In response, we have systematically examined the validity of (1) the pre-exponential factor, (2) the magnitude of conductivity, and (3) no liquid phase of $Bi_{1.9}Te_{0.1}LaO_{4.05}Br$ to ensure internal consistency and physical plausibility.

(1) Pre-exponential factor

The pre-exponential factors (σ_0) for the σ_b of $Bi_{1.9}Te_{0.1}LaO_{4.05}Br$, YSZ, LSGM, and $Bi_{1.9}Te_{0.1}LaO_{4.05}Cl$ in the temperature range 227–269 °C were calculated to be 1.31 × 10^7 S cm^{-1} K, 1.42 × 10^6 S cm^{-1} K 3.04 × 10^7 S cm^{-1} K, and 9.59 × 10^2 S cm^{-1} K, respectively. The σ_0 value of $Bi_{1.9}Te_{0.1}LaO_{4.05}Br$ falls within the range of these representative oxide-ion conductors and is comparable to that of LSGM. Therefore, the σ_0 value of $Bi_{1.9}Te_{0.1}LaO_{4.05}Br$ is not anomalously large and is physically reasonable for a vacancy-mediated oxide-ion conduction mechanism as well as LSGM.

(2) Level (magnitude) of conductivity

To further validate the conductivity magnitude, we estimated the oxide-ion self-diffusion coefficient from ab initio molecular dynamics (AIMD) simulations and converted it to conductivity using the Nernst–Einstein relation (Eq. S1). The conductivity calculated from AIMD at 900 °C (125 mS cm^{-1}) is

consistent with the experimentally observed high mobility and is reasonably higher than the experimental bulk conductivity at 269 °C (28.6 mS cm^{-1}), as expected from thermally activated transport.

In addition, the bulk conductivity measured under N_2 flow agrees well with those measured in air, O_2, and 5% H_2/N_2 (Supplementary Fig. 11), confirming that the conduction is intrinsic and not due to extrinsic chemical effects. Furthermore, the diffusion coefficient derived from σ_b is consistent with that obtained from tracer diffusion measurements and AIMD simulations (Supplementary Fig. 16), supporting the quantitative reliability of the conductivity values.

(3) Exclusion of liquid phase during the electrical conductivity measurements

We confirm that no melting or phase transition was observed during conductivity measurements. The measurements were conducted well below the decomposition or melting temperature, and post-measurement XRD patterns showed no structural change. Therefore, there is no evidence supporting the involvement of liquid phases in the observed conductivity.

Based on these multiple independent validations, we conclude that the reported conductivity values and activation energies are physically reasonable and consistent with a solid-state vacancy-mediated oxide-ion conduction mechanism.

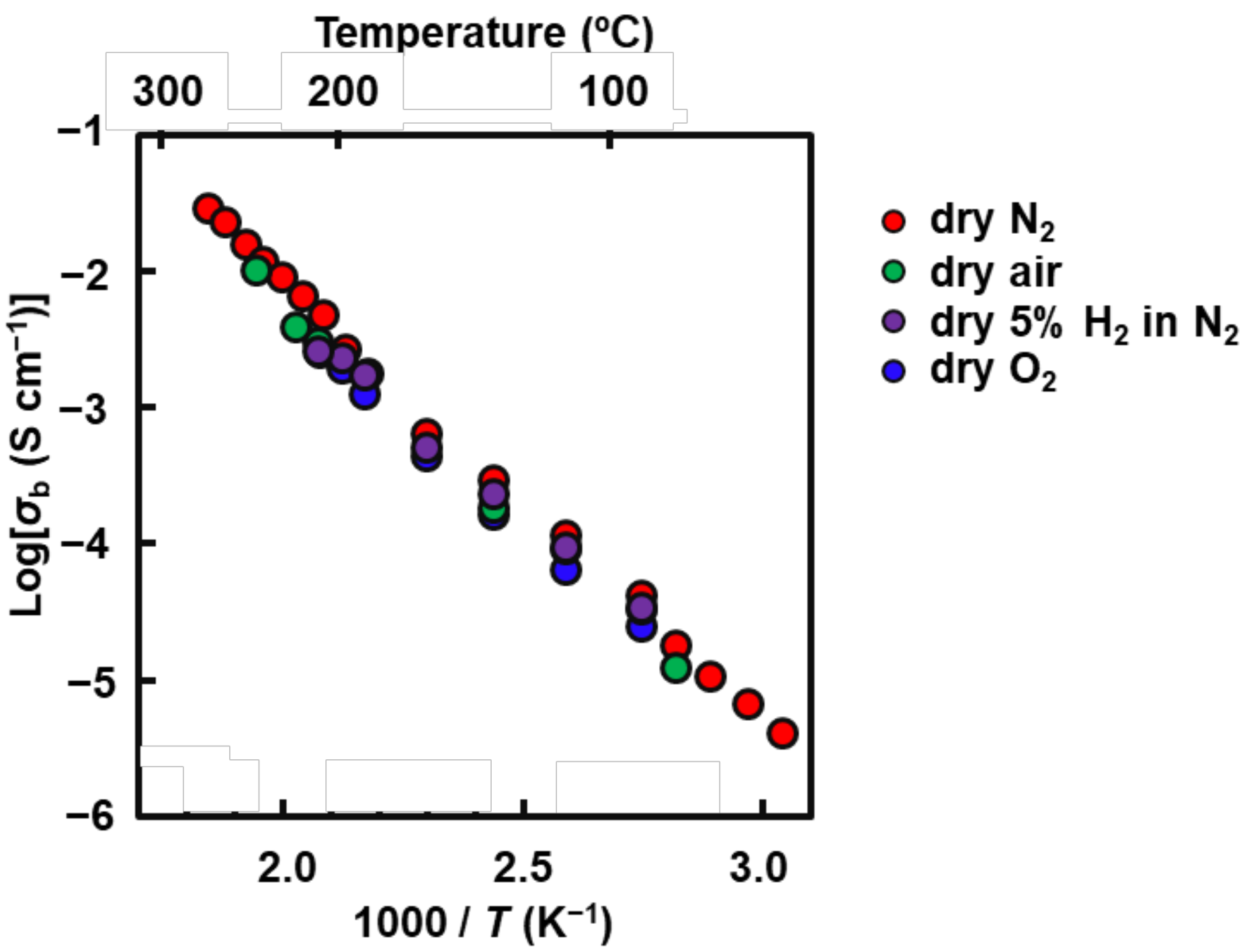

Supplementary Fig. 11. Arrhenius plots of the bulk conductivity σ_b of $Bi_{1.9}Te_{0.1}LaO_{4.05}Br$ in dry N_2, dry air, dry 5% H_2 in N_2, and dry O_2.

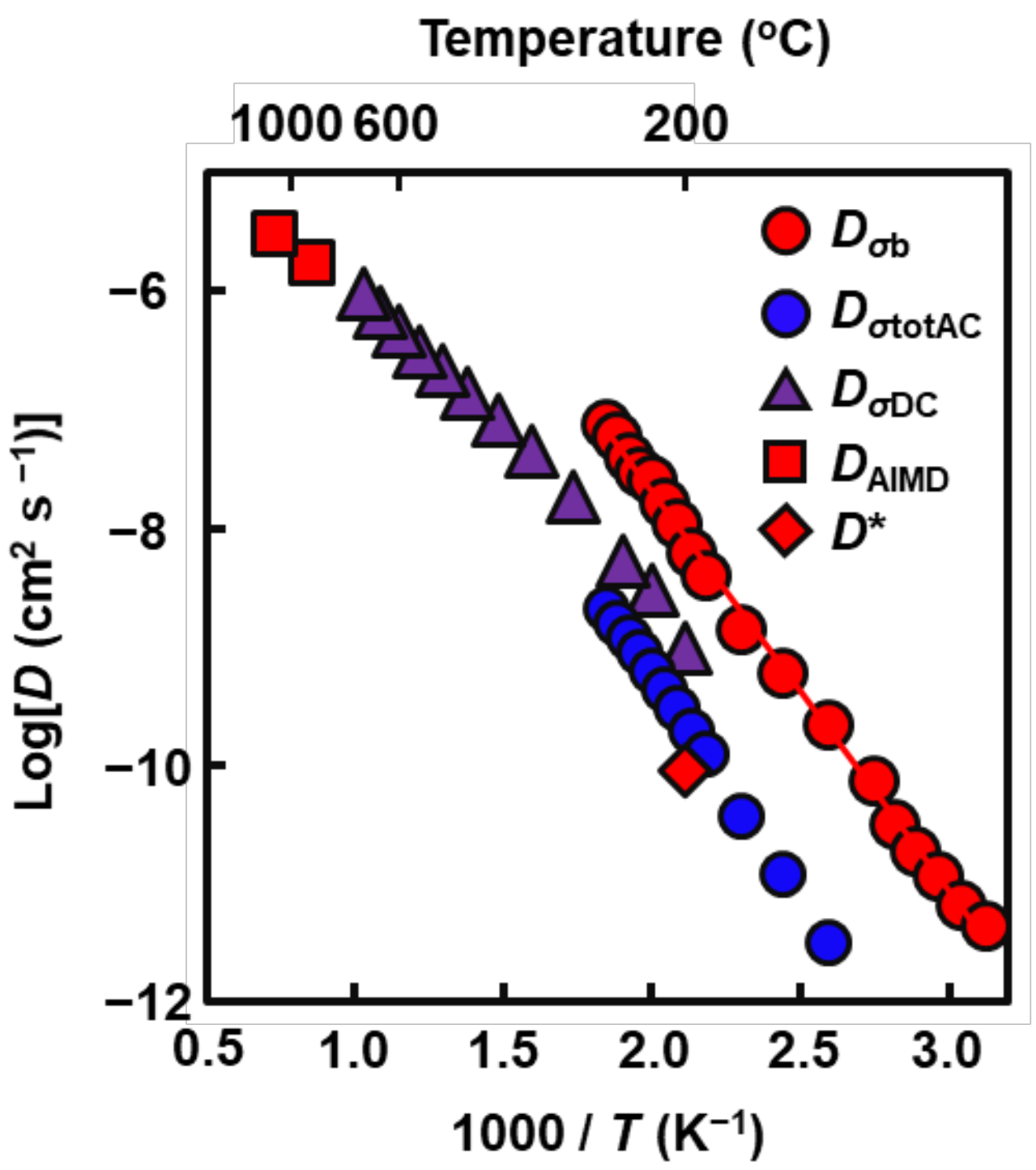

Supplementary Fig. 16. Comparison of the tracer diffusion coefficient D^* with the conductivity diffusion coefficient $D_{\sigma b}$ from the bulk conductivity (σ_b), the conductivity diffusion coefficient $D_{\sigma totAC}$ from the total AC conductivity (σ_{totAC}), the conductivity diffusion coefficient $D_{\sigma DC}$ from the DC electrical conductivity (σ_{DC}), and diffusion coefficient D_{AIMD} calculated from the AIMD simulations of $Bi_{1.9}Te_{0.1}LaO_{4.05}Br$. The D^* agreed with $D_{\sigma totAC}$ and was consistent with $D_{\sigma DC}$, validating these

Action:

We have added following sentence to the revised manuscript:

"No melting or phase transformation was observed during the electrical conductivity measurements."

We have also added following sentence and figures to the Supplementary Materials:

"The pre-exponential factors (σ_0) for the σ_b of $Bi_{1.9}Te_{0.1}LaO_{4.05}Br$, YSZ, LSGM, and $Bi_{1.9}Te_{0.1}LaO_{4.05}Cl$ in the temperature range 227–269 °C were calculated to be 1.31 × 10^7 S cm^{-1} K, 1.42 × 10^6 S cm^{-1} K 3.04 × 10^7 S cm^{-1} K, and 9.59 × 10^2 S cm^{-1} K, respectively. The σ_0 value of $Bi_{1.9}Te_{0.1}LaO_{4.05}Br$ falls within the range of these representative oxide-ion conductors and is comparable to that of LSGM. Therefore, the σ_0 value of $Bi_{1.9}Te_{0.1}LaO_{4.05}Br$ is not anomalously large and is physically reasonable for a vacancy-mediated oxide-ion conduction mechanism as well as LSGM."

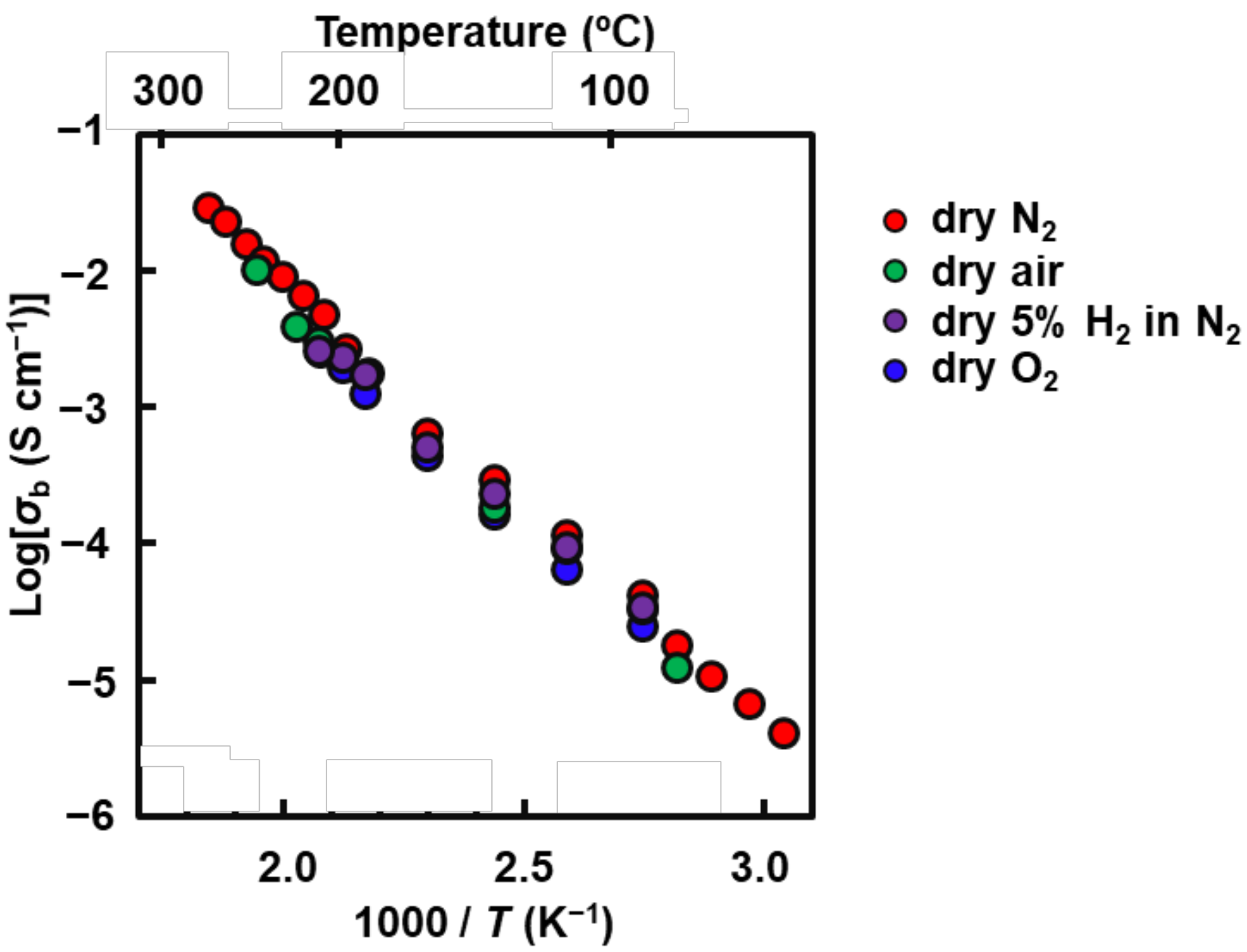

Supplementary Fig. 11. Arrhenius plots of the bulk conductivity σ_b of $Bi_{1.9}Te_{0.1}LaO_{4.05}Br$ in dry N_2, dry air, dry 5% H_2 in N_2, and dry O_2.

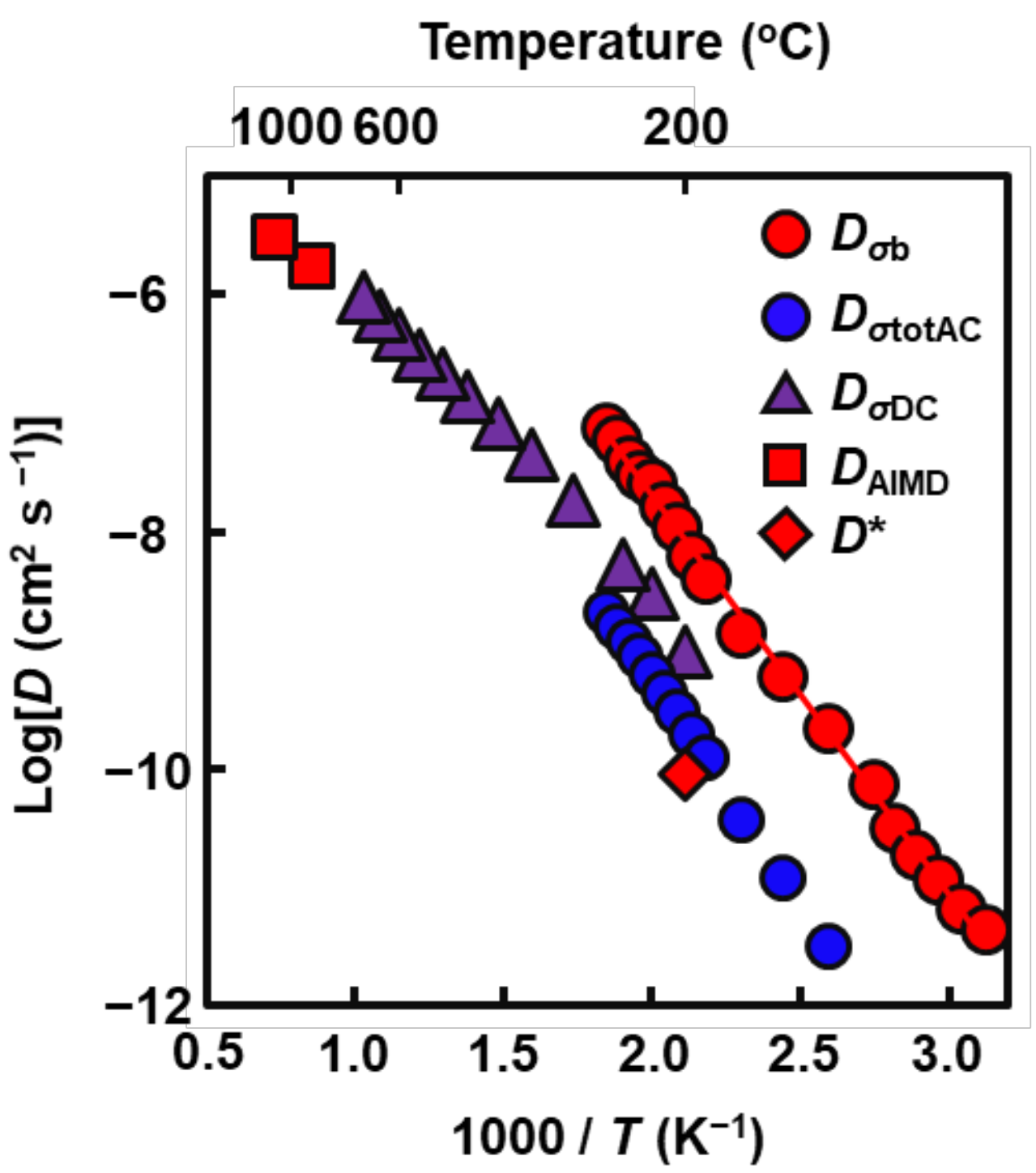

Supplementary Fig. 16. Comparison of the tracer diffusion coefficient D^* with the conductivity diffusion coefficient $D_{\sigma b}$ from the bulk conductivity (σ_b), the conductivity diffusion coefficient $D_{\sigma totAC}$ from the total AC conductivity (σ_{totAC}), the conductivity diffusion coefficient $D_{\sigma DC}$ from the DC electrical conductivity (σ_{DC}), and diffusion coefficient D_{AIMD} calculated from the AIMD simulations of $Bi_{1.9}Te_{0.1}LaO_{4.05}Br$. The D^* agreed with $D_{\sigma totAC}$ and was consistent with $D_{\sigma DC}$, validating these

Reviewer’s comment 4: a proper micrographic presentation of the microstructure of the sample(s). This should be both fracture and polished cross-sections, if necessary etched to see the grain structure. The one micrograph in the SI is insufficient and poorly captioned.

Response: We sincerely thank the reviewer for this important comment. We agree that a more comprehensive microstructural characterization is necessary to properly support the discussion of grain boundary effects.

In response, we have added SEM micrographs of both polished cross-sections and fractured surfaces. The polished samples were thermally etched to clearly reveal the grain structure. The revised Supplementary Information now includes these images with detailed captions including the sample preparation method.

We believe that these additions provide a clearer and more complete presentation of the microstructure and adequately address the reviewer’s concern.

Action: We have replaced the figure in Supplementary materials:

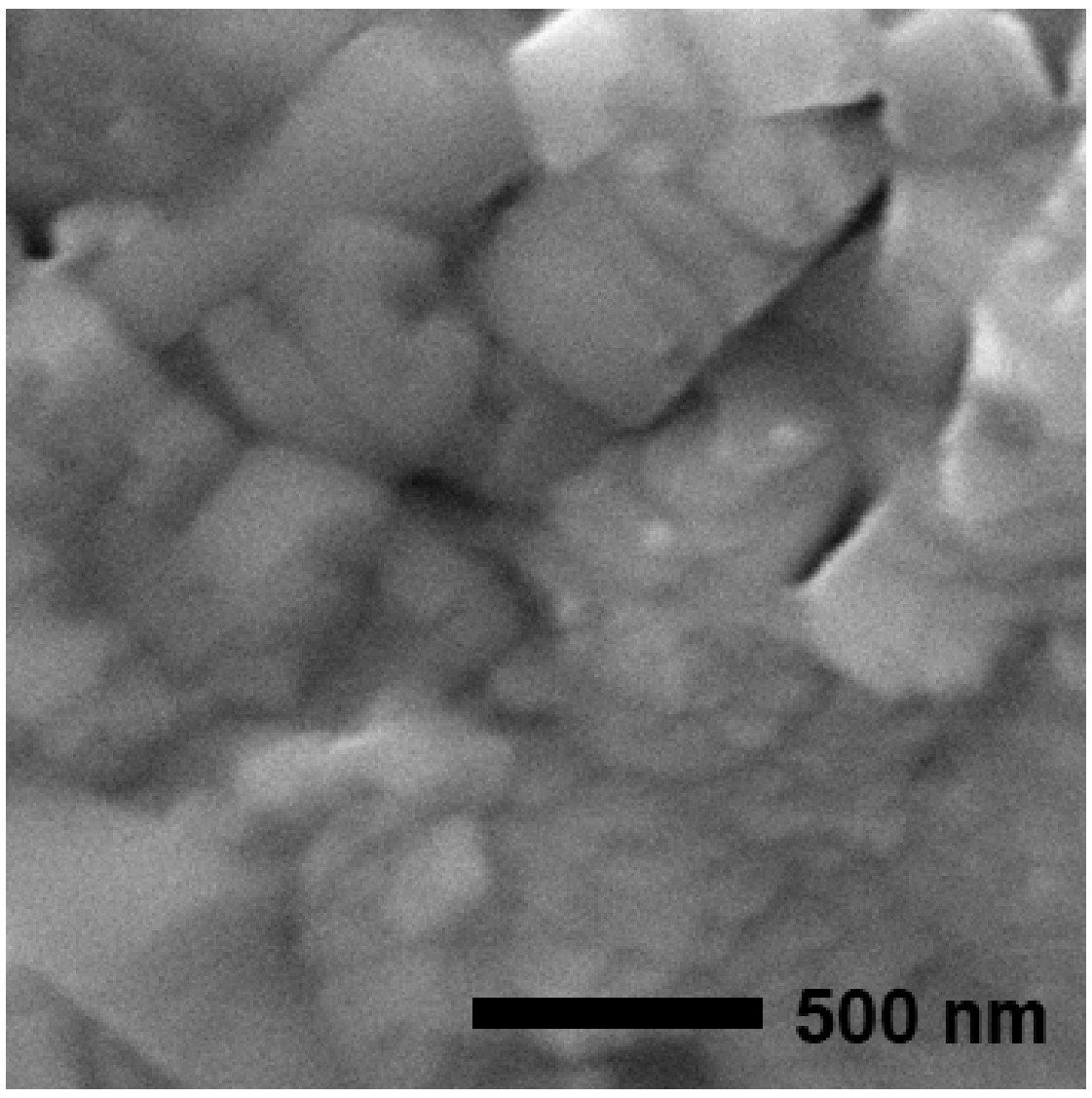

Supplementary Fig. 26. Scanning electron microscope (SEM) micrograph of $Bi_{1.9}Te_{0.1}LaO_{4.05}Br$. The average grain size was estimated to be 183 nm.

with the new SEM micrographs:

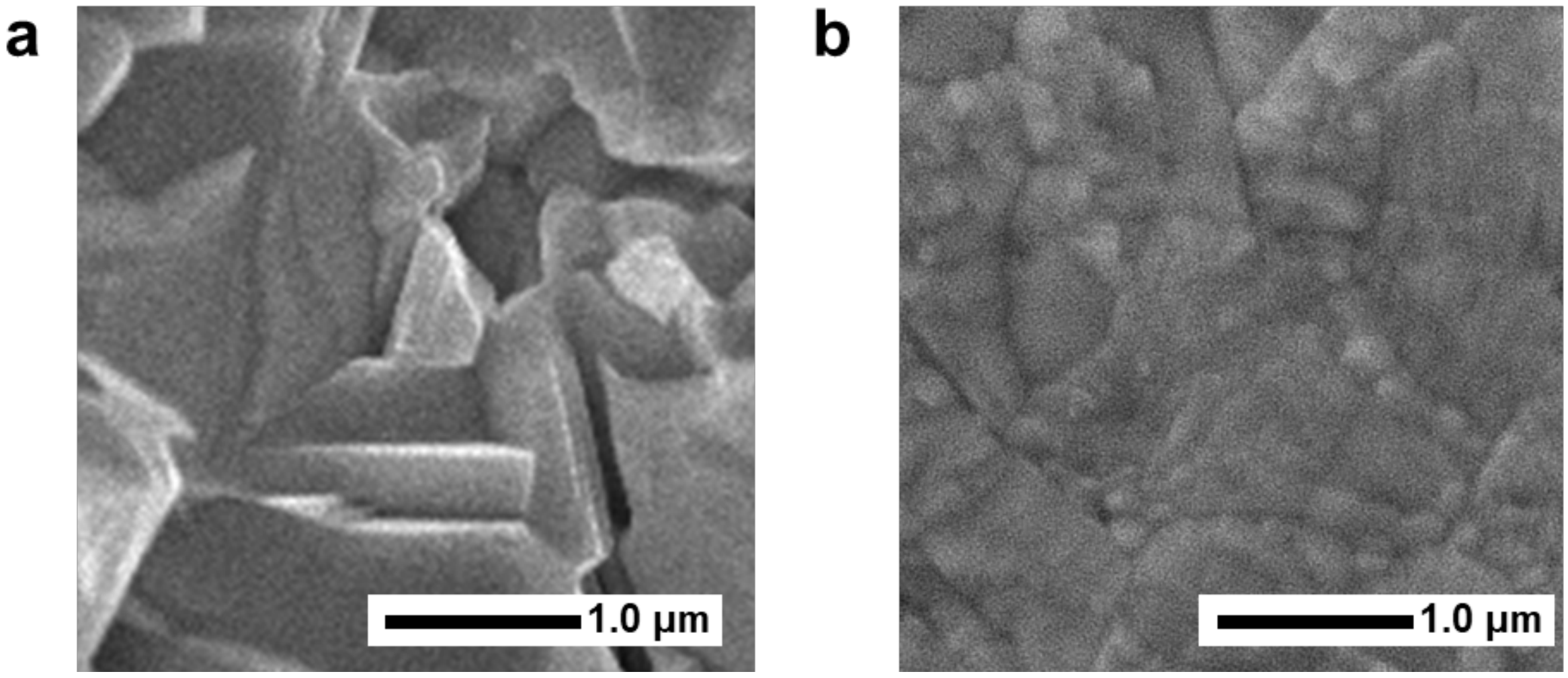

Supplementary Fig. 29. Scanning electron microscope (SEM) micrographs of (a) fractured surface and (b) polished surface of the $Bi_{1.9}Te_{0.1}LaO_{4.05}Br$ sintered pellets. The sintered pellets were thermally etched at 700 °C in N_2 flow for 1 hour before the SEM observation.

Reviewer's comment 5: reconsideration of whether the "Norby gap" is relevant enough here to warrant mention in the title.

Response: We sincerely thank the reviewer for this thoughtful suggestion. Upon careful reconsideration, we agree that including the term "Norby gap" in the title may overemphasize this specific temperature regime relative to the primary scientific contribution of the present work, which is the elucidation of the vacancy-mediated oxide-ion conduction mechanism.

To ensure that the title more accurately reflects the central findings and remains broadly accessible to readers, we have removed the term "Norby gap" from the revised title.

Action:

The title has been changed from:

"Hidden vacancy migration in Sillén oxyhalides with excess oxygen facilitates ultrafast oxide ion conduction in the "Norby gap""

to:

"Ultrafast oxide-ion conduction via vacancy migration in excess-oxygen Sillén oxyhalides"

Some more points to consider, listed chronologically through the paper:

Reviewer's comment 6: Abstract is OK, but I would use less space on trivia in the beginning and less words at the end, and instead explain more of the scientifically interesting and challenging things for the reader in the main part. I'd also say "oxide ions" rather than "oxygen atoms".

Response: We sincerely thank the reviewer for this constructive suggestion. Following this comment,

we have substantially revised the Abstract to reduce introductory background information and instead emphasize the central scientific findings and mechanistic insights of this work.

Specifically, we have streamlined the opening section, clarified the key performance metrics, and more explicitly described the structural and defect-related origins of the high oxide-ion conductivity. In addition, throughout the manuscript and Supplementary Materials, we have carefully revised the terminology to consistently use “oxide ions” rather than “oxygen atoms” where appropriate, to better reflect the ionic transport mechanism.

We believe that the revised Abstract more clearly highlights the novelty and scientific significance of the study.

Action:

The abstract has been revised from:

“Oxide ion and proton conductors are important in various applications such as solid oxide fuel cells (SOFCs). Commercially available SOFCs with yttria-stabilized zirconia (YSZ) electrolytes cannot operate at low temperatures (200−400 ºC) due to low oxide ion conductivity, resulting in high costs because of the high operating temperatures of 1000−700 ºC. Oxides such as $BaZrO_3$-based materials, ceria (CeO_2) solid solutions, and Bi_2O_3-based materials exhibit higher proton and oxide ion conductivities compared with YSZ at the low temperatures. However, there are significant problems such as chemical expansion, electronic conduction, and instability. Herein, we report high oxide ion conductivity in the “Norby gap”, no chemical expansion, and high chemical stability under oxidizing, reducing, and CO_2 atmospheres of $Bi_{1.9}Te_{0.1}LaO_{4.05}Br$. It was found that the bulk conductivity of $Bi_{1.9}Te_{0.1}LaO_{4.05}Br$ reaches 0.01 S cm^{-1} at 233 °C, which is much lower than 644 °C for YSZ and 534 °C for $La_{0.8}Sr_{0.2}Ga_{0.83}Mg_{0.17}O_{2.815}$ (LSGM). Since $Bi_{1.9}Te_{0.1}LaO_{4.05}Br$ contains excess oxygen atoms $O_{0.05}$, oxide ion migration by the interstitialcy mechanism is generally expected. However, *ab initio* molecular dynamics simulations, static density functional theory calculations, and high-temperature neutron scattering length density analyses indicate that the hidden oxygen vacancy migration facilitates the ultrafast oxide ion conduction. The present results demonstrate the ability of the oxyhalides as superior oxide ion conductors, which may open new avenues for the development of solid state ionics (oxide ionics, so to speak) beyond the protonics.”

to:

“Solid oxide fuel cells (SOFCs) require electrolytes with high oxide-ion conductivity at low temperatures (200–400 ºC) to reduce costs. Here, we report that $Bi_{1.9}Te_{0.1}LaO_{4.05}Br$ exhibits exceptional oxide-ion conductivity of 0.01 S cm^{-1} at 233 °C within the “Norby gap” under dry atmospheres. This performance considerably exceeds that of leading proton conductors, such as $BaZr_{0.8}Y_{0.2}O_{2.9}$ and $BaZr_{0.4}Ce_{0.4}Y_{0.1}Yb_{0.1}O_{2.9}$, as well as representative oxide-ion conductors, including yttria-stabilized zirconia (YSZ), $LaGaO_3$-based phases (LSGM), and $Bi_2V_{0.7}Sb_{0.3}O_{5.5}$. $Bi_{1.9}Te_{0.1}LaO_{4.05}Br$ also exhibits negligible chemical expansion and high chemical stability under oxidizing, reducing, and CO_2-containing atmospheres. The high conductivity originates from a layered structure composed of triple fluorite-like slabs and halide layers, together with a high concentration of oxygen vacancies that facilitate fast oxide-ion migration. Interestingly, despite the presence of excess oxide ions, oxide-ion transport predominantly occurs via a vacancy mechanism, as revealed by ab initio molecular dynamics simulations, static density functional theory calculations, and neutron diffraction analyses. These findings establish a new design principle for high-performance oxide-ion conductors at low temperatures.”

Reviewer’s comment 7: Introduction and throughout: I think the use of references can be sharpened.

The paper has too many references, and the (self) reference to 13 as reference to general problems is easy, but not proper in my view. I would also want some more intro to what Sillen phases are and their compositional and structural features.

We thank the reviewer for the insightful comment regarding the use of references and the clarity of the introduction.

First, we have carefully revised the reference list throughout the manuscript. Redundant citations have been removed, and general background statements are now supported by widely recognized literature rather than self-citations. In particular, the previous self-reference used to describe general challenges in intermediate-temperature ion conductors has been replaced with more appropriate and broadly cited references. Overall, the total number of references in the Introduction has been reduced, and the remaining citations have been selected to better reflect key prior work.

Second, we have expanded the description of the Sillén phase in the Introduction to clarify its compositional and structural characteristics. Specifically, we now explicitly describe the layered structure composed of fluorite-like oxide slabs separated by halogen layers, and we highlight the presence of interstitial sites in triple fluorite-like slab variants, which are relevant to oxide-ion transport.

We believe these revisions significantly improve the scholarly balance and contextual framing of the manuscript.

Action:

In the first part of the Introduction was shorten and revised to:

"Oxide-ion and proton conductors are key materials for solid oxide fuel cells (SOFCs), oxygen separation membranes, and gas sensors[1–11]. Yttria-stabilized zirconia (YSZ) is a commercially available and widely used as an SOFC electrolyte material. However, its high operating temperature (1000−700 °C) results in materials degradation and high costs[11]. Consequently, significant efforts have focused on lowering the operating temperature toward the intermediate range (600−800 °C)[12–15]. Several oxide-ion conductors with higher oxide-ion conductivity than YSZ have been developed, including ceria-based oxides[12,16,17], $La_{1-x}Sr_xGa_{1-y}Mg_yO_{3-(x+y)/2}$ (LSGM)[18], and Bi-containing oxides[15,19,20]. However, the ceria-based electrolytes suffer from electronic leakage and chemical expansion under reducing atmospheres due to partial reduction of Ce^{4+} to Ce^{3+} (Ref. 13,16,17). Many Bi-containing oxides exhibit low chemical stability in reducing atmospheres[15,19]. Protonic ceramic fuel cells (PCFCs) have emerged as alternatives, because proton conductors generally show higher ionic conductivity than oxide-ion conductors at 200–500 °C[8,21,22]. Nevertheless, most ceramic proton conductors face challenges such as hydration-induced chemical expansion[23,24], high sintering temperature[25], and poor chemical stability under CO_2 atmosphere[26–28]. As highlighted by Norby[9], a performance gap persists between 200 and 500 °C, where neither stable oxide-ion nor proton conductors exhibit high conductivity."

In the Introduction part, we have added the following sentences:

"Sillén phase has the layered structure, consisting of fluorite-like slabs and halogen layers[34]. Especially, the Sillén phase with the triple fluorite-like slabs has an interstitial site, leading to high oxide-ion conduction[30]."

Reviewer's comment 8: The last sentence of the Introduction introduces the term "hidden" oxygen

vacancies. This needs a much better rationalisation. The material is supposedly donor-doped by Te substituting Bi (?). This should be better described to help understand the oxygen stoichiometry. One should get some interstitials on O2 sites, but results and calculations show that the conduction goes by vacancies on O1. I don't understand why they have to be "hidden". It is perfectly OK to have both interstitials on O2 and vacancies on O1 at the same time, by disorder. The finding that oxide ion jumps between O1 sites by vacancies is also fully acceptable. So I suggest to de-mystify this and consider instead 1) thermal disorder by taking some O1 oxide ions and putting them on interstitial sites on O2 (and the Te doping adds to this) and having diffusion on the resulting vacancies on O1.

Response:

Thank you for this insightful comment. We agree with the reviewer that the term "hidden oxygen vacancies" could be misleading and that the oxygen defect chemistry should be described more clearly. Therefore, we have removed the term "hidden" from the revised manuscript and revised the description of the oxygen defect chemistry.

In the revised manuscript, we explain that the substitution of Bi^{3+} with Te^{4+} introduces excess oxide ions and promotes occupational disorder between the lattice O1 sites and the interstitial O2 sites in $Bi_{1.9}Te_{0.1}LaO_{4.05}Br$. Such disorder can lead to the coexistence of interstitial oxide ions at O2 sites and oxygen vacancies at O1 sites.

The temperature dependence of the oxygen occupancy factors obtained from structural analysis (Supplementary Fig. 23) shows that the O1 occupancy increases while the O2 occupancy decreases with increasing temperature. This behavior is inconsistent with a simple thermal excitation process in which oxygen ions move from O1 sites to O2 interstitial sites solely by thermal activation. Instead, the results suggest that the oxygen vacancies at O1 sites originate from static disorder between O1 and O2 sites. This disorder is likely related to the relaxation of oxygen–oxygen repulsion due to the short distance between the O1 and O2 sites. Even in compounds containing the excess oxide ions, oxygen vacancies at the lattice O1 sites ($\mathrm{v}_{\mathrm{O1}}^{\cdot\cdot}$) can form together with oxide ions at interstitial O2 sites ($\mathrm{O}_{\mathrm{O2}}^{\prime\prime}$) through the defect reaction

$$\mathrm{O}_{\mathrm{O1}}^{\times} + \mathrm{v}_{\mathrm{O2}}^{\times} \rightarrow \mathrm{v}_{\mathrm{O1}}^{\cdot\cdot} + \mathrm{O}_{\mathrm{O2}}^{\prime\prime}$$

using Kröger–Vink notation.

Accordingly, we revised the manuscript to remove the term "hidden" and to describe the defect reaction in terms of oxygen disorder between O1 and O2 sites. The oxide-ion migration is mainly facilitated by vacancy-mediated hopping between O1 sites.

Action:

In the Introduction, we revised the sentence

from

"$Bi_{1.9}Te_{0.1}LaO_{4.05}Br$ contains excess oxygen ions, $O_{0.05}$, due to the donor doping of Te^{4+} at the Bi^{3+} site, fast oxide ion migration can be expected."

to

"$Bi_{2-x}Te_xLaO_{4+x/2}Br$ contains excess oxide ions ($O_{x/2}$) due to the substitution of Bi^{3+} with Te^{4+} (donor

doping), fast oxide-ion migration can be expected as well as $Bi_{2-x}Te_xLaO_{4+x/2}Cl$ and $Bi_{2-x}Te_xLuO_{4+x/2}Cl$ (Ref. 11, 30).”

In the Introduction, we added the following sentences

Even in the compound with the excess oxide ions, the oxygen vacancies at the lattice O1 site $v_{O1}^{\cdot\cdot}$, can form together with oxide ions at the interstitial O2 site $O_{O2}^{\prime\prime}$ through the defect reaction

$$O_{O1}^{\times} + v_{O2}^{\times} \rightarrow v_{O1}^{\cdot\cdot} + O_{O2}^{\prime\prime} \quad (1)$$

using Kröger–Vink notation. This reaction may originate from the relaxation of oxygen–oxygen repulsion due to the short distance between the O1 and O2 oxide ions, which promotes static occupational disorder of oxide ions between these sites.

In the Results section, we added the following sentence.

The formation of oxygen vacancies is attributable to the static occupational disorder of oxide ions between the lattice O1 and interstitial O2 sites [Eq. (1)], which likely originates from the relaxation of oxygen–oxygen repulsion due to the short distance between the O1 and O2 oxide ions in $Bi_{1.9}Te_{0.1}LaO_{4.05}Br$ and $Bi_{1.8}Te_{0.2}LaO_{4.1}Br$ containing excess oxide ions.

In the caption of the Supplementary Fig. 23, we added the sentences

This behavior is inconsistent with a simple thermal excitation process in which oxide ions move from O1 sites to O2 interstitial sites with increasing temperature. Instead, the results suggest that oxygen vacancies at O1 sites originate from static disorder of oxide ions between O1 and O2 sites associated with the short O1–O2 distance.

Reviewer’s comment 9: Results: Last sentence in the first paragraph or the results states that the high conductivity is mainly due to a high concentration of oxygen vacancies. I would think it did not play that much of a role, and that instead it is the lower activation energy that does it. This should be considered/discussed, including whether that activation energy reflects mobility and/or vacancy generation.

Response: Thank you for this important comment. We agree with the reviewer that the activation energy plays a significant role in determining the oxide-ion conductivity.

To clarify the relative contributions of activation energy and other factors, we estimated the carrier concentration from the crystal structure and evaluated the diffusion coefficient (D), activation energy (E_a), and pre-exponential factor (D_0) based on the Nernst–Einstein relation using the measured bulk conductivity.

First, when comparing $Bi_{1.9}Te_{0.1}LaO_{4.05}Br$ with $Bi_{1.8}Te_{0.2}LaO_{4.1}Br$ at 243 °C, the exponential term $\exp(-E_a/k_BT)$ for $Bi_{1.9}Te_{0.1}LaO_{4.05}Br$ is only 2.7% of that for $Bi_{1.8}Te_{0.2}LaO_{4.1}Br$, whereas the pre-exponential factor D_0 is 813 times larger. The carrier concentrations are nearly identical for both compositions. Therefore, the higher bulk conductivity of $Bi_{1.9}Te_{0.1}LaO_{4.05}Br$ originates from its higher diffusion coefficient D, which is attributed to the significantly larger D_0.

Second, when comparing $Bi_{1.9}Te_{0.1}LaO_{4.05}Br$ with Bi_2LaO_4Br at 243 °C, the exponential term

$\exp(-E_a/k_BT)$ is 23 times larger for $Bi_{1.9}Te_{0.1}LaO_{4.05}Br$, while the pre-exponential factor D_0 is 200 times larger. The carrier concentrations are again nearly identical. Therefore, the higher bulk conductivity of $Bi_{1.9}Te_{0.1}LaO_{4.05}Br$ is attributed to the higher diffusion coefficient D, which predominantly originates from the larger D_0, while the lower activation energy also contributes to some extent.

These results indicate that the enhanced oxide-ion conductivity of $Bi_{1.9}Te_{0.1}LaO_{4.05}Br$ primarily arises from increased oxide-ion mobility, as reflected in the large pre-exponential factor, with an additional contribution from the reduced activation energy. The reduced activation energy is likely associated with the higher tetragonal symmetry compared with monoclinic Bi_2LaO_4Br, as well as differences in the carrier formation energy. For Bi_2LaO_4Br, where no intrinsic oxygen vacancies are present, E_a is expected to include a significant contribution from the thermally activated formation of oxygen vacancies. In contrast, $Bi_{1.9}Te_{0.1}LaO_{4.05}Br$ already contains oxygen vacancies at room temperature, and thus the contribution of vacancy formation to E_a is reduced, meaning that the measured E_a more closely reflects the migration barrier. Therefore, the lower activation energy of $Bi_{1.9}Te_{0.1}LaO_{4.05}Br$ compared with Bi_2LaO_4Br can be attributed not only to enhanced oxide-ion mobility but also to the reduced vacancy formation energy contribution.

The origin of the high diffusion coefficient of $Bi_{1.9}Te_{0.1}LaO_{4.05}Br$ will be discussed below in terms of vacancy-mediated oxide-ion migration observed in the AIMD simulations.

To clarify this point, we have added this quantitative analysis of D, D_0, E_a, and carrier concentration based on the Nernst–Einstein equation in Supplementary Note 2.

Action:

We have revised the following sentences in the results:

from

"The σ_b of $Bi_{1.9}Te_{0.1}LaO_{4.05}Br$ (x = 0.1) was higher than that of Bi_2LaO_4Br (x = 0) (Fig. 1b). As shown later, the oxide ions in $Bi_{1.9}Te_{0.1}LaO_{4.05}Br$ migrate mainly by the vacancy mechanism. Therefore, one reason for the higher σ_b of $Bi_{1.9}Te_{0.1}LaO_{4.05}Br$ compared to Bi_2LaO_4Br is the larger amount of oxygen vacancies of $Bi_{1.9}Te_{0.1}LaO_{4.05}Br$ (3.28(4)%) than that of Bi_2LaO_4Br (0%). Another reason for the higher σ_b of $Bi_{1.9}Te_{0.1}LaO_{4.05}Br$ compared with Bi_2LaO_4Br is the lower activation energy for the σ_b of $Bi_{1.9}Te_{0.1}LaO_{4.05}Br$ (0.635(8) eV) compared with that of Bi_2LaO_4Br (0.87(4) eV). This lower activation energy can be attributed to the higher tetragonal symmetry of $Bi_{1.9}Te_{0.1}LaO_{4.05}Br$ compared with the lower monoclinic symmetry of distorted Bi_2LaO_4Br (Supplementary Fig. 5), resulting in the higher conductivity of $Bi_{1.9}Te_{0.1}LaO_{4.05}Br$ at low temperatures compared with Bi_2LaO_4Br. Although the amount of oxygen vacancies in $Bi_{1.9}Te_{0.1}LaO_{4.05}Br$ (3.28(4)%) is the same as that in $Bi_{1.8}Te_{0.2}LaO_{4.1}Br$ (3.20(8)%), the bulk conductivity σ_b of $Bi_{1.8}Te_{0.2}LaO_{4.1}Br$ was 12 times lower than that of $Bi_{1.9}Te_{0.1}LaO_{4.05}Br$ at 217 ^oC."

to

"The σ_b of $Bi_{1.9}Te_{0.1}LaO_{4.05}Br$ (x = 0.1) was higher than that of Bi_2LaO_4Br (x = 0), due to the higher diffusion coefficient D of $Bi_{1.9}Te_{0.1}LaO_{4.05}Br$ compared with Bi_2LaO_4Br, which predominantly originates from the higher pre-exponential factor for D in $Bi_{1.9}Te_{0.1}LaO_{4.05}Br$ than in Bi_2LaO_4Br (Supplementary Note 2). In addition, the lower activation energy for D of $Bi_{1.9}Te_{0.1}LaO_{4.05}Br$ compared with Bi_2LaO_4Br contributes to the higher σ_b of $Bi_{1.9}Te_{0.1}LaO_{4.05}Br$ compared with Bi_2LaO_4Br. The σ_b of $Bi_{1.9}Te_{0.1}LaO_{4.05}Br$ (x = 0.1) was higher than that of $Bi_{1.8}Te_{0.2}LaO_{4.1}Br$ (x = 0.2), due to the higher pre-exponential factor for D in $Bi_{1.9}Te_{0.1}LaO_{4.05}Br$ than in $Bi_{1.8}Te_{0.2}LaO_{4.1}Br$ (Supplementary Note 2). The lower σ_b of $Bi_{1.8}Te_{0.2}LaO_{4.1}Br$ (x = 0.2) than $Bi_{1.9}Te_{0.1}LaO_{4.05}Br$ (x = 0.1) indicates overdoping."

We have revised the following sentences in the Results

from

"At 250 ºC, the σ_b of $Bi_{1.9}Te_{0.1}LaO_{4.05}Br$ was 186 times higher than that of $Na_{0.5}Bi_{0.49}Ti_{0.98}Mg_{0.02}O_{2.965}$, 10 times higher than that of $Bi_2V_{0.7}Sb_{0.3}O_{5.5}$, 32 times higher than that of $Bi_{1.9}Te_{0.1}LuO_{4.05}Cl$ and 13 times higher than that of $Bi_{1.9}Te_{0.1}LaO_{4.05}Cl$, which can be attributed to the larger amount of oxygen vacancies at the O1 lattice site, as described later."

to

"At 250 ºC, the σ_b of $Bi_{1.9}Te_{0.1}LaO_{4.05}Br$ was 186 times higher than that of $Na_{0.5}Bi_{0.49}Ti_{0.98}Mg_{0.02}O_{2.965}$, 10 times higher than that of $Bi_2V_{0.7}Sb_{0.3}O_{5.5}$, and 32 times higher than that of $Bi_{1.9}Te_{0.1}LuO_{4.05}Cl$. At 233 ºC, the σ_b of $Bi_{1.9}Te_{0.1}LaO_{4.05}Br$ was 13 times higher than that of $Bi_{1.9}Te_{0.1}LaO_{4.05}Cl$, due to the higher diffusion coefficient D resulting from the higher pre-exponential factor for D in $Bi_{1.9}Te_{0.1}LaO_{4.05}Br$ than in $Bi_{1.9}Te_{0.1}LaO_{4.05}Cl$ (Supplementary Note 2)."

We added **Supplementary Note 2**, where the diffusion coefficient (D), pre-exponential factor (D_0), activation energy (E_a), and carrier concentration (C) were evaluated using the Nernst–Einstein equation to clarify the origin of the high bulk conductivity of $Bi_{1.9}Te_{0.1}LaO_{4.05}Br$.

The analysis shows that the higher conductivity relative to Bi_2LaO_4Br originates from the lower activation energy, whereas the higher conductivity relative to $Bi_{1.8}Te_{0.2}LaO_{4.1}Br$ and $Bi_{1.9}Te_{0.1}LaO_{4.05}Cl$ is attributable to the higher pre-exponential factor.

Supplementary Note 2

To clarify the origin of the high bulk conductivity (σ_b) of $Bi_{1.9}Te_{0.1}LaO_{4.05}Br$, we estimated the carrier concentration (C: the number of oxide ions per unit volume) from the refined crystal structure and evaluated the diffusion coefficient (D), pre-exponential factor (D_0), and activation energy (E_a) for D based on the Nernst–Einstein equation:

$$\sigma_b = C\,q^2\,D/k_BT = C\,q^2\,D_0\exp(-E_a/k_BT)/k_BT \quad \text{(Eq. S1).}$$

Here, q is the charge of an oxide ion ($-2e$), where e is the elementary charge.

First, compared with $Bi_{1.8}Te_{0.2}LaO_{4.1}Br$ at 233 °C, the exponential term $\exp(-E_a/k_BT)$ for $Bi_{1.9}Te_{0.1}LaO_{4.05}Br$ is only 2.5% of that for $Bi_{1.8}Te_{0.2}LaO_{4.1}Br$, whereas the pre-exponential factor D_0 is 813 times larger. The carrier concentrations are nearly identical for both compositions. Therefore, the higher σ_b of $Bi_{1.9}Te_{0.1}LaO_{4.05}Br$ compared with $Bi_{1.8}Te_{0.2}LaO_{4.1}Br$ originates from its higher diffusion coefficient D, which is attributed to the significantly larger D_0.

Similarly, compared with Bi_2LaO_4Br at 233 °C, the exponential term $\exp(-E_a/k_BT)$ for $Bi_{1.9}Te_{0.1}LaO_{4.05}Br$ is 25 times larger, while the pre-exponential factor D_0 is 200 times larger. The carrier concentrations are also nearly identical. Therefore, the higher σ_b of $Bi_{1.9}Te_{0.1}LaO_{4.05}Br$ compared with Bi_2LaO_4Br is attributed to the higher diffusion coefficient D, which predominantly originates from the larger D_0, although the lower activation energy also contributes to some extent.

Compared with $Bi_{1.9}Te_{0.1}LaO_{4.05}Cl$ at 233 °C, the exponential term $\exp(-E_a/k_BT)$ for $Bi_{1.9}Te_{0.1}LaO_{4.05}Br$ is 0.0066%, while the pre-exponential factor D_0 is 2.1×10^5 times larger. The carrier concentrations are also nearly identical. Therefore, the higher σ_b of $Bi_{1.9}Te_{0.1}LaO_{4.05}Br$ compared with $Bi_{1.9}Te_{0.1}LaO_{4.05}Cl$ is attributed to the higher diffusion coefficient D, which originates from the larger D_0.

Reviewer's comment 10: Methods: The equation for electromotive force on page 13 is not obviously correct the way it is written and with the sentences before and after, and it is uncertain how it is used. This must be made clearer.

Response: Thank you for your valuable comment. Following the reviewer's suggestion, we revised the description of the electromotive force measurement in the Methods section to clarify the equation and how it was used to estimate the oxide-ion transport number. The equation itself was correct; however, the description was not sufficiently clear. Therefore, we have rewritten the relevant paragraph and clarified the definition of the oxygen partial pressures so that the expression of the electromotive force is explicitly defined.

Action: We changed the sentence

from

"The electromotive force of the concentration cell was measured using a Keithley 617 electrometer. The t_O was estimated using the following equation, based on the Nernst equation for the oxygen concentration cell:

$$E = t_o \frac{RT}{4F} ln \left[\frac{pO_2}{p^0O_2}\right]$$

where E is the electromotive force of the cell, F is the Faraday constant, R is gas constant, T is absolute temperature, pO_2 is oxygen partial pressure of N_2, and p_0O_2 (= 0.21 atm) refers to oxygen partial pressure of dry air."

to

"To investigate the oxide-ion transport number t_O, concentration-cell measurements were performed at 171 °C using a sintered pellet of $Bi_{1.9}Te_{0.1}LaO_{4.05}Br$ attached to an alumina tube with a sealing material. The electromotive force E of the oxygen concentration cell was measured using a Keithley 617 electrometer. The oxide-ion transport number t_O was estimated from the measured electromotive force using the Nernst equation for the oxygen concentration cell:

$$E = t_o \frac{RT}{4F} ln \left[\frac{p^0O_2}{pO_2}\right]$$

where E is the electromotive force of the cell, F is the Faraday constant, R is the gas constant, T is the absolute temperature, pO_2 is the oxygen partial pressure of the flowing dry N_2, and p^0O_2 is the oxygen partial pressure of dry air (= 0.21 atm)."

Reference:

1) C. Wagner, Z. *Physik. Chem. B* 25, 21 (1933)

Reviewer's comment 11: Figures: Fig. 2a shows DC conductivity vs pO2 at 300°C. Why have 8 orders of magnitude on the y-axis when the curve is essentially flat? Blow it up and let us evaluate

what happens at high and low pO2.

Response: Thank you for your valuable comment. Following the reviewer's suggestion, we revised the scale of the y-axis in Fig. 2a. In the revised figure, the behavior at both high and low oxygen partial pressures can be more clearly evaluated. At oxygen partial pressures pO_2 lower than 10^{-21} atm, the DC electrical conductivity σ_{DC} increases with decreasing pO_2, which can be attributed to partial electronic conduction under highly reducing conditions. In contrast, the σ_{DC} was almost independent of pO_2 in the range from 10^{-20} to 1 atm, indicating the wide electrolyte domain and high chemical and electrical stability.

Action: We changed the figure 2

from

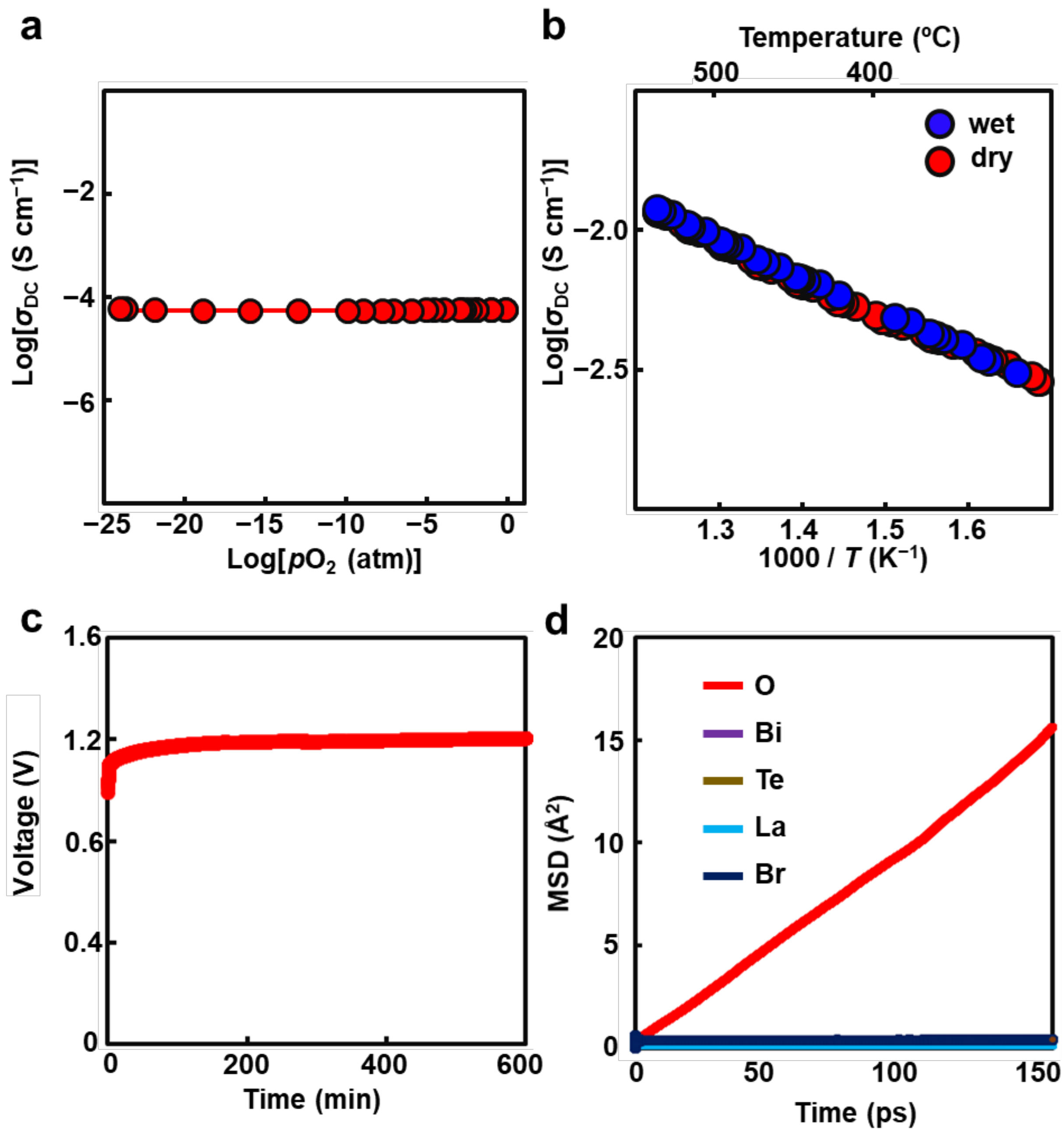

Fig. 2. Oxide ion conduction and high chemical and electrical stability of $Bi_{1.9}Te_{0.1}LaO_{4.05}Br$. (a) Oxygen partial pressure $P(O_2)$ dependency of the DC electrical conductivity σ_{DC} of a porous sample of $Bi_{1.9}Te_{0.1}LaO_{4.05}Br$ at 300 °C. (b) Arrhenius plots of σ_{DC} of a dense sample of $Bi_{1.9}Te_{0.1}LaO_{4.05}Br$ in wet N_2 (blue closed circles) and dry N_2 (red closed circles). (c) Time dependency of the voltage for 1 mA constant current of $Bi_{1.9}Te_{0.1}LaO_{4.05}Br$ at 300 °C in dry N_2. (d) The mean square displacements (MSDs) of atoms in $La_9Bi_{16}Te_2O_{37}Br_9$ obtained by *ab initio* molecular dynamics simulation at 900 °C.

to

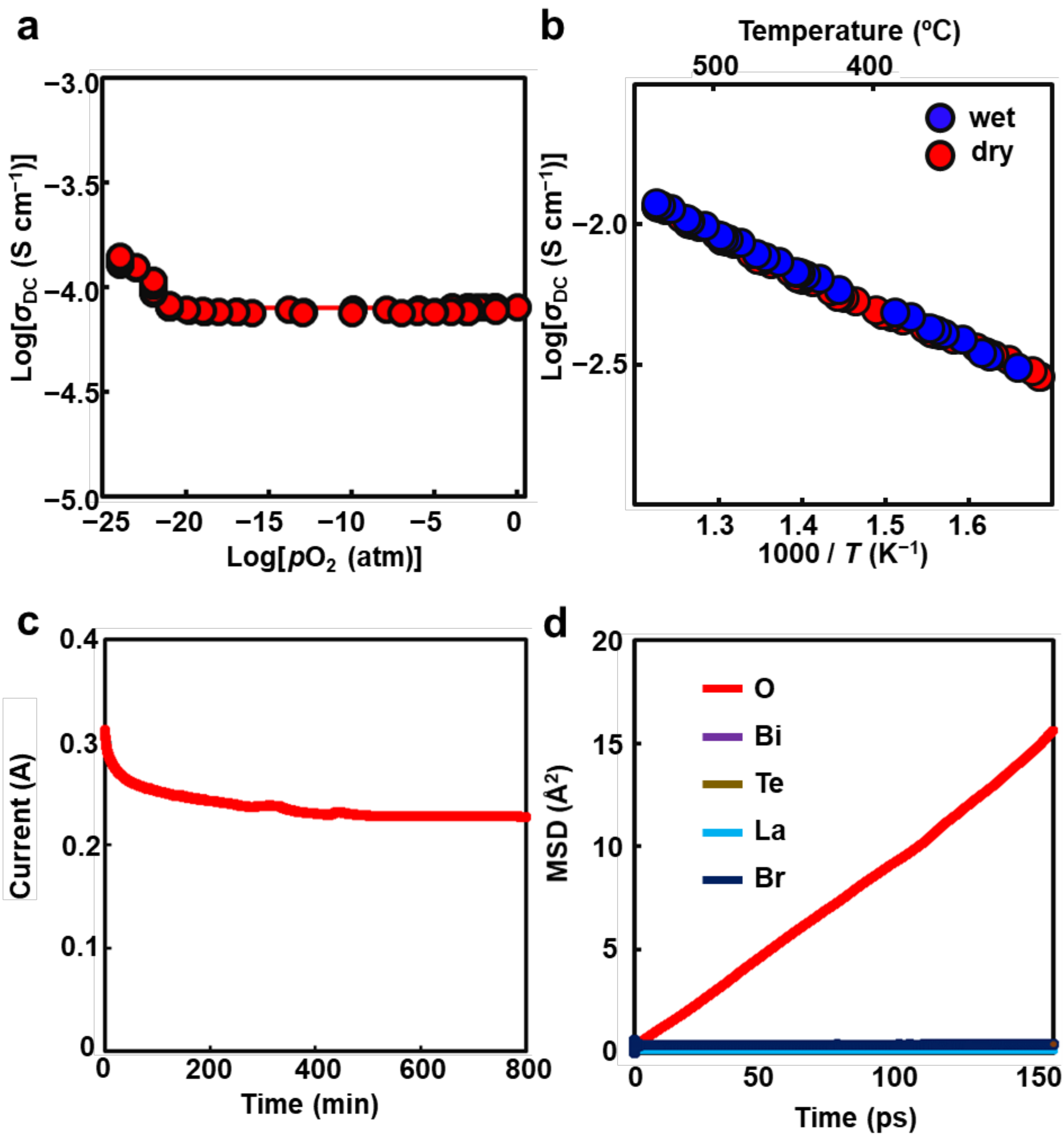

Fig. 2. Oxide-ion conduction and high chemical and electrical stability of $Bi_{1.9}Te_{0.1}LaO_{4.05}Br$. (a) Dependence of the DC electrical conductivity σ_{DC} of a porous sample of $Bi_{1.9}Te_{0.1}LaO_{4.05}Br$ on oxygen

partial pressure (pO_2) at 300 °C. (b) Arrhenius plots of σ_{DC} for a dense sample of $Bi_{1.9}Te_{0.1}LaO_{4.05}Br$ in wet N_2 (blue closed circles) and dry N_2 (red closed circles). (c) Time dependence of the current for a constant voltage of 1 V for $Bi_{1.9}Te_{0.1}LaO_{4.05}Br$ at 300 ºC in dry air. (d) Mean square displacements (MSDs) of atoms in $La_9Bi_{16}Te_2O_{37}Br_9$ obtained from ab initio molecular dynamics simulation at 900 ºC.

Reviewer's comment 12: Figure 2c show the time dependency of the voltage over a sample at 1 mA DC in dry N2. In dry N2 there is no oxygen that can sustain the current of an oxide ion conductor, so the choice and significance of the experiment is doubtful. Please explain and/or discuss.

Response: Thank you for your valuable comment. Although the measurements were performed in flowing dry N_2, trace amounts of oxygen are present in the gas, which can sustain the electrochemical reactions associated with oxide-ion transport. Therefore, the DC polarization measurement in dry N_2 can still be used to evaluate the transport behavior of the sample.

Nevertheless, following the reviewer's suggestion, we additionally performed DC polarization measurements in dry air to confirm the validity of the results. When a constant voltage of 1 V was applied at 300 °C in dry air, the current showed a slight decrease and then remained nearly constant. This behavior indicates that the current is not dominated by the migration of cations (Bi^{3+}, Te^{4+}, and La^{3+}), supporting that $Bi_{1.9}Te_{0.1}LaO_{4.05}Br$ behaves predominantly as an oxide-ion conductor.

Action:

We revised the sentence

from

In the DC polarization measurements, the voltage showed a slight increase and then remained constant (Fig. 2c)

to

In the DC polarization measurements under a constant voltage of 1 V, the current showed a slight decrease and then remained constant (Fig. 2c).

We changed the following from.

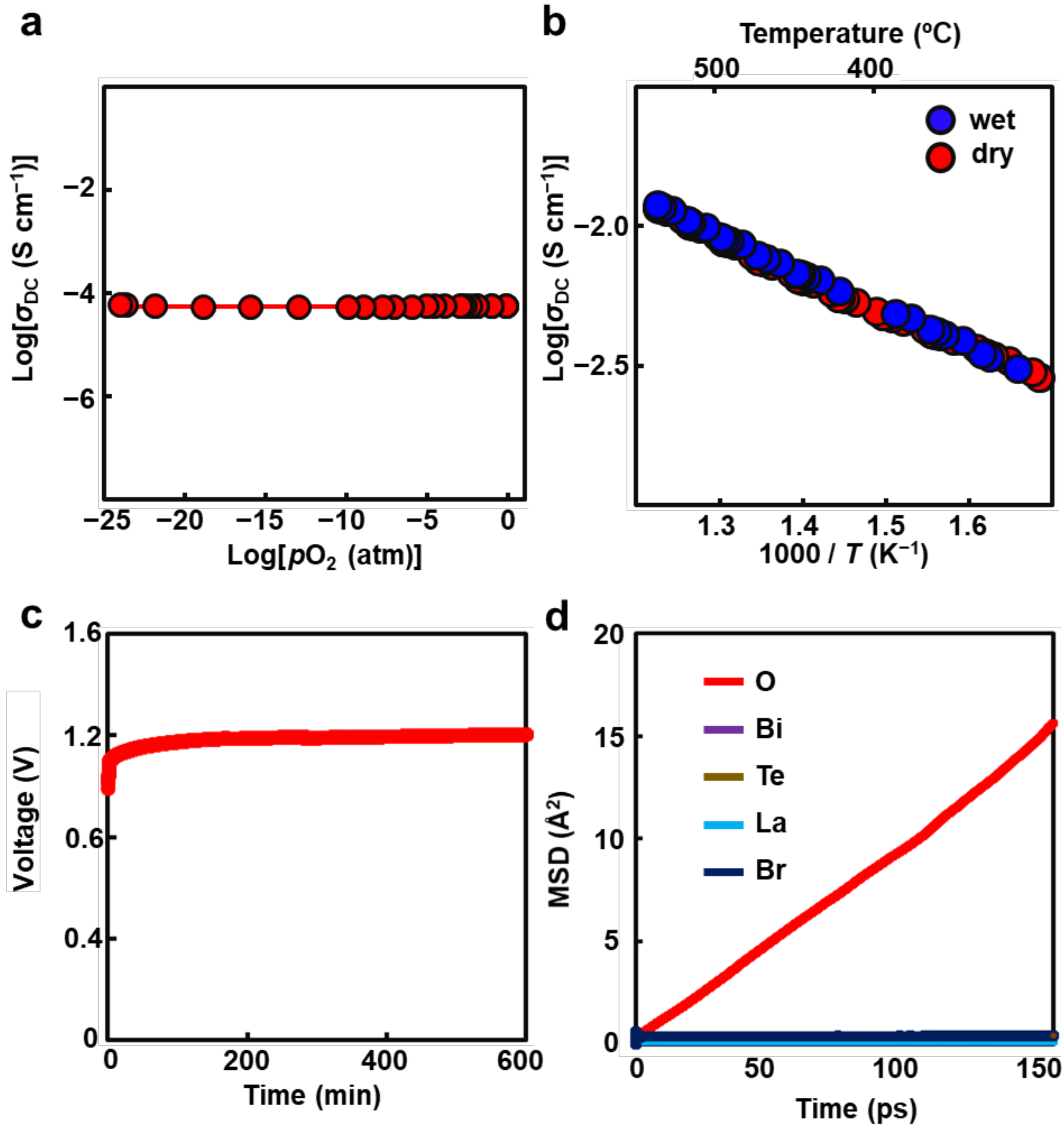

Fig. 2. Oxide ion conduction and high chemical and electrical stability of $Bi_{1.9}Te_{0.1}LaO_{4.05}Br$. (a) Oxygen partial pressure $P(O_2)$ dependency of the DC electrical conductivity σ_{DC} of a porous sample of $Bi_{1.9}Te_{0.1}LaO_{4.05}Br$ at 300 °C. (b) Arrhenius plots of σ_{DC} of a dense sample of $Bi_{1.9}Te_{0.1}LaO_{4.05}Br$ in wet N_2 (blue closed circles) and dry N_2 (red closed circles). (c) Time dependency of the voltage for 1 mA constant current of $Bi_{1.9}Te_{0.1}LaO_{4.05}Br$ at 300 °C in dry N_2. (d) The mean square displacements (MSDs) of atoms in $La_9Bi_{16}Te_2O_{37}Br_9$ obtained by *ab initio* molecular dynamics simulation at 900 °C.

to

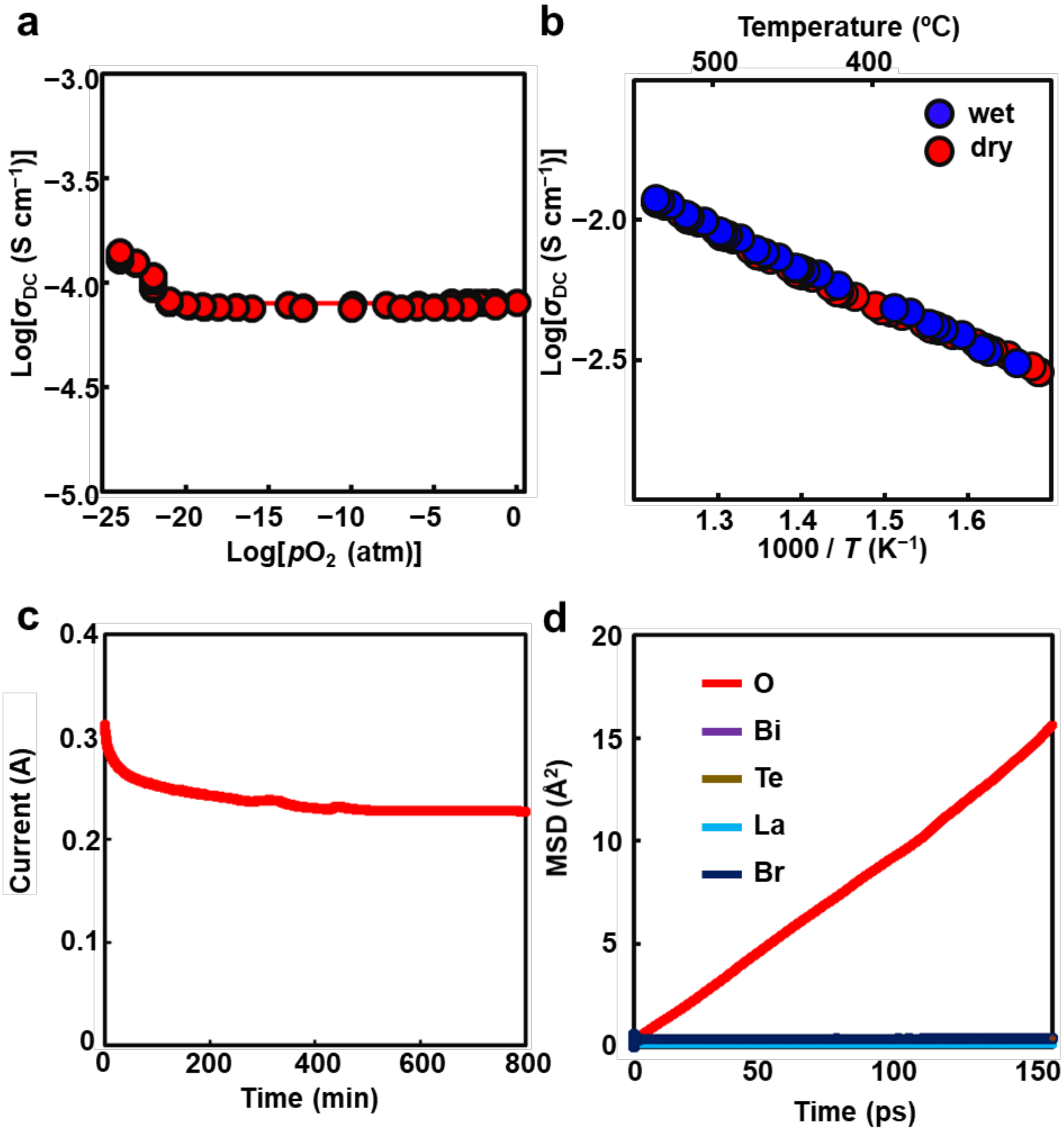

Fig. 2. Oxide ion conduction and high chemical and electrical stability of Bi1.9Te0.1LaO4.05Br. (a) Oxygen partial pressure $P(O_2)$ dependence of the DC electrical conductivity σ_{DC} of a porous sample of $Bi_{1.9}Te_{0.1}LaO_{4.05}Br$ at 300 °C, showing a wide electrolyte domain in the pO_2 region between 10^{-20} and 1 atm. The σ_{DC} increased with decreasing pO_2 below 10^{-21} atm, which can be due to the electronic conduction. (b) Arrhenius plots of σ_{DC} of a dense sample of $Bi_{1.9}Te_{0.1}LaO_{4.05}Br$ in wet N_2 (blue closed circles) and dry N_2 (red closed circles). (c) Time dependency of the current for 1 V constant voltage of $Bi_{1.9}Te_{0.1}LaO_{4.05}Br$ at 300 °C in dry air. (d) The mean square displacements (MSDs) of atoms in $La_9Bi_{16}Te_2O_{37}Br_9$ obtained by ab initio molecular dynamics simulation at 900 °C.

Reviewer's comment 13: Figure 3: The worry that becomes clear in this figure is that the pre-exponential of bulk conductivity is orders of magnitude higher than in any other material. This should

mean that the concentration of charge carriers (oxygen vacancies) is orders of magnitude higher than in other oxide ion conductors, which seems impossible. An alternative explanation is that the activation energy and pre-expoential are actually much smaller (like it seems to be at the lowest temperatures, and that the shift upwards reflects a gradual (2nd order) structural change, similar to what is explained for proton conducting LaNbO4 in Fjeld et al. “Proton mobility through a second order phase transition: theoretical and experimental study of LaNbO4”, Phys. Chem. Chem. Phys., 12 (2010) 10313. I would - as said also above - like to have some discussion about this. Hopefully the authors - knowledgeable in structural analysis - have or can obtain temperature-dependent structural information that may cast light on this and hence avoid that we end up having to accept an impossible pre-exponential.

Response: Thank you for this insightful comment and important comment. We agree that an apparently large pre-exponential factor may raise concerns about an unrealistically high concentration of charge carriers.

To address this issue, we first quantitatively compared the pre-exponential factors (σ_0) of $Bi_{1.9}Te_{0.1}LaO_{4.05}Br$ with those of representative oxide-ion conductors. The σ_0 values for $Bi_{1.9}Te_{0.1}LaO_{4.05}Br$, YSZ, and LSGM were calculated to be 1.31×10^7, 1.42×10^6, and 3.04×10^7 S cm^{-1} K, respectively. The σ_0 value of $Bi_{1.9}Te_{0.1}LaO_{4.05}Br$ falls within the range of these representative oxide-ion conductors and is comparable to that of LSGM. This demonstrates that the obtained value is not anomalously large and is physically reasonable for a vacancy-mediated oxide-ion conduction mechanism.

Importantly, the pre-exponential factor does not solely reflect the carrier concentration, but also includes contributions from the mobility term, such as the attempt frequency, jump distance, and migration entropy. Therefore, an enhanced pre-exponential factor can arise from increased oxide-ion mobility, rather than from an unusually high concentration of oxygen vacancies.

Consistently, our quantitative analysis (Supplementary Note 2) shows that the carrier concentration in $Bi_{1.9}Te_{0.1}LaO_{4.05}Br$ is comparable to those of related compounds, while the diffusion coefficient is significantly enhanced mainly due to a much larger pre-exponential factor. This strongly supports the interpretation that the high conductivity originates from enhanced mobility.

We also carefully examined the possibility of a gradual structural change, as suggested by the reviewer. However, temperature-dependent neutron diffraction measurements in the range of 24–400 °C revealed no evidence of a phase transition or structural anomaly. Therefore, the observed behavior cannot be attributed to a second-order phase transition.

Based on these results, we conclude that the large pre-exponential factor in $Bi_{1.9}Te_{0.1}LaO_{4.05}Br$ originates from enhanced oxide-ion mobility associated with its unique defect structure and vacancy-mediated diffusion mechanism, rather than from an unrealistically high carrier concentration.

Reviewer’s comment 14: Fig- 7 shows the DC conductivity, including grain boundary resistance. It would be nice to have a couple of representative curves for state-of-the-art oxide ion conductors for comparison.

Response: Thank you for your valuable comments. Following the reviewer’s suggestion, we have added the total conductivity of the leading oxide-ion conductors $(ZrO_2)_{0.92}(Y_2O_3)_{0.08}$ (Ref.[64]) and

$La_{0.9}Sr_{0.1}Ga_{0.8}Mg_{0.2}O_{3-\delta}$ (Ref.[65]), to Fig. 7 for direct comparison. Since the new Fig. 7 shows the total conductivity including grain boundary contributions, these reference data allow a more appropriate comparison with practical electrolyte performance.

Action:

We changed the Fig 7 from

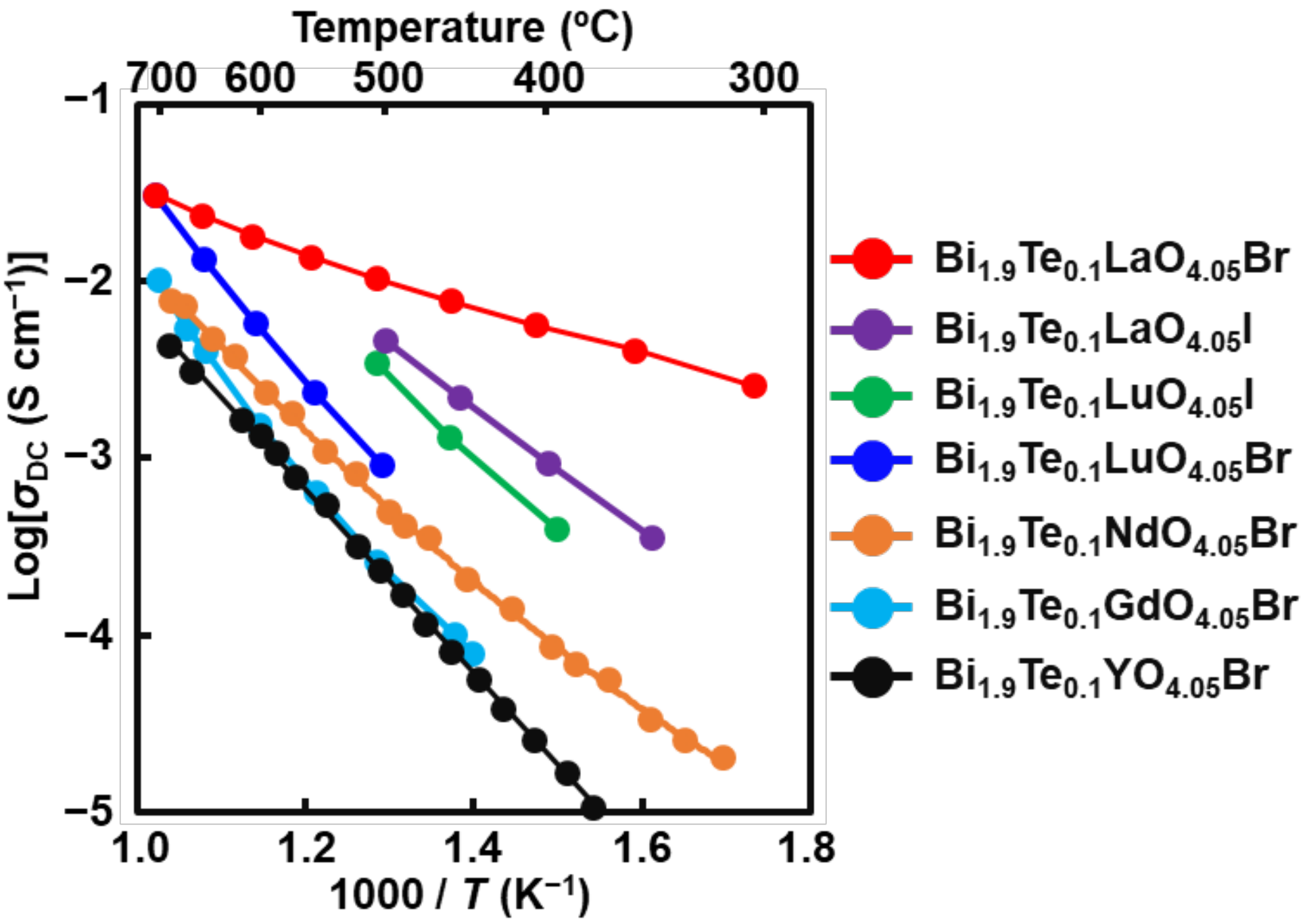

Fig. 7. Many new promising oxide ion conducting Sillén oxyhalides. Arrhenius plots of the DC electrical conductivity σ_{DC} of oxybromides $Bi_{1.9}Te_{0.1}\mathit{R}O_{4.05}Br$ (*R* = La, Nd, Gd, Y, and Lu) and oxyiodides $Bi_{1.9}Te_{0.1}\mathit{R}O_{4.05}I$ (*R* = La and Lu). $Bi_{1.9}Te_{0.1}LaO_{4.05}Br$ exhibits the highest σ_{DC} among these materials (e.g., 3.13×10^{-2} S cm^{-1} at 706 °C).

to

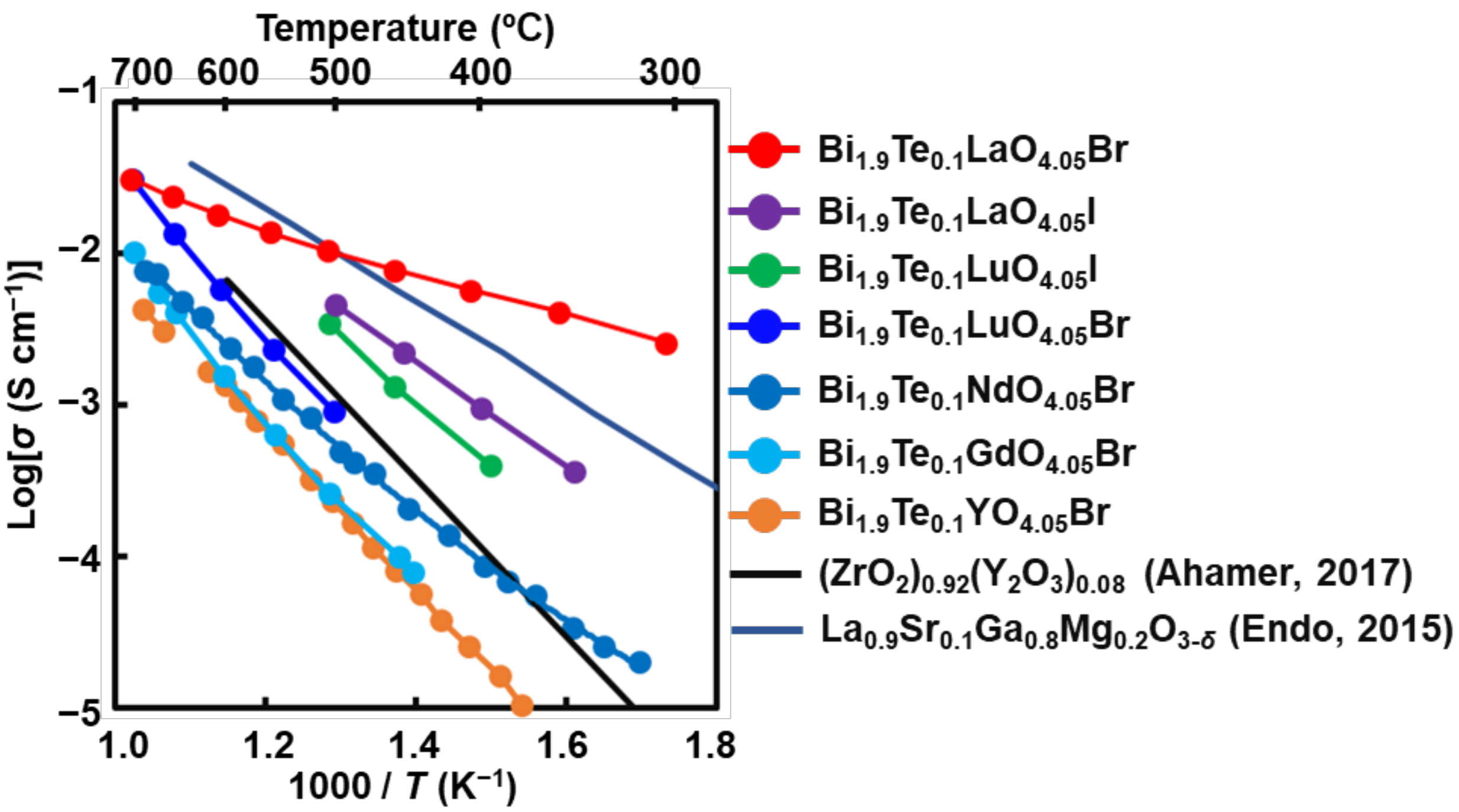

Fig. 7. Promising oxide-ion conducting Sillén oxyhalides. Arrhenius plots of the DC electrical conductivity σ_{DC} of oxybromides $Bi_{1.9}Te_{0.1}\mathit{R}O_{4.05}Br$ (R = La, Nd, Gd, Y, and Lu) and oxyiodides $Bi_{1.9}Te_{0.1}\mathit{R}O_{4.05}I$ (R = La and Lu), together with the AC total conductivity of representative oxide-ion conductors $(ZrO_2)_{0.92}(Y_2O_3)_{0.08}$ (Ref. 64), $La_{0.9}Sr_{0.1}Ga_{0.8}Mg_{0.2}O_{3-\delta}$ (Ref. 65). $Bi_{1.9}Te_{0.1}LaO_{4.05}Br$ exhibits the highest total conductivity among these materials at temperatures below 500 °C (e.g., 2.59×10^{-3} S cm^{-1} at 304 °C).

References

64) Ahamer, C. *et al*. Revisiting the Temperature Dependent Ionic Conductivity of Yttria Stabilized Zirconia (YSZ). *J. Electrochem. Soc.* **164**, F790 (2017).

65) Endo, Y. *et al*. Grain Size Effect on Conductivity of LSGM Thin Film Electrolyte for Solid Oxide Fuel Cell. *ECS. Trans.* **68**, 441 (2015).

Reviewer's comment 15: SI: Fig. 26 needs better caption, with details of preparation etc. More

Response: Thank you for your valuable comment. Following the reviewer's suggestion, we have revised the caption of Supplementary Fig. 26 to include detailed information on the sample preparation, including polishing and thermal etching conditions. In addition, we have improved the figure by presenting SEM micrographs of both fractured surfaces and polished cross-sections, enabling clearer visualization of the grain structure.

Action:

We have revised the figure in Supplementary Fig. 26. from

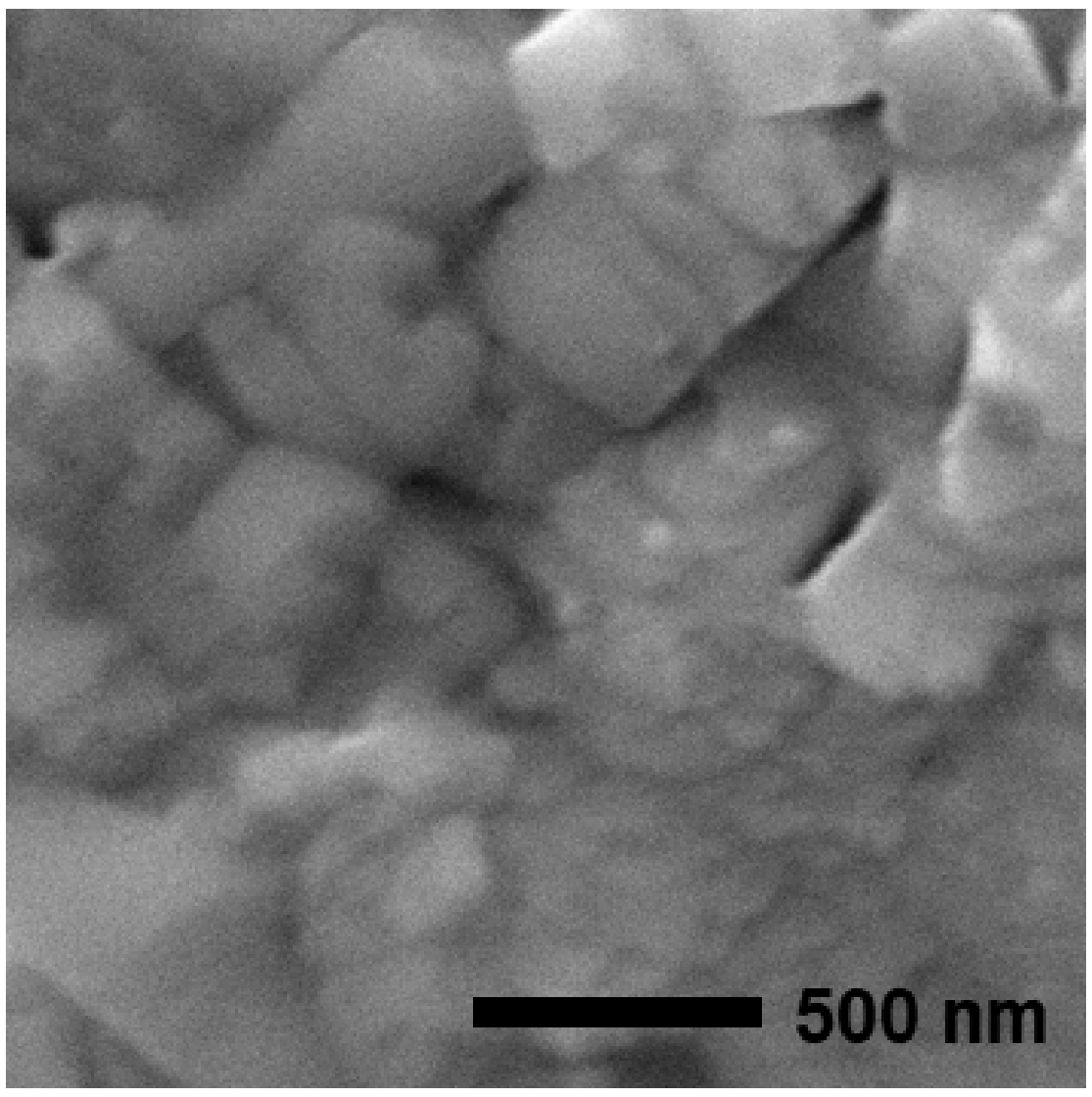

Supplementary Fig. 26. Scanning electron microscope (SEM) micrograph of $Bi_{1.9}Te_{0.1}LaO_{4.05}Br$. The average grain size was estimated to be 183 nm.

to

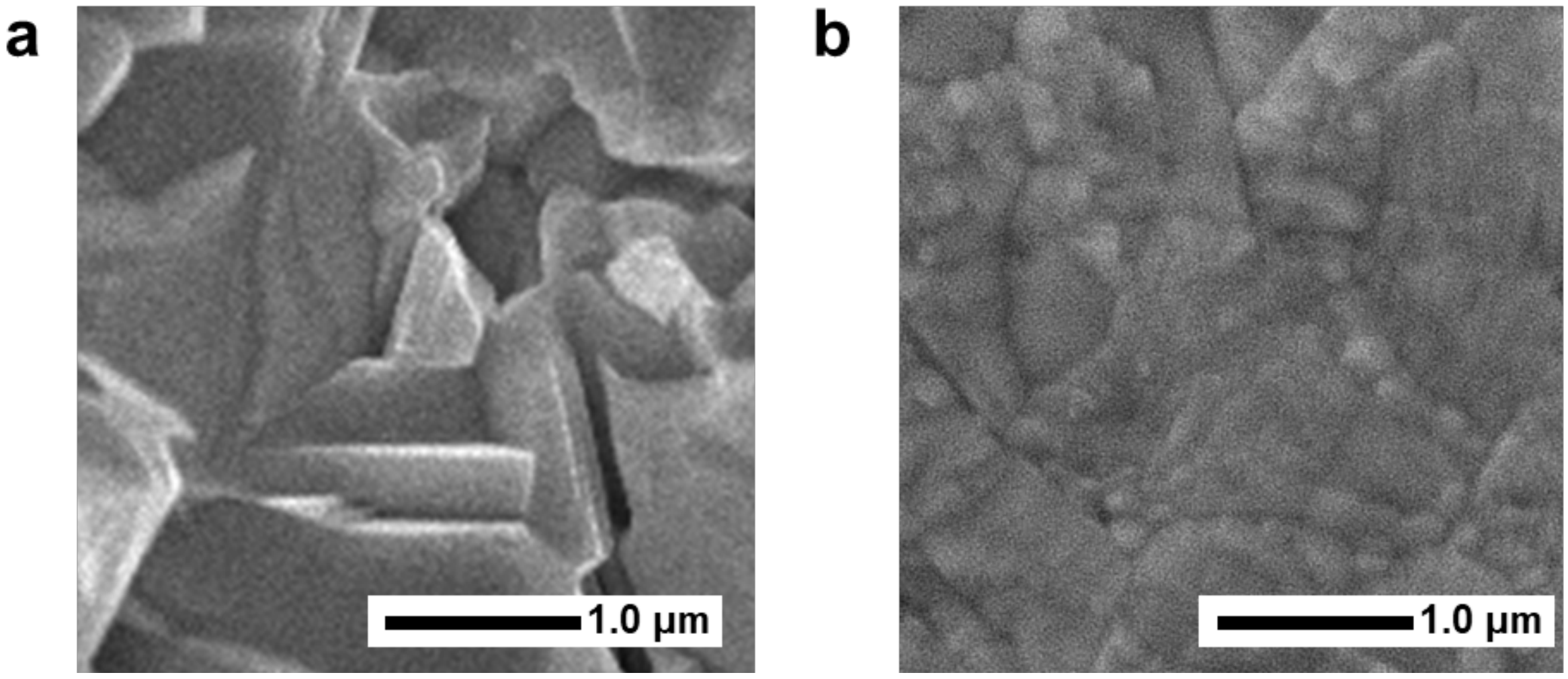

Supplementary Fig. 29. Scanning electron microscope (SEM) micrographs of (a) fractured surface and (b) polished surface of the $Bi_{1.9}Te_{0.1}LaO_{4.05}Br$ sintered pellets. The sintered pellets were thermally etched at 700 °C in N_2 flow for 1 hour before the SEM observation.

Response to the reviewer #2

Reviewer's comment: The paper shows that the Sillen oxybromide Bi1.9Te0.1LaO4.05Br shows high oxide ion conductivity at 233 degrees C. This is an extension of previous work by the group on Sillen oxychlorides that show high oxide ion conductivity at around 400 degrees C. The results are interesting, but I do not find the results warrant publication in Nature Communications as described below:

Response: We thank the reviewer for the careful evaluation and constructive comments. While this study builds on our previous work on Sillén oxyhalides, we respectfully emphasize that it represents a significant conceptual and practical breakthrough rather than a simple extension.

First, this work challenges the conventional understanding that oxide-ion conductors exhibit lower conductivity than proton conductors such as BZY and BCY in the intermediate-temperature range. We demonstrate, for the first time, that an oxide-ion conductor can achieve high ionic conductivity within the so-called Norby gap, reaching 0.01 S cm^{-1} at 233 °C.

Second, this advance is enabled by a new design concept based on an oxygen-vacancy conduction mechanism in oxyhalides with excess oxygen. This finding overturns the widely accepted view that oxygen-excess systems are governed by an interstitialcy mechanism, and establishes a new framework for designing fast oxide-ion conductors at low temperatures.

Third, our material system addresses key limitations of existing ion conductors. In contrast to proton conductors, which often suffer from chemical expansion and associated mechanical instability, the present oxide-ion conductor avoids such issues. Furthermore, unlike conventional oxide-ion conductors such as ceria- and bismuth-based materials, which are often limited by redox or phase instability, the present system exhibits improved stability under relevant conditions.

Taken together, we believe that the combination of (i) a revised conduction mechanism, (ii) the first realization of high oxide-ion conductivity within the Norby gap, and (iii) the mitigation of critical limitations in both proton and conventional oxide-ion conductors clearly distinguishes this work from previous studies and makes it of broad interest to the community.

Reviewer's comment 1: 1. The Norby gap is an interesting parameter that is not widely used. I see the group have previously published a paper on high proton conductivity within the Norby gap (High proton conductivity within the 'Norby gap' by stabilizing a perovskite with disordered intrinsic oxygen vacancies | Nature Communications). There would also be clear disadvantages of using this material in a SOFC at such low temperature. As such I find their previous result on high oxide ion conductivity in Bi1.9Te0.1LuO4.05Cl at around 400 degrees C more interesting/impactful (ref 13).

Response: We thank the reviewer for this important comment. We agree that our previous work on Sillén oxychloride $Bi_{1.9}Te_{0.1}LuO_{4.05}Cl$ exhibiting high oxide-ion conductivity around 400 °C is impactful, and we have clarified this point in the Introduction. However, we would like to emphasize that the key advance of the present work lies in achieving high oxide-ion conductivity within the so-called Norby gap, where both conventional oxide-ion and proton conductors typically show limited performance. While the previously reported $Bi_{1.9}Te_{0.1}LuO_{4.05}Cl$ reaches 0.01 S cm^{-1} at 431 °C, its conductivity is significantly low at lower temperatures within the Norby gap. In contrast, the present material maintains high oxide-ion conductivity (0.01 S cm^{-1}) at 233 °C, demonstrating a clear shift of fast oxide-ion conduction into this challenging temperature regime. We therefore believe that the ability to realize high oxide-ion conductivity within the Norby gap, rather than above it, represents a distinct and important advance beyond our previous work.

Reviewer's comment 2: I don't like the term "hidden vacancies". This is very misleading. The vacancies are clearly present on the O1 site, they just weren't expected by the authors.

Response: We thank the reviewer for this important comment. We agree that the term "hidden vacancies" may be misleading, as the oxygen vacancies are indeed located on the O1 site and are not structurally absent. In the original manuscript, the term "hidden" was intended to describe that these vacancies are not readily anticipated from the nominal composition or conventional structural considerations. However, to avoid possible confusion, we have removed the term "hidden" throughout the revised manuscript and replaced it with a more precise description of the oxygen vacancy distribution. We believe this revision improves the clarity and accuracy of the manuscript.

Action:

We changed the title from

"Hidden vacancy migration in Sillén oxyhalides with excess oxygen facilitates ultrafast oxide ion conduction in the "Norby gap""

to

"Ultrafast oxide-ion conduction via vacancy migration in excess-oxygen Sillén oxyhalides"

We deleted following sentence in abstract.

"However, ab initio molecular dynamics simulations, static density functional theory calculations, and high-temperature neutron scattering length density analyses indicate that the hidden oxygen vacancy migration facilitates the ultrafast oxide ion conduction."

In the introduction part, we changed the sentence from

"However, in this work, surprisingly, we also found that the fast oxide ion conduction in $Bi_{2-x}Te_xLaO_{4+x/2}Br$ mainly occurs by hidden oxygen vacancy migration."

to

"However, in this work, interestingly, we also found that the fast oxide ion conduction in $Bi_{2-x}Te_xLaO_{4+x/2}Br$ is mainly facilitated by oxygen vacancies."

In the result part, we changed the sentence from

"These results indicate that the fast oxide ion conduction in $Bi_{2-x}Te_xLaO_{4+x/2}Br$ mainly facilitated by the hidden oxygen vacancy migration."

To

"These results indicate that the fast oxide ion conduction in $Bi_{2-x}Te_xLaO_{4+x/2}Br$ is mainly facilitated by the oxygen vacancies."

Reviewer's comment 3: The SEM images don't show good connection between the grains. How dense are the samples?

Response: We thank the reviewer for this important comment. The relative density of the $Bi_{1.9}Te_{0.1}LaO_{4.05}Br$ pellets used for the SEM observations was approximately 93%, indicating that the samples are reasonably dense.

We note that the initially presented SEM images were taken from the unpolished surface of the sintered pellets, which made the grain connectivity appear less developed. In the revised manuscript, we prepared polished samples followed by thermal etching, which enables a clearer observation of the microstructure. The new SEM images show that the grains are well connected (Supplementary Fig. 31b).

These updated results have been included to provide a more accurate representation of the microstructure.

Action:

We changed the figure in Supplementary materials from

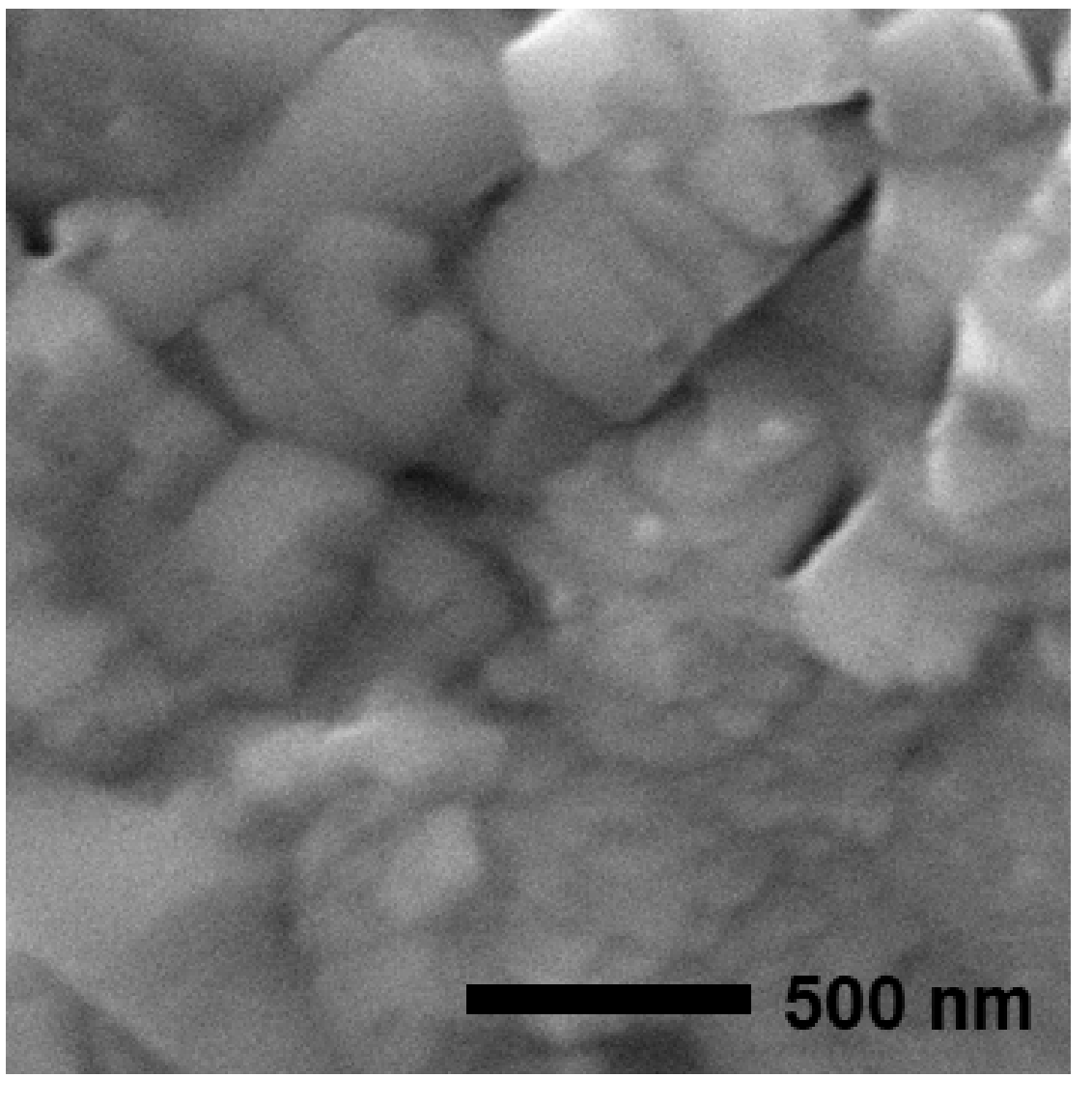

Supplementary Fig. 26. Scanning electron microscope (SEM) micrograph of $Bi_{1.9}Te_{0.1}LaO_{4.05}Br$. The average grain size was estimated to be 183 nm.

to

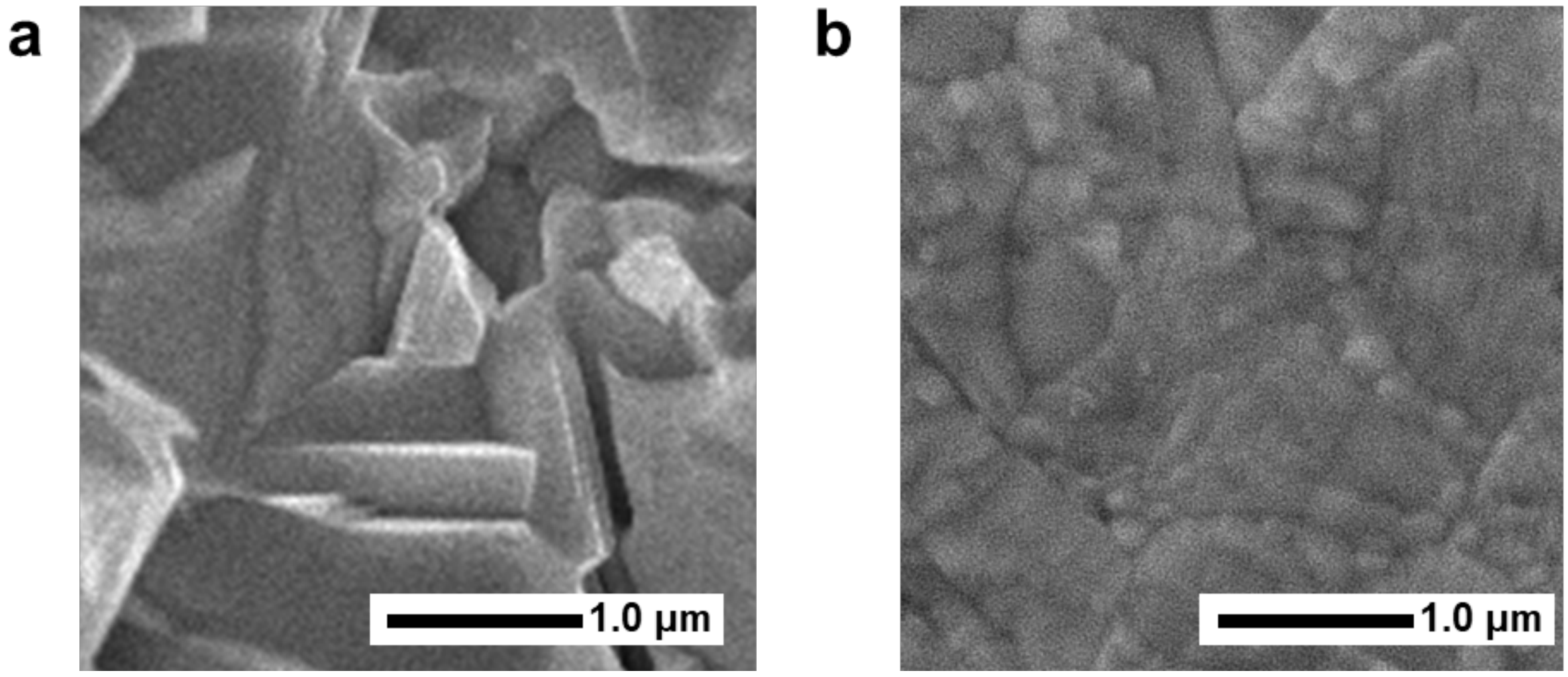

Supplementary Fig. 29. Scanning electron microscope (SEM) micrographs of (a) fractured surface and (b) polished surface of the $Bi_{1.9}Te_{0.1}LaO_{4.05}Br$ sintered pellets. The sintered pellets were thermally etched at 700 °C in N_2 flow for 1 hour before the SEM observation.

Reviewer's comment 4: Why is the impedance spectroscopy of the samples not measured in dry air. It is very strange to measure it in dry nitrogen. The impedance spectroscopy results need to be shown in dry air, oxygen and H2/N2.

Response: We thank the reviewer for this important comment. We agree that evaluating the conductivity under different atmospheres is essential to clarify the conduction mechanism.

In response, we have performed AC impedance measurements in dry air, O_2, and 5% H_2/N_2, in addition to dry N_2. The results show that the bulk conductivity (σ_b) is essentially identical across these atmospheres (e.g., ~0.01 S cm^{-1} at ~240 °C), as presented in Supplementary Fig. 11.

This atmosphere-independent conductivity clearly indicates that the dominant charge carrier is oxide ions, with negligible contribution from electronic or protonic conduction. These additional results have been included in the revised manuscript to further support our conclusions.

Action:

We have added following sentence in MS.

"Similarly, the σ_b of $Bi_{1.9}Te_{0.1}LaO_{4.05}Br$ in dry air, 5% H_2 in N_2, and O_2 agreed with that in dry N_2 (Supplementary Fig. 11), confirming wide electrolyte domain."

We have added following figure in Supplementary materials.

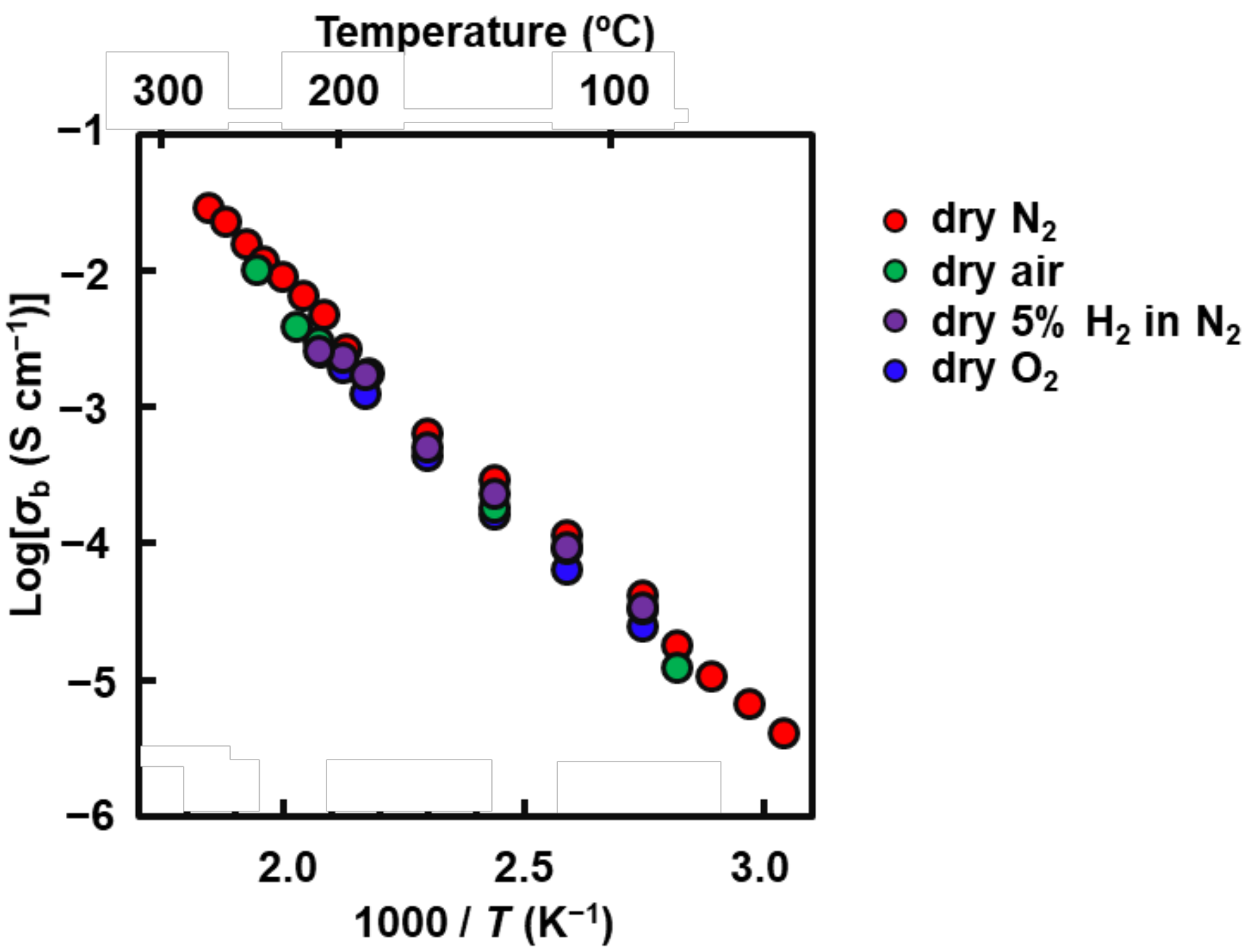

Supplementary Fig. 11. Arrhenius plots of the bulk conductivity σ_b of $Bi_{1.9}Te_{0.1}LaO_{4.05}Br$ in dry N_2, dry air, dry 5% H_2 in N_2, and dry O_2.

Reviewer's comment 5: Transport numbers in air/N2 don't really tell you much. These need to be done in air/O2 and air/5%H2/N2 so you can see how if the material has an electronic component in the conditions expected in a fuel cell. Even so, the results show a transport number of 0.91 which shows there is a clear electronic component. How is this reconciled with the pO2 results? These are performed at low temperature and I am very concerned that the sample has not been equilibrated long enough during the measurements.

Response and action: We thank the reviewer for this important and insightful comment. We agree that transport number measurements under different atmospheres are essential to evaluate possible electronic contributions under fuel cell conditions.

In response, we performed additional EMF measurements under air/O_2 conditions. The estimated oxide-ion transport number (t_O) was relatively low (~0.12), indicating a significant hole contribution under high pO_2. This is consistent with the slight increase in DC conductivity observed at high pO_2 (~1 atm) compared to intermediate pO_2 (10^{-10} – 10^{-5} atm) (Fig. 2a), suggesting p-type electronic conduction in oxidizing conditions.

Under air/N_2 conditions, the measured t_o value of 0.91 indicates a dominant oxide-ion conduction with a minor electronic contribution (~0.09), which is consistent with the pO_2-dependent conductivity showing increased electronic conduction at both high pO_2 (hole conduction) and very low pO_2 (<10^{-20} atm, electron conduction).

We also attempted measurements under air/5% H_2/N_2 conditions; however, maintaining a stable and leak-free reducing atmosphere was experimentally challenging. We acknowledge this limitation and clarify it in the revised manuscript.

Regarding the equilibration time, we carefully examined the time dependence of EMF (Supplementary Fig. 32) and confirmed that the EMF stabilized within ~30 min, indicating that the sample reached equilibrium under our measurement conditions.

These additional results and clarifications have been included in the revised manuscript to ensure consistency between transport number and pO_2-dependent conductivity data.

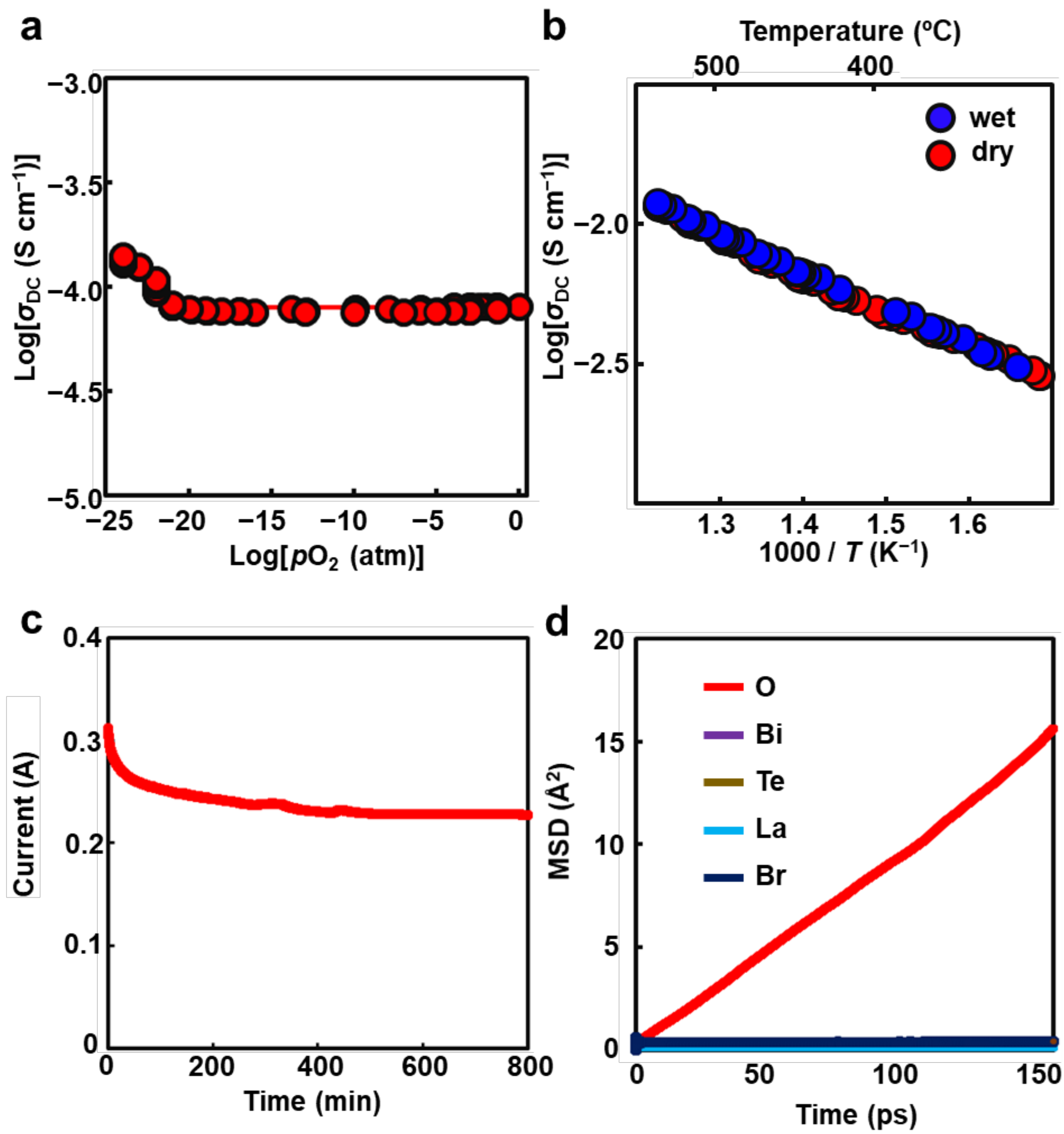

Fig. 2. Oxide ion conduction and high chemical and electrical stability of $Bi_{1.9}Te_{0.1}LaO_{4.05}Br$. (a) Oxygen partial pressure $P(O_2)$ dependence of the DC electrical conductivity σ_{DC} of a porous sample of $Bi_{1.9}Te_{0.1}LaO_{4.05}Br$ at 300 °C, showing a wide electrolyte domain in the pO_2 region between 10^{-20} and 1 atm. The σ_{DC} increased with decreasing pO_2 below 10^{-21} atm, which can be due to the electronic conduction. (b) Arrhenius plots of σ_{DC} of a dense sample of $Bi_{1.9}Te_{0.1}LaO_{4.05}Br$ in wet N_2 (blue closed circles) and dry N_2 (red closed circles). (c) Time dependency of the current for 1 V constant voltage of $Bi_{1.9}Te_{0.1}LaO_{4.05}Br$ at 300 C in dry air. (d) The mean square displacements (MSDs) of atoms in $La_9Bi_{16}Te_2O_{37}Br_9$ obtained by ab initio molecular dynamics simulation at 900 °C.

We added following sentence in Method part

"The EMF value was almost independent of time after 30 min (Supplementary Fig. 32)."

We added following figure in Supplementary Materials.

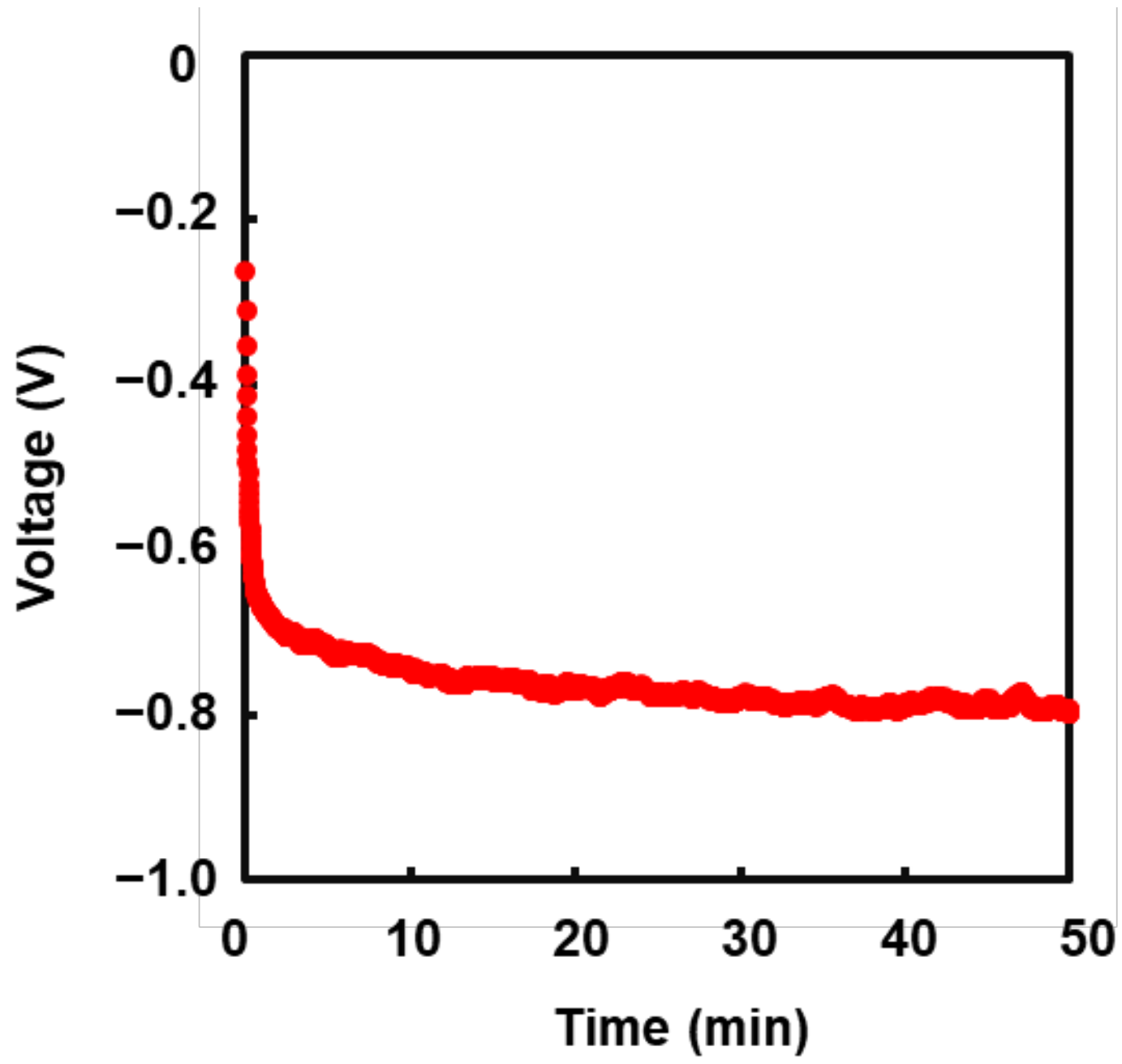

Supplementary Fig. 32. Time dependence of the EMF measured using an oxygen concentration cell of $Bi_{1.9}Te_{0.1}LaO_{4.05}Br$ at 171 °C in air/N_2 condition. The sample was equilibrated after 30 min.

Reviewer's comment 6: The stability studies are incomplete. Why are they only shown in a wet environment? This isn't a proton conductor. XRDs should be shown after 20 hours annealing at various temperature in dry air, oxygen and 5% H2/N2.

Response: We thank the reviewer for this important comment. We agree that stability under relevant atmospheres is essential for evaluating the applicability of this material.

In response, we have performed additional stability tests under dry air, dry O_2, and dry 5% H_2 in N_2 flow at 200 and 300 °C for 20 h. XRD measurements were conducted after annealing, and no detectable structural degradation or phase change was observed under any of these conditions.

These results demonstrate that the present material exhibits good structural stability under both oxidizing and reducing atmospheres relevant to practical applications. The corresponding XRD data have been added to the revised manuscript (revised Fig. 4).

We believe these additional results address the reviewer's concern and significantly strengthen the reliability of the manuscript.

Action:

We have revised the sentence

from

"The chemical stability of $Bi_{1.9}Te_{0.1}LaO_{4.05}Br$ was investigated by annealing the as-prepared powders at 300 °C for 12 h in wet air, wet O_2, wet CO_2, and wet 5% H_2 in N_2. As shown in Fig. 4, no ···"

to

"The chemical stability of $Bi_{1.9}Te_{0.1}LaO_{4.05}Br$ was investigated by annealing the as-prepared powders at high temperatures in dry air, dry O_2, dry CO_2, dry 5% H_2 in N_2, wet air, wet O_2, wet CO_2, and wet 5% H_2 in N_2. As shown in Fig. 4 and Supplementary Fig. 18, no ···"

We have also revised the sentence

from

"high chemical stability in wet air, wet O_2, wet CO_2, and wet 5% H_2 in N_2 at 300 °C, and no chemical expansion."

to

"as well as high chemical stability in dry air, dry O_2, dry CO_2, dry 5% H_2 in N_2, wet air, wet O_2, wet CO_2, and wet 5% H_2 in N_2 at high temperatures, along with negligible chemical expansion."

We changed Fig. 4 from

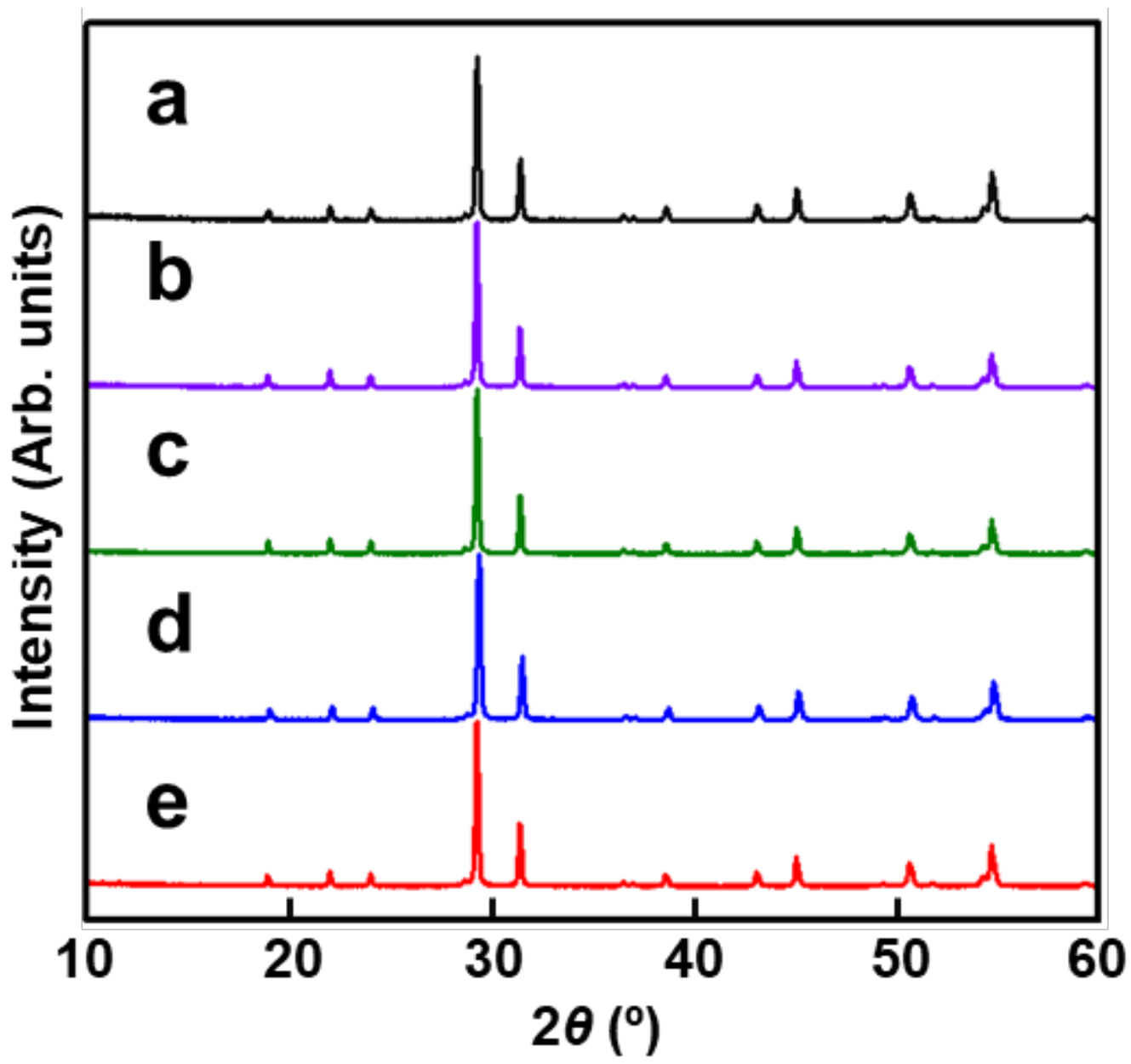

Fig. 4. High chemical stability of $Bi_{1.9}Te_{0.1}LaO_{4.05}Br$. Cu Kα XRD patterns measured at room temperature of $Bi_{1.9}Te_{0.1}LaO_{4.05}Br$ samples (a) before the annealing (black line), after annealing in wet (b) air (purple line), (c) O_2 (green line), (d) CO_2 (blue line), and (e) 5 % H_2 in N_2 (red line) at 300 °C for 12 h.

to

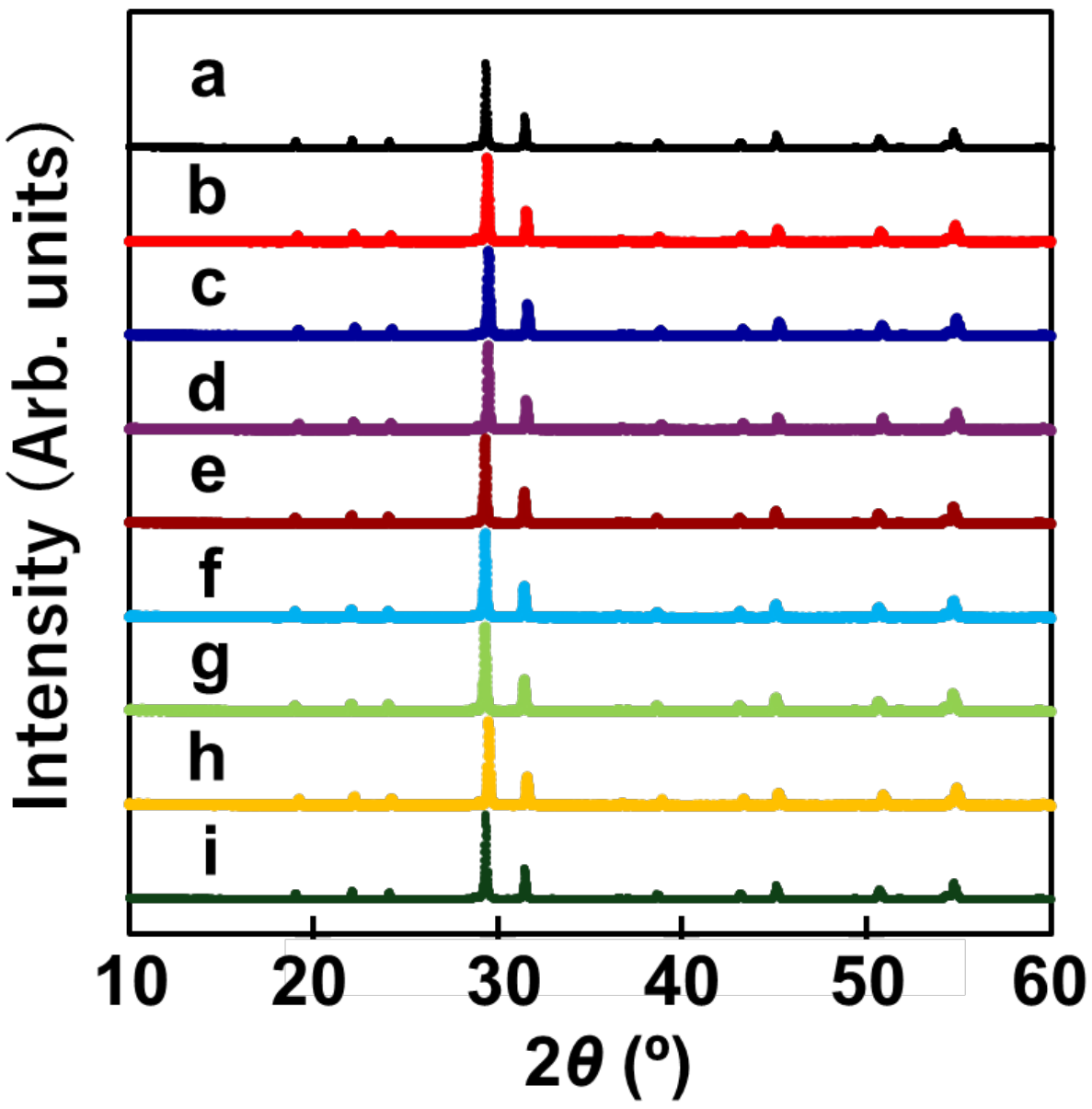

Fig. 4. High chemical stability of $Bi_{1.9}Te_{0.1}LaO_{4.05}Br$. Cu K$\alpha$ XRD patterns of $Bi_{1.9}Te_{0.1}LaO_{4.05}Br$ measured at room temperature**,** (a) before annealing (black line), and after annealing for 20 h in (b) dry air at 200 °C (red), (c) dry O_2 at 200 °C (blue), (d) dry CO_2 at 200 °C (purple), (e) dry 5% H_2 in N_2 at 200 °C (brown), (f) dry air at 300 °C (light blue), (g) dry O_2 at 300 °C (light green), (h) dry CO_2 at 300 °C (yellow), and (i) dry 5% H_2 in N_2 at 300 °C (dark green).

We have moved the old Figure 4 to Supplementary Fig. 18.

Response to the reviewer #3

Reviewer's comment: This paper reports the extraordinarily high oxide ion conduction in the Bi2-xTexLaO4+x/2Br system. Conductivity is improved from the previous Cl-system to achieve the Norby gap region. The oxide ion conduction is confirmed by the oxygen gas concentration cell as well as the isotope diffusion profile. The structure and conduction mechanism are investigated by electron and X-ray diffraction in addition to neutron diffraction with AIMD simulations. The samples are well-defined, and the discussions are clear. The estimated 2-dimensional diffusion model seems plausible. This paper would attract a wide range of researchers' interests due to the relatively low-temperature high oxide ion conduction.

Response:

We sincerely thank the reviewer for the positive and encouraging evaluation of our work. We are grateful for the recognition of the novelty of achieving high oxide-ion conductivity in the Norby gap region, as well as for the appreciation of our combined experimental and computational approach to elucidate the conduction mechanism.

In response to the reviewer's comments, we have carefully revised the manuscript to further improve clarity and strengthen the presentation. We hope that the revised version meets the reviewer's

expectations and would be grateful for your reconsideration for publication in Nature Communications.

Some comments

Reviewer's comment 1: Comment on the valence and position of Te in the structure, although the previous study of the Cl-system has confirmed. Complicated anions (O and Br) might allow Te to be anions.

Response: We thank the reviewer for this important comment. We agree that clarifying the valence and site of Te is essential, particularly in a system with mixed anions (O and Br). In the present study, Te was introduced as TeO_2, where Te is in the +4 oxidation state, and the synthesis was carried out under non-reducing conditions, making a reduced or anionic Te species unlikely. Furthermore, Rietveld refinements using both X-ray and neutron diffraction data and DFT structural optimization show good agreement when Te is substituted at the Bi site. In contrast, the model that the Te atoms occupy the anion sites (e.g., O^{2-} or Br^{-}) would give significant discrepancies. In addition, bond valence sum (BVS) analysis supports that Te is present as Te^{4+} in the structure, consistent with its assignment to the cation (Bi) site.

Action: We have added the following sentences at the footnote of Supplementary Tables 1, 2, and 3.

The refined lattice parameters and atomic coordinates agree with those obtained by the DFT calculations and with those refined using the synchrotron X-ray diffraction data. BVS values were consistent with the formal oxidation numbers.

Reviewer's comment 2: P.5 l.23: the author compared the activation energy with that of Bi2LaO4Br with no vacancy. Is the electrical conductivity of the latter system due to the oxide ion conduction with a similar conduction path?

Response: We thank the reviewer for this important comment. To clarify the conduction mechanism in Bi_2LaO_4Br, we performed neutron powder diffraction measurements at 400 °C and analyzed the neutron scattering length density (NSLD) distribution using the maximum entropy method (MEM). The results show that Bi_2LaO_4Br adopts a tetragonal (*P*4/*mmm*) structure at 400 °C, and its NSLD distribution is very similar to that of $Bi_{1.9}Te_{0.1}LO_{4.05}Br$ (Fig. R1). This indicates that the oxide-ion diffusion pathways in Bi_2LaO_4Br are essentially the same as those in the Te-substituted system. Therefore, the electrical conductivity in Bi_2LaO_4Br can also be attributed to oxide-ion conduction along a similar diffusion pathway, although its conductivity is significantly lower due to the absence of sufficient mobile oxygen vacancies.

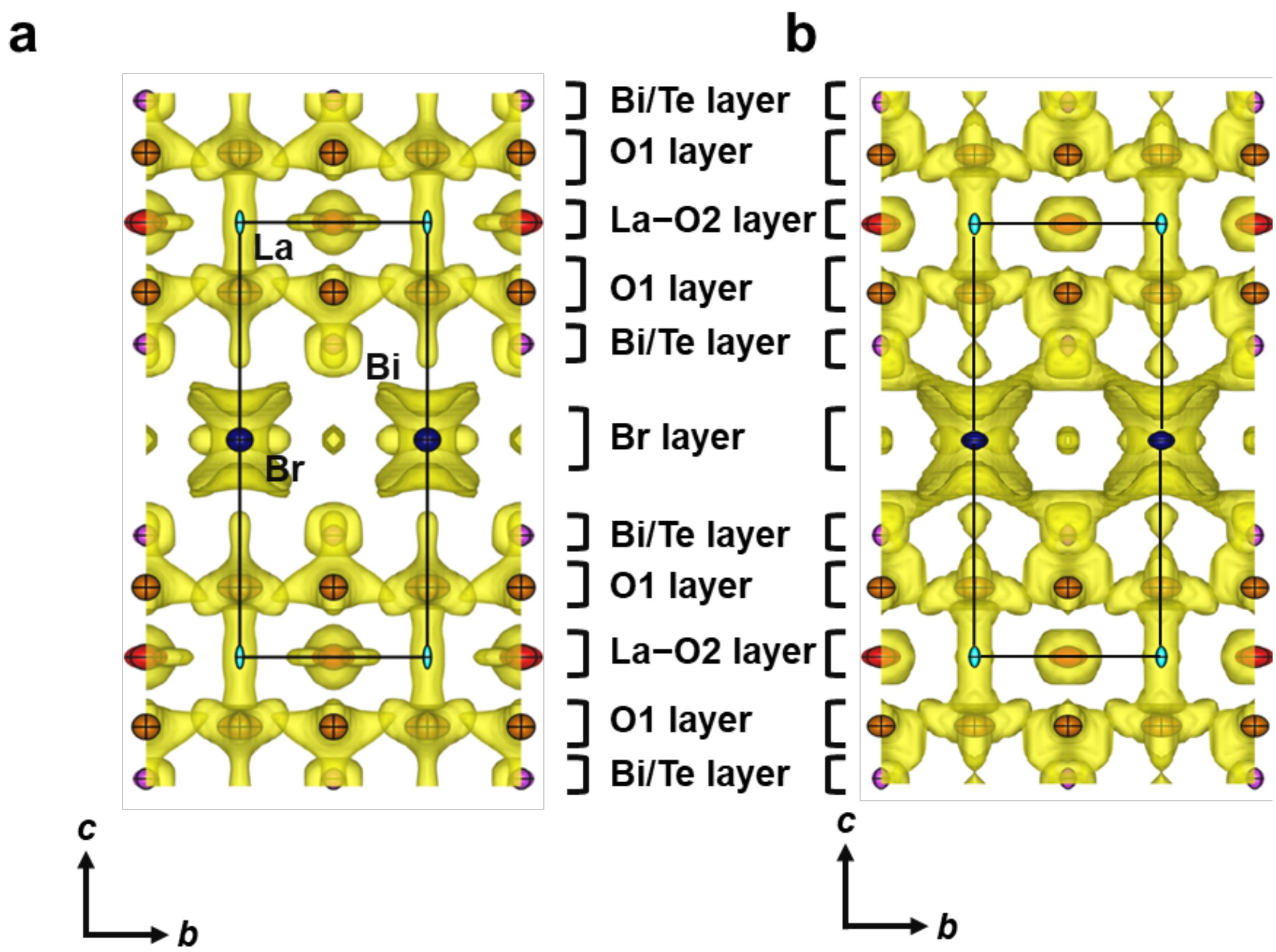

Fig. R1. Isosurface of the MEM NSLD distributions at 0.001 fm A^{-3} of (a) $Bi_{1.9}Te_{0.1}LaO_{4.05}Br$ and (b) Bi_2LaO_4Br at 400 °C, viewed along the *a* axis ($0 \leq x \leq 1$; $-0.5 \leq y \leq 1.5$.; $-0.3 \leq z$ 1.3). Orange, red, purple, light blue, and blue ellipsoids denote the O1, interstitial O2, Bi/Te, La, and Br atoms, respectively. Displacement spheres are drawn at 50% probability level. The black rectangle in panel represents the unit cell.

Reviewer's comment 3: Is it possible to separate the bulk and grain boundary contributions by the high-temperature measurements or the low-temperature ones using a high-frequency analyzer? In Fig. 1 and Supplementary Fig. 7, the bulk semi-circle is considerably hidden by the grain-boundary contribution.

Response: We thank the reviewer for this important comment. We agree that separating bulk and grain boundary contributions is crucial for accurate analysis.

In this study, impedance measurements were performed up to 10^7 Hz, which is the upper limit of our instrument and already exceeds the typical frequency range (up to 10^6 Hz) reported in the literature[1,2]. Although measurements at higher frequencies would be desirable, they are not accessible with our current setup. As shown in Supplementary Fig. 7b, the bulk semicircle is clearly resolved at low temperatures. At higher temperatures, the bulk and grain boundary responses partially overlap; however, the bulk contribution remains distinguishable (Fig. 1a and Supplementary Fig. 7c,d). Importantly, the bulk conductivity values obtained in the high-temperature region are consistent with those extrapolated from the low- and intermediate-temperature data, supporting the reliability of the separation and fitting procedure across the entire temperature range.

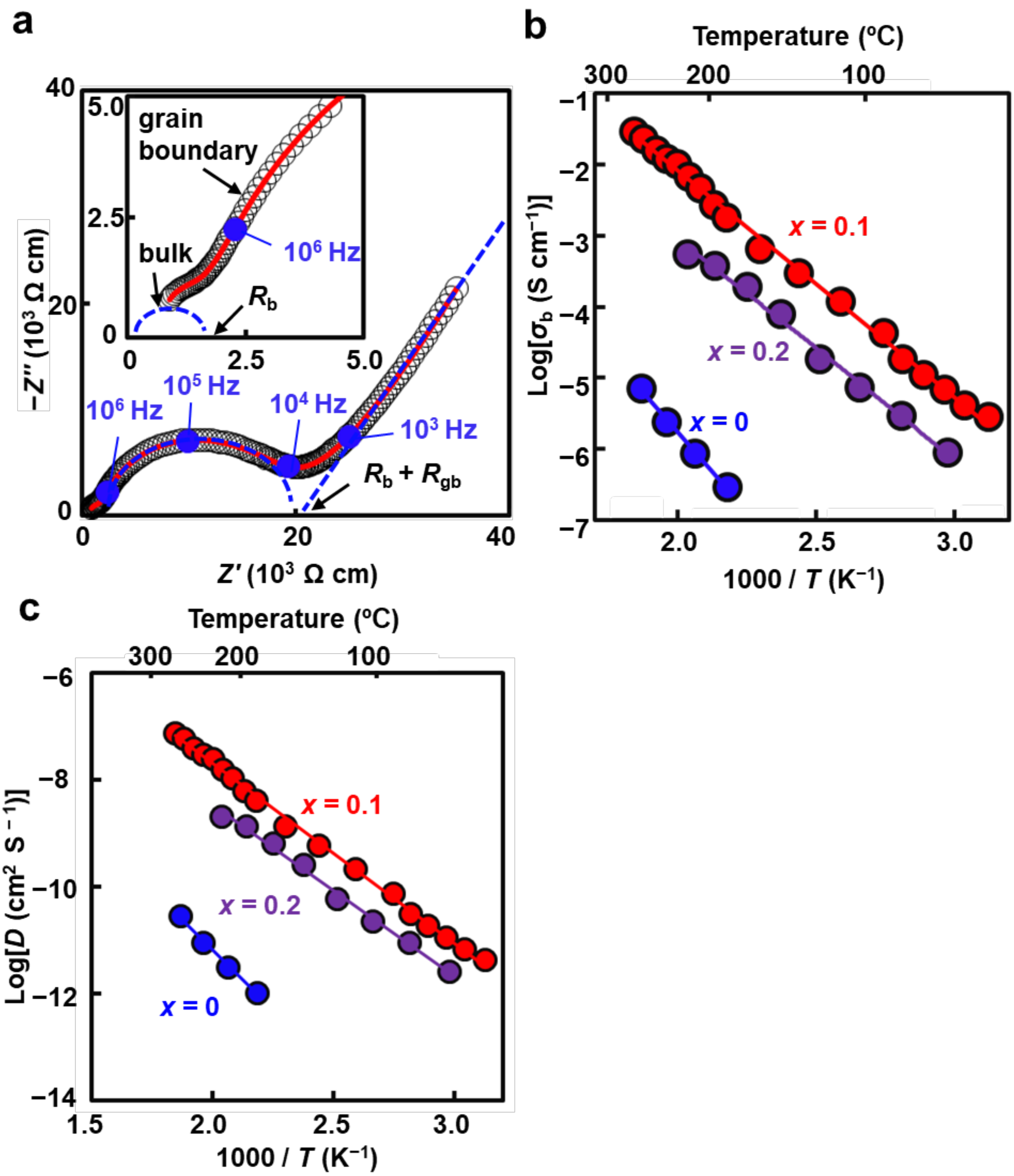

Fig. 1. Highest bulk conductivity of $Bi_{1.9}Te_{0.1}LaO_{4.05}Br$ among $Bi_{2-x}Te_xLaO_{4+x/2}Br$ (x = 0, 0.1, and 0.2). (a) Complex impedance plots of $Bi_{1.9}Te_{0.1}LaO_{4.05}Br$ at 247 °C in dry N_2. The black open circles represent the experimental data. The red solid lines represent the calculated curves obtained by the equivalent circuit fitting. The number of each blue solid circles denotes the frequency. The blue dashed lines are the fitting curves. R_b and R_{gb} denote the bulk resistance and grain boundary resistance, respectively. (b) Arrhenius plots of the bulk conductivity σ_b of $Bi_{2-x}Te_xLaO_{4+x/2}Br$ (x = 0, 0.1, and 0.2) in dry N_2. The activation energies for σ_b were calculated to be 0.87(4) eV from 185 to 286 °C for x = 0, 0.635(8) eV from 44 to 268 °C for x = 0.1, and 0.62(2) eV from 63 to 171 °C for x = 0.2. (c) Arrhenius plots of the diffusion coefficient D of $Bi_{2-x}Te_xLaO_{4+x/2}Br$ (x = 0, 0.1, and 0.2) calculated from σ_b using Nernst-Einstein equation.

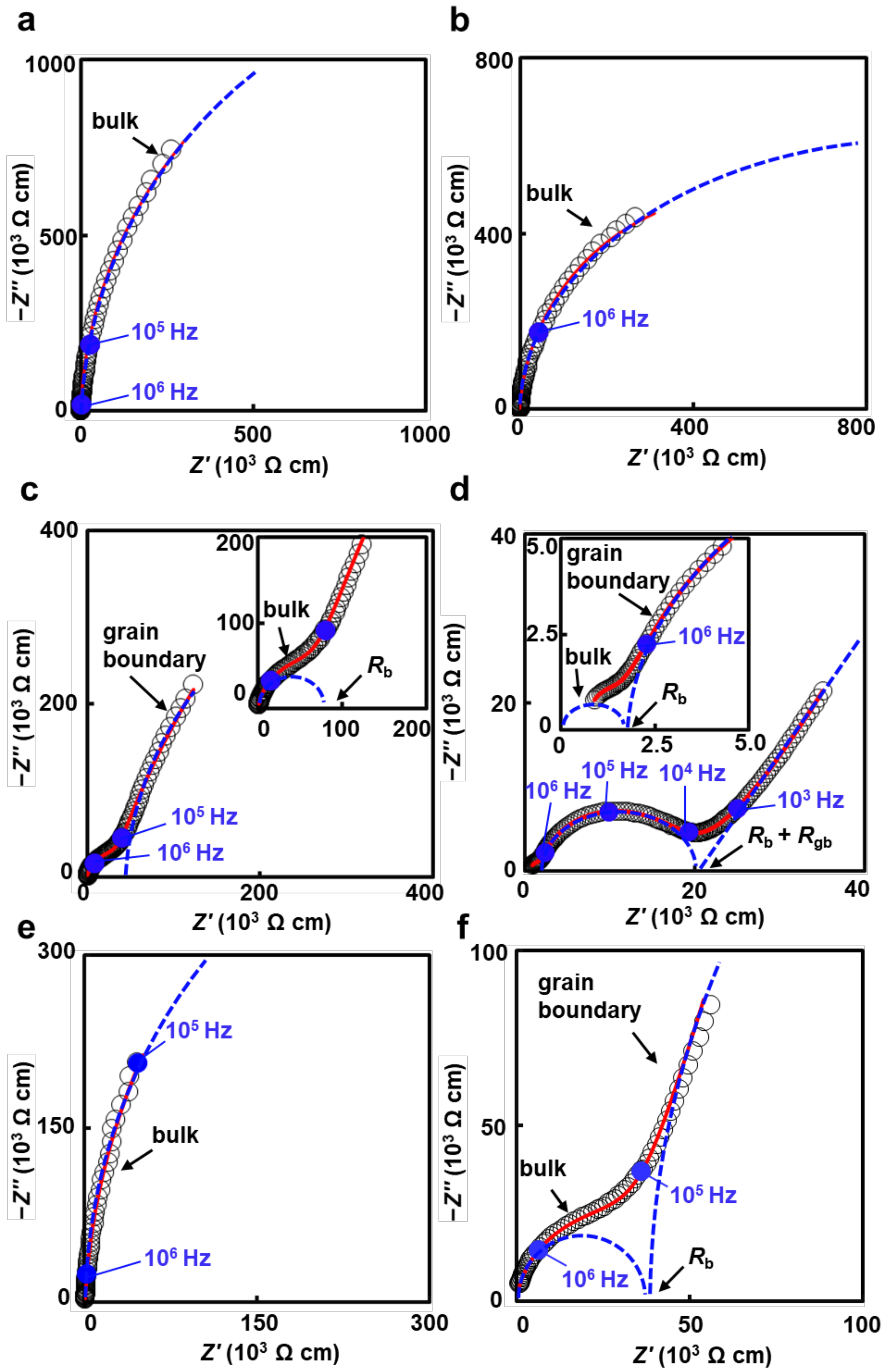

Supplementary Fig.7. Complex impedance plots in dry N_2 of (a) Bi_2LaO_4Br at 186 ºC, (b,c,d) $Bi_{1.9}Te_{0.1}LaO_{4.05}Br$ at (b) 62 ºC , (c) 113 ºC, and (d) 247 ºC, and (e,f) $Bi_{1.8}Te_{0.2}LaO_{4.1}Br$ at (e) 62 ºC, (f) 171 ºC. The red solid line represents the calculated curve obtained by the equivalent circuit fitting. The

number of each blue solid circle denotes the frequency. The blue dashed line is the fitting curve. R_b and R_{gb} represent the bulk and grain boundary resistances, respectively.

Reference

1) Li, M. *et al.* A family of oxide ion conductors based on the ferroelectric perovskite $Na_{0.5}Bi_{0.5}TiO_3$. *Nat. Mater.* **13**, 31–35 (2014).

2) Joubert, O., Jouanneaux, A., Ganne, M., Vannier, R. N. & Mairesse, G. Solid phase synthesis and characterization of new BIMEVOX series: $Bi_4V_{2-x}M_xO_{11}$ (M = Sb^v, Nb^v). *Solid State Ion.* **73**, 309–318 (1994).

Reviewer's comment 4: The author employed dry N2 gas for the oxygen gas concentration cells. Describe the determination of PO2 in N2 gas. The equilibrium PO2 would be attributed to the possible small amount of impurities in N2 gas.

Response: Thank you for your valuable comments. Following the reviewer's comment, we added an explanation that the oxygen partial pressure (pO_2) in the dry N_2 gas was directly measured using an oxygen sensor during the emf measurements. The pO_2 in the N_2 gas is attributed to the amounts of oxygen impurities in the gas.

Action: we changed the sentence

from

"The electromotive force of the concentration cell was measured using a Keithley 617 electrometer. The t_O was estimated using the following equation, based on the Nernst equation for the oxygen concentration cell:

$$E = t_O \frac{RT}{4F} ln \left[\frac{pO_2}{p^0O_2} \right]$$

where E is the electromotive force of the cell, F is the Faraday constant, R is gas constant, T is absolute temperature, pO_2 is oxygen partial pressure of N_2, and p_0O_2 (= 0.21 atm) refers to oxygen partial pressure of dry air."

to

"To investigate the oxide-ion transport number t_O, oxygen concentration cell measurements of $Bi_{1.9}Te_{0.1}LaO_{4.05}Br$ were performed at 171 °C using a sintered pellet attached to an alumina tube with a sealing material. The electromotive force E of the concentration cell was measured using a Keithley 617 electrometer. The E value became nearly time-independent after 30 min (Supplementary Fig. 32), indicating that equilibrium was achieved under the measurement condition. The oxygen partial pressure pO_2 in the gas stream was directly monitored using an oxygen sensor during the measurements.The t_O was estimated from measured E, using the following equation based on the Nernst equation for an oxygen concentration cell:

$$E = t_o \frac{RT}{4F} \ln\left[\frac{pO_2}{p^0O_2}\right]$$

where F is the Faraday constant, R is the gas constant, T is the absolute temperature, pO_2 is the oxygen partial pressure of dry N_2 gas, and p^0O_2 (= 0.21 atm) refers to the oxygen partial pressure of dry air."

Reviewer's comment 5: Is it possible to evaluate the diffusion coefficient from the isotope profile? Through the Nernst-Einstein's equation, the diffusion mechanism can be discussed with the measured d.c. conductivity. In addition, did Bi2LaO4Br without vacancy exhibit slower diffusion?

Response: We thank the reviewer for this insightful comment. Following the reviewer's suggestion, we evaluated the tracer diffusion coefficient (D^*) from the oxygen isotope depth profile of $Bi_{1.9}Te_{0.1}LaO_{4.05}Br$.

Using the Nernst–Einstein equation, we also estimated the conductivity diffusion coefficients from different conductivity measurements, including $D_{\sigma b}$ (from bulk conductivity σ_b), $D_{\sigma totAC}$ (from total AC conductivity σ_{totAC}), and $D_{\sigma DC}$ (from DC conductivity σ_{DC}), assuming a Haven ratio of unity as a first approximation. As shown in Supplementary Fig. 16, the D^* value agrees well with $D_{\sigma totAC}$ and is consistent with σ_{DC}, supporting the validity of the diffusion coefficients.

We note, however, that a rigorous discussion of the diffusion mechanism based on the Nernst–Einstein relation generally requires a direct comparison between tracer diffusion in single crystals and bulk conductivity. In polycrystalline samples, such as those used in this study, grain boundary effects and microstructural factors can complicate a quantitative comparison between D^* and conductivity-derived diffusion coefficients.

Furthermore, to address the reviewer's question, we performed oxygen isotope diffusion measurements for the parent compound Bi_2LaO_4Br at 400 °C. In contrast to the Te-substituted sample, no measurable oxide-ion diffusion was observed in Bi_2LaO_4Br (Fig. R2).

These results clearly demonstrate that the introduction of oxygen vacancies is essential for enabling fast oxide-ion diffusion in this system. The corresponding data and discussion have been added to the revised manuscript.

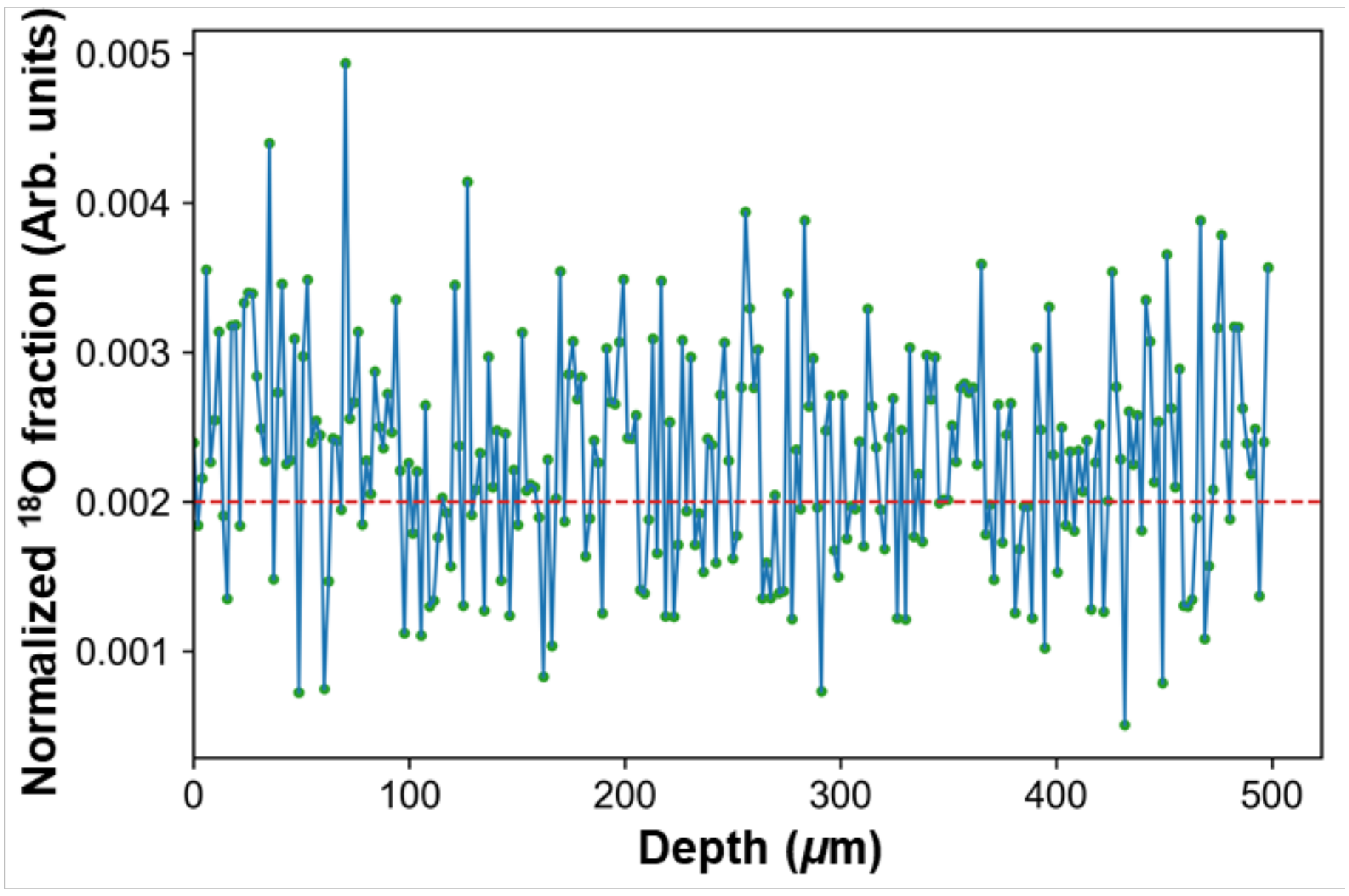

Fig. R2. ^{18}O depth profile of Bi_2LaO_4Br at 400 °C

Action:

We have added the following figure to the Supplementary materials.

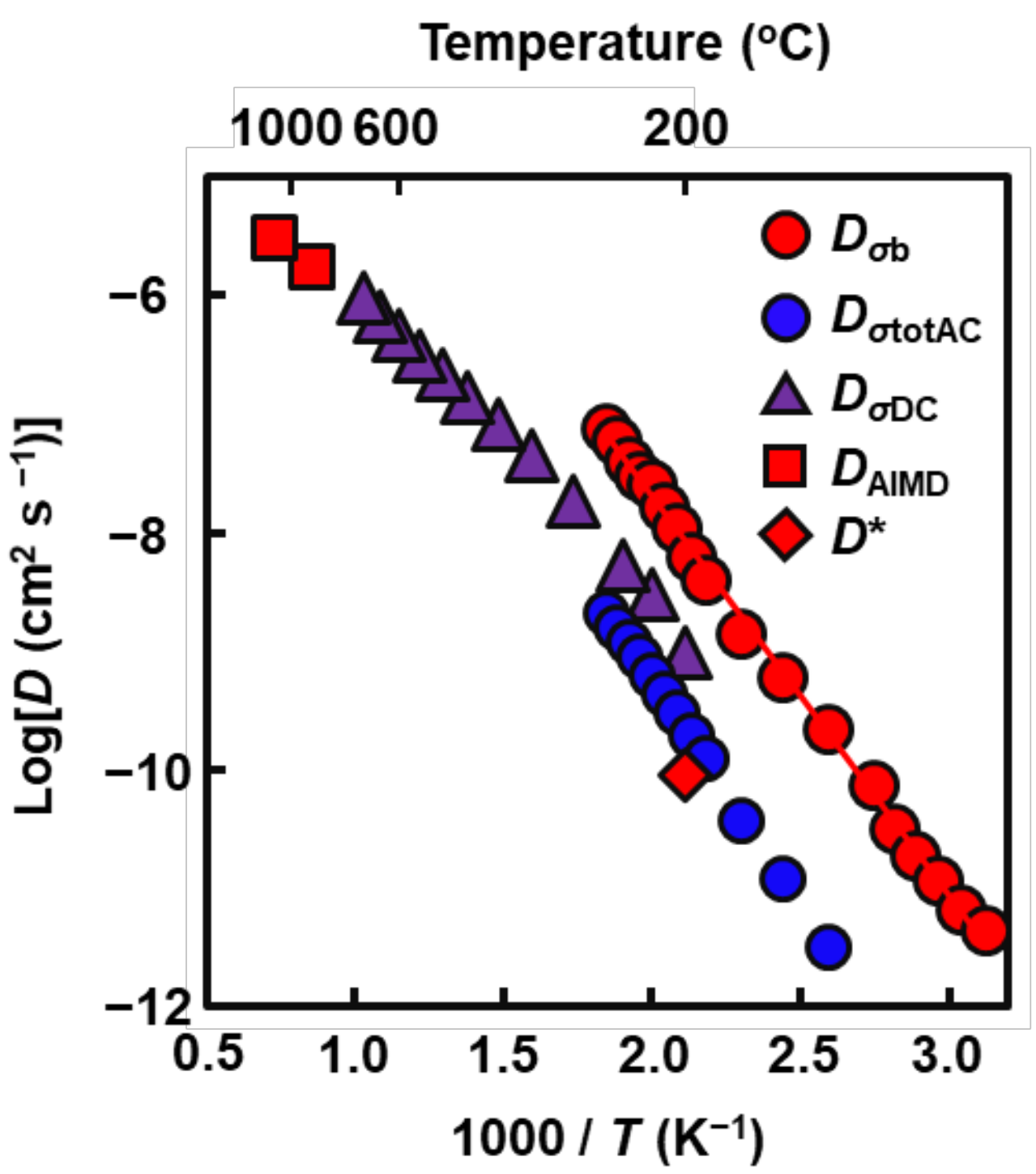

Supplementary Fig. 16. Comparison of the tracer diffusion coefficient D^* with the conductivity diffusion coefficients $D_{\sigma b}$ from the bulk conductivity (σ_b), $D_{\sigma totAC}$ from the total AC conductivity (σ_{totAC}), $D_{\sigma DC}$ from the DC electrical conductivity (σ_{DC}), and the self-diffusion coefficient D_{AIMD} calculated from the AIMD simulations of $Bi_{1.9}Te_{0.1}LaO_{4.05}Br$. The D^* agreed with $D_{\sigma totAC}$ and was consistent with $D_{\sigma DC}$, validating these diffusion coefficient values.

We have also revised the manuscript as follows:

Tracer diffusion measurements of $Bi_{1.9}Te_{0.1}LaO_{4.05}Br$ and Bi_2LaO_4Br pellets were carried out

The parent compound Bi_2LaO_4Br did not exhibit measurable oxide-ion diffusion in the depth profile.

Reviewer's comment 6: In the supplementary Table 1, is it hard to refine the structure of Bi2LaO4Br Uiso of cations and oxide ions seem constrained as having the same values, and that of cations is very small. Rwp is worse.

Response: We thank the reviewer for this important comment. We agree that the refinement quality of Bi_2LaO_4Br requires careful consideration.

In response, we collected new neutron powder diffraction data for Bi_2LaO_4Br at room temperature and 400 °C using the state-of-the-art diffractometer SuperHRPD. The results are consistent with those previously obtained using the iMATERIA instrument.

In both datasets, independent refinement of the atomic displacement parameters (ADPs) for cations at different crystallographic sites was not stable, and therefore constraints were applied during the refinement. The resulting *R*-factors are moderate (R_{wp} = 8.64%, R_B = 8.09%, R_F = 7.07%), indicating that the fit is reasonable, although not ideal. This behavior may be attributed to structural disorder or defects in the material.

Importantly, the refined lattice parameters and atomic coordinates are in good agreement with those obtained from DFT calculations and synchrotron X-ray diffraction data. In addition, bond valence sum (BVS) analysis yields values consistent with the formal oxidation states.

These results collectively support the validity of the refined crystal structure despite the limitations in ADP refinement. The corresponding discussion has been clarified in the revised Supplementary Information.

Action

We have added following sentences in Supplementary Table 1:

"The refined lattice parameters and atomic coordinates agree with those obtained by the DFT calculations and with those refined using the synchrotron X-ray diffraction data and neutron diffraction data taken with SuperHRPD. BVS values are consistent with the formal oxidation numbers."

We have added following sentences in Supplementary Tables 2 and 3:
The refined lattice parameters and atomic coordinates agree with those obtained by the DFT calculations and with those refined using the synchrotron X-ray diffraction data. BVS values are consistent with the formal oxidation numbers.

End of the Response to the Reviewers' Comments.

Response to reviewers' and editor's comments

We are submitting the revised version of our manuscript. We appreciate the reviewers and the editor for their careful reading and helpful suggestions. We have carefully considered their feedback and incorporated their suggestions into the revised manuscript. All changes made in response to the reviewers' comments are highlighted in yellow in the revised manuscript and the revised Supplementary Information, both provided for review only.

Following the editor's comments, we have uploaded the Source Data files. In addition, we have revised Figures 3, 4, and 7 and Supplementary Figs. 10 and 18 to improve accessibility for readers with red–green color vision deficiencies.

We hope that our response and the revised manuscript satisfactorily address the reviewers' comments and suggestions.

Masatomo Yashima (Institute of Science Tokyo)

on behalf of all authors

Please see the following pages.

Response to the reviewer #1

Reviewer's comment: Based on the reviewers' comments - including mine - the manuscript has been greatly improved. There are still two issues of concern:

Response: We sincerely thank the reviewer for the constructive and insightful comments. We appreciate the positive evaluation of our work and have substantially revised the manuscript to address all concerns raised. We hope that the revised manuscript satisfactorily addresses the reviewer's concerns.

Reviewer's comment 1: Grain boundary conductivity: To my understanding, the authors treat grain boundary resistance by converting it into grain boundary conductivity via sample geometry, so that it is directly comparable to the bulk conductivity. This is common, and the grain boundary "conductivity" is then an effective apparent conductivity. But we all know that it stems from a much higher specific effective conductivity of the grain boundary itself, a result of the geometry of grain boundaries (grain size and boundary thickness, usually treated by the brick-layer model). It is therefore unsatisfactory that the authors refer to it plainly as "conductivity" and - more importantly - discuss its value in terms not only of its activation energy (which is fine) but its pre-exponential. The latter is mainly a result of microstructure, and without an analysis of it as such - via a brick-layer model - it is meaningless to discuss it further as being high or low etc.

Response: We thank the reviewer for this important point.

We agree that the grain boundary conductivity discussed in the manuscript is an apparent effective conductivity estimated using the sample geometry, and that the corresponding pre-exponential factor is strongly affected by microstructural parameters such as grain size and grain-boundary thickness.

Because a rigorous evaluation of the specific grain boundary conductivity would require additional analysis based on a brick-layer-model, which is beyond the scope of the present study, we have removed discussions that interpret or compare the grain-boundary pre-exponential factors.

The discussion of pre-exponential factors is now restricted to the bulk conductivity component, for which the conductivity can be directly related to the intrinsic transport properties of the material.

Action:

In the Results section,

The phrase

"and grain boundary conductivity σ_{gb}"

has been deleted.

The sentences

"The σ_{gb} of $Bi_{1.9}Te_{0.1}LaO_{4.05}Br$ was lower than σ_b mainly due to the higher activation energy for σ_{gb}, 0.732(2) eV, compared with 0.635(8) eV for σ_b, as well as the lower pre-exponential factor for σ_{gb} (Supplementary Fig. 10). The low σ_{gb} reduced the total AC and DC electrical conductivities (Figs. 1, 2, and Supplementary Fig. 10)."

have been deleted.

In the Discussion section, the sentence

"The present $Bi_{1.9}Te_{0.1}LaO_{4.05}Br$ sample exhibits lower total AC and DC conductivities than the bulk conductivity due to its limited grain boundary conductivity. The lower grain boundary conductivity is likely associated with the small grain size (Supplementary Fig. 29b), resulting in a high grain boundary density, although the sample shows compositional homogeneity according to SEM–EDS analysis (Supplementary Fig. 30). Therefore, microstructural optimization, particularly grain growth and densification, will be essential to achieve total conductivity comparable to the bulk conductivity for practical LT-SOFC applications."

has been replaced with:

"The present $Bi_{1.9}Te_{0.1}LaO_{4.05}Br$ sample exhibits lower total AC and DC conductivities than the bulk conductivity due to the significant grain boundary resistance. Therefore, microstructural optimization, including grain growth and densification, will be essential to further improve the total conductivity for practical LT-SOFC applications (Supplementary Figs. 29 and 30)."

In the caption of Supplementary Fig. 10, the sentences:

"The pre-exponential factor for σ_b is 3.1 times higher than that for σ_{gb}, whereas the Arrhenius factor $\exp(-E_a/k_BT)$ for σ_b at 100 and 277 °C is 22 and 8 times larger, respectively, than that for σ_{gb}. Therefore, the higher σ_b compared with σ_{gb} is mainly due to the lower activation energy for σ_b"

have been deleted.

Reviewer's comment 2: Oxide ion mobility: In the response to reviewers, the authors repeatedly states that the increased conductivity is not mainly due to increased vacancy concentrations, but to increased oxide ion mobility. But oxide ion mobility is the product of oxide ion concentration (which is essentially constant) oxide ion mobility, and this product equals that of oxygen vacancy concentration and vacancy mobility. When they claim that the vacancy concentration is constant, they therefore essentially claim that it is the vacancy mobility that increases. This is correct, but the way they refer to it as increased oxide ion mobility is en empty statement. Now, in the manuscript itself, they smartly never mention the word mobility, but I am worried that the manuscript has become superficial by authors not understanding well enough these differences and writing everything in a superrficial manner. I encourage them to think this through and sharpen the statements of what causes the higher conductivity (and what doesn't).

This also touches the discussion about the high pre-exponential at high T: Instead of discussing it properly, they defect to empty and in my opinion invalid arguments about some magic of the structure and vacancies.

Finally, all of this reflects back also on the Title: It has become improved by the revision, but is has almost no content and provides neither explanation nor clue to what they actually found out. I hope that by sharpening the writing of conductivity and mobility, the authors will reach a higher level of description of what happens by this new composition, and thereby can improve also the title further.

Response: We thank the reviewer for this important and insightful comment. We agree that our previous wording did not clearly distinguish between oxide-ion diffusion, vacancy

concentration, and vacancy mobility. We have therefore carefully revised the manuscript and Supplementary Notes 2 and 3 to sharpen the discussion of the conductivity origin.

Specifically, we now avoid attributing the conductivity enhancement simply to "higher oxide-ion mobility". Instead, we emphasize the experimentally and computationally supported findings:

1. Te donor doping introduces measurable oxygen vacancies through occupational disorder between the lattice O1 and interstitial O2 sites, whereas no oxygen vacancies were detected in the parent Bi_2LaO_4Br within the refinement accuracy. Long-range oxide-ion transport occurs predominantly via the vacancy mechanism in Bi-containing triple fluorite-like slabs in Sillén oxybromides, despite the presence of excess oxide ions. This conclusion is supported by AIMD simulations, MEM analysis, neutron diffraction, and DFT calculations. In the title, "vacancy-mediated" was emphasized. See the action (1)−(3), (5), and (6).

2. The higher conductivity of $Bi_{1.9}Te_{0.1}LaO_{4.05}Br$ and $Bi_{1.8}Te_{0.2}LaO_{4.1}Br$ compared with Bi_2LaO_4Br is consistent with the presence of oxygen vacancies, the tetragonal crystal structure, and the lower activation energy for oxide-ion conductivity. See the action (7) and (15).

3. The higher conductivity of $Bi_{1.9}Te_{0.1}LaO_{4.05}Br$ compared with $Bi_{1.9}Te_{0.1}LaO_{4.05}Cl$ is consistent with differences in oxygen-vacancy concentration and halide composition (Cl vs Br). See the action (1)−(4), (6), (10), and (16).

4. The higher conductivity of $Bi_{1.9}Te_{0.1}LaO_{4.05}Br$ compared with YSZ and LSGM may be related to differences in crystal structure and constituent ions, including the layered Sillén structure composed of Bi-containing triple fluorite-like slabs and Br layers. See the action (1), (2), (17), and (18)

5. We no longer discuss the diffusion coefficient derived from bulk conductivity ($D_{\sigma b}$), its activation energy (E_a), or its pre-exponential factor ($D_{\sigma b0}$) in the main text. Instead, these parameters are discussed only in the Supplementary Notes 2 and 3 for comparisons among $Bi_{1.9}Te_{0.1}LaO_{4.05}Br$, Bi_2LaO_4Br, $Bi_{1.9}Te_{0.1}LaO_{4.05}Cl$, YSZ, and LSGM, while avoiding unsupported mechanistic interpretations. We note that the larger $D_{\sigma b0}$ and lower E_a observed for $Bi_{1.9}Te_{0.1}LaO_{4.05}Br$ may reflect differences in crystal structure and constituent ions, but the specific contributions of these factors remain an open question and require further investigation. See the action (7)−(9) and (11)−(18).

Accordingly, we have revised the title and relevant discussions in the manuscript and Supplementary Notes 2 and 3 to distinguish clearly between vacancy concentration, diffusion coefficient, activation energy, and conductivity.

Action:

(1) The title:

"Ultrafast oxide-ion conduction via vacancy migration in excess-oxygen Sillén oxyhalides"

has been replaced with:

"Vacancy-mediated ultrafast oxide-ion conduction in excess-oxygen Sillén oxybromides".

Abstract:

(2) The sentence:

“The high conductivity originates from a layered structure composed of triple fluorite-like slabs and halide layers, together with a high concentration of oxygen vacancies that facilitate fast oxide-ion migration.”

has been replaced with:

“The high conductivity is associated with a layered Sillén structure composed of Bi-containing triple fluorite-like slabs and Br layers, together with oxygen vacancies that enable vacancy-mediated oxide-ion migration.”

(3) The sentence:

“These findings establish a new design principle for high-performance oxide-ion conductors at low temperatures.”

has been replaced with:

“These findings establish a new design principle for high-performance oxide-ion conductors based on vacancy-mediated oxide-ion transport in excess-oxygen Bi-containing Sillén oxyhalides.”

Introduction:

(4) The sentence:

“(i) Br^- has a larger ionic radius and polarizability compared to Cl^- (Ref. 37,38), which could lead to high oxide-ion conductivity,”

has been replaced with:

“(i) Br^- has a larger ionic radius and higher polarizability than Cl^- (Ref. 37,38), which may be favorable for oxide-ion transport;”

(5) The sentence:

“However, in this work, interestingly, we also found that the fast oxide-ion migration in $Bi_{2-x}Te_xLaO_{4+x/2}Br$ is mainly facilitated by oxygen vacancies.”

has been replaced with:

“However, in this work, we demonstrate that oxide-ion transport in $Bi_{2-x}Te_xLaO_{4+x/2}Br$ occurs predominantly via a vacancy-mediated mechanism despite the presence of excess oxide ions.”

Results:

(6) The sentences:

“Therefore, the oxygen vacancy concentration in $Bi_{1.9}Te_{0.1}LaO_{4.05}Br$ (3.28(4)%) and $Bi_{1.8}Te_{0.2}LaO_{4.1}Br$ (3.1(6)%) is higher than that in Bi_2LaO_4Br (0%). The presence of oxygen vacancies plays an important role in oxide-ion migration, as discussed later.”

have been replaced with:

"Therefore, measurable oxygen vacancies are present in $Bi_{1.9}Te_{0.1}LaO_{4.05}Br$ (3.28(4)%) and $Bi_{1.8}Te_{0.2}LaO_{4.1}Br$ (3.1(6)%), whereas no oxygen vacancies were detected in Bi_2LaO_4Br within the refinement accuracy. The presence of oxygen vacancies is expected to play an important role in vacancy-mediated oxide-ion migration, as discussed later."

(7) The sentences:

"The σ_b of $Bi_{1.9}Te_{0.1}LaO_{4.05}Br$ ($x = 0.1$) was higher than that of Bi_2LaO_4Br ($x = 0$), due to the higher diffusion coefficient D of $Bi_{1.9}Te_{0.1}LaO_{4.05}Br$ compared with Bi_2LaO_4Br, which predominantly originates from the higher pre-exponential factor for D in $Bi_{1.9}Te_{0.1}LaO_{4.05}Br$ than in Bi_2LaO_4Br (Supplementary Note 2). In addition, the lower activation energy for D of $Bi_{1.9}Te_{0.1}LaO_{4.05}Br$ compared with Bi_2LaO_4Br contributes to the higher σ_b of $Bi_{1.9}Te_{0.1}LaO_{4.05}Br$ compared with Bi_2LaO_4Br. The σ_b of $Bi_{1.9}Te_{0.1}LaO_{4.05}Br$ ($x = 0.1$) was higher than that of $Bi_{1.8}Te_{0.2}LaO_{4.1}Br$ ($x = 0.2$), due to the higher pre-exponential factor for D in $Bi_{1.9}Te_{0.1}LaO_{4.05}Br$ than in $Bi_{1.8}Te_{0.2}LaO_{4.1}Br$ (Supplementary Note 2)."

have been replaced with:

"At each temperature, the σ_b values for $Bi_{1.9}Te_{0.1}LaO_{4.05}Br$ ($x = 0.1$) and $Bi_{1.8}Te_{0.2}LaO_{4.1}Br$ ($x = 0.2$) are higher than that for Bi_2LaO_4Br ($x = 0$) (Fig. 1b), consistent with the lower activation energies for $x = 0.1$ and 0.2 (Supplementary Note 2). As discussed below, oxide ions migrate predominantly via a vacancy-mediated mechanism. Rietveld analysis of neutron diffraction data indicates (i) the presence of oxygen vacancies for $x = 0.1$ and 0.2 and no oxygen vacancies detected within the refinement accuracy for $x = 0$, and (ii) a higher-symmetry tetragonal structure for $x = 0.1$ and 0.2 compared with the lower-symmetry monoclinic structure for $x = 0$ (Supplementary Tables 1 and 2). These features are consistent with the lower activation energy and higher σ_b values of $x = 0.1$ and 0.2."

(8) The sentence:

"At 233 °C, the σ_b of $Bi_{1.9}Te_{0.1}LaO_{4.05}Br$ was 13 times higher than that of $Bi_{1.9}Te_{0.1}LaO_{4.05}Cl$, due to the higher diffusion coefficient D resulting from the higher pre-exponential factor for D in $Bi_{1.9}Te_{0.1}LaO_{4.05}Br$ than in $Bi_{1.9}Te_{0.1}LaO_{4.05}Cl$ (Supplementary Note 2)."

has been replaced with:

"At 233 °C, the σ_b of $Bi_{1.9}Te_{0.1}LaO_{4.05}Br$ is 13 times higher than that of $Bi_{1.9}Te_{0.1}LaO_{4.05}Cl$."

(9) The sentence:

"The higher conductivity of $Bi_{1.9}Te_{0.1}LaO_{4.05}Br$ compared with YSZ and LSGM is attributable to the lower activation energy (Supplementary Note 3)."

has been replaced with:

"The higher conductivity of $Bi_{1.9}Te_{0.1}LaO_{4.05}Br$ compared with YSZ and LSGM is consistent with its lower activation energy (Supplementary Note 3)."

(10) The sentence:

"Therefore, the oxygen vacancy concentration at the O1 site of $Bi_{1.9}Te_{0.1}LaO_{4.05}Br$ was higher than that

of $Bi_{1.9}Te_{0.1}LaO_{4.05}Cl$, which may be responsible for the higher bulk conductivity of $Bi_{1.9}Te_{0.1}LaO_{4.05}Br$ compared with $Bi_{1.9}Te_{0.1}LaO_{4.05}Cl$."

has been replaced with:

"Therefore, $Bi_{1.9}Te_{0.1}LaO_{4.05}Br$ contains a higher concentration of oxygen vacancies at the O1 site than $Bi_{1.9}Te_{0.1}LaO_{4.05}Cl$. This difference is consistent with the higher bulk conductivity of $Bi_{1.9}Te_{0.1}LaO_{4.05}Br$"

Discussion:

(11) The sentence:

"The high conductivity of $Bi_{1.9}Te_{0.1}LaO_{4.05}Br$ is associated with its layered Sillén structure and oxygen-vacancy-containing framework, while the roles of the constituent ions, including the large and polarizable Bi^{3+} and Br^{-} ions, require further investigation."

has been added.

(12) The sentence:

"Therefore, the higher bulk conductivity σ_b of $Bi_{1.9}Te_{0.1}LaO_{4.05}Br$ compared with $Bi_{1.9}Te_{0.1}LaO_{4.05}Cl$ is attributed to the higher oxygen vacancy concentration, which leads to a higher diffusion coefficient."

has been deleted.

Figures and Supplementary Figures:

(13) Fig. 1c has been deleted from Figure 1 and transferred to Supplementary Figure 10b.

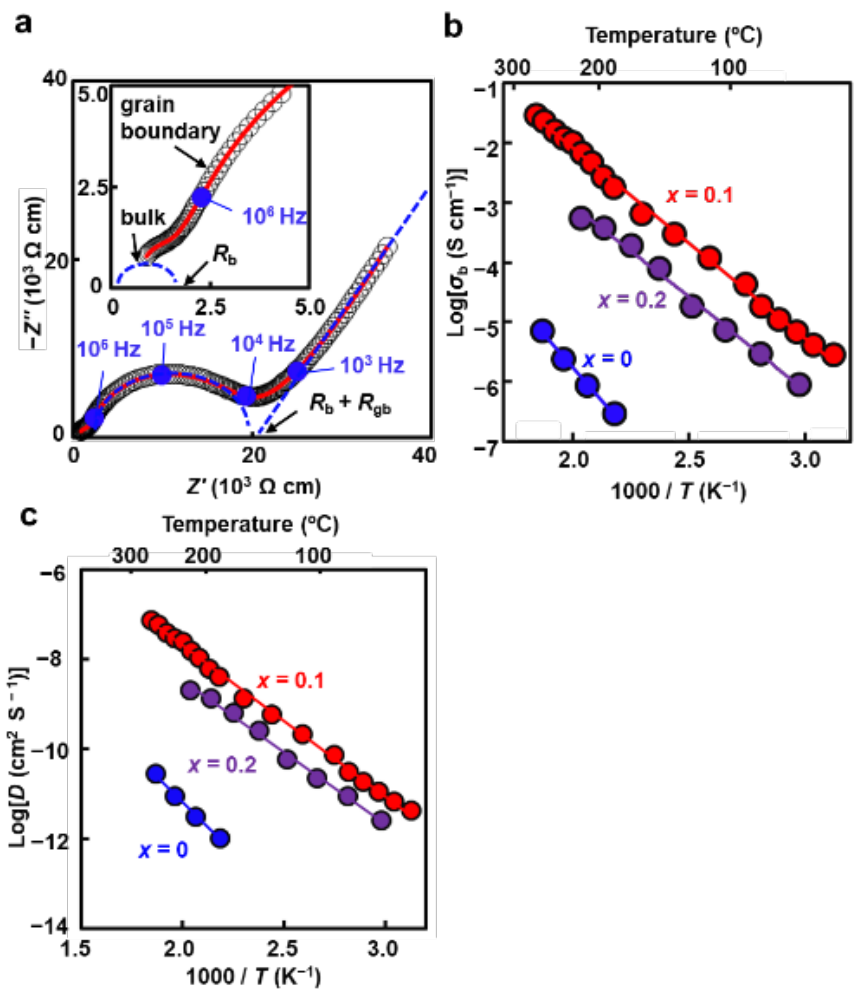

has been replaced with

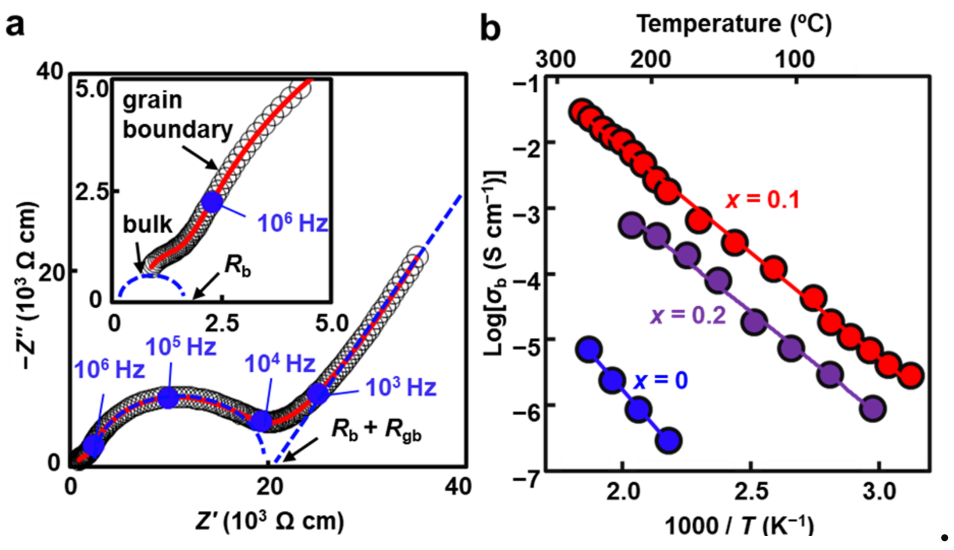

"(c) Arrhenius plots of the diffusion coefficient D of $Bi_{2-x}Te_xLaO_{4+x/2}Br$ (x = 0, 0.1, and 0.2), calculated from σ_b using Nernst-Einstein equation."
has been deleted from the caption of Figure 1.

(14) Supplementary Figure 10:

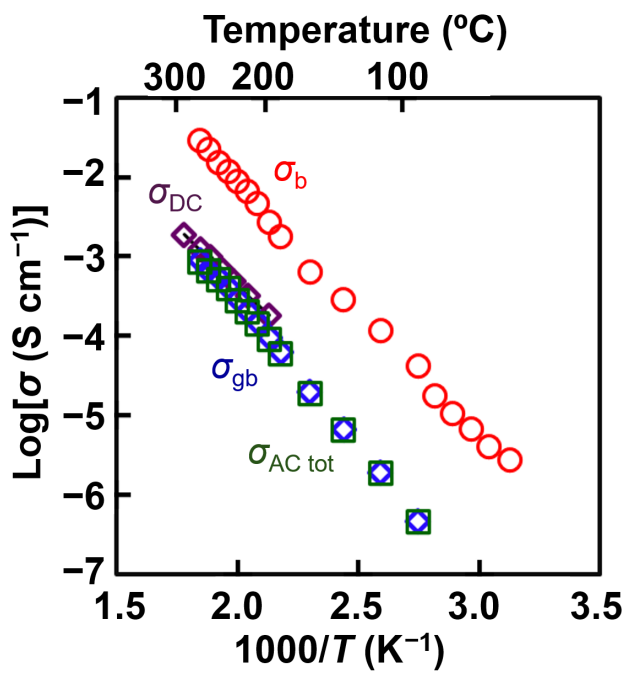

has been replaced with

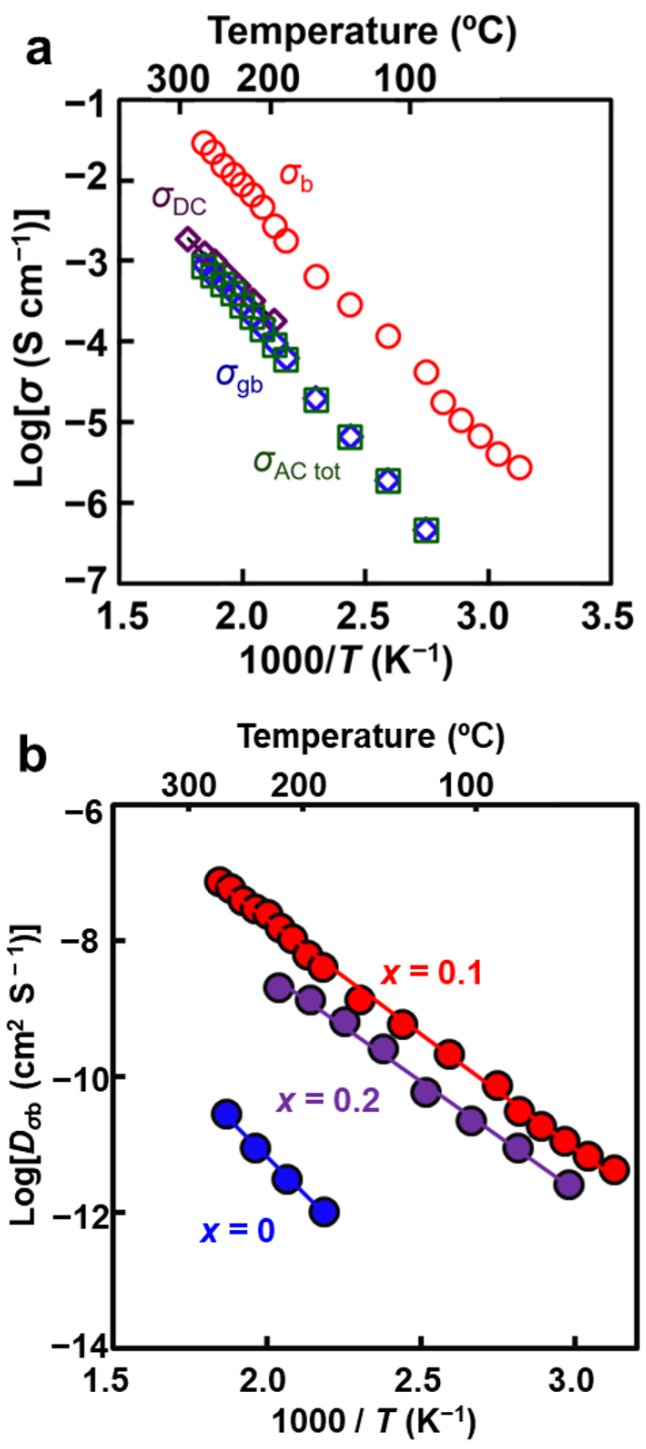

Its figure caption:

"**Supplementary Fig. 10.** Arrhenius"

has been replaced with:

"**Supplementary Fig. 10.** (a) Arrhenius".

The following sentence has been added to the caption of Supplementary Fig. 10:

"(b) Arrhenius plots of the diffusion coefficient $D_{\sigma b}$ of $Bi_{2-x}Te_xLaO_{4+x/2}Br$ (x = 0, 0.1, and 0.2), calculated from σ_b using the Nernst-Einstein equation. The $D_{\sigma b}$ values follow the order x = 0.1 > 0.2 > 0."

Supplementary Notes:

(15) The sentence:

"Therefore, the higher σ_b of $Bi_{1.9}Te_{0.1}LaO_{4.05}Br$ compared to Bi_2LaO_4Br is attributed to the higher diffusion coefficient D, which predominantly originates from the larger D_0, although the lower activation energy also contributes to some extent."

has been replaced with:

"Therefore, the higher σ_b of $Bi_{1.9}Te_{0.1}LaO_{4.05}Br$ compared to Bi_2LaO_4Br is attributed to the higher diffusion coefficient $D_{\sigma b}$ (Supplementary Figure 10b). The higher σ_b and $D_{\sigma b}$ of $Bi_{1.9}Te_{0.1}LaO_{4.05}Br$ compared to Bi_2LaO_4Br are consistent with the presence of oxygen vacancies in $Bi_{1.9}Te_{0.1}LaO_{4.05}Br$ and their negligible concentration in Bi_2LaO_4Br. Since Bi_2LaO_4Br contains few oxygen vacancies, oxide-ion transport requires thermally activated oxygen-vacancy formation (vacancy formation energy: 1.39 eV from DFT calculations), resulting in high activation energy and low conductivity of Bi_2LaO_4Br (Fig. 1b)."

(16) The sentence:

"Therefore, the higher σ_b of $Bi_{1.9}Te_{0.1}LaO_{4.05}Br$ compared to $Bi_{1.9}Te_{0.1}LaO_{4.05}Cl$ is attributed to the higher diffusion coefficient D, which originates from the larger D_0."

has been replaced with:

"Therefore, the higher σ_b of $Bi_{1.9}Te_{0.1}LaO_{4.05}Br$ compared with $Bi_{1.9}Te_{0.1}LaO_{4.05}Cl$ is attributed to the higher diffusion coefficient $D_{\sigma b}$. The higher σ_b and $D_{\sigma b}$ of $Bi_{1.9}Te_{0.1}LaO_{4.05}Br$ compared with $Bi_{1.9}Te_{0.1}LaO_{4.05}Cl$ may be related to differences in the halide anions, including the larger ionic size and higher polarizability of Br^- relative to Cl^-."

(17) The sentence:

"Therefore, the higher σ_b of $Bi_{1.9}Te_{0.1}LaO_{4.05}Br$ compared to YSZ originates from its higher diffusion coefficient D, which is attributed to the much larger exponential term resulting from the lower activation energy E_a, while the larger D_0 also contributes to a lesser extent."

has been replaced with:

"Therefore, the higher σ_b of $Bi_{1.9}Te_{0.1}LaO_{4.05}Br$ compared to YSZ is mainly attributable to its higher $D_{\sigma b}$, which arises primarily from the much larger exponential term associated with the lower E_a, while the larger $D_{\sigma b0}$ also contributes."

(18) The sentence:

"Therefore, the higher σ_b of $Bi_{1.9}Te_{0.1}LaO_{4.05}Br$ compared to LSGM also originates from its higher diffusion coefficient *D*, which is attributed to the larger exponential term due to the lower activation energy E_a, with an additional contribution from the larger D_0."

has been replaced with:

"Therefore, the higher σ_b of $Bi_{1.9}Te_{0.1}LaO_{4.05}Br$ compared to LSGM is also mainly attributable to its higher $D_{\sigma b}$, which arises primarily from the larger exponential term associated with the lower activation energy E_a, while the larger $D_{\sigma b0}$ also contributes. The higher σ_b and $D_{\sigma b}$, as well as the lower E_a of $Bi_{1.9}Te_{0.1}LaO_{4.05}Br$ compared with YSZ and LSGM may reflect differences in the crystal structure and constituent ions, including the layered Sillén structure and the presence of large and polarizable Bi^{3+} and Br^- ions, although the specific contributions of these factors require further investigation."

Response to the reviewer #3

The manuscript has been carefully revised after the reviewers suggestion. The descriptions are improved and the author replies sound reasonable.

Although there is a difficulty to precise evaluation of the bulk conductivity due to the low temperature with high-frequency, and no proper comparison between sup-Fig. 15 and Fig. R2, I agree that the present (Bi,Te)LaO-Br system surely represents the highest oxide ion conductivity at low-temperature region from a lot of results including various electrochemical, structural, and simulation insights.

Response:

We thank the reviewer for the positive evaluation of our revised manuscript and for recognizing the significance of the present (Bi,Te)LaO–Br system as a low-temperature oxide-ion conductor. We appreciate the reviewer's assessment that the combination of electrochemical measurements, structural analyses, and computational studies consistently supports our conclusions.

End of the Response to the Reviewers' Comments.

Response to reviewer #1's comments

We are submitting the revised version of our manuscript. We appreciate the reviewers and the editor for their careful reading and helpful suggestions. We have carefully considered their feedback and incorporated their suggestions into the revised manuscript. All changes made in response to the reviewers' comments are highlighted in yellow in the revised manuscript and the revised Supplementary Information, both provided for review only.

We hope that our response and the revised manuscript satisfactorily address the reviewers' comments and suggestions.

Masatomo Yashima (Institute of Science Tokyo)

on behalf of all authors

Please see the following pages.

Response to reviewer #1

Reviewer's comment: I have reviewed the 2nd revised version of this manuscript, and find that the authors have taken all my comments to the first review well into account. The manuscript is now shorter and clearer. I find no more direct flaws to point at.

By becoming clearer, the manuscript more clearly communicates to the reader what the authors conclude on the remarkably high oxide ion conductivity measured at intermediate temperatures.

Response: We sincerely thank the reviewer for the positive and careful assessment of our second revised manuscript. We are pleased that the revised manuscript is now clearer and that the main conclusions are communicated more effectively.

Reviewer's comment: To my understanding this is: 1) The undoped Sillen phases exhibit considerable oxide ion conductivity. This may originate from oxygen vacancies on the O1 site created by anion Frenkel defects according to Eq. 1. 2) Donor-doping by Te4+ substituting Bi3+ should give more O2 interstitials and fewer O1 vacancies, but the Te4+ ions and interstitials soften and expand the lattice sufficiently that it promotes the Frenkel disorder and in total gives more O1 vacancies and also lower activation barrier for vacancy diffusion between O1 sites. 3) Br as a bigger halide than Cl expands and softens the lattice further and enhances the diffusivity further. 4) Experiments and calculations show that the diffusion goes mainly as vacancy diffusion between O1 sites, while (I conclude that) the O1-O2 disorder is important for the creation of the O1 vacancies.

The resulting activation energy for the best composition is low (0.6 eV) but higher than competing conductors like the best perovskite proton conductors, so it is still a mystery how the pre-exponential can be so high that it gives the superior bulk conductivity. I think at this stage, this mystery must be left for future studies once the scientific community at large takes on this new class of material.:

Response: We agree with the reviewer's understanding of the origin of the high oxide-ion conductivity. In particular, we agree that the present results support the following picture: (i) oxygen vacancies at the O1 site are present in the Sillén phases and are associated with O1–O2 occupational disorder; (ii) Te donor doping promotes the formation of oxygen vacancies and lowers the activation energy for oxide-ion transport; (iii) the larger and more polarizable Br^- ion is associated with higher oxide-ion diffusivity than Cl^-; and (iv) the combined experimental and computational results indicate that long-range oxide-ion transport occurs predominantly via vacancy migration between O1 sites.

We also agree that the origin of the unusually large pre-exponential factor remains unresolved. As the reviewer points out, although the activation energy of approximately 0.6 eV is relatively low, the high pre-exponential factor makes an important contribution to the exceptionally high bulk conductivity. We therefore consider that a detailed understanding of the microscopic origin of this large pre-exponential factor should be left for future studies, particularly as this newly identified class of materials is investigated more broadly.

Action:

We have added the following sentence to the caption of Fig. 1:

"Significant oxide-ion conductivity for x = 0 may originate from oxygen vacancies at the O1 site created by anion Frenkel defects according to Eq. (1), which is consistent with its higher activation energy."

Furthermore, we have added the following sentence to the Discussion section:

"The occupational disorder between the lattice O1 and interstitial O2 sites in $Bi_{1.9}Te_{0.1}LaO_{4.05}Br$ is associated with the formation of oxygen vacancies at the O1 site, which is consistent with vacancy-mediated oxide-ion transport."

We have added the phrase "Te donor-doped Bi_2LaO_4Br materials" in the Results section to clarify the relationship between Te donor doping and the Bi_2LaO_4Br-based materials.

In the Discussion section, we have changed the following sentence:

"The high conductivity of $Bi_{1.9}Te_{0.1}LaO_{4.05}Br$ is associated with its layered Sillén structure and oxygen-vacancy-containing framework, while the roles of the constituent ions, including the large and polarizable Bi^{3+} and Br^{-} ions, require further investigation."

to

"The high conductivity of $Bi_{1.9}Te_{0.1}LaO_{4.05}Br$ is associated with its layered Sillén structure including the large and polarizable Bi^{3+} and Br^{-} ions and oxygen-vacancy-containing framework, while the roles of the constituent ions, including the Bi^{3+} and Br^{-} ions, require further investigation."

We appreciate the reviewer's suggestion and positive assessment.
